# Green by Design: Convergent Synthesis, Computational Analyses, and Activity Evaluation of New FXa Inhibitors Bearing Peptide Triazole Linking Units

**DOI:** 10.3390/pharmaceutics14010033

**Published:** 2021-12-24

**Authors:** Diego F. Rodríguez, Francisca Durán-Osorio, Yorley Duarte, Pedro Olivares, Yanina Moglie, Kamal Dua, Flavia C. Zacconi

**Affiliations:** 1 Facultad de Química y de Farmacia, Pontificia Universidad Católica de Chile, Santiago 7820436, Chile; dfrodriguez1@uc.cl (D.F.R.); fnduran@uc.cl (F.D.-O.); 2 Center for Bioinformatics and Integrative Biology, Facultad de Ciencias de la Vida, Universidad Andrés Bello, Santiago 8370035, Chile; yorley.duarte@unab.cl (Y.D.); pedropablo.olivares@gmail.com (P.O.); 3 Departamento de Química INQUISUR, Universidad Nacional del Sur (UNS)-CONICET, Bahía Blanca 8000, Argentina; 4 Discipline of Pharmacy, Graduate School of Health, University of Technology Sydney, Ultimo, NSW 2007, Australia; kamal.dua@uts.edu.au; 5 Faculty of Health, Australian Research Centre in Complementary and Integrative Medicine, University of Technology Sydney, Ultimo, NSW 2007, Australia; 6 Institute for Biological and Medical Engineering, Schools of Engineering, Medicine and Biological Sciences, Pontificia Universidad Católica de Chile, Santiago 7820436, Chile; 7 Centro de Investigaciones en Nanotecnología y Materiales Avanzados, CIEN-UC, Pontificia Universidad Católica de Chile, Santiago 7820436, Chile

**Keywords:** FXa inhibitors, DOACs, green chemistry, microwave synthesis, click chemistry, drug discovery, Ullmann-Goldberg reaction

## Abstract

Green chemistry implementation has led to promising results in waste reduction in the pharmaceutical industry. However, the early sustainable development of pharmaceutically active compounds and ingredients remains a considerable challenge. Herein, we wish to report a green synthesis of new pharmaceutically active peptide triazoles as potent factor Xa inhibitors, an important drug target associated with the treatment of diverse cardiovascular diseases. The new inhibitors were synthesized in three steps, featuring cycloaddition reactions (high atom economy), microwave-assisted organic synthesis (energy efficiency), and copper nanoparticle catalysis, thus featuring Earth-abundant metals. The molecules obtained showed FXa inhibition, with IC_50_-values as low as 17.2 μM and no associated cytotoxicity in HEK293 and HeLa cells. These results showcase the environmental potential and chemical implications of the applied methodologies for the development of new molecules with pharmacological potential.

## 1. Introduction

The factor Xa (FXa) is a serine protease playing a pivotal role in the activation of the blood clotting cascade. This enzyme links the intrinsic and extrinsic coagulation pathways, catalyzes thrombin production and amplifies the initial response of the coagulation process [1]. Recently, a new generation of direct FXa inhibitors for the treatment and prevention of thromboembolic disorders has emerged: DOACS, direct oral anticoagulants (apixaban, rivaroxaban, edoxaban and betrixaban), applied for the treatment of deep vein thrombosis, stroke, and pulmonary embolism [2]. Additionally, recent research has revealed the increased risk of thromboembolic manifestations associated with COVID-19 caused by severe acute respiratory syndrome coronavirus 2 (SARSs-CoV-2) [3]. However, the underlying mechanisms by which COVID-19 induces an increase in the procoagulant state leading to thrombosis are still unknown [4]. The need to generate more effective therapies against COVID-19, and the urgency to reduce the cases of mortality, have promoted various studies that evaluate the effectiveness of anticoagulant therapy for thromboprophylaxis in patients with COVID-19 [5]. Nevertheless, direct FXa inhibitors lead to important side effects, including a significant increase in gastrointestinal bleeding [6], drug interaction [7] and skin toxicity [8,9]. In addition, their synthesis has severe limitations, including the need of numerous synthetic steps in general and the use of toxic and dangerous reagents and solvents [10,11]. Therefore, the design and sustainable synthesis of new FXa inhibitors is absolutely necessary.

The crystal structures of the FXa and FXa-ligand complexes present valuable information for the design of new inhibitors. From this perspective, the S4 and S1 pockets in the active site of FXa are central inhibitor recognition regions.

**Figure 1:**
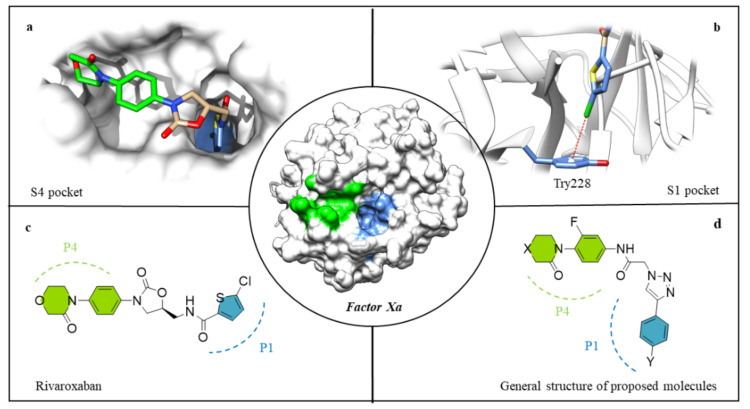
Rivaroxaban interaction in the S4 pocket (**a**), rivaroxaban interaction in the S1 pocket (**b**), chemical structure of rivaroxaban (**c**), general chemical structure of the molecules synthesized in this work (**d**). In the FXa, the blue fragment represents S1 and the fragment in green S4, respectively.

The S4 pocket of FXa is a hydrophobic area formed by the Tyr99, Thr98, Phe174 and Trp215 entities. The presence of aromatic rings and their respective π-electrons makes this an attractive region for the binding of hydrophobic and positively charged functional groups [12,13]. This characteristic structural motif has been exploited in the design of diverse FXa inhibitors, in which their P4 ring (generally an aryl group) was located between the aromatic side chains of Tyr99 and Phe174 (Figure 1a, highlighted in green) [14].

S1 is a deep anion pocket formed by Trp215, Gly216 and Ala190, Cys191, Gln192, with Asp189 and Try228 residues at the bottom side [15]. This anionic character of the S1 pocket allowed for the early design of potent FXa inhibitors using benzamidine/amidine motifs [16]. However, the developed class of compounds was later replaced due to their low bioavailability and inadequate pharmacokinetics [17]. The current strategy uses several types of specific interactions for the design of neutral groups with high affinities to the S1, e.g., halogen bonding, which has been described as essential in FXa inhibitors displaying a halo-aryl group [18,19,20]. For example, the structure of the FXa-rivaroxaban complex revealed a halogen–π interaction between the chlorine of the thiophene group, and the Tyr228 residue located in the lower part of the S1 pocket (Figure 1b, highlighted in blue).

**Figure 2:**
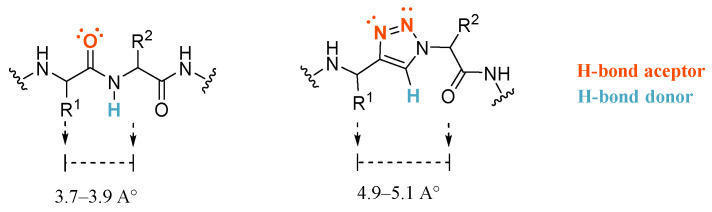
1,2,3-triazoles 1-4-disubstitutes as an isostere of *trans*-amide bonds.

Regarding the linker, different structures have been described to connect the fundamental P1 and P4 motives. FXa inhibitors based on peptides showed high affinities, although the low stability in vivo hampered their potential, eventually leading to the evolution of peptide-like inhibitors with an amide bond alternative [21,22]. Bioisosteric amide exchange has become a robust methodology for the partial or total replacement of the peptide bond. The use of 1,2,3-triazoles as a peptidomimetic motif has proven to improve stability without decreasing biological activity (Figure 2). [23,24] Furthermore, the synthesis of 1,2,3-triazoles via copper-catalyzed click-type Huisgen 1,3-dipolar cycloaddition presents considerable advantages, such as high regioselectivity and atom economy, mild reaction conditions, few byproducts, and simple purification procedures, rendering click chemistry an excellent tool for drug discovery and green chemistry [25,26].

The development and design of new sustainable methodologies for green organic syntheses have become increasingly important in recent years [27]. This is especially relevant for drug discovery and the pharmaceutical industry (which commonly shows the highest E-factors) [28,29]. Accordingly, different companies have already implemented the 12 Principles of Green Chemistry in the synthesis of active pharmaceutical ingredients (APIs), reducing the associated environmental impact [28]. However, the implementation of the green chemistry principles is still a big challenge, and there are only few reports about the thorough implementation of the latter in the early steps of research towards APIs. Although there are different studies which focus on the green design of inhibitors [30], it remains relatively underexplored, likewise in the design and synthesis of FXa inhibitors. 

**Figure 3:**
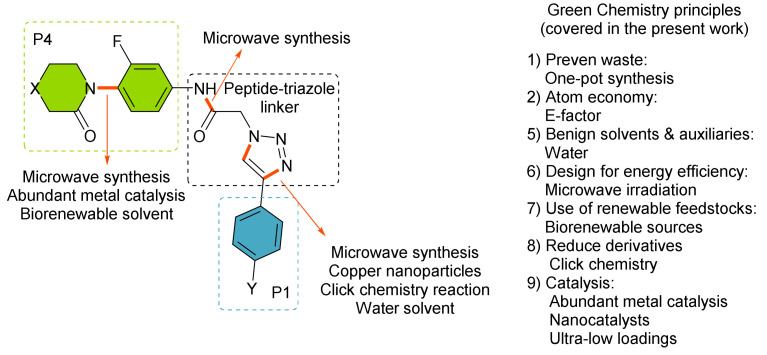
General structure of the molecules designed from this work.

Here, we report the synthesis of a new series of molecules with promising activity as direct FXa inhibitors, while considering the Principles of Green Chemistry (Figure 3, low catalyst loading, copper nanoparticles CuNPs, green solvents, microwave-assisted chemistry and one-pot procedures) [27]. 

## 2. Materials and Methods

### 2.1. Chemistry

Anhydrous copper (II) chloride (Merck, Kenilworth, NJ, USA), lithium powder, 4,4′-di-*tert*-butylbiphenyl (DTBB, Merck), activated charcoal (Norit CA1, Merck) and sodium azide (Merck) were purchased from the mentioned suppliers. All starting alkynes, amines, and fluoro-anilines were purchased from suppliers offering the best grade (Sigma-Aldrich, Saint Luis, MO, USA; Alfa Aesar, Ward Hill, MA, USA; AK Scientific, Union City, CA, USA; Ambeed, Arlington Heights, IL, USA) and used without further purification.

The reactions requiring anhydrous conditions were carried out under nitrogen atmosphere and the solvents were appropriately dried before use, except for 2-methyltetrahydrofuran (2-MeTHF), which was acquired in its anhydrous form. Ullman-Goldberg-type reactions, copper-catalyzed azide-alkyne cycloadditions (CuAAC), and peptide couplings were placed in a microwave Synthesis Reactor Monowave 200 (Anton Paar, Graz, Austria). All reactions were monitored by thin-layer chromatography (TLC). Column chromatography purifications were performed using standard silica gel (230–400 mesh; Sigma-Aldrich). The melting points of solid derivatives were measured using an Electrothermal IA9100 digital melting point apparatus (Cole-Palmer, Staffordshire, UK). NMR spectra were recorded on Bruker Advance 300 (precursors **6-a**, **d**, **e** and **7-d**, **e**) and 400 spectrometers (300 and 400 MHz for ^1^H NMR, 75 and 100 MHz for ^13^C NMR, 376 MHz for ^19^F NMR) (Bruker, Billerica, MA, USA), with CDCl_3_ as a solvent, if not indicated otherwise. The NMR data are reported in δ (ppm) referenced from tetramethylsilane (TMS). IR spectra were recorded in KBr pellets on a Bruker Vector 22 spectrophotometer. High-resolution mass spectra (HRMS) were recorded on a Thermo Scientific Exactive Plus Orbitrap (Thermo Scientific, Walthman, MA, USA).

General procedure of Ullmann-Goldberg reaction (**3a–d**)

A mixture of CuI (0.5 mmol, 95.0 mg), 3-fluoro-4-iodoaniline (3.0 mmol, 711 mg), δ-valerolactame (2.5 mmol, 248 mg), K_3_PO_4_ (5.0 mmol, 1061 mg), *N,N’*-dimethylethylenediamine (DMEDA) (1.0 mmol, 108 μL) and anhydrous 2-MeTHF (6 mL) was placed in the microwave reactor (Synthesis Reactor Monowave 200, Anton Paar) and irradiated continuously with initial power (850 watts) at 120 °C for 2 h. The crude residue was purified by flash column chromatography on hand-packed columns, with silica gel and hexane/ethyl acetate as the solvent mixture, to obtain the corresponding isolated arylamines.

Procedure for the preparation of CuNPs/C

Anhydrous copper (II) chloride (135 mg, 1 mmol) was added to a suspension of lithium powder (14 mg, 2 mmol) and 4,4′-di-*tert*-butylbiphenyl (DTBB, 27 mg, 0.1 mmol) in THF (2 mL) at room temperature under nitrogen atmosphere. The reaction mixture was initially dark blue and rapidly changed to black (ca. 5–10 min), indicating that the suspension of copper nanoparticles was formed. This suspension was diluted with THF (8 mL), followed by the addition of activated carbon (800 mg). The resulting mixture was stirred for 3 h at room temperature, filtered, and the solid successively washed with water (20 mL) and diethyl ether (20 mL), and successively dried under vacuum (15 Torr). 

General procedure for the CuNPs/C-catalyzed multicomponent CuAAC reaction in water (**6a–e**)

A mixture of NaN_3_ (0.7 mmol, 45.5 mg), ethyl-2-bromoacetate (0.5 mmol, 83.5 mg, 55.7 μL), and ethynylbenzene (0.5 mmol) was added to a suspension of CuNPs/C (20 mg, 0.5 mol% Cu) in H_2_O (2 mL) and placed in the microwave reactor (Synthesis Reactor Monowave 200, Anton Paar) and irradiated with initial power (850 watts) at 85 °C for 30 min. Water (30 mL) was added to the resulting mixture, followed by extraction with EtOAc (3 × 10 mL). The collected organic phases were dried with anhydrous MgSO_4_ and the solvent removed in vacuo to give the corresponding triazole.

General procedure for the hydrolysis of ethyl ester derivatives (**7a–e**)

Ethyl-2-(4-phenyl-1*H*-1,2,3-triazol-1-yl)acetate (0.5 mmol, 115.6 mg) was dissolved in methanol and an aqueous solution of NaOH (1 mL, 2M) was added. The reaction vessel was placed in the microwave reactor (Synthesis Reactor Monowave 200, Anton Paar) and irradiated with initial power (850 watts) at 120 °C for 30 min. Water (30 mL) was added to the resulting mixture, which was acidified (pH = 3) using HCl (1 M) and extracted with ethyl acetate (3 × 10 mL). The unified organic phases were dried with anhydrous MgSO_4_, and the solvent was removed in vacuo to give the corresponding acids.

General procedure of the peptide coupling reaction (**8a–t**)

A mixture of HATU (0.5 mmol, 190.1 mg), EDC (0.5 mmol, 95.9 mg), and 2-(4-phenyl-1*H*-1,2,3-triazol-1-yl)acetic acid (**7a**) (0.5 mmol, 101.6 mg) was weighed and transferred into a dried reaction tube. The tube was evacuated and backfilled with dry N_2_. The reaction was stirred at 0 °C for 1 h in dry dichloromethane (DCM) (8 mL). Subsequently, the 1-(4-amino-2-fluorophenyl)piperidin-2-one (**3a**) (0.65 mmol, 135.4 mg) and pyridine (Py) (0.5 mmol, 40 μL) were added, and the reaction was placed in the microwave reactor (Synthesis Reactor Monowave 200, Anton Paar) and irradiated with initial power (850 watts) at 70 °C for 30 min. The remaining mixture was washed once with an aqueous solution of citric acid (10% *w*/*w*), saturated aqueous NaHCO_3_, brine and was dried over anhydrous MgSO_4_. The solvent was removed under vacuum, and the remaining product purified by flash column chromatography on silica gel, with n-hexane/ethyl acetate as the solvent mixture.

### 2.2. FXa In Vitro Inhibition Assay

The final compounds were evaluated in vitro for their FXa enzyme inhibitory activity using the fluorometric Factor Xa Assay Kit (SensoLyte^®^ Rh 110 factor Xa Assay Kit *Fluorometric*, Anaspec, Fremont, CA, USA). The assay was prepared according to the instructions. The synthesized compounds **8a–t** and the positive controls, rivaroxaban (gold standard inhibitor) and Gabexate mesylate (FXa kit inhibitor), were dissolved in DMSO at a concentration of 10 mM and then screened at 100 μM. The 96-well plate for fluorescence testing was prepared with 40 μL of enzyme and 10 μL of each compound, incubated for 10 min at 37 °C, and the enzymatic reaction was initiated by the addition of 50 μL of the FXa substrate (FXa, S-2222). The molecules with percentages of inhibition over 50% were evaluated in a dilution series spanning over a range of 1 nM to 100 μM to determine the corresponding IC_50_ values. The statistical analysis of triplicates was made with GraphPad Prism v. 8.0.2 (GraphPad Inc., San Diego, CA, USA).

### 2.3. Computational Analyses 

The computations were performed using Schrödinger’s Small-Molecule Drug Discovery Suite. The initial setup of the FXa enzyme structure (PDB: 2P16) was prepared using Protein Preparation Wizard of Schrödinger [31,32] to add hydrogens, assign bond orders and generate rotamers and protonation states. Compound structures were drawn with ChemDraw (PerkinElmer, Waltham, MA, USA) and prepared using the software LigPrep [33], while ionization/tautomeric states were predicted using Epik [34]. The protein was subjected to a molecular minimization with the Impref module of impact.

The docking calculations using rigid-receptor and flexible-ligands were performed with Glide through the Single Precision (SP) mode [35,36]. The docking grid box was centered on the co-crystallized apixaban to define the center of the grid box. The docking poses for each molecule were analyzed by using the Emodel score and their interaction with residues at binding sites. The three most energetically favorable conformations were selected as the best poses.

### 2.4. Molecular Dynamics Simulations (MD)

Four independent MD simulations were performed using the Desmond program in the Schrödinger suite. Each protein–ligand complex was solvated using explicit TIP3P water models and an orthorhombic box with periodic boundary conditions. All complexes were neutralized with 0.15 mol L^−1^ of NaCl and parametrized with OPLSe force field. Each simulation was performed for a total of 200 ns, with a recording interval of 100 ps. NPT ensemble at the standard conditions of T = 310 K and P = 1 atm were used.

### 2.5. Cell Viability Analysis 

The human cell lines (HEK293 and HeLa) applied in the cell viability assays were purchased from ATCC (Manassas, VA, USA). HEK293 and HeLa cell lines were cultivated in Dulbecco’s modified Eagle medium, supplemented with 10% fetal bovine serum, 100 units/mL of penicillin and 100 mg/mL of streptomycin and kept at 37 °C in a humidified atmosphere containing 5% CO_2_. A 100-μL aliquot of adherent cells was used to seed 96-well cell culture plates at 25,000 cells/well and allowed to adhere for 16 h prior to compound addition. Cells were treated with each compound in DMEM containing 10% FBS for 48 h. Subsequently, 10 μL of Cell Counting Kit-8 reagent (CCK-8; Sigma-Aldrich, Merck KGaA, Darmstadt Germany) was added into each well and incubated for 4 h at 37 °C in a humidified atmosphere containing 5% CO_2_. The absorbance measurement detected the cell viability at 450 nm in a Synergy H1 microplate reader (Biotek Instruments, Winooski, VT, USA). All experiments were performed in triplicate.

## 3. Results and Discussion

### 3.1. Synthesis and Optimization

**Scheme 1:**
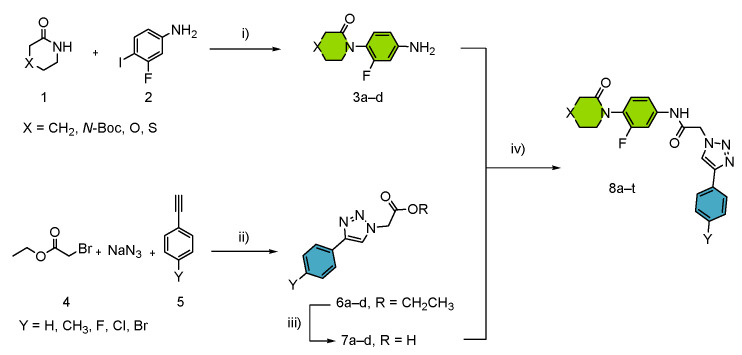
General procedure for the synthesis of compounds **8a–t**. **i**) Reaction conditions: **1** (2.5 mmol, 248 mg), **2** (3.0 mmol, 711 mg), K_3_PO_4_ (5.0 mmol, 1061 mg), CuI (0.5 mmol, 95 mg), DMEDA (1.0 mmol, 108 μL), 2-MeTHF (6 mL), under N_2_ atmosphere, 120 °C, MW, 2 h. **ii**) **4** (0.5 mmol, 55 μL), **5** (0.5 mmol), NaN_3_ (0.7 mmol, 45 mg), CuNPs/C (0.5 mol%, 20 mg), solvent (2 mL), 120 °C, MW, 30 min. **iii**) **6** (0.5 mmol), solvent (2 mL, H_2_O:CH_3_OH 1:1), NaOH (1 mL, 2 M), 120 °C, MW, 30 min. **iv**) **3** (0.65 mmol), **7**(0.5 mmol), HATU (0.5 mmol, 190 mg), EDC (0.5 mmol, 96 mg), Py (0.5 mmol, 40 μL), DCM (8 mL), 70 °C, MW, 30 min. Structure **8a–t**: green colour: P1 enzyme interactions; blue colour: P4 enzyme interactions.

The synthetic route for the peptide-1,2,3-triazole derivatives **8a–t** is represented in Scheme 1. The planned convergent synthesis brought the possibility of a rapid and straightforward access of the general inhibitor structure. For development purposes, the process was divided into three steps:

(i) Synthesis of the aryl-amide motive P1 by chemoselective copper-catalyzed Ullmann-Goldberg reactions in a benign solvent.
(ii) Synthesis of the triazole linker and P2 motif by a multicomponent click chemistry reaction catalyzed by carbon-supported copper nanoparticles CuNPs/C.
(iii) Efficient microwave-assisted synthesis of final products through peptide coupling.

**Scheme 2:**
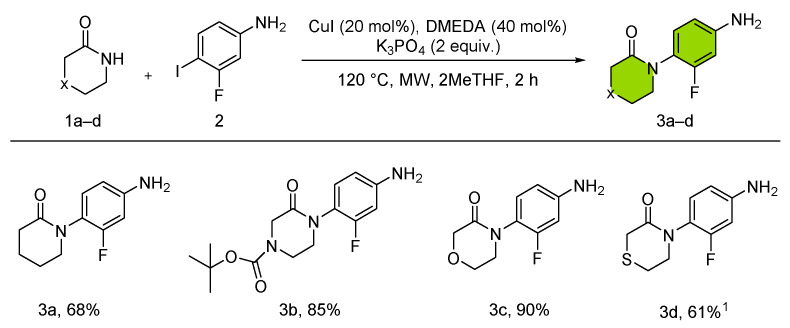
Modified Ullmann-Goldberg reaction between lactam derivatives **1a–d** and 3-fluoro-4-iodoaniline, leading to arylamines **3a–d**. ^1^ MW, 140 °C.

We started our investigation with the optimization of the modified Ullmann-Goldberg reaction using δ-valerolactame (**1a**) and 3-fluoro-4-iodoaniline (**2**) as model substrates (Table S1). Under optimized reaction conditions, we decided to replace toxic toluene with a green alternative solvent. Among the bio-based solvents, 2-MeTHF represents a viable alternative for polar aprotic solvents [37,38]. The reaction in 2-MeTHF furnished the product **3a** in 68% yield. This solvent allowed us to significantly increase the reaction yield in comparison with the use of toluene as a solvent. The methodology could be successfully extended to lactam derivatives with different heteroatoms in 4-position (Scheme 2).

The copper-catalyzed azide-alkyne cycloaddition (CuAAC) is a powerful tool for the synthesis of diverse drug candidates. Multicomponent CuAAC reactions have some essential advantages over conventional reactions, such as the generation of organic azides from corresponding halides in situ, eschewal of purification steps, waste reduction and implementation of more user-friendly and safer conditions in general [39]. We tested different methodologies to obtain compound (**6a**), starting from ethyl-2-bromoacetate (**4**), phenylacetylene (**5a**) and sodium azide. The standard conditions of the multicomponent reaction were used (Cu/CuSO_4_ in H_2_O:*t*-BuOH under microwave heating at 125 °C) [39]. However, the reaction only resulted in a yield of 55%. Hence, we changed our focus towards more efficient catalysts, specifically copper nanoparticles supported on activated carbon CuNPs/C (which presents high reactivity at low catalyst loadings) [40,41]. Upon applying the nanocatalyst, the corresponding triazole (**6a**) could be obtained in 72% yield after only 30 min of reaction time, with water as a solvent and without the need for further additives. The optimized reaction conditions (see Table S2) were extended to differently substituted phenylacetylenes **5**. The products **6b–e** were obtained in moderate to good yields (68–84%), avoiding the need for purification by column chromatography. The corresponding acids (**7a–e**) were subsequently obtained by basic hydrolysis with aqueous NaOH under microwave heating at 120 °C for 30 min.

**Scheme 3:**
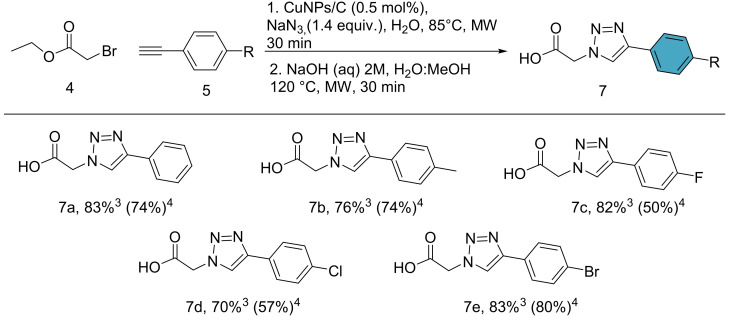
One-pot multicomponent CuAAC and hydrolysis reaction. Reaction conditions: (**1**) **4** (0.5 mmol), **5a** (0.5 mmol), NaN_3_ (0.7 mmol), CuNPs/C (0.5 mol%, 20 mg), H_2_O:MeOH (2 mL, 1:1), 85 °C, MW, 30 min. (**2**) NaOH solution (1 mL, 2 M), 120 °C, MW. (**3**) Yield of the one-pot multicomponent click reaction/basic hydrolysis. (**4**) Yield of the multicomponent click reaction and subsequent basic hydrolysis with intermediary ester purification. Yields indicate isolated products.

One-pot reactions are a very efficient methodology for the reduction of waste in chemical transformations, avoiding additional steps of purification. Therefore, we turned our attention to a one-pot approach of the multicomponent click reaction and the subsequent hydrolysis. We started off with a mixture of H_2_O:MeOH as solvent to avoid the triazole esters being trapped inside of the catalyst in the first step (Figure S1 from Supplementary Materials). The second step is initiated by the addition of NaOH solution and microwave heating directly. The results of the one-pot procedure are summarized in Scheme 3, where the products **7a–e** were furnished with yield increases of up to 30%, as compared to the interrupted process with intermediate ester purification.

**Scheme 4:**
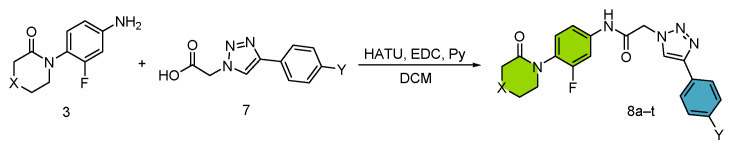
Synthesis of new peptide triazole **8a–t**.

The final step of the synthesis of the amide products had to be an efficient peptide-coupling protocol. The various aryl amines **3a–d** and triazole acids **7a–e** were successfully coupled using HATU/EDC as activating agents and pyridine as a base at room temperature, furnishing products **8** in moderate to good yields (Scheme 4). 

The same reaction conditions using microwave heating (70 °C) instead boosted the yields into the good to excellent range, while reducing the reaction time by up to 96% (see Table S3). Hence, the microwave-assisted methodology was applied for all products (Table 1).

### 3.2. In Vitro FXa Inhibition Analysis

The FXa inhibition capacities of the newly synthesized molecules were evaluated using the SensoLyte^®^ 520 Factor Xa Assay Kit. The FXa inhibition begins after cleavage of the FXa protease from the chromogenic substrate (S-2222), which generates rhodamine 110 (Rh110) as a free fluorophore and can be detected at an excitation/emission of 490 nm/520 nm. The FXa inhibition values and several IC_50_ values of compounds **8a–t** are summarized in Table 2.

Interestingly, the FXa inhibition results indicated that twenty percent of the newly synthesized peptide triazoles gave inhibition percentages greater or equal to 50%. Moreover, these compounds (**8a**, **8k**, **8l** and **8p**) showed IC_50_ values in the range of 17.2–76.2 μM, suggesting moderate inhibitory efficiency against the FXa blood protein. It should be noted that the peptide triazoles do not present pan-assay interference compound (PAINS) fragments in their structure.

### 3.3. Computational Analysis 

#### 3.3.1. Molecular Docking

**Figure 4:**
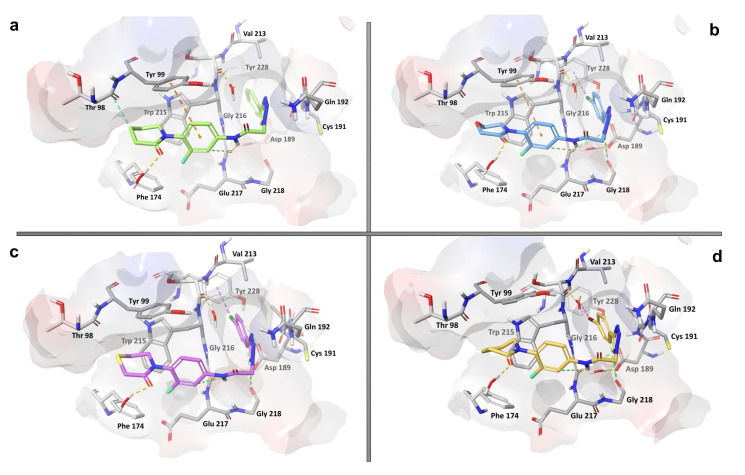
Molecular docking poses of active peptide-1,2,3-triazole derivatives **8a, 8k, 8l** and **8p** (**a**–**d**) into the active site of the FXa enzyme (PDB: 2P16). The dotted lines indicate the most common ligand–protein interactions: H bonds in yellow, π–π stackings in orange, halogen–π interactions in magenta and aromatic H bonds in green.

Considering the biological activity exhibited and the structural similarity present among the synthesized molecules, the molecular docking calculations were carried out for the four compounds showing the highest activity against FXa to elucidate the molecular basis of this behavior. First, graphical inspection of the molecular docking results was carried out for all compounds, and then the explanation of atomic level molecular interactions of these ligands with the enzyme was intended (Figure 4). 

Compounds **8a, 8k, 8l** and **8p** were docked to the FXa enzyme binding site, displaying binding energy values of −9.59, −9.76, −8.90 and −9.82 kcal/mol, respectively, being slightly superior to rivaroxaban (−8.81 kcal/mol). The molecular docking analysis showed similar interactions between all docked ligands and the FXa binding site residues. 

Compound **8p**, which exhibited the highest activity against FXa (IC_50_ = 17.2 μM), established a hydrogen bond interaction between the NH group of the peptide linker and the C=O group of the Gly216 residue with a length of 3.17 Å (Figure 4d). Other interactions resulting from aromatic rings in the ligand **8p** are aromatic H bonds with Gly216 (3.22 Å), Gly218 (3.69 Å) and Asp189 (3.69 Å). The aromatic H bond plays an important role for the stability of several proteins if donors and acceptors occur at successive turns [42]. Another relevant interaction, though not perfectly visualized in the structural conformation, is represented by hydrophobic T-shaped π–π interactions of the central aromatic moiety of compound **8p** with the Tyr99 residue (<5.0 Å). In addition, the presence of Br in compound **8p** in the *p* position of the phenyl ring establishes a halogen–π interaction with the Tyr228 residue (3.74 Å) located on one side of the S1 pocket (Figure 4d). The halogen–π interaction has been described for some FXa inhibitors already, such as the anticoagulant rivaroxaban, featuring Cl and Br atoms in their structure [18,43]. The specific alignment of rivaroxaban in the active site allows for the chlorine on the thiophene moiety to interact with the Tyr228 residue in the S1 pocket, mainly through noncovalent halogen–π interactions. This interaction has been described as key for the ligand binding and recognition to the enzyme representing a similar relevance to halogen bond interactions [44,45]. A common halogen–π bond describes the aromatic rings as donors and the C–X bonds (with X = F, Cl, Br, I) as acceptors [46]. This characteristic is perfectly in line with the conformations adopted by compounds **8p**, **8k** and **8l** with residue Tyr228 of S1.

Similarly, the enzyme–ligand interactions for the other three active compounds **8a**, **8k** and **8l** are governed by H bonds between the NH group of the linker with Gly216 and T-shaped π–π contacts of the central aromatic portions and the Tyr99 residue, with distances of less than 3.00 Å and 5.12 Å, respectively. In addition, they exhibit other noticeable interactions, as well such as Van der Waals and electrostatic contacts with the amino acid residues Gly216, Gly218, Phe174 and Asp189. Compounds **8k** and **8l** also engage in halogen–π interactions with residue Tyr228 at distances of 3.91 Å and 3.88 Å, respectively. The differences in length of the halogen–π interaction and the Tyr228 residue are not only related to the structural conformations adopted by each compound, but also to the exact type of halogen atom present, since distances and bonding forces increase with their molecular weight (F < Cl < Br < I) [46]. The performed docking studies showed that the lactam ring of all compounds exhibited a Van der Waals interaction with Thr98 and Phe174, while the heteroatom is solvent-exposed. Therefore, the change of the heteroatom in this moiety of the molecule did not have any effect on amino acid residues, although the C=O group presented a stable H bond with a water molecule. The molecular docking studies showed that the active compounds that successfully docked into the FXa enzyme displayed similar behaviors as compared to rivaroxaban, mainly H bondings with Gly216, hydrophobic interactions (T-shaped π–π and aromatic H bond type) with Tyr99 and Gly218 and finally, halogen–π interactions with Tyr228.

#### 3.3.2. Molecular Dynamics Simulation

**Figure 5:**
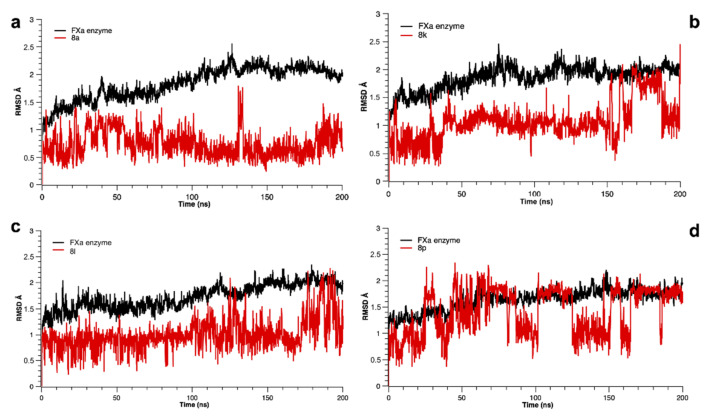
Root mean square deviation (RMSD) as a function of simulated times for the complexes formed between FXa and the active compounds **8a**, **8k**, **8l** and **8p** (indicated by **a**–**d**, respectively).

Molecular dynamics (MD) simulations of 200 ns were performed to gain more detailed structural insights into the enzyme-ligand interactions and to evaluate the differences of the binding mode between the four most active compounds **8a, 8k, 8l** and **8p** and the FXa enzyme. The root mean square deviation (RMSD) analysis revealed a generally high stability of the protein-ligand complexes during simulation (Figure 5). The compounds did not show any significant changes in RMSD values, fluctuating between 0.5 and 2.0 Å. A minor instability in trajectories of the **8p**–FXa complex could be observed starting from 40 ns, where the RMSD analysis showed an oscillation of about ∼2.0 Å, but reaching final equilibration after 160 ns of simulation time. Nevertheless, such slight fluctuations do not interfere with the overall system stability, since deviations of less than 3.0 Å indicate an equilibrium of the ligand and the active site [47]. Despite a pronounced rotation of the peptide-1,2,3-triazole derivatives, the main interactions shown in molecular docking are preserved for all complexes over the simulation period, highlighting the participation of π–π stacking interactions between the central aromatic moiety of the substrates and the residues Tyr99 and Trp215, respectively. Furthermore, the hydrogen bonding interactions between the peptide linker and the residues Gly216 and Gly218 of the active site, as well as the halogen–π interaction with the aromatic ring of Tyr228 in the S1 pocket could be observed. These residues were the main stabilizing element between the compounds and the active site of the protein. Finally, although compound **8p** showed a certain degree of instability through its flexible interactions, this compound engaged in a stable hydrogen bond with the Arg143 residue during more than 90% of the trajectory time, contributing significantly to the stabilization inside the binding site. 

### 3.4. Cell Viability Analysis 

**Figure 6:**
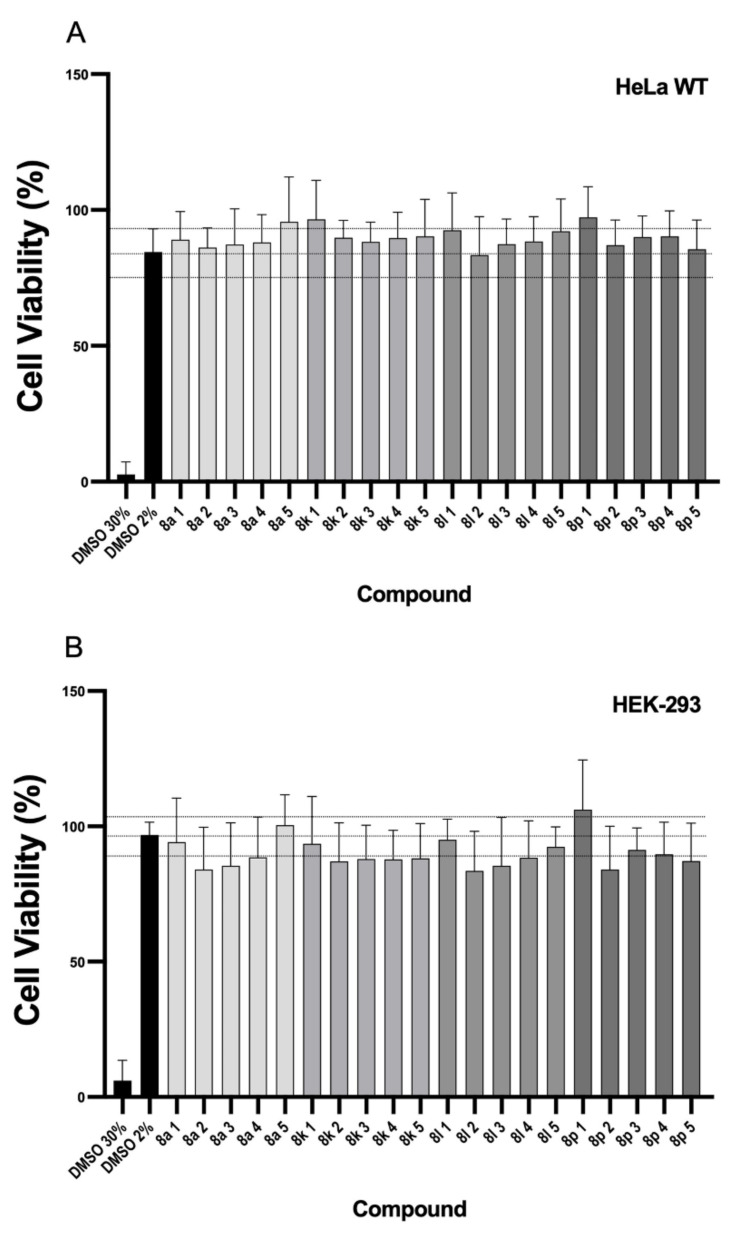
Effect of the selected compounds on cell viability using the CCK-8 assay in (**A**) HeLa and (**B**) HEK93 cell lines. Concentrations used were 10 mM (1), 100 μM (2), 10 μM (3), 100 nM (4), and 10 nM (5). The upper and lower lines define ± 2 SD limits from the average value of control wells treated only with the vehicle (DMSO 2%). All values are means ± SD (*n* = 3). SD: standard deviation.

Cell viability assays were performed with HeLa and HEK293 cells using the CCK-8 method. Compounds **8a**, **8k**, **8l** and **8p** were assayed with decreasing concentrations: 10 mM (1), 100 μM (2), 10 μM (3), 100 nM (4) and 10 nM (5) (Figure 6). In the HeLa cell strain no signs of toxicity were observed with any compound, as defined by the value obtained after subtracting two standard deviations of the average value of control wells treated with a blank only (culture medium with 2% DMSO). Similarly, HEK-293 cells did not show any cell mortality in the studied ranges of concentration with any of the compounds. These results indicate a potential applicability of the compounds as FXa inhibitors without a prejudicial degree of toxicity.

### 3.5. Drug Likeness Prediction 

All active compounds have favorable predicted in silico values of pharmacokinetic properties, indicating their potential as anticoagulants. Molecular descriptors were determined using QikProp of Schrödinger (Table 3) [48].

## 4. Conclusions

The development of new and sustainable synthetic methodologies to access active pharmacological molecules that fulfil one or several of the Principles of Green Chemistry is a great challenge today. In the present work, we demonstrated that applying these principles in the first steps of the development of new pharmaceutically active compounds is a reliable methodology and could lead to a shift in the pharmaceutical industry towards a more sustainable future. The newly synthesized FXa inhibitors presented IC_50_ values of 17.2–76.2 μM and were obtained in a convergent three-step synthesis. A microwave-assisted key step using CuNPs and biorenewable solvents gave access to the new derivatives in only 3.5 h reaction time. The peptide triazoles presented very adequate drug-like properties, the binding mode of the FXa inhibitors was thoroughly studied by molecular docking experiments, and successful molecular dynamics simulations validated the design of the synthesized structures. Finally, the cell viability analysis demonstrated the safety of the new FXa inhibitors in different cell types, such as HEK293 and HeLa.

## Figures and Tables

**Table 1:** Microwave-assisted amide synthesis ^1^.

Entry	Nomenclature	X	Y	Yield (%) ^2^
1	**8a**	CH_2_	H	50
2	**8b**	*N*-Boc	H	70
3	**8c**	O	H	43
4	**8d**	S	H	82
5	**8e**	CH_2_	F	19
6	**8f**	*N*-Boc	F	62
7	**8g**	O	F	52
8	**8h**	S	F	42
9	**8i**	CH_2_	Cl	19
10	**8j**	*N*-Boc	Cl	69
11	**8k**	O	Cl	27
12	**8l**	S	Cl	51
13	**8m**	CH_2_	Br	27
14	**8n**	*N*-Boc	Br	56
15	**8o**	O	Br	66
16	**8p**	S	Br	83 (44) ^3^
17	**8q**	CH_2_	CH_3_	30
18	**8r**	*N*-Boc	CH_3_	57
19	**8s**	O	CH_3_	31
20	**8t**	S	CH_3_	57

^1^ Reaction conditions: **3** (0.65 mmol), **7** (0.5 mmol), HATU (0.5 mmol), EDC (0.5 mmol), Py (0.5 mmol), solvent (8 mL), N_2_ atmosphere, 70 °C, MW, 30 min. ^2^ Yields of isolated products after column chromatographic. ^3^ Yield using 2-MeTHF as solvent under optimized conditions.

**Table 2:** FXa inhibition and IC_50_ values of novel compounds **8a**–**t**.

Entry	%Inhibition FXa ^1^	IC_50_ (μM)
**8a**	68	41.2 ± 1.2
**8b**	24	-
**8c**	17	-
**8d**	33	-
**8e**	17	-
**8f**	44	-
**8g**	36	-
**8h**	25	-
**8i**	29	-
**8j**	23	-
**8k**	59	76.2 ± 16.9
**8l**	50	52.9 ± 5.5
**8m**	29	-
**8n**	22	-
**8o**	37	-
**8p**	65	17.2 ± 0.8
**8q**	21	-
**8r**	27	-
**8s**	37	-
**8t**	31	-
Gabexate mesylate	93	0.34 ± 0.10
Apixaban	97	0.0028 ± 0.0001
Rivaroxaban	94	0.0007 ± 0.0001

^1^ Inhibition percentage at 100 μM; standard inhibitor gabexate mesylate. Control samples: apixaban and rivaroxaban.

**Table 3:** Drug likeness score of most active compounds.

Compound	logPo/w	mol MW	H Bond Donors	H Bond Acceptors	logS	logBB	PMDCK	PHOA
**8a**	3.37	393.42	1.0	8.0	−5.89	−0.88	473.25	100.00
**8k**	2.82	429.84	1.0	9.7	−5.56	−0.82	879.37	92.01
**8l**	3.82	445.90	1.0	8.5	−6.85	−0.76	1663.45	100.00
**8p**	3.90	490.35	1.0	8.5	−6.89	−0.70	1982.33	100.00

logPo/w = predicted octanol/water partition coefficient. Recommended values, −2.0–6.5. H bond donors = estimated number of hydrogen bonds that would be donated in an aqueous solution. H bond acceptors = estimated number of hydrogen bonds that would be accepted in an aqueous solution. logS = predicted aqueous solubility. Recommended values, −6.5 to 0.5. logBB = predicted brain/blood partition coefficient. Recommended values, −3.0 to 1.2. Predicted apparent MDCK cell permeability in nm/sec. MDCK cells are considered to be a good mimic for the blood–-brain barrier. <25 poor, >500 great. PHOA= predicted human oral absorption on 0 to 100% scale. Recommended values, >80% is high <25% is poor.

## Supplementary Materials

The following are available online at https://www.mdpi.com/article/10.3390/pharmaceutics14010033/s1.
