# Supplementary material for: Green by Design: Convergent Synthesis, Computational Analyses, and Activity Evaluation of New FXa Inhibitors Bearing Peptide Triazole Linking Units
Source: Pharmaceutics. 2021 Dec 24;14(1):33. doi: 10.3390/pharmaceutics14010033 (PMC8780263; doi:10.3390/pharmaceutics14010033)
Supplement: Supplementary file 1 [file pharmaceutics-14-00033-s001.zip › pharmaceutics-1499950-SI.pdf]

pharmaceutics

Supplementary Materials

Green by Design: Convergent Synthesis, Computational Analyses, and Activity Evaluation of New FXa Inhibitors Bearing Peptide Triazole Linking Units

Diego F. Rodríguez [1], Francisca Durán-Osorio [1], Yorley Duarte [2], Pedro Olivares [2], Yanina Moglie [3,*], Kamal Dua [4,5] and Flavia C. Zacconi [1,6,7,*]

[1] Facultad de Química y de Farmacia, Pontificia Universidad Católica de Chile, Chile; dfrodriguez1@uc.cl (D.F.R.); fnduran@uc.cl (F.D.-O.); fzacconi@uc.cl (F.C.Z.)
[2] Center for Bioinformatics and Integrative Biology, Facultad de Ciencias de la Vida, Universidad Andrés Bello, Santiago, Chile; yorley.duarte@unab.cl (Y.D.); pedropablo.olivares@gmail.com (P.O.)
[3] Departamento de Química INQUISUR, Universidad Nacional del Sur (UNS)-CONICET, Argentina; ymoglie@uns.edu.ar
[4] Discipline of Pharmacy, Graduate School of Health, University of Technology Sydney, Ultimo, NSW 2007, Australia; kamal.dua@uts.edu.au
[5] Faculty of Health, Australian Research Centre in Complementary and Integrative Medicine, University of Technology Sydney, Ultimo, NSW 2007, Australia
[6] Institute for Biological and Medical Engineering, Schools of Engineering, Medicine and Biological Sciences, Pontificia Universidad Católica de Chile, Chile
[7] Centro de Investigaciones en Nanotecnología y Materiales Avanzados, CIEN-UC, Pontificia Universidad Católica de Chile, Chile
* Correspondence: ymoglie@uns.edu.ar (Y.M.); fzacconi@uc.cl (F.C.Z.)

Table of contents

1. *General information* 2
2. *Optimization of Ullmann-Goldberg reaction* 2
3. *Optimization of the CuNPs/C-catalyzed multicomponent CuAAC reaction in water* 3
4. *Comparison of amide synthesis by conventional heating and MW heating* 4
5. *General procedure of Ullman-Goldberg reaction (**3a-d**)* 5
6. *Procedure for the preparation of CuNPs/C* 6
7. *Characterization of CuNPs/C* 6
8. *General procedure for the CuNPs/C-catalyzed multicomponent CuAAC reaction in water (**6a-6e**)* 9
9. *General procedure for the hydrolysis of ethyl ester derivates (**7a-e**)* 10
10. *General procedure of peptide coupling reaction (**8a-t**)* 11
11. *Spectroscopic data of new synthesized compounds* 19
    11.1. *^{1}H NMR and ^{13}C NMR spectra* 19
    11.2. *^{19}F NMR spectra* 53
    11.3. *FTIR spectra* 67
    11.4. *HRMS spectra* 84

12. *In Silico Pharmacokinetics Prediction* 102

13. *References* 103

1. *General information*

Anhydrous copper (II) chloride (Merck), lithium powder, DTBB (4,4'-di-*tert*-butylbiphenyl, Merck), activated charcoal (Norit CA1, Merck) and sodium azide (Merck) are commercially available. All the starting alkynes, aminesand fluoroanilines were commercially available, and acquired in the best grade (Merck, Alfa Aesar, Ak Scientific, Ambeed) and were used without further purification.

The reactions requiring anhydrous conditions were carried out under nitrogen atmosphere and the solvents were appropriately dried before use, except for 2-MeTHF which was acquired in anhydrous form (Sigma-Aldrich, 41427). Ullman-Goldberg type reactions, copper-catalyzed azide-alkyne cycloadditions (CuAAC) and peptide couplings were placed in a microwave Synthesis Reactor Monowave 200, Anton Paar. All reactions were monitored by thin layer chromatography (TLC). Column chromatography purifications were performed using silica gel. The melting points of solid derivatives were measured using an Electrothermal IA9100 digital melting point apparatus (Staffordshire, UK). NMR spectra were recorded on Bruker Advance 300 and 400 spectrometers (300 and 400 MHz for ^{1}H NMR; 75 and 100 MHz for ^{13}C NMR, 376 MHz for ^{19}F NMR) with $CDCl_3$ as a solvent. If not indicated otherwise, the nuclear magnetic resonance (NMR) data are reported in δ (ppm) from tetramethylsilane (TMS). IR spectra were recorded in KBr pellet form, on a BRUKER VECTOR 22 spectrophotometer. High resolution mass spectra (HRMS) were recorded on a Thermo Scientific Exactive Plus Orbitrap.

2. *Optimization of Ullmann-Goldberg reaction*

At the outset, we began our investigation with the optimization of the modified Ullmann-Goldberg reaction using δ-valerolactame (1a) and 3-fluoro-4-iodoaniline (2) as a model substrate (Table S1). In the starting conditions, we used conventional reflux conditions in toluene for 24 h to give (3a) in a yield of 29%. To reduce the reaction time, we evaluated the microwave irradiation together with an increase in the reaction temperature (entry 2-4, Table S1). The temperature profile presented an optimal reaction temperature at 140 °C under microwave irradiation, and in these conditions (entry 4. Table S1) the compound (3a) could be obtained in a reaction time of only 2 hours and with a 4% more reaction yield with respect to conventional reflux heating. Later, we studied the effect of different solvents (entry 5-6, Table S1) and ligands (entry 7) with the objective to increase the yield of the reaction. However, these experiments were unsuccessful. Then we decided to reduce the amount of ligand and catalyst to make the reaction truly catalytic. Under these conditions (entry 8, Table S1), surprisingly, there was no significant decrease in reaction yield. In addition, aiming to develop a safe and green methodology we decided to replace the toxic toluene by a green alternative, among the bio-based solvents, 2-MeTHF represents a viable alternative for replacement of different polar aprotic solvents. The use of 2-MeTHF at 120 °C furnished the product 3a in 68% yield.

Table S1. Optimization of the modified Ullmann-Goldberg reaction. [1]

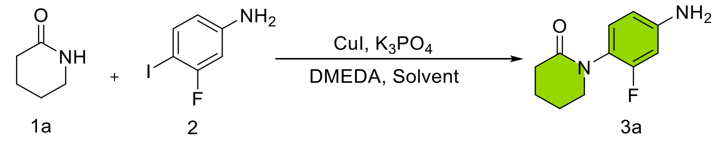

Entry	Solvent	T (°C)	CuI (mol%)	Ligand	ligand (mol%)	Heating	Time (h)	Yield (%)[2]
1	Toluene	110	40	DMEDA	85	Refluxing	24	29
2	Toluene	120	40	DMEDA	85	MW	2	19
3	Toluene	160	40	DMEDA	85	MW	2	20
4	Toluene	140	40	DMEDA	85	MW	2	33
5	1,4-dioxane	140	40	DMEDA	85	MW	2	33
6	THF	140	40	DMEDA	85	MW	2	25
7	Toluene	140	40	CyDMEDA	85	MW	2	20
8	Toluene	140	20	DMEDA	40	MW	2	28
9	**2-MeTHF**	140	20	DMEDA	40	MW	2	48
10	**2-MeTHF**	160	20	DMEDA	40	MW	2	28
11	**2-MeTHF**	120	20	DMEDA	40	MW	2	68

[1] Reaction conditions: 1a (2.5 mmol, 248 mg), 2 (3.0 mmol, 711 mg), K_3PO_4 (5.0 mmol, 1061 mg), CuI (0.5 mmol, 95 mg), ligand (1.0 mmol, 108 µL), in solvent (6 mL), under N_2 atmosphere, at microwave radiation (initial power 850 watts), reaction time 2 h. [2] Yields of isolated products after column chromatography.

3. *Optimization of the CuNPs/C-catalyzed multicomponent CuAAC reaction in* water

Table S2. Optimization of multicomponent 1,3-dipolar azide-alkyne cycloaddition catalyzed by CuNPs/C using ethyl-2-bromoacetate as the azide precursor.[1]

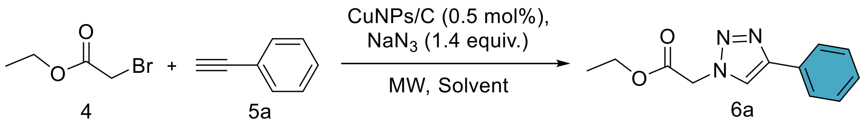

Entry	Solvent	T (°C)	Time (min)	Yield (%)[2]
1	H_2O	70	30	72
2	H_2O	70	60	78
3	H_2O	85	30	75
4	H_2O	100	30	53
5	H_2O	85	15	67
6	H_2O:MeOH	85	30	71

[1] Reaction conditions: 4 (0.5 mmol, 55 µL), 5a (0.5 mmol, 55 µL), NaN_3 (0.7 mmol, 45 mg), CuNPs/C (0.5 mol%, 20 mg), solvent (2 mL), at microwave radiation (initial power 850 watts) for 30 minutes. [2] Yields of isolated products.

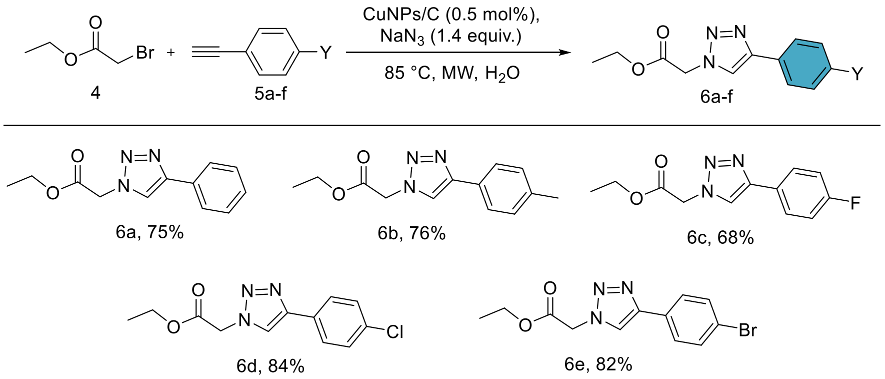

Scheme S1. Multicomponent 1,3-dipolar azide-alkyne cycloaddition catalyzed by CuNPs/C using ethyl-2-bromoacetate as the azide precursors. Reaction conditions: 4 (0.5 mmol, 55 μL), 5a-f (0.5 mmol), NaN_3 (0.7 mmol, 45 mg), CuNPs/C (0.5 mol%, 20 mg), water (2 mL), at microwave irradiation (initial power 850 watts), reaction time 30 minutes. Yields of isolated products.

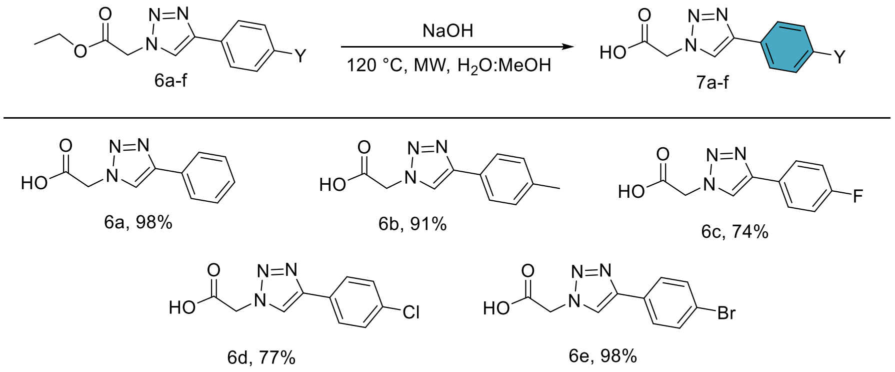

Scheme S2. Ester hydrolysis reaction. Reaction conditions: 6 (0.5 mmol), NaOH solution (1 mL, 2 M), in solvent (2 mL, water: methanol 1:1), at microwave radiation (initial power 850 watts), for 30 minutes. Yields of isolated products.

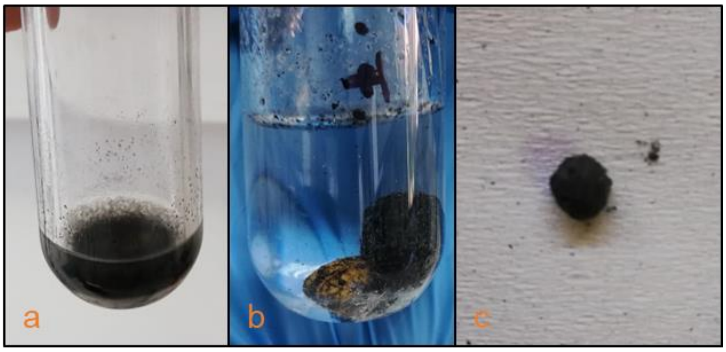

Figure S1. Effect of the solvent system on the catalyst. (**a**) Mixture H_2O:MeOH (1:1), (**b**) H_2O, (**c**) catalyst after a reaction that uses H_2O as a solvent.

4. *Comparison of amide synthesis by conventional heating and MW heating*

Table S3. Comparison of the amide syntheses.[1]

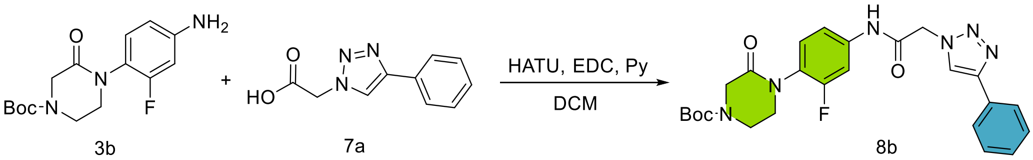

Entry	Heating	T (°C)	Time (h)	Yield (%)[2]
1	r.t.	25	12	60
2	MW	70	0.5	70

[1] Reaction conditions: 3b (0.65 mmol, 201 mg), 7a (0.5 mmol, 102 mg), HATU (0.5 mmol, 190 mg), EDC (0.5 mmol, 96 mg), Py (0.5 mmol, 40 μL), DCM (8 mL), at microwave irradiation (initial power 850 watts) for 30 minutes. [2] Yields of isolated products.

5. *General procedure of Ullman-Goldberg reaction (**3a-d**)*

A mixture of CuI (0.5 mmol, 95.0 mg), 3-fluoro-4-iodoaniline (3.0 mmol, 711 mg), δ-valerolactame (2.5 mmol, 248 mg), K_3PO_4 (5.0 mmol, 1061 mg), *N,N′*-dimethylethylenediamine (DMEDA) (1.0 mmol, 108 μL) and anhydrous 2-MeTHF (6 mL) was placed in the microwave reactor (Synthesis Reactor Monowave 200, Anton Paar) and irradiated with initial power (850 watts) at 140°C for 2 hours. The crude residue was purified by flash column chromatography on hand-packed columns of silica gel 60 (230-400 mesh) (Sigma-Aldrich, Missouri, USA) as the stationary phase and hexane/ethyl acetate as the solvent mixture (mobile phase), to obtain the corresponding isolated arylamines.

1-(4-amino-2-fluorophenyl)piperidin-2-one (**3a**)

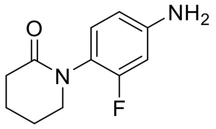

Brown solid; 68% yield; mp: 132-133 °C; IR (KBr) ν / cm^{-1} 3449, 3202, 2955, 1666, 1342, 957; ^{1}H NMR (400 MHz, $CDCl_3$) δ 1.85 – 1.92 (m, 4H), 2.46 – 2.54 (m, 2H), 3.45 – 3.53 (m, 2H), 3.82 (s, 2H), 6.29 – 6.38 (m, 2H), 6.90 (t, *J* = 8.6 Hz, 1H). ^{13}C NMR (101 MHz, $CDCl_3$) δ 21.4, 23.5, 32.6, 51.7 (d, *J* = 1.7 Hz), 102.8 (d, *J* = 23.2 Hz), 110.9 (d, *J* = 2.8 Hz), 120.5 (d, *J* = 13.7 Hz), 129.3 (d, *J* = 3.7 Hz), 148.0 (d, *J* = 10.4 Hz), 158.3 (d, *J* = 246.9 Hz), 170.4; ^{19}F NMR (376 MHz, $CDCl_3$) δ -121.94 ppm; HRMS (FTMS + pESI) *m/z*, calcd. for $C_{11}H_{13}FN_2O$ $[M + H]^+$: 209.1090, found 209.1310.

tert-butyl 4-(4-amino-2-fluorophenyl)-3-oxopiperazine-1-carboxylate (**3b**)

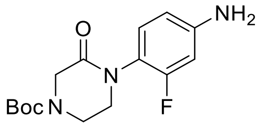

Pale yellow solid; 85% yield; mp: 164-165 °C; IR (KBr) ν / cm^{-1} 3410, 3332, 3240, 2879, 1705, 1519,1280; ^{1}H NMR (400 MHz, $CDCl_3$) δ 1.47 (s, 9H), 3.57 (t, *J* = 5.3 Hz, 2H), 3.73 (t, *J* = 5.3 Hz, 2H), 3.88 (s, 2H), 4.20 (s, 2H), 6.33 – 6.39 (m, 2H), 6.93 (t, *J* = 8.6 Hz, 1H); ^{13}C NMR (101 MHz, $CDCl_3$) δ 28.4, 40.5, 48.2, 50.1 (d, *J* = 1.7 Hz), 80.9 , 102.7 (d, *J* = 23.1 Hz), 110.9 (d, *J* = 2.6 Hz), 118.9 (d, *J* = 13.5 Hz), 129.2 (d, *J* = 3.1 Hz), 148.4 (d, *J* = 10.7 Hz), 153.9, 158.2 (d, *J* = 247.4 Hz), 166.2; ^{19}F NMR (376 MHz, $CDCl_3$) δ -121.47 ppm; HRMS (FTMS + pESI) *m/z*, calcd. for $C_{15}H_{20}FN_3O_3$ $[M + H]^+$: 310.1567 found 310.1560.

4-(4-amino-2-fluorophenyl)morpholin-3-one (**3c**)

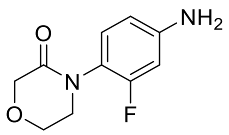

White solid; 90% yield; mp: 172-174 °C; IR (KBr) ν / cm^{-1} 3471, 3356, 2877, 1674, 1234, 1103; ^{1}H NMR (400 MHz, $CDCl_3$) δ 3.65 (dd, *J* = 5.9, 4.2 Hz, 2H), 3.84 (s, 2H), 4.00 (dd, *J* = 5.8, 4.3 Hz, 2H), 4.33 (s, 2H), 6.41 – 6.47 (m, 2H), 7.01 (t, *J* = 8.3 Hz, 1H); ^{13}C NMR (101 MHz, $CDCl_3$) δ 50.2, 64.3, 68.7, 102.9 (d, *J* = 23.2 Hz), 111.1 (d, *J* = 2.8 Hz), 118.8 (d, *J* = 13.4 Hz), 129.5 (d, *J* = 3.1 Hz), 148.2 (d, *J* = 10.7 Hz), 158.5 (d, *J* = 248.0 Hz), 167.2; ^{19}F NMR (376 MHz, $CDCl_3$) δ -121.24 ppm; HRMS (FTMS + pESI) *m/z*, calcd. for $C_{10}H_{11}FN_2O_2$ $[M + H]^+$: 211.0883 found 211.0875.

4-(4-amino-2-fluorophenyl)thiomorpholin-3-one (**3d**)

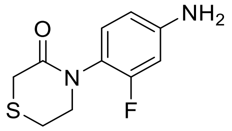

Yellow solid; 61% yield; mp: 150-152 °C; IR (KBr) ν / cm^{-1} 3417, 3340, 3232, 3024, 2931, 1674, 1249; ^{1}H NMR (400 MHz, $CDCl_3$) δ 3.01 (dd, *J* = 6.5, 5.0 Hz, 2H), 3.45 (s, 2H), 3.77 – 3.90 (m, 4H), 6.39 – 6.44 (m, 2H), 7.00 (t, *J* = 8.3 Hz, 1H); ^{13}C NMR (101 MHz, $CDCl_3$) δ 26.7, 30.5, 52.8 (d, *J* = 1.3 Hz), 102.9 (d, *J* = 23.2 Hz), 111.0 (d, *J* = 2.8 Hz), 120.4, 129.6 (d, *J* = 3.1 Hz), 147.9 (d, *J* = 10.6 Hz), 158.3 (d, *J* = 247.4 Hz), 167.1; ^{19}F NMR (376 MHz, $CDCl_3$) δ -121.07 ppm; HRMS (FTMS + pESI) *m/z*, calcd. for $C_{10}H_{11}FN_2OS$ $[M + H]^+$: 227.0654 found 227.0645.

6. *Procedure for the preparation of CuNPs/C*

Anhydrous copper (II) chloride (135 mg, 1 mmol) was added to a suspension of lithium powder (14 mg, 2 mmol) and 4,4'-di-*tert*-butylbiphenyl (DTBB, 27 mg, 0.1 mmol) in THF (2 mL) at room temperature under a nitrogen atmosphere. The reaction mixture, which was initially dark blue, rapidly changed to black (ca. 5–10 min), indicating that the suspension of copper nanoparticles was successfully formed. This suspension was diluted with THF (8 mL) followed by the addition of the activated carbon (800 mg). The resulting mixture was stirred for 3 h at room temperature, filtered, and the solid successively washed with water (20 mL), diethyl ether (20 mL) and then dried under vaccum (15 Torr).

7. *Characterization of CuNPs/C*

The copper nanoparticles on activated carbon (CuNPs/C) were previously reported in the literature. Reproduced from Ref. [1,2] with permission from the Royal Society of Chemistry.

The nanocatalyst (CuNPs/C) was characterized by different means of transmission electron microscopy (TEM), energy-dispersive X-ray spectroscopy (EDX) and X-ray photoelectron spectroscopy (XPS).

Analysis by TEM revealed the presence of spherical nanoparticles dispersed on the active carbon with diameters of ca. 6±2 nm (Figure s2).

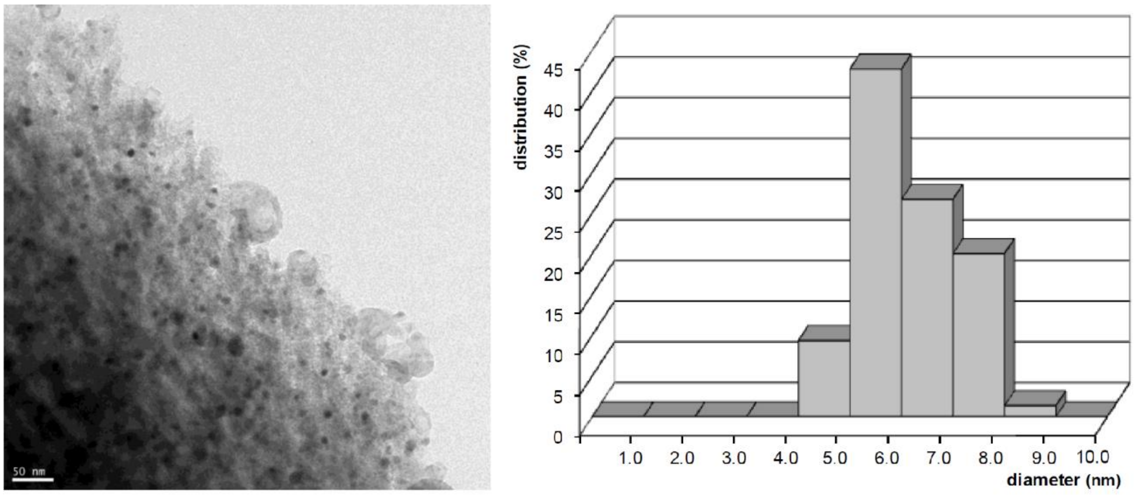

Figure S2. TEM micrograph and size distribution of CuNPs on active carbon

Energy-dispersive Xray (EDX) analysis on various regions confirmed the presence of copper, with energy bands of 8.04, 8.90 keV (K lines) and 0.92 keV (L line).

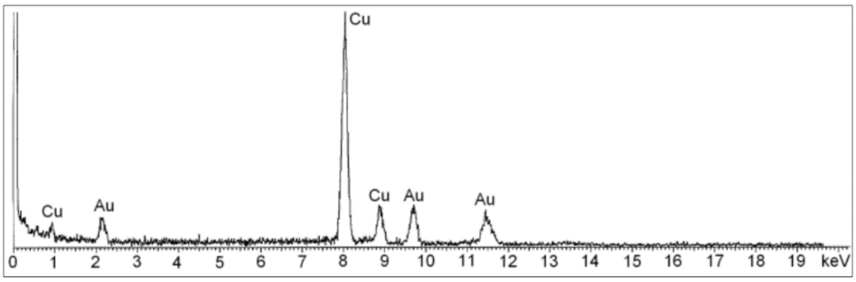

Figure S3. EDX spectrum of CuNPs/C

The copper loading (1.6 wt%) and BET area (1224 m^2 g−1). were determined using inductively coupled plasma optical emission spectroscopy (ICP-OES) and adsorption isotherms, respectively.

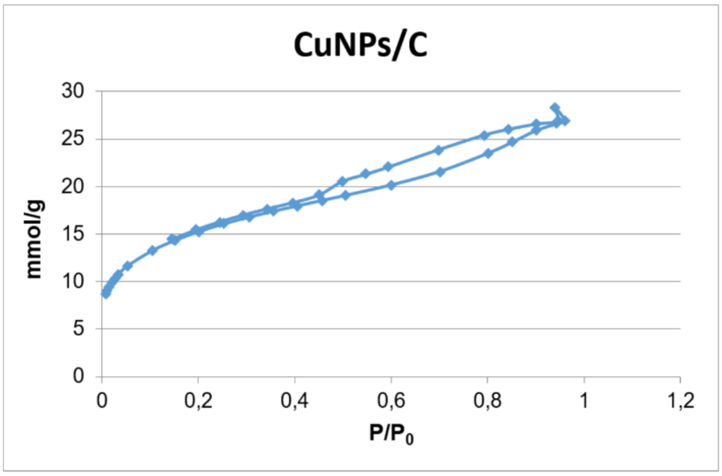

Figure S4. Adsorption isotherm of CuNPs/C

Analysis by XPS revealed that the surface of the CuNPs is oxidized and consists of both Cu(I) and Cu(II) oxides.

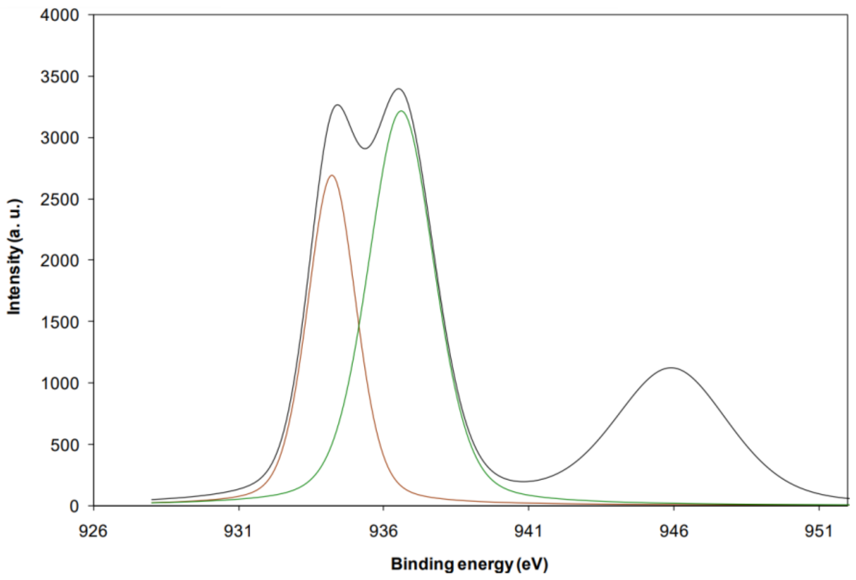

Figure S5. XPS spectrum of the CuNPs/C at the Cu $2p_{3/2}$ level

The selected-area electron-diffraction pattern (SAED) of the CuNPs/C is also in agreement with the presence of Cu_2O and CuO.

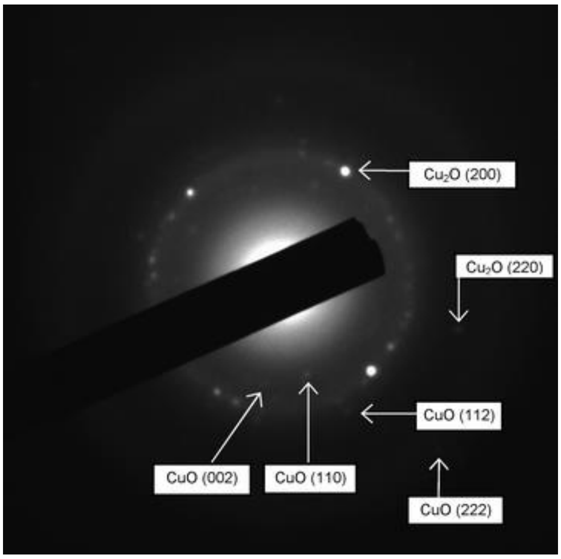

Figure S6. Selected area electron diffraction (SAED) pattern of the CuNPs/C.

The XRD diffractogram did not show any significant peak due to the amorphous character of the sample, to the fact that the crystal domains are <10 nm, and/or low copper loading weight.

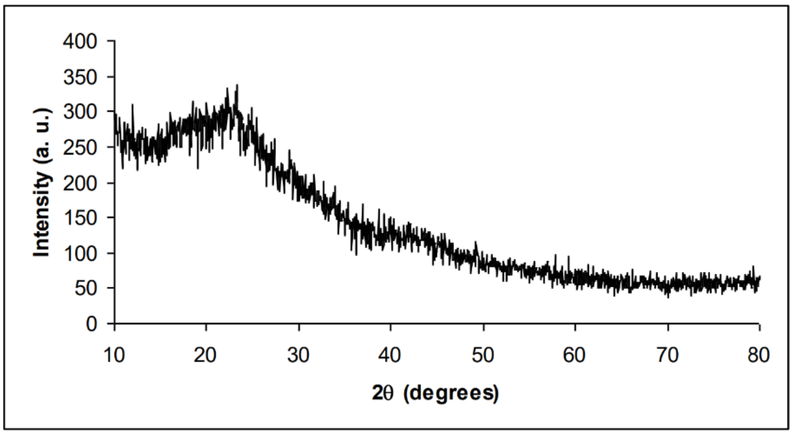

Figure S7. XRD spectrum of CuNPs/C

8. *General procedure for the CuNPs/C-catalyzed multicomponent CuAAC reaction in water (**6a-6e**)*

A mixture of NaN_3 (0.7 mmol, 45.5 mg), ethyl-2-bromoacetate (0.5 mmol, 83.5 mg, 55.7 μL) and the ethynylbenzene (0.5 mmol,) was added to a suspension of CuNPs/C (20 mg, 0.5 mol% Cu) in H_2O (2 mL) and was placed in the microwave reactor (Synthesis Reactor Monowave 200, Anton Paar) and irradiated with initial power (850 watts) at 85 °C for 30 minutes. Water (30 mL) was added to the resulting mixture, followed by extraction with EtOAc (3 × 10 mL). The collected organic phases were dried with anhydrous $MgSO_4$ and the solvent was removed in vacuo to give the corresponding triazol.

Ethyl 2-(4-phenyl-1*H*-1,2,3-triazol-1-yl)acetate (**6a**)[3]

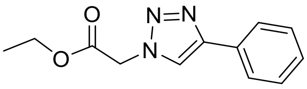

White solid, yield 75%; mp: 94-95 °C; IR (KBr) ν / cm^{-1} = 3132, 2993, 1759, 1504, 1342 , 1234, 1026, 779, 702; ^1H NMR (300 MHz, $CDCl_3$) δ 1.29 (t, *J* = 7.1 Hz, 3H), 4.26 (q, *J* = 7.1 Hz, 2H), 5.18 (s, 2H), 7.29 – 7.36 (m, 1H), 7.37 – 7.45 (m, 2H), 7.80 – 7.86 (m, 2H), 7.91 (s, 1H); ^{13}C NMR (75 MHz, $CDCl_3$) δ 14.1, 51.0, 62.4, 121.0, 125.8, 128.3, 128.8, 130.4, 148.2, 166.3 ppm; HRMS (FTMS + pESI) *m*/*z*, calcd. for $C_{12}H_{13}N_3O_2$ $[M + H]^+$: 232.1086 found 232.1081.

Ethyl 2-(4-(*p*-tolyl)-1*H*-1,2,3-triazol-1-yl)acetate (**6b**)[4]

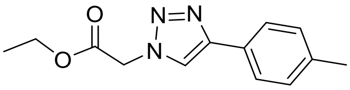

Yellow solid, yield 76%; mp: 228-229 ºC; IR (KBr) ν / cm^{-1} = 3109, 2993, 1743, 1473, 1350, 1296, 771; ^1H NMR (400 MHz, $CDCl_3$) δ 1.29 (t, *J* = 7.2 Hz, 3H), 2.37 (s, 3H), 4.25 (q, *J* = 7.1 Hz, 2H), 5.17 (s, 2H), 7.22 (d, *J* = 7.9 Hz, 2H), 7.72 (d, *J* = 8.2 Hz, 2H), 7.87 (s, 1H); ^{13}C NMR (101 MHz, $CDCl_3$) δ 14.1, 21.3, 51.0, 62.5, 120.8, 125.8, 127.6, 129.6, 138.2, 148.3, 166.4 ppm; HRMS m/z calcd. for $C_{13}H_{15}N_3O_2$ $[M + 1]^+$: 246.1242, found 246.1238.

Ethyl 2-(4-(4-fluorophenyl)-1*H*-1,2,3-triazol-1-yl)acetate (**6c**)

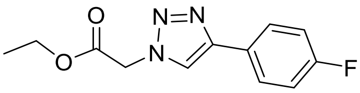

White solid, yield 68%; mp: 139-140 °C; IR (KBr) ν / cm^{-1} = 3109, 2993, 1743, 1612,1496, 1342, 1234, 771; ^1H NMR (400 MHz, $CDCl_3$) δ 1.30 (t, *J* = 7.2 Hz, 3H), 4.27 (q, *J* = 7.1 Hz, 2H), 5.20 (s, 2H), 7.07 – 7.14 (m, 2H), 7.77 – 7.83 (m, 2H), 7.89 (s, 1H).^{13}C NMR (101 MHz, $CDCl_3$) δ 14.1, 51.0, 62.6, 115.9 (d, *J* = 21.9 Hz), 120.9, 126.7 (d, *J* = 3.3 Hz), 127.6 (d, *J* = 8.1 Hz), 147.4, 162.8 (d, *J* = 247.4 Hz), 166.4; ^{19}F NMR (376 MHz, $CDCl_3$) δ -113.45 ppm; HRMS (FTMS + pESI) *m*/*z*, calcd. for $C_{12}H_{12}FN_3O_2$ [M + H]$^+$: 250.0992 found 250.0986.

Ethyl 2-(4-(4-chlorophenyl)-1*H*-1,2,3-triazol-1-yl)acetate (**6d**)

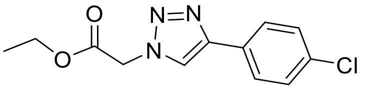

White solid, yield 84%; mp: 1410-141 °C; IR (KBr) ν / cm^{-1} = 3124, 2993, 1743, 1604, 1481, 1342 , 1242, 694; ^1H NMR (300 MHz, $CDCl_3$) δ 1.29 (t, *J* = 7.2 Hz, 3H), 4.26 (q, *J* = 7.2 Hz, 2H), 5.18 (s, 2H), 7.37 (d, *J* = 8.4 Hz, 2H), 7.75 (d, *J* = 8.5 Hz, 2H), 7.90 (s, 1H); ^{13}C NMR (75 MHz, $CDCl_3$) δ 14.1, 51.0, 62.6, 121.2, 127.1, 129.0, 129.1, 134.1, 147.2, 166.3 ppm; HRMS (FTMS + pESI) *m*/*z*, calcd. for $C_{12}H_{12}ClN_3O_2$ [M + H]$^+$: 266.0696 found 266.0691.

Ethyl 2-(4-(4-bromophenyl)-1*H*-1,2,3-triazol-1-yl)acetate (**6e**)[5]

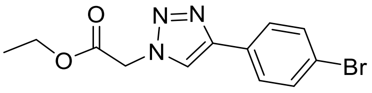

white solid; yield 82%; mp: 129-131 °C; IR (KBr) ν / cm^{-1} = 3124, 2993, 1735, 1604, 1550, 1242 , 1018, 694, 579; ^1H NMR (300 MHz, $CDCl_3$) δ 1.30 (t, *J* = 7.2 Hz, 3H), 4.28 (q, *J* = 7.1 Hz, 2H), 5.19 (s, 2H), 7.54 (d, *J* = 8.5 Hz, 2H), 7.70 (d, *J* = 8.5 Hz, 2H), 7.91 (s, 1H); ^{13}C NMR (75 MHz, $CDCl_3$) δ 14.2, 51.1, 62.6, 121.2, 122.3, 127.4, 129.5, 132.1, 147.3, 166.3 ppm; HRMS calcd for $C_{12}H_{12}BrN_3O_2$(M^+): 310.0191, found 310.0186.

9. *General procedure for the hydrolysis of ethyl ester derivates (**7a-e**)*

Ethyl 2-(4-phenyl-1H-1,2,3-triazol-1-yl)acetate (0.5 mmol, 115.6 mg) was dissolved in methanol and an aqueous solution of NaOH (1 mL, 2M) was added. The reaction tube was placed in the microwave reactor (Synthesis Reactor Monowave 200, Anton Paar) and irradiated with initial power (850 watts) at 120 °C for 30 minutes. Water (30 mL) was added to the resulting mixture, then acidified (pH= 3) using HCl (1 M), and extracted three times with ethyl acetate (3 × 10 mL). The collected organic phases were dried with anhydrous $MgSO_4$, and the solvent was removed in vacuo to give the corresponding acid.

2-(4-phenyl-1*H*-1,2,3-triazol-1-yl)acetic acid (**7a**)

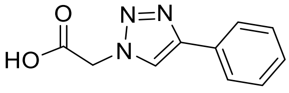

White solid, yield 98%; mp: 198-200 °C; IR (KBr) ν / cm^{-1} = 3147, 3055, 2970, 2700, 2529, 2507, 1728, 1612, 1558, 1342, 1226, 1095, 763; ^1H NMR (400 MHz, DMSO-d_6) δ 5.34 (s, 2H), 7.31 – 7.37 (m, 1H), 7.46 (t, *J* = 7.6 Hz, 2H), 7.83 – 7.88 (m, 2H), 8.55 (s, 1H), 13.46 (s, 1H); ^{13}C NMR (101 MHz, DMSO-d_6) δ 50.7, 122.7, 125.1, 127.9, 128.9, 130.6, 146.3, 168.6 ppm; HRMS (FTMS + pESI) *m*/*z*, calcd. for $C_{10}H_9N_3O_2$ [M + H]$^+$: 204.0773 found 204.0765.

2-(4-(*p*-tolyl)-1*H*-1,2,3-triazol-1-yl)acetic acid (**7b**)

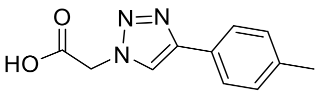

White solid, yield 91%; mp: 208 °C; IR (KBr) ν / cm^{-1} 3147, 3039, 2916, 2700, 2529, 2507, 1728, 1620, 1465, 1342, 1226 , 1087, 810; ^{1}H NMR (400 MHz, DMSO-*d*$_6$) δ 2.33 (s, 3H), 5.32 (s, 2H), 7.26 (d, *J* = 7.9 Hz, 2H), 7.74 (d, *J* = 8.1 Hz, 2H), 8.48 (s, 1H), 13.44 (s, 1H); ^{13}C NMR (101 MHz, DMSO-*d*$_6$) δ 20.8, 50.6, 122.3, 125.1, 127.9, 129.5, 137.2, 146.4, 168.6 ppm; HRMS (FTMS + pESI) *m/z*, calcd. for $C_{11}H_{11}N_3O_2$ [M + H]$^+$: 218.0929 found 218.0922.

2-(4-(4-fluorophenyl)-1*H*-1,2,3-triazol-1-yl)acetic acid (**7c**)

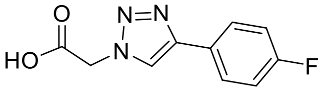

White solid, yield 74%; mp: 228 °C; IR (KBr) ν / cm^{-1} 3155, 3055, 2916, 2708, 2592, 2507, 1728, 1612, 1566, 1504, 1342, 1242, 1087, 894; ^{1}H NMR (400 MHz, DMSO-*d*$_6$) δ 5.34 (s, 2H), 7.26 – 7.34 (m, 2H), 7.87 – 7.94 (m, 2H), 8.55 (s, 1H), 13.47 (s, 1H); ^{13}C NMR (101 MHz, DMSO-*d*$_6$) δ 50.7, 115.9 (d, *J* = 21.7 Hz), 122.6, 127.2 (d, *J* = 8.2 Hz), 145.4, 161.8 (d, *J* = 244.6 Hz), 168.6; ^{19}F NMR (376 MHz, DMSO-*d*$_6$) δ -114.09 ppm; HRMS (FTMS + pESI) *m/z*, calcd. for $C_{10}H_8FN_3O_2$ [M + H]$^+$: 222.0679 found 222.0670.

2-(4-(4-chlorophenyl)-1*H*-1,2,3-triazol-1-yl)acetic acid (**7d**)

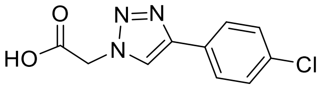

White solid, yield 77%; mp: 250-251 °C; IR (KBr) ν / cm^{-1} = 3150, 2916, 2700, 2592, 2507, 1728, 1604, 1550, 1489, 1342, 1234, 1087, 817; ^{1}H NMR (300 MHz, DMSO-*d*$_6$) δ 5.34 (s, 2H), 7.52 (d, *J* = 8.6 Hz, 2H), 7.88 (d, *J* = 8.6 Hz, 2H), 8.59 (s, 1H), 13.47 (s, 1H); ^{13}C NMR (75 MHz, DMSO-*d*$_6$) δ 50.7, 123.1, 126.8, 129.0, 129.5, 132.3, 145.2, 168.5 ppm; HRMS (FTMS + pESI) *m/z*, calcd. for $C_{10}H_8ClN_3O_2$ [M + H]$^+$: 238.0383 found 283.0376.

2-(4-(4-bromophenyl)-1*H*-1,2,3-triazol-1-yl)acetic acid (**7e**)

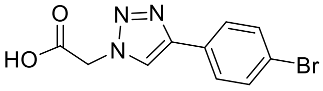

White solid, yield 98%; mp: 245-246 °C; IR (KBr) ν / cm^{-1} = 3150, 2916, 2700, 2592, 2507, 1728, 1604, 1550, 1489, 1342, 1234, 1087, 817; ^{1}H NMR (300 MHz, DMSO-*d*$_6$) δ 5.34 (s, 2H), 7.65 (d, *J* = 8.5 Hz, 2H), 7.82 (d, *J* = 8.5 Hz, 2H), 8.60 (s, 1H), 13.32 (s, 1H); ^{13}C NMR (75 MHz, DMSO-*d*$_6$) δ 51.2, 121.3, 123.6, 127.6, 130.4, 132.4, 145.7, 169.0 ppm; HRMS (FTMS + pESI) *m/z*, calcd. for $C_{10}H_8BrN_3O_2$ [M + H]$^+$: 281.9878 found 281.9698.

10. *General procedure of peptide coupling reaction (**8a-t**)*

A mixture of HATU (0.5 mmol, 190.1 mg), EDC (0.5 mmol, 95.9 mg) and 2-(4-phenyl-1H-1,2,3-triazol-1-yl)acetic acid (7a) (0.5 mmol, 101.6 mg) were weighed and transferred into a dried reaction tube. The tube was evacuated and backfilled with dry N_2. The reaction was stirred at 0 °C for 1 hour after adding dry dichloromethane (DCM) (8 mL). Next, the 1-(4-amino-2-fluorophenyl)piperidin-2-one (3a) (0.65 mmol, 135.4 mg) and pyridine (Py) (0.5 mmol, 40 μL) were added to the reaction tube. The reaction was placed in the microwave reactor (Synthesis Reactor Monowave 200, Anton Paar) and irradiated with initial power (850 watts) at 70 °C for 30 minutes. The remaining mixture was washed

once with citric acid (10% w/w), saturated $NaHCO_3$, brine, and dried over anhydrous $MgSO_4$. The solvent was removed under vacuum, and the remaining product was purified by flash column chromatography on silica gel 60 (230-400 mesh) (Sigma-Aldrich, USA) using n-hexane/ethyl acetate as the solvent mixture.

N-(3-fluoro-4-(2-oxopiperidin-1-yl)phenyl)-2-(4-phenyl-1*H*-1,2,3-triazol-1-yl)acetamide (**8a**)

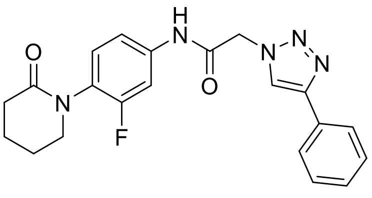

Beige solid; yield: 50%; mp: 250-251 °C; IR (KBr) ν / cm^{-1} 3435, 3273, 3208, 2959, 2857, 1713, 1635, 1550, 1229; ^1H NMR (400 MHz, DMSO-d_6) δ 1.79 – 1.89 (m, 4H), 2.38 (t, *J* = 6.2 Hz, 2H), 3.50 (t, *J* = 5.6 Hz, 2H), 5.43 (s, 2H), 7.29 – 7.38 (m, 3H), 7.46 (dd, *J* = 8.3, 7.0 Hz, 2H), 7.62 (dd, *J* = 12.5, 2.1 Hz, 1H), 7.85 – 7.91 (m, 2H), 8.61 (s, 1H), 10.91 (s, 1H); ^{13}C NMR (101 MHz, DMSO-d_6) δ 20.9, 22.9, 32.2, 50.8, 52.3, 106.9 (d, *J* = 25.3 Hz), 115.3 (d, *J* = 3.3 Hz), 123.0, 125.1, 126.2 (d, *J* = 13.3 Hz), 127.8, 128.9, 129.4 (d, *J* = 2.9 Hz), 130.7, 138.5 (d, *J* = 10.7 Hz), 146.2, 156.9 (d, *J* = 246.5 Hz), 164.6, 168.8; ^{19}F NMR (376 MHz, DMSO-d_6) δ -119.09 ppm; HRMS (FTMS + pESI) *m/z*, calcd. for $C_{21}H_{20}FN_5O_2$ $[M-H]^-$: 392.1528, found 392.1537.
tert-butyl4-(2-fluoro-4-(2-(4-phenyl-1*H*-1,2,3-triazol-1-yl)acetamido)phenyl)-3-oxopiperazine-1 carboxylate (**8b**)

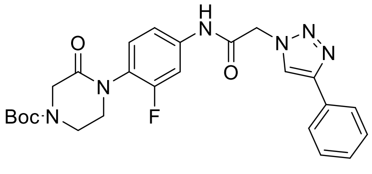

White solid; yield: 70%; mp: 195-196 °C; IR (KBr) ν / cm-1 3468, 3282, 3134, 2978, 2858, 1701, 1647, 1618, 1577, 1153; ^1H NMR (400 MHz, DMSO-d_6) δ 1.44 (s, 9H), 3.63 (t, *J* = 5.8 Hz, 2H), 3.68 (t, *J* = 5.5 Hz, 2H), 4.09 (s, 2H), 5.42 (s, 2H), 7.31 – 7.50 (m, 5H), 7.64 (dd, *J* = 12.5, 2.2 Hz, 1H), 7.84 – 7.91 (m, 2H), 8.60 (s, 1H), 10.80 (s, 1H); ^{13}C NMR (101 MHz, DMSO-d_6) δ 28.0, 40.0, 49.1 (2C), 52.3, 79.8, 106.9 (d, *J* = 25.2 Hz), 115.4 (d, *J* = 3.2 Hz), 123.0, 124.5 (d, *J* = 13.4 Hz), 125.1, 127.9, 128.9, 129.3 (d, *J* = 2.3 Hz), 130.7, 138.9 (d, *J* = 10.7 Hz), 146.2, 153.2, 156.7 (d, *J* = 247.2 Hz), 164.7, 165.0; ^{19}F NMR (376 MHz, DMSO-d_6) δ -118.80 ppm; HRMS (FTMS + pESI) *m/z*, calcd. for $C_{25}H_{27}FN_6O_4$ $[M + H]^+$: 495.2151, found 495.2130.

N-(3-fluoro-4-(3-oxomorpholino)phenyl)-2-(4-phenyl-1*H*-1,2,3-triazol-1-yl)acetamide (**8c**)

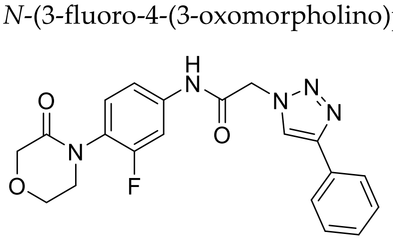

White solid; yield: 43%; mp: 254-255 °C; IR (KBr) ν / cm^{-1} 3505, 3275, 3123, 2941, 2829, 1715, 1651, 1548, 1130; ^1H NMR (400 MHz, DMSO-d_6) δ 3.64 (t, *J* = 4.9 Hz, 2H), 3.97 (t, *J* = 5.1 Hz, 2H), 4.22 (s, 2H), 5.42 (s, 2H), 7.31 – 7.49 (m, 5H), 7.65 (dd, *J* = 12.5, 2.2 Hz, 1H), 7.85 – 7.90 (m, 2H), 8.60 (s, 1H), 10.84 (s, 1H). ^{13}C NMR (101 MHz, DMSO-d_6) δ 49.4, 52.4, 63.5, 67.7, 107.0 (d, *J* = 25.2 Hz), 115.5 (d, *J* = 3.2 Hz), 123.1, 124.0 (d, *J* = 13.4 Hz), 125.2, 127.9, 128.9, 129.3 (d, *J* = 2.6 Hz), 130.7, 139.1 (d, *J* = 10.6 Hz), 146.3, 156.9 (d, *J* = 247.2 Hz), 164.7, 166.0; ^{19}F NMR (376 MHz, DMSO-d_6) δ -118.75 ppm; HRMS (FTMS + pESI) *m/z*, calcd. for $C_{20}H_{18}FN_5O_3$ $[M-H]^-$: 394.1321, found 394.1327.

N-(3-fluoro-4-(3-oxothiomorpholino)phenyl)-2-(4-phenyl-1*H*-1,2,3-triazol-1-yl)acetamide (**8d**)

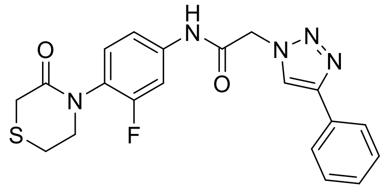

Yellow paled solid; yield: 82%; mp: 276-277 °C; IR (KBr) ν / cm-1 3491, 3291, 3140, 2974, 2859, 1712, 1666, 1612, 1550, 1110; ^{1}H NMR (400 MHz, DMSO-*d*6) δ 3.02 (t, *J* = 5.7 Hz, 2H), 3.43 (s, 2H), 3.87 (t, *J* = 5.9 Hz, 2H), 5.42 (s, 2H), 7.30 – 7.39 (m, 3H), 7.46 (dd, *J* = 8.4, 6.9 Hz, 2H), 7.60 – 7.66 (m, 1H), 7.84 – 7.91 (m, 2H), 8.60 (s, 1H), 10.79 (s, 1H); ^{13}C NMR (101 MHz, DMSO-*d*6) δ 26.0, 29.3, 51.4, 52.3, 106.9 (d, *J* = 25.3 Hz), 115.3 (d, *J* = 3.1 Hz), 123.1, 125.1, 125.5 (d, *J* = 13.3 Hz), 127.9, 128.9, 129.4 (d, *J* = 2.8 Hz), 130.7, 138.6 (d, *J* = 10.6 Hz), 146.2, 156.7 (d, *J* = 246.6 Hz), 164.6, 166.7; ^{19}F NMR (376 MHz, DMSO-*d*6) δ -118.65 ppm; HRMS (FTMS + pESI) *m/z*, calcd. for $C_{20}H_{18}FN_5O_2S$ [M-H]$^-$: 410.1092, found 410.1097.

N-(3-fluoro-4-(2-oxopiperidin-1-yl)phenyl)-2-(4-(4-fluorophenyl)-1*H*-1,2,3-triazol-1-yl)acetamide (**8e**)

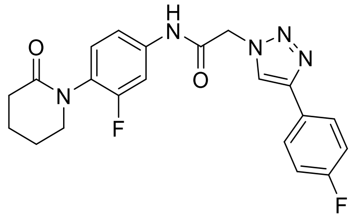

Beige solid; yield:19%; mp: >308 (Decomposition) °C; IR (KBr) ν / cm-1 3421, 3273, 3136, 2949, 2870, 1712, 1635, 1614, 1552, 1157, 1170 ;^{1}H NMR (400 MHz, DMSO-*d*6) δ 1.78 – 1.91 (m, 4H), 2.38 (t, *J* = 6.2 Hz, 2H), 3.51 (t, *J* = 5.6 Hz, 2H), 5.42 (s, 2H), 7.25 – 7.38 (m, 4H), 7.56 – 7.64 (m, 1H), 7.86 – 7.97 (m, 2H), 8.60 (s, 1H), 10.79 (s, 1H); ^{13}C NMR (101 MHz, DMSO-*d*6) δ 20.9, 22.9, 32.2, 50.8, 52.3, 106.9 (d, *J* = 25.4 Hz), 115.3 (d, *J* = 3.0 Hz), 115.8 (d, *J* = 21.6 Hz), 122.9, 126.2 (d, *J* = 13.5 Hz), 127.2 (d, *J* = 8.1 Hz), 127.2 (d, *J* = 3.1 Hz), 129.4 (d, *J* = 3.1 Hz), 138.4 (d, *J* = 10.6 Hz), 145.4, 156.9 (d, *J* = 246.5 Hz), 161.8 (d, *J* = 244.5 Hz), 164.6, 168.8; ^{19}F NMR (376 MHz, DMSO-*d*6) δ -114.12 ppm, -119.05; HRMS (FTMS + pESI) *m/z*, calcd. for $C_{21}H_{19}F_2N_5O_2$ [M-H]$^-$: 410.1434, found 410.1442.

tert-butyl 4-(2-fluoro-4-(2-(4-(4-fluorophenyl)-1*H*-1,2,3-triazol-1-yl)acetamido)phenyl)-3-oxopiperazine-1-carboxylate (**8f**)

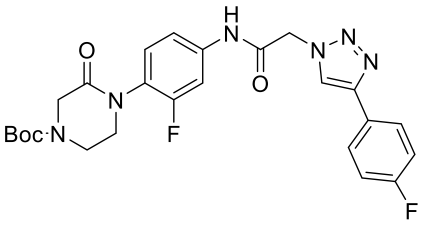

White solid; yield: 62%; mp: 252-253 °C; IR (KBr) ν / cm-1 3288, 3142, 2987, 2866, 1710, 1685, 1641, 1552, 1155, 1124; ^{1}H NMR (400 MHz, DMSO-*d*6) δ 1.44 (s, 9H), 3.62 (t, *J* = 5.2 Hz, 2H), 3.69 (t, *J* = 5.3 Hz, 2H), 4.09 (s, 2H), 5.43 (s, 2H), 7.26 – 7.44 (m, 4H), 7.64 (dd, *J* = 12.4, 2.2 Hz, 1H), 7.88 – 7.97 (m, 2H), 8.60 (s, 1H), 10.80 (s, 1H). ^{13}C NMR (101 MHz, DMSO-*d*6) δ 28.0, 41.3, 47.7, 49.1, 52.4, 79.8, 107.0 (d, *J* = 25.0 Hz), 115.4 (d, *J* = 3.1 Hz), 115.8 (d, *J* = 21.6 Hz), 122.9, 124.5 (d, *J* = 13.3 Hz), 127.2 (d, *J* = 8.2 Hz), 127.3 (d, *J* = 3.2 Hz), 129.3 (d, *J* = 2.6 Hz), 138.9 (d, *J* = 10.6 Hz), 145.4, 153.2, 156.7 (d, *J* = 246.9

Hz), 161.8 (d, J = 244.5 Hz), 164.7, 165.0; ^{19}F NMR (376 MHz, DMSO-d_6) δ -118.8, -114.1 ppm; HRMS (FTMS + pESI) m/z, calcd. for $C_{25}H_{26}F_2N_6O_4$ [M-H]$^-$: 511.1911, found 511.1924.

N-(3-fluoro-4-(3-oxomorpholino)phenyl)-2-(4-(4-fluorophenyl)-1*H*-1,2,3-triazol-1-yl)acetamide (**8g**)

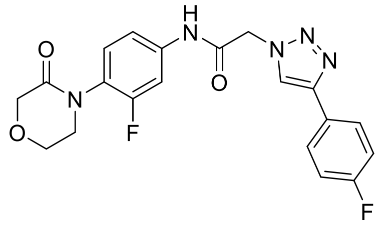

White solid; yield: 52%; mp: 273-274 °C; IR (KBr) ν / cm^{-1} 3285, 3142, 2981, 2874, 1716, 1652, 1552, 1132, 1115; ^{1}H NMR (400 MHz, DMSO-d_6) δ 3.64 (t, J = 5.1 Hz, 2H), 3.97 (t, J = 5.0 Hz, 2H), 4.22 (s, 2H), 5.43 (s, 2H), 7.22 – 7.48 (m, 3H), 7.65 (dd, J = 12.4, 2.2 Hz, 1H), 7.89 – 7.96 (m, 4H), 8.60 (s, 1H), 10.82 (s, 1H). ^{13}C NMR (101 MHz, DMSO-d_6) δ 49.4, 52.4, 63.5, 67.7, 107.0 (d, J = 25.1 Hz), 115.4 (d, J = 3.2 Hz), 115.9 (d, J = 21.7 Hz), 123.0, 124.1 (d, J = 13.5 Hz), 127.2 (d, J = 8.3 Hz), 127.3, 129.4 (d, J = 2.6 Hz), 139.0 (d, J = 10.6 Hz), 145.4, 156.9 (d, J = 247.2 Hz), 161.8 (d, J = 244.5 Hz), 164.7, 166.0; ^{19}F NMR (376 MHz, DMSO-d_6) δ -118.7, -114.1 ppm; HRMS (FTMS + pESI) m/z, calcd. for $C_{20}H_{17}F_2N_5O_3$ [M-H]$^-$: 412.1226, found 412.1235.

N-(3-fluoro-4-(3-oxothiomorpholino)phenyl)-2-(4-(4-fluorophenyl)-1*H*-1,2,3-triazol-1-yl)acetamide (**8h**)

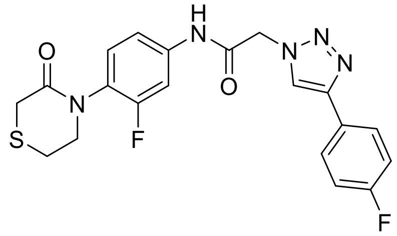

Yellow pale solid; Yield: 42%; mp: 266-267 °C; IR (KBr) ν / cm^{-1} 3288, 3138, 2945, 2856, 1716, 1652, 1550, 1159, 1141; ^{1}H NMR (400 MHz, DMSO-d_6) δ 3.02 (t, J = 5.7 Hz, 2H), 3.43 (s, 2H), 3.87 (t, J = 5.9 Hz, 2H), 5.43 (s, 2H), 7.24 – 7.40 (m, 4H), 7.59 – 7.68 (m, 1H), 7.87 – 7.97 (m, 2H), 8.60 (s, 1H), 10.80 (s, 1H). ^{13}C NMR (101 MHz, DMSO-d_6) δ 26.0, 29.4, 51.4, 52.4, 106.9 (d, J = 25.2 Hz), 115.3 (d, J = 3.3 Hz), 115.9 (d, J = 21.7 Hz), 123.0, 125.5 (d, J = 13.4 Hz), 127.2 (d, J = 8.4 Hz), 127.3, 129.4 (d, J = 2.6 Hz), 138.6 (d, J = 10.7 Hz), 145.4, 156.7 (d, J = 246.7 Hz), 161.8 (d, J = 244.5 Hz), 164.6, 166.8; ^{19}F NMR (376 MHz, DMSO-d_6) δ -118.6, -114.1 ppm; HRMS (FTMS + pESI) m/z, calcd. for $C_{20}H_{17}F_2N_5O_2S$ [M-H]$^-$: 428.0998, found 428.1007.

2-(4-(4-chlorophenyl)-1*H*-1,2,3-triazol-1-yl)-*N*-(3-fluoro-4-(2-oxopiperidin-1-yl)phenyl)acetamide (**8i**)

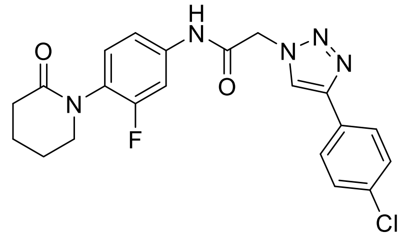

White solid; yield: 19 %; mp: 302-303 °C; IR (KBr) ν / cm^{-1} 3273, 3122, 2947, 2862, 1710, 1635, 1614, 1552, 1171, 823; ^{1}H NMR (400 MHz, DMSO-d_6) δ 1.78 – 1.91 (m, 4H), 2.38 (t, J = 6.1 Hz, 2H), 3.50 (t, J = 5.5 Hz, 2H), 5.42 (s, 2H), 7.32 (d, J = 4.1 Hz, 2H), 7.52 (d, J = 8.2 Hz, 2H), 7.56 – 7.64 (m, 1H), 7.91 (d, J = 8.1 Hz, 2H), 8.65 (s, 1H), 10.76 (s, 1H). ^{13}C NMR (101 MHz, DMSO-d_6) δ 20.9, 22.9, 32.2, 50.8, 52.4, 106.9 (d, J = 25.2 Hz), 115.3 (d, J = 3.0 Hz), 123.4, 126.2 (d, J = 13.4 Hz), 126.8,

129.0, 129.4 (d, *J* = 3.1 Hz), 129.6, 132.3, 138.4 (d, *J* = 10.6 Hz), 145.2, 156.9 (d, *J* = 246.5 Hz), 164.5, 168.8; ^{19}F NMR (376 MHz, DMSO-*d*$_6$) δ -119.0 ppm; HRMS (m/z): HRMS (FTMS + pESI) *m*/*z*, calcd. for $C_{21}H_{19}ClFN_5O_2$ [M-H]$^-$: 426.1138, found 426.1152.

tert-butyl 4-(4-(2-(4-(4-chlorophenyl)-1*H*-1,2,3-triazol-1-yl)acetamido)-2-fluorophenyl)-3-oxopiperazine-1-carboxylate (**8j**)

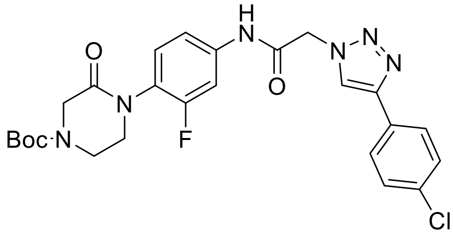

White solid; yield: 69%; mp: 213-214 °C; IR (KBr) ν / cm^{-1} 3286, 3140, 2985, 2864, 1716, 1708, 1685, 1550, 1153, 812; ^{1}H NMR (400 MHz, DMSO-*d*$_6$) δ 1.44 (s, 9H), 3.62 (t, *J* = 5.3 Hz, 2H), 3.69 (t, *J* = 5.4 Hz, 2H), 4.09 (s, 2H), 5.44 (s, 2H), 7.32 – 7.45 (m, 2H), 7.48 – 7.56 (m, 2H), 7.65 (dd, *J* = 12.5, 2.1 Hz, 1H), 7.87 – 7.94 (m, 2H), 8.65 (s, 1H), 10.81 (s, 1H). ^{13}C NMR (101 MHz, DMSO-*d*$_6$) δ 28.0, 41.3, 47.7, 49.1, 52.4 , 79.8, 107.0 (d, *J* = 25.0 Hz),115.4, 123.4, 124.5 (d, *J* = 13.3 Hz), 126.8, 129.0, 129.3 (d, *J* = 2.8 Hz), 129.6, 132.3, 138.9 (d, *J* = 10.6 Hz), 145.2, 153.2, 156.7 (d, *J* = 247.1 Hz), 164.6, 165.0; ^{19}F NMR (376 MHz, , DMSO-*d*$_6$) δ -118.8 ppm; HRMS (FTMS + pESI) *m*/*z*, calcd. for $C_{25}H_{26}ClFN_6O_4$ [M-H]$^-$: 527.1615, found 527.1630.

2-(4-(4-chlorophenyl)-1*H*-1,2,3-triazol-1-yl)-*N*-(3-fluoro-4-(3-oxomorpholino)phenyl)acetamide (**8k**)

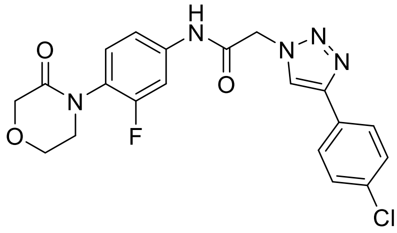

White solid; yield: 27%; mp: 290-291 °C ; IR (KBr) ν / cm^{-1} 3273, 3120, 2986, 2864, 1710, 1647, 1550, 1132, 829; ^{1}H NMR (400 MHz, , DMSO-*d*$_6$) δ 3.64 (t, *J* = 5.1 Hz, 2H), 3.97 (t, *J* = 5.3 Hz, 2H), 4.22 (s, 2H), 5.44 (s, 2H), 7.33 – 7.46 (m, 2H), 7.48 – 7.55 (m, 2H), 7.65 (dd, *J* = 12.4, 2.2 Hz, 1H), 7.87 – 7.95 (m, 2H), 8.65 (s, 1H), 10.81 (s, 1H). ^{13}C NMR (101 MHz, DMSO-*d*$_6$) δ 49.9, 52.9, 63.9, 68.2, 107.5 (d, *J* = 25.2 Hz), 115.9 (d, *J* = 3.2 Hz), 123.9, 124.6 (d, *J* = 13.4 Hz), 127.3, 129.5, 129.8 (d, *J* = 2.8 Hz), 130.0, 132.8, 139.4 (d, *J* = 10.7 Hz), 145.6, 157.4 (d, *J* = 247.3 Hz), 165.1, 166.5; ^{19}F NMR (376 MHz, DMSO-*d*$_6$) δ -118.7 ppm; HRMS (FTMS + pESI) *m*/*z*, calcd. for $C_{20}H_{17}ClFN_5O_3$ [M-H]$^-$: 428.0931, found 428.0942.

2-(4-(4-chlorophenyl)-1*H*-1,2,3-triazol-1-yl)-*N*-(3-fluoro-4-(3-oxothiomorpholino)phenyl)acetamide (**8l**)

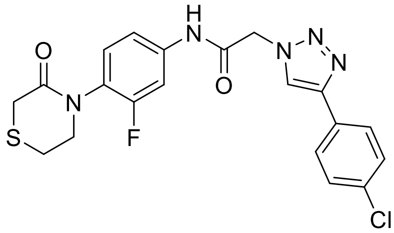

White solid; yield: 51 %; mp: 295-296 °C; IR (KBr) ν / cm^{-1} 3280, 3120, 2976, 2856, 1699, 1654, 1550, 1112, 821; ^{1}H NMR (400 MHz, DMSO-d_6) δ 3.02 (t, *J* = 5.8 Hz, 2H), 3.43 (s, 2H), 3.86 (t, *J* = 5.9 Hz, 2H), 5.47 (s, 2H), 7.34 (t, *J* = 8.6 Hz, 1H), 7.41 (dd, *J* = 8.7, 2.2 Hz, 1H), 7.47 – 7.56 (m, 2H), 7.67 (dd, *J* = 12.5, 2.2 Hz, 1H), 7.86 – 7.95 (m, 2H), 8.67 (s, 1H), 11.19 (s, 1H). ^{13}C NMR (101 MHz, DMSO-d_6) δ 26.0, 29.4, 51.4, 52.4, 106.9 (d, *J* = 25.2 Hz), 115.3 (d, *J* = 3.0 Hz), 123.4, 125.4 (d, *J* = 13.4 Hz), 126.9, 129.0, 129.3 (d, *J* = 2.9 Hz), 129.6, 132.3, 138.8 (d, *J* = 10.7 Hz), 145.1, 156.7 (d, *J* = 246.5 Hz), 164.6, 166.7; ^{19}F NMR (376 MHz, DMSO-d_6) δ -118.7 ppm; HRMS (FTMS + pESI) *m*/*z*, calcd. for $C_{20}H_{17}ClFN_5O_2S$ [M-H]$^-$: 444.0703, found 444.0716.

2-(4-(4-bromophenyl)-1*H*-1,2,3-triazol-1-yl)-*N*-(3-fluoro-4-(2-oxopiperidin-1-yl)phenyl)acetamide (**8m**)

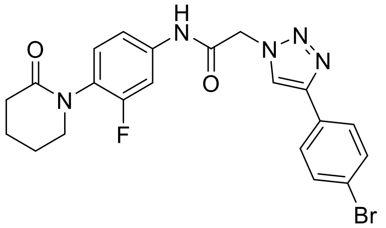

White solid; Yield: 27%; mp: 300-301 °C; IR (KBr) ν / cm-1 3275, 3118, 2951, 2858, 1712, 1635, 1551, 1118, 567; ^{1}H NMR (400 MHz, DMSO-d_6) δ 1.77 – 1.90 (m, 4H), 2.38 (t, *J* = 6.2 Hz, 2H), 3.50 (t, *J* = 5.6 Hz, 2H), 5.43 (s, 2H), 7.27 – 7.38 (m, 2H), 7.57 – 7.70 (m, 3H), 7.84 (d, *J* = 8.5 Hz, 2H), 8.66 (s, 1H), 10.84 (s, 1H). ^{13}C NMR (101 MHz, DMSO-d_6) δ 21.4, 23.4, 32.7, 51.3, 52.9, 107.4 (d, *J* = 25.4 Hz), 115.8 (d, *J* = 3.2 Hz), 121.3, 123.9, 126.7 (d, *J* = 13.3 Hz), 127.6, 129.9 (d, *J* = 2.9 Hz), 130.4, 132.4, 138.9 (d, *J* = 10.6 Hz), 145.7, 157.4 (d, *J* = 246.3 Hz), 165.0, 169.2; ^{19}F NMR (376 MHz, DMSO-d_6) δ -119.0 ppm; HRMS (FTMS + pESI) *m*/*z*, calcd. for $C_{21}H_{19}BrFN_5O_2$ [M-H]$^-$: 470.0633, found 470.0684.

tert-butyl 4-(4-(2-(4-(4-bromophenyl)-1*H*-1,2,3-triazol-1-yl)acetamido)-2-fluorophenyl)-3-oxopiperazine-1-carboxylate (**8n**)

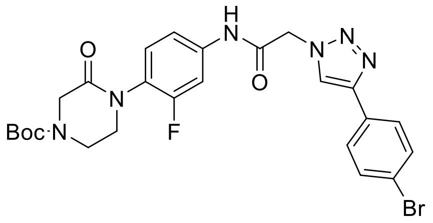

White solid; yield: 56 %; mp: 214-215 °C; IR (KBr) ν / cm^{-1} 3286, 3138, 2985, 2864, 1710, 1685, 1641, 1550, 1153, 567; ^{1}H NMR (400 MHz, DMSO-d_6) δ 1.44 (s, 9H), 3.62 (t, *J* = 5.0 Hz, 2H), 3.69 (t, *J* = 5.0 Hz, 2H), 4.09 (s, 2H), 5.44 (s, 2H), 7.32 – 7.45 (m, 2H), 7.60 – 7.70 (m, 3H), 7.81 – 7.88 (m, 2H), 8.66 (s, 1H), 10.80 (s, 1H). ^{13}C NMR (101 MHz, DMSO-d_6) δ 28.4, 41.8, 47.8, 49.6, 52.9, 80.2, 107.4 (d, *J* = 25.0 Hz), 115.8 (d, *J* = 3.0 Hz), 121.3, 123.9, 125.0 (d, *J* = 13.3 Hz), 127.6, 129.8 (d, *J* = 2.8 Hz), 130.4, 132.4, 139.4 (d, *J* = 10.6 Hz), 145.7, 153.7, 157.2 (d, *J* = 247.0 Hz), 165.1, 165.5; ^{19}F NMR (376 MHz, DMSO-d_6) δ -118.8 ppm; HRMS (FTMS + pESI) *m*/*z*, calcd. for $C_{25}H_{26}BrFN_6O_4$ [M-H]$^-$: 571.1110, found 471.1138.

2-(4-(4-bromophenyl)-1*H*-1,2,3-triazol-1-yl)-*N*-(3-fluoro-4-(3-oxomorpholino)phenyl)acetamide (**8o**)

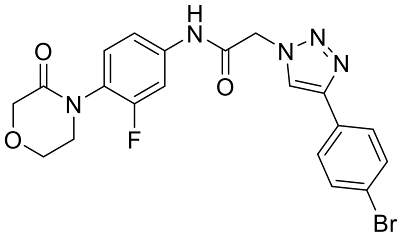

White solid; Yield: 66%; mp: 302-303 °C; IR (KBr) ν / cm-1 3277, 3116, 2983, 2862, 1715, 1647, 1552, 1130, 567; ^{1}H NMR (400 MHz, DMSO-d_6) δ 3.64 (t, *J* = 5.0 Hz, 2H), 3.97 (t, *J* = 5.1 Hz, 2H), 4.22 (s, 2H), 5.45 (s, 2H), 7.35 – 7.47 (m, 2H), 7.62 – 7.71 (m, 3H), 7.81 – 7.89 (m, 2H), 8.66 (s, 1H), 10.95 (s, 1H); ^{13}C NMR (101 MHz, DMSO-d_6) δ 49.4, 52.4, 63.5, 67.7, 107.0 (d, *J* = 25.1 Hz), 115.4 (d, *J* = 3.2 Hz), 120.8, 123.4, 124.0 (d, *J* = 13.4 Hz), 127.1, 129.3 (d, *J* = 2.6 Hz), 129.9, 131.9, 139.0 (d, *J* = 10.6 Hz), 145.2, 156.9 (d, *J* = 247.3 Hz), 164.6, 166.0; ^{19}F NMR (376 MHz, DMSO-d_6) δ -118.7 ppm; HRMS (FTMS + pESI) *m*/*z*, calcd. for $C_{20}H_{17}BrFN_5O_3$ [M-H]$^-$: 472.0426, found 472.0438.

2-(4-(4-bromophenyl)-1*H*-1,2,3-triazol-1-yl)-*N*-(3-fluoro-4-(3-oxothiomorpholino)phenyl)acetamide (**8p**)

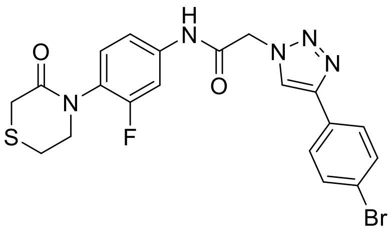

White solid; yield: 83%; mp: 280-281 °C; IR (KBr) ν / cm^{-1} 3280, 3118, 2976, 2858, 1701, 1652, 1551, 1112, 565; ^{1}H NMR (400 MHz, DMSO-d_6) δ 3.02 (t, *J* = 5.7 Hz, 2H), 3.43 (s, 2H), 3.86 (t, *J* = 5.8 Hz, 2H), 5.45 (s, 2H), 7.30 – 7.42 (m, 2H), 7.61 – 7.70 (m, 3H), 7.81 – 7.88 (m, 2H), 8.67 (s, 1H), 11.01 (s, 1H). ^{13}C NMR (101 MHz, DMSO-d_6) δ 26.0, 29.4, 51.4, 52.4, 106.9 (d, *J* = 25.1 Hz), 115.3 (d, J = 3.0 Hz), 120.8, 123.4, 125.4 (d, *J* = 13.4 Hz), 127.1, 129.3 (d, *J* = 2.6 Hz), 129.9, 131.9, 138.7 (d, *J* = 10.7 Hz), 145.2, 156.7 (d, *J* = 246.7 Hz), 164.6, 166.7; ^{19}F NMR (376 MHz, DMSO-d_6) δ -118.7 ppm; HRMS (FTMS + pESI) *m*/*z*, calcd. for $C_{20}H_{17}BrFN_5O_2S$ [M-H]$^-$: 488.0197, found 488.0214.

N-(3-fluoro-4-(2-oxopiperidin-1-yl)phenyl)-2-(4-(*p*-tolyl)-1*H*-1,2,3-triazol-1-yl)acetamide (**8q**)

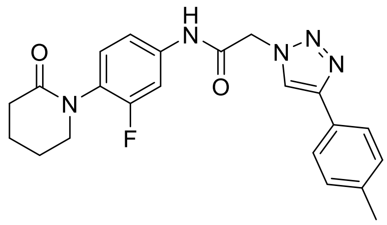

White solid; yield: 30%; mp: 301-302 °C; IR (KBr) ν / cm^{-1} 3275, 3136, 2947, 2862, 1712, 1631, 1554, 1172; ^{1}H NMR (400 MHz, DMSO-d_6) δ 1.78 – 1.90 (m, 4H), 2.33 (s, 3H), 2.38 (t, *J* = 6.2 Hz, 2H), 3.50 (t, *J* = 5.5 Hz, 2H), 5.40 (s, 2H), 7.07 – 7.42 (m, 4H), 7.49 – 7.69 (m, 1H), 7.72 – 7.84 (m, 2H), 8.54 (s, 1H), 10.75 (s, 1H). ^{13}C NMR (101 MHz, DMSO-d_6) δ 20.8, 20.9, 22.9, 32.2, 50.8, 52.3, 106.9 (d, *J* = 25.3 Hz), 115.3 (d, *J* = 3.1 Hz), 122.6, 125.1, 126.2 (d, *J* = 13.4 Hz), 127.9, 129.4 (d, *J* = 3.0 Hz), 129.5, 137.2, 138.4 (d, J = 10.7 Hz), 146.3, 156.9 (d, *J* = 246.5 Hz), 164.6, 168.8; ^{19}F NMR (376 MHz, DMSO-d_6) δ -119.0 ppm; HRMS (FTMS + pESI) *m*/*z*, calcd. for $C_{22}H_{22}FN_5O_2$ [M+H]$^+$: 408.1831, found: 408.1849.

tert-butyl-4-(2-fluoro-4-(2-(4-(*p*-tolyl)-1*H*-1,2,3-triazol-1-yl)acetamido)phenyl)-3-oxopiperazine-1-carboxylate (**8r**)

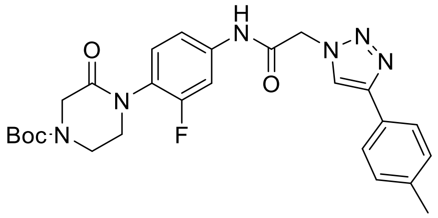

White solid; yield: 57%; mp: 204-205 °C; IR (KBr) ν / cm^{-1} 3302, 3113, 2978, 2866, 1693, 1608, 1547, 1153; 1H NMR (400 MHz, DMSO-*d*$_6$) δ 1.44 (s, 9H), 2.33 (s, 3H), 3.62 (t, *J* = 5.3 Hz, 2H), 3.69 (t, *J* = 5.5 Hz, 2H), 4.10 (s, 2H), 5.41 (s, 2H), 7.27 (d, *J* = 7.9 Hz, 2H), 7.34 – 7.44 (m, 2H), 7.65 (dd, *J* = 12.5, 2.1 Hz, 1H), 7.74 – 7.80 (m, 2H), 8.54 (s, 1H), 10.80 (s, 1H).^{13}C NMR (101 MHz, DMSO-*d*$_6$) δ 20.8, 28.0, 41.3, 47.8, 49.1, 52.3, 79.8, 107.0 (d, *J* = 25.1 Hz), 115.4 (d, *J* = 3.2 Hz), 122.6, 124.5 (d, *J* = 13.3 Hz), 125.1, 127.9, 129.3 (d, *J* = 3.0 Hz), 129.5, 137.2, 138.9 (d, *J* = 10.6 Hz), 146.3, 153.2, 156.7 (d, *J* = 247.0 Hz), 164.7, 165.0; ^{19}F NMR (376 MHz, DMSO-*d*$_6$) δ -118.8 ppm; HRMS (FTMS + pESI) *m*/*z*, calcd. for $C_{26}H_{29}FN_6O_4$ [M-H]$^-$: 507.2161, found 507.2176.

N-(3-fluoro-4-(3-oxomorpholino)phenyl)-2-(4-(*p*-tolyl)-1*H*-1,2,3-triazol-1-yl)acetamide (**8s**)

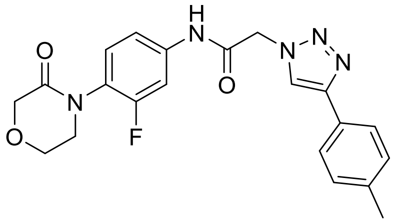

White solid; yield: 31%; mp: 282-283 °C; IR (KBr) ν / cm^{-1} 3278, 3116, 2981, 2862, 1712, 1651, 1150; ^{1}H NMR (400 MHz, DMSO-*d*$_6$) δ 2.33 (s, 3H), 3.64 (t, *J* = 4.9 Hz, 2H), 3.97 (t, *J* = 5.1 Hz, 2H), 4.22 (s, 2H), 5.41 (s, 2H), 7.27 (d, *J* = 7.9 Hz, 2H), 7.35 – 7.46 (m, 2H), 7.65 (dd, *J* = 12.4, 2.2 Hz, 1H), 7.77 (d, *J* = 8.1 Hz, 2H), 8.54 (s, 1H), 10.80 (s, 1H); ^{13}C NMR (101 MHz, DMSO-*d*$_6$) δ 20.8, 49.4, 52.3, 63.5, 67.7, 107.0 (d, *J* = 25.0 Hz), 115.4 (d, *J* = 3.2 Hz), 122.6, 124.1 (d, *J* = 13.4 Hz), 125.1, 127.9, 129.3 (d, *J* = 2.8 Hz), 129.5, 137.2, 139.0 (d, *J* = 10.7 Hz), 146.3, 156.9 (d, *J* = 247.2 Hz), 164.7, 166.0; ^{19}F NMR (376 MHz, DMSO-*d*$_6$) δ -118.7 ppm; HRMS (FTMS + pESI) *m*/*z*, calcd. for $C_{21}H_{20}FN_5O_3$ [M-H]$^-$: 408.1477, found 408.1486.

N-(3-fluoro-4-(3-oxothiomorpholino)phenyl)-2-(4-(*p*-tolyl)-1*H*-1,2,3-triazol-1-yl)acetamide (**8t**)

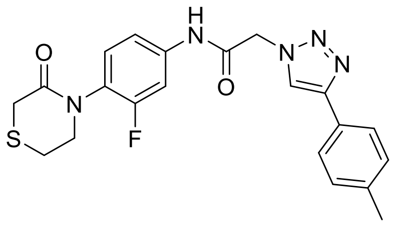

Yellow pale solid; yield: 57%; mp: 285-286 °C; IR (KBr) ν / cm^{-1} 3502, 3279, 3120, 2974, 2858, 1701, 1654, 1551, 1110; ^{1}H NMR (400 MHz, DMSO-*d*$_6$) δ 2.33 (s, 3H), 3.02 (t, *J* = 5.8 Hz, 2H), 3.44 (s, 2H), 3.86 (t, *J* = 5.8 Hz, 2H), 5.41 (s, 2H), 7.27 (d, *J* = 7.8 Hz, 2H), 7.32 – 7.38 (m, 2H), 7.60 – 7.68 (m, 1H), 7.77 (d, *J* = 7.9 Hz, 2H), 8.54 (s, 1H), 10.79 (s, 1H).^{13}C NMR (101 MHz, DMSO-*d*$_6$) 20.8, 26.0, 29.4, 51.4, 52.3, 106.9 (d, *J* = 25.2 Hz), 115.3 (d, *J* = 3.0 Hz), 122.6, 125.1, 125.4 (d, *J* = 13.4 Hz), 127.9, 129.3 (d, J = 2.5 Hz), 129.5, 137.2, 138.7 (d, *J* = 10.7 Hz), 146.3, 156.7 (d, *J* = 246.7 Hz), 164.7, 166.7; ^{19}F NMR (376 MHz, DMSO-*d*$_6$) -118.6 ppm; HRMS (FTMS + pESI) *m*/*z*, calcd. for $C_{21}H_{20}FN_5O_2S$ [M-H]$^-$: 424.1249, found 424.1265.

11. Spectroscopic data of new synthesized compounds

11.1. *¹H NMR and ¹³C NMR spectra*

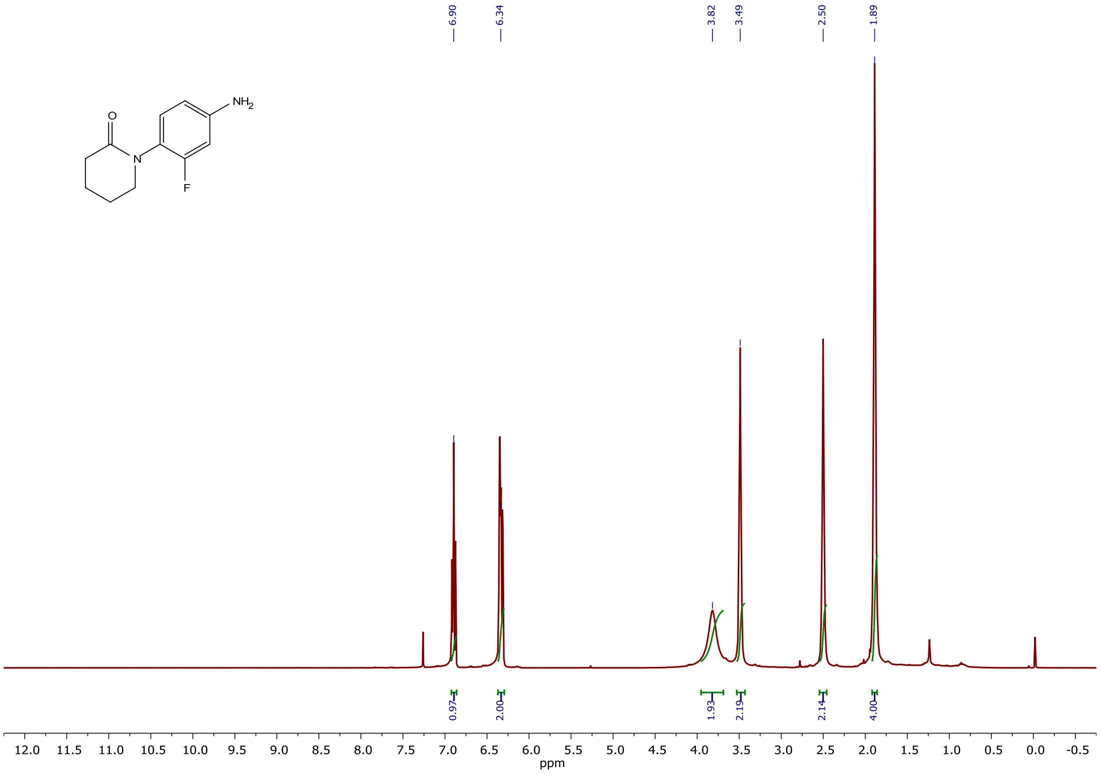

Figure S8. ¹H NMR (400 MHz, $CDCl_3$) of 1-(4-amino-2-fluorophenyl)piperidin-2-one (**3a**).

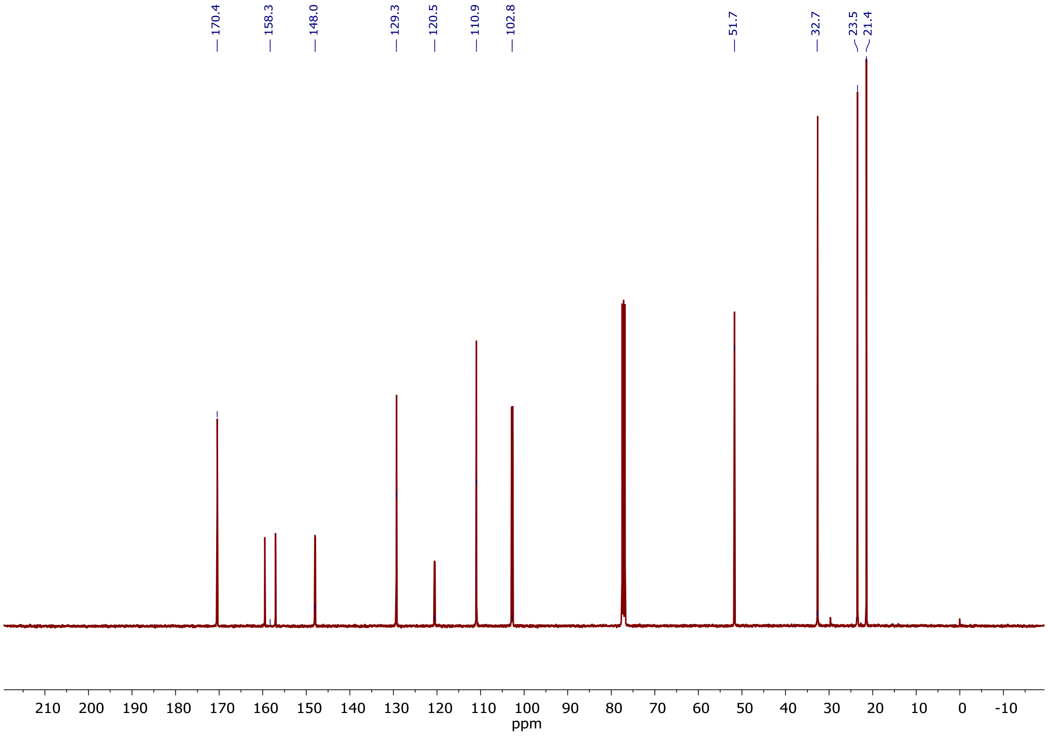

Figure S9. ¹³C NMR (100 MHz, $CDCl_3$) of 1-(4-amino-2-fluorophenyl)piperidin-2-one (**3a**).

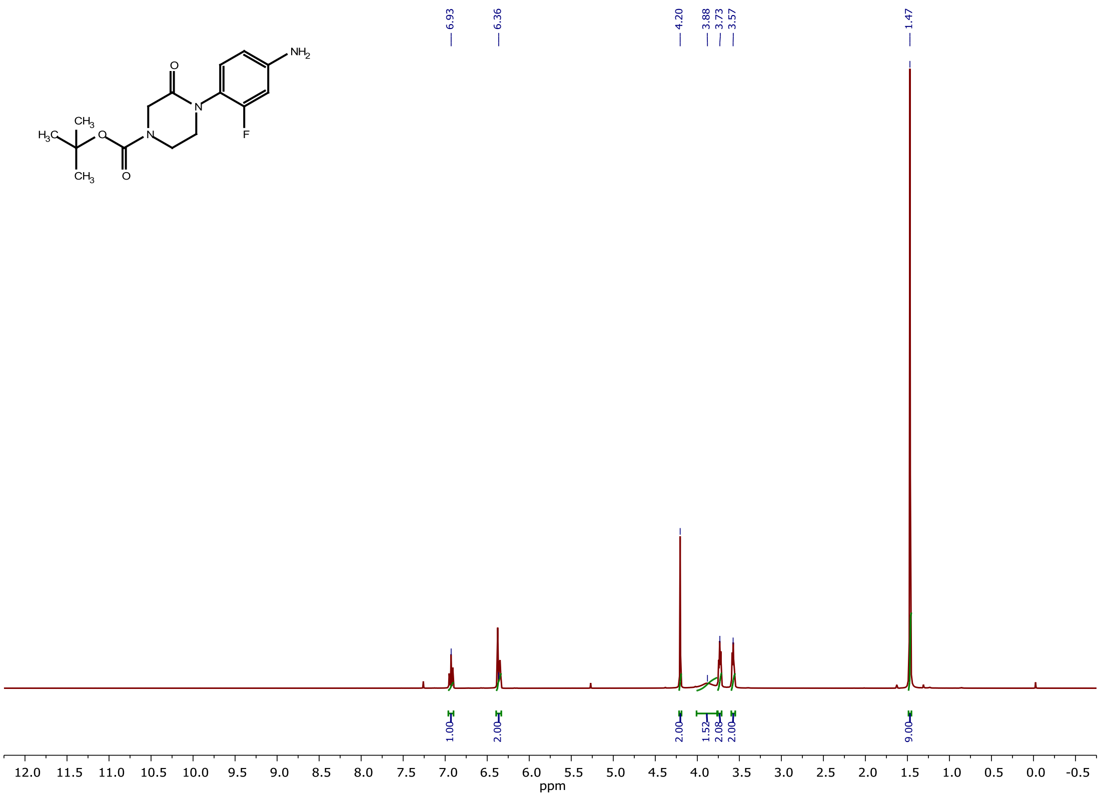

Figure S10. ^{1}H NMR (400 MHz, $CDCl_3$) of *tert*-butyl 4-(4-amino-2-fluorophenyl)-3-oxopiperazine-1-carboxylate (**3b**).

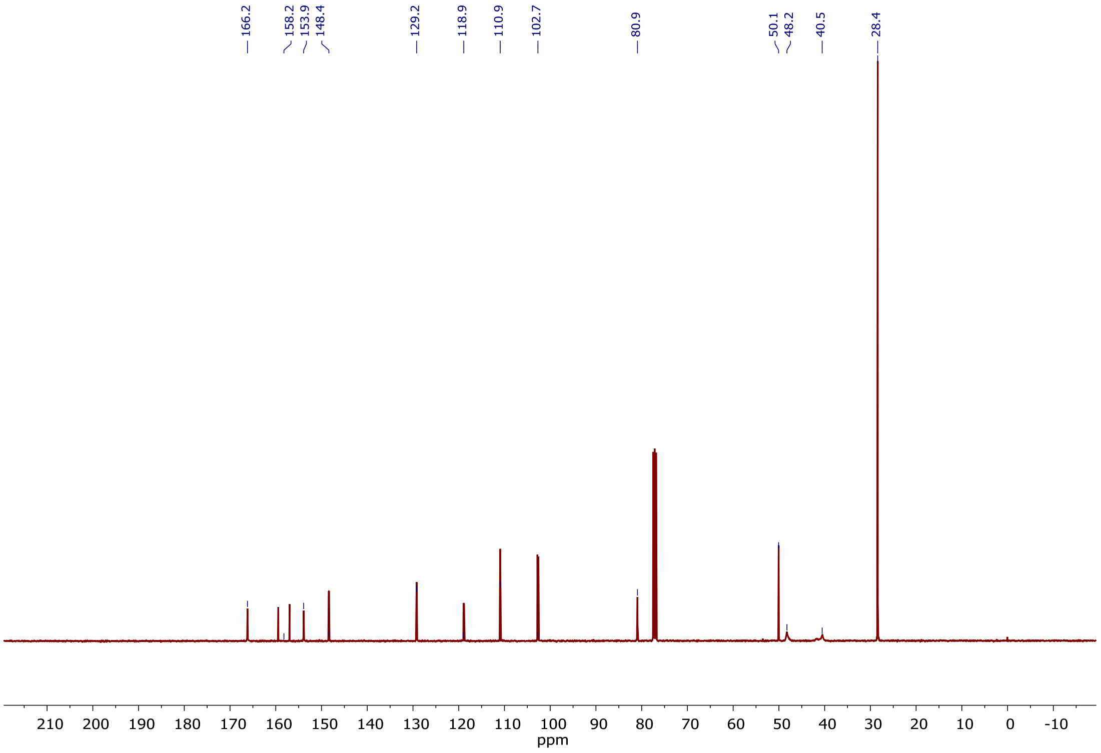

Figure S11. ^{13}C NMR (100 MHz, $CDCl_3$) of *tert*-butyl 4-(4-amino-2-fluorophenyl)-3-oxopiperazine-1-carboxylate (**3b**).

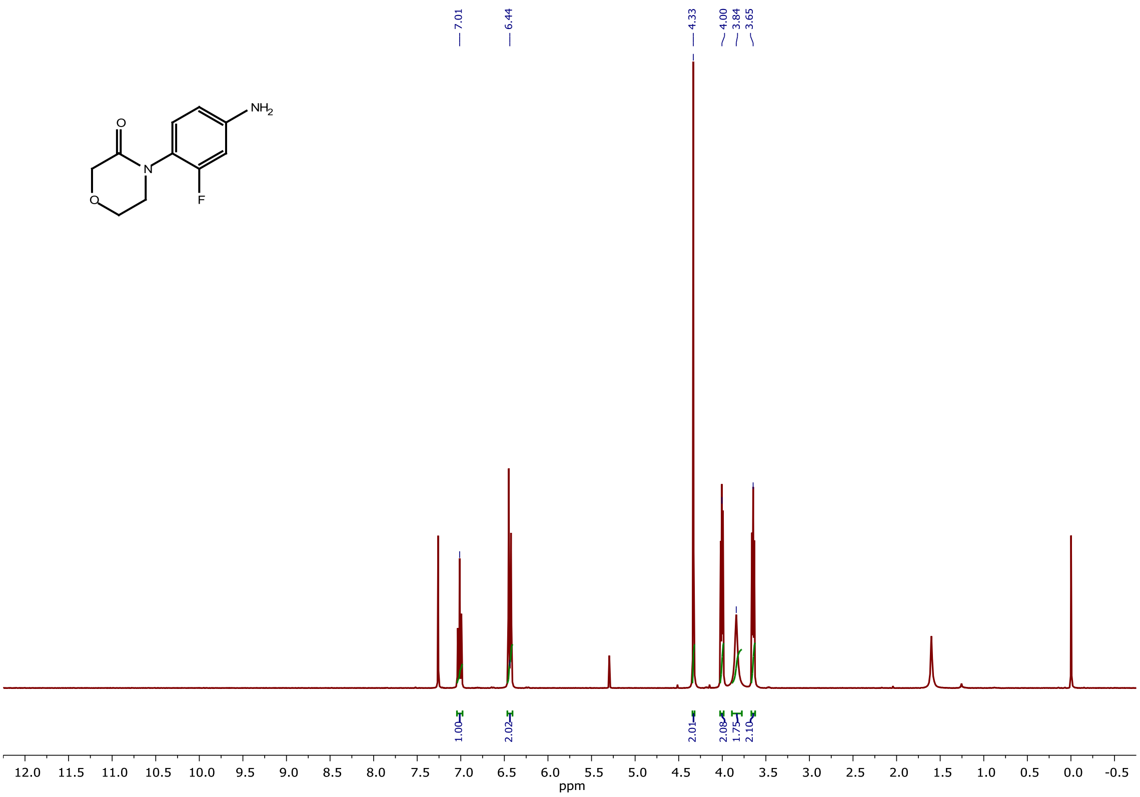

Figure S12. ^1H NMR (400 MHz, $CDCl_3$) of 4-(4-amino-2-fluorophenyl)morpholin-3-one (**3c**).

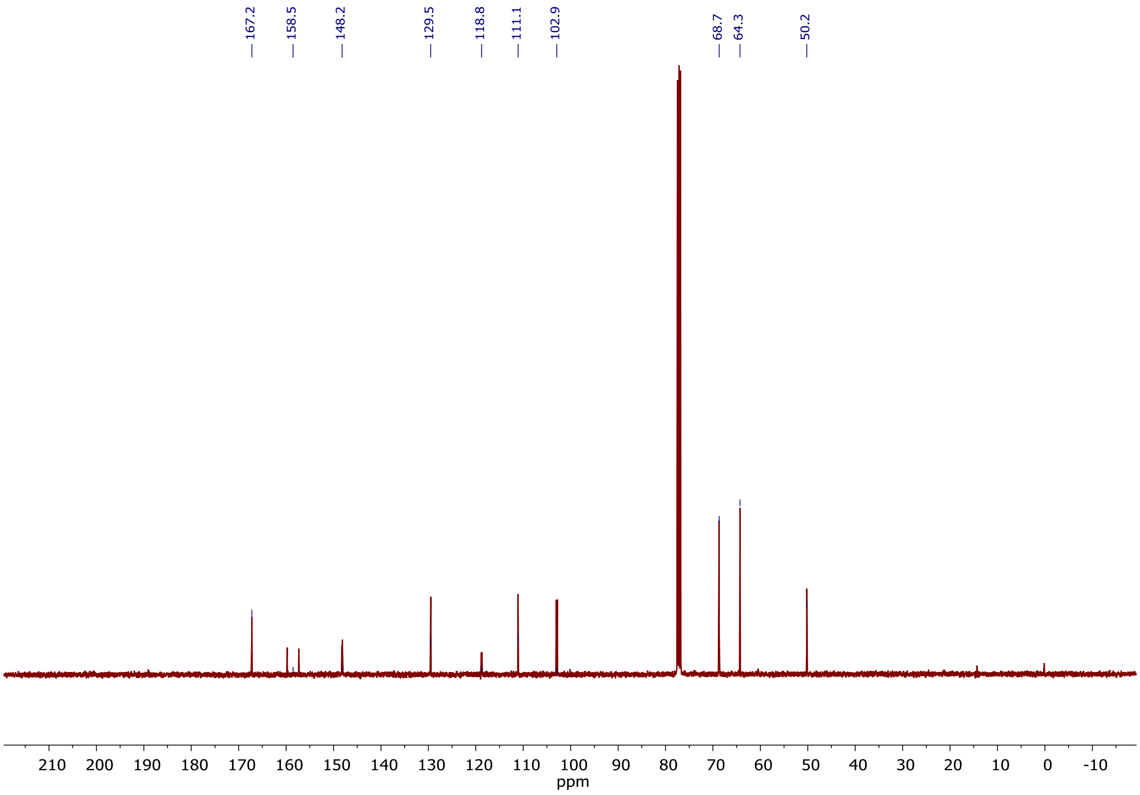

Figure S13. ^{13}C NMR (100 MHz, $CDCl_3$) of 4-(4-amino-2-fluorophenyl)morpholin-3-one (**3c**).

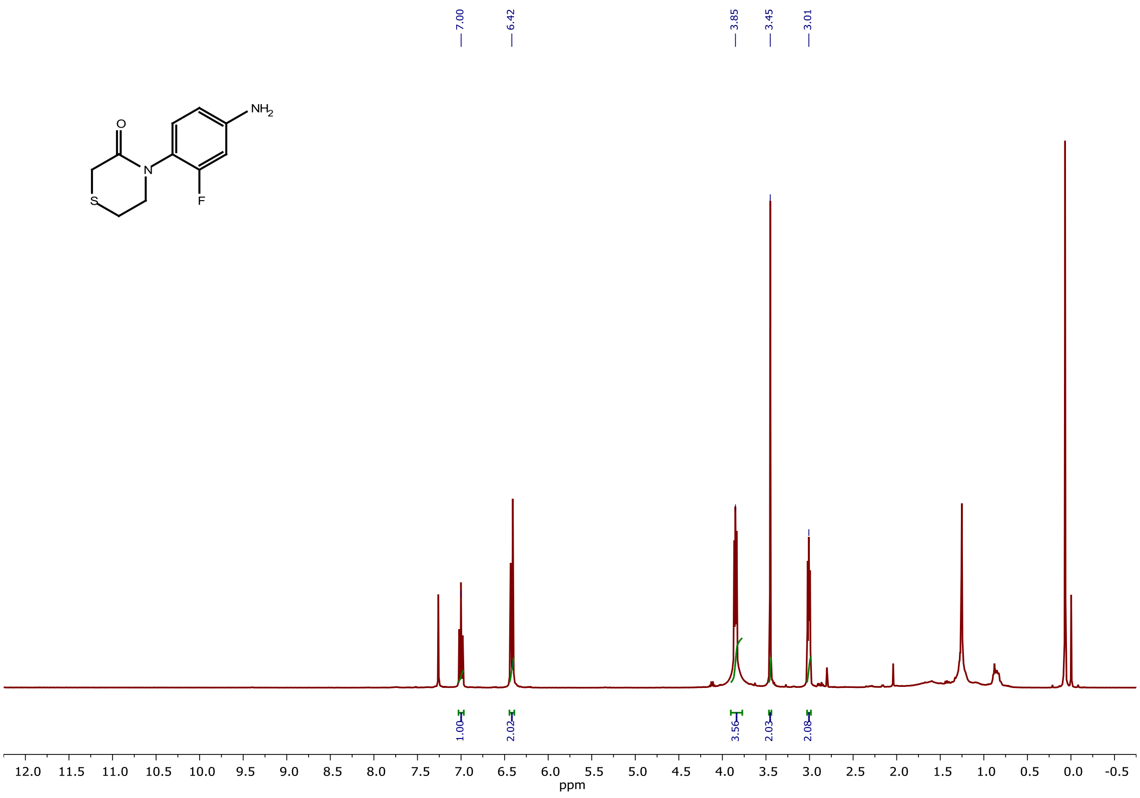

Figure S14. ^{1}H NMR (400 MHz, $CDCl_3$) of 4-(4-amino-2-fluorophenyl)thiomorpholin-3-one (**3d**).

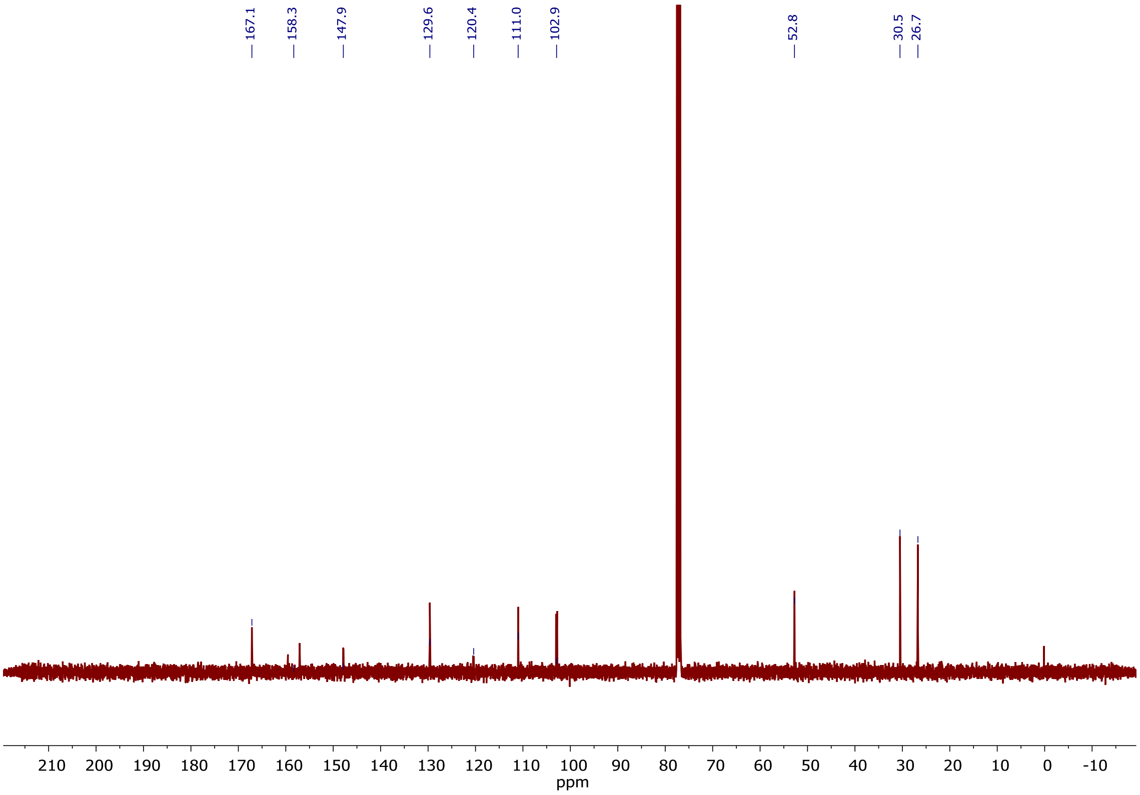

Figure S15. ^{13}C NMR (100 MHz, $CDCl_3$) of 4-(4-amino-2-fluorophenyl)thiomorpholin-3-one (**3d**).

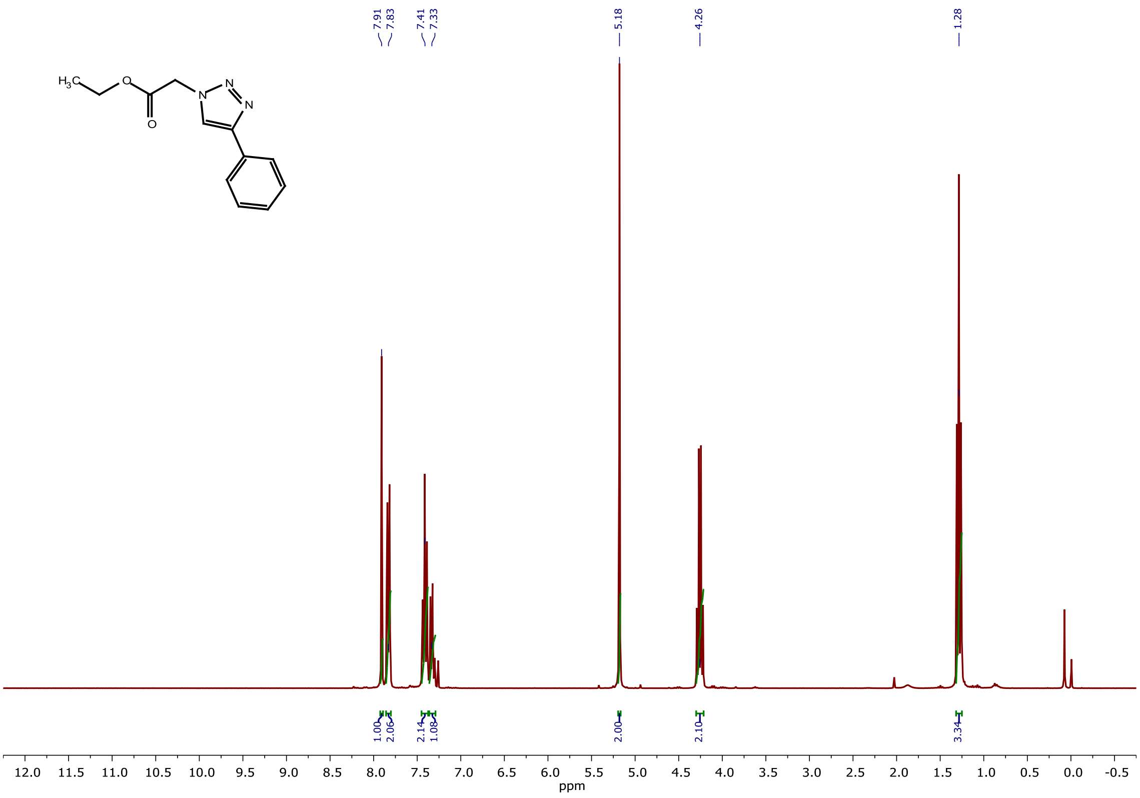

Figure S16. ^{1}H NMR (300 MHz, $CDCl_3$) of ethyl 2-(4-phenyl-1*H*-1,2,3-triazol-1-yl)acetate (**6a**).

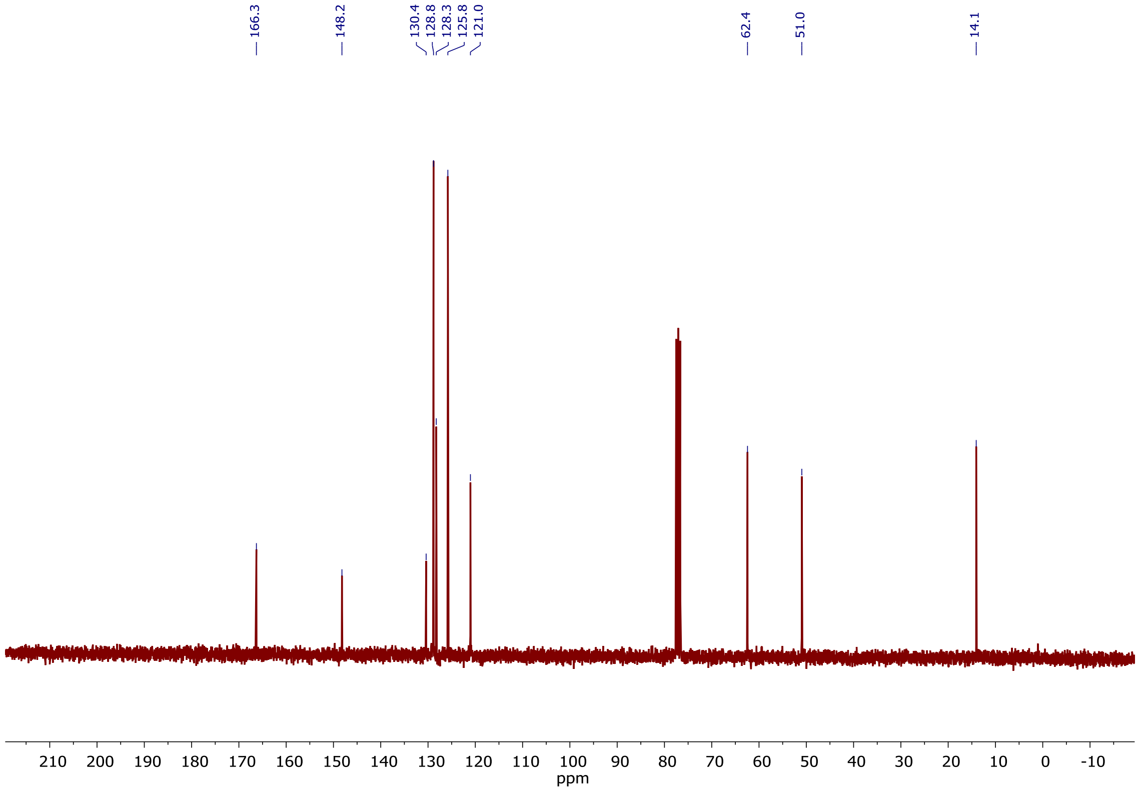

Figure S17. ^{13}C NMR (75 MHz, $CDCl_3$) of ethyl 2-(4-phenyl-1*H*-1,2,3-triazol-1-yl)acetate (**6a**).

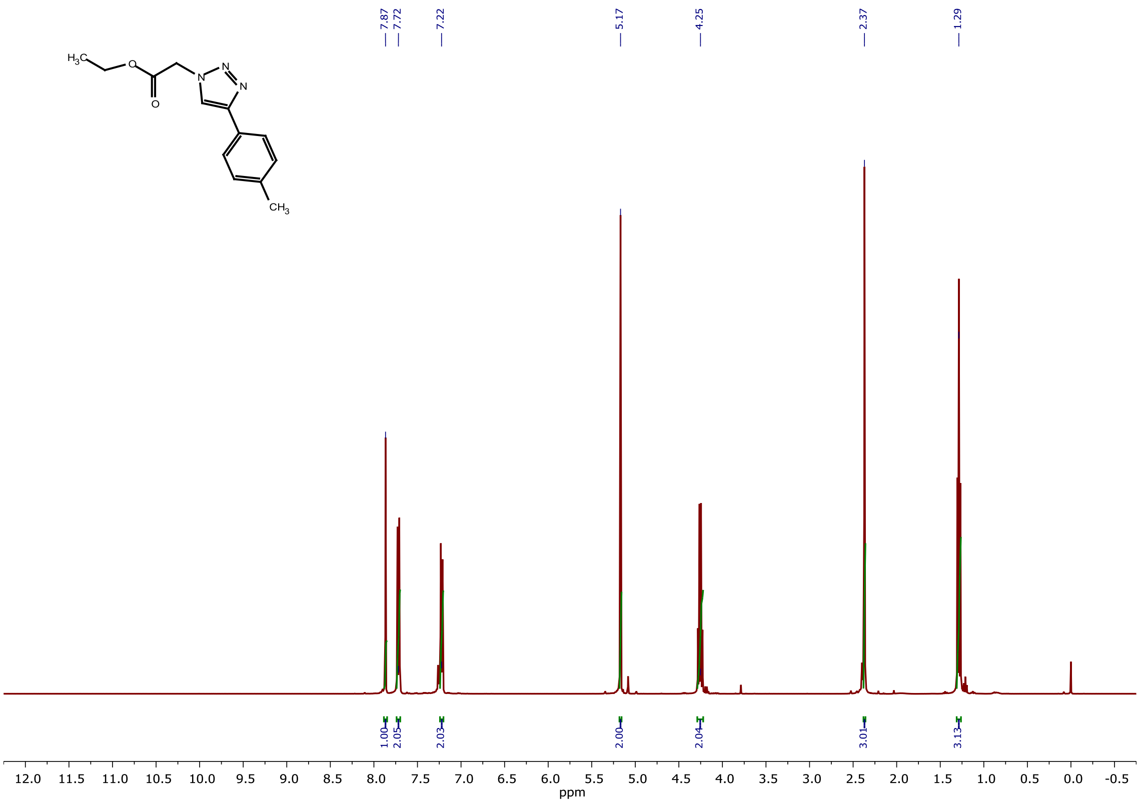

Figure S18. ^{1}H NMR (400 MHz, $CDCl_3$) Ethyl 2-(4-(*p*-tolyl)-1*H*-1,2,3-triazol-1-yl)acetate **(6b).**

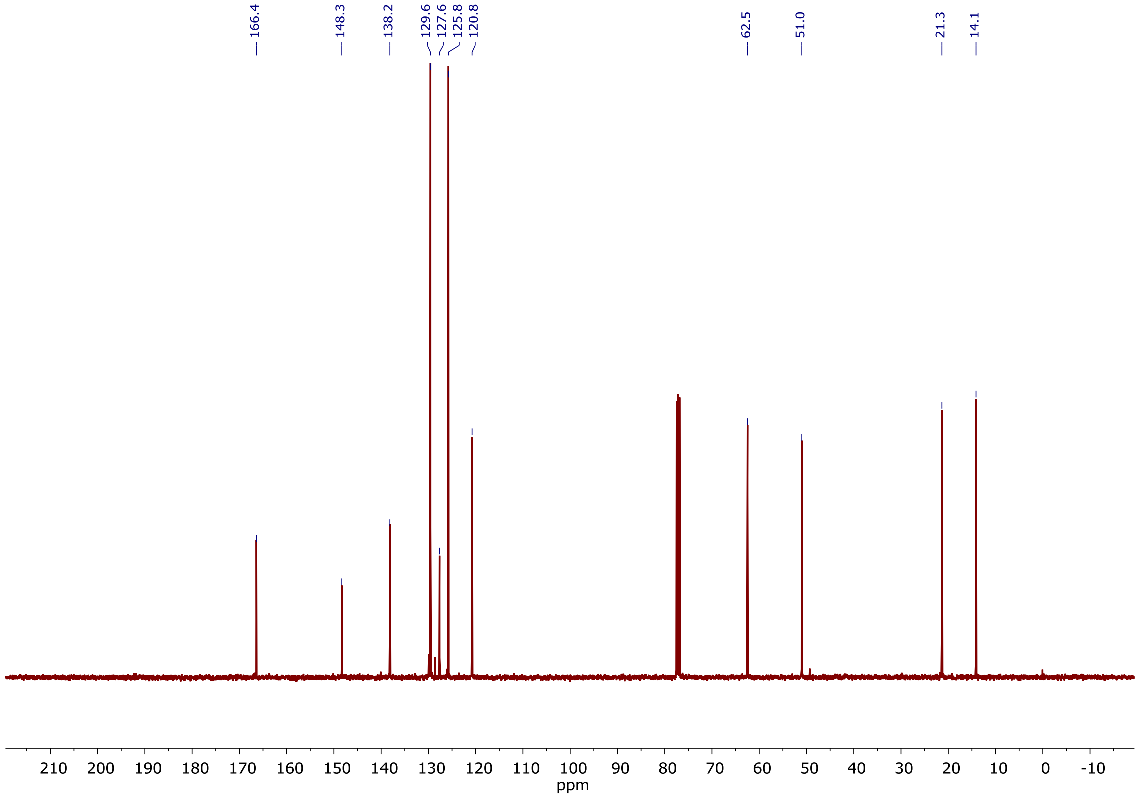

Figure S19. ^{13}C NMR (101 MHz, $CDCl_3$) Ethyl 2-(4-(*p*-tolyl)-1*H*-1,2,3-triazol-1-yl)acetate **(6b).**

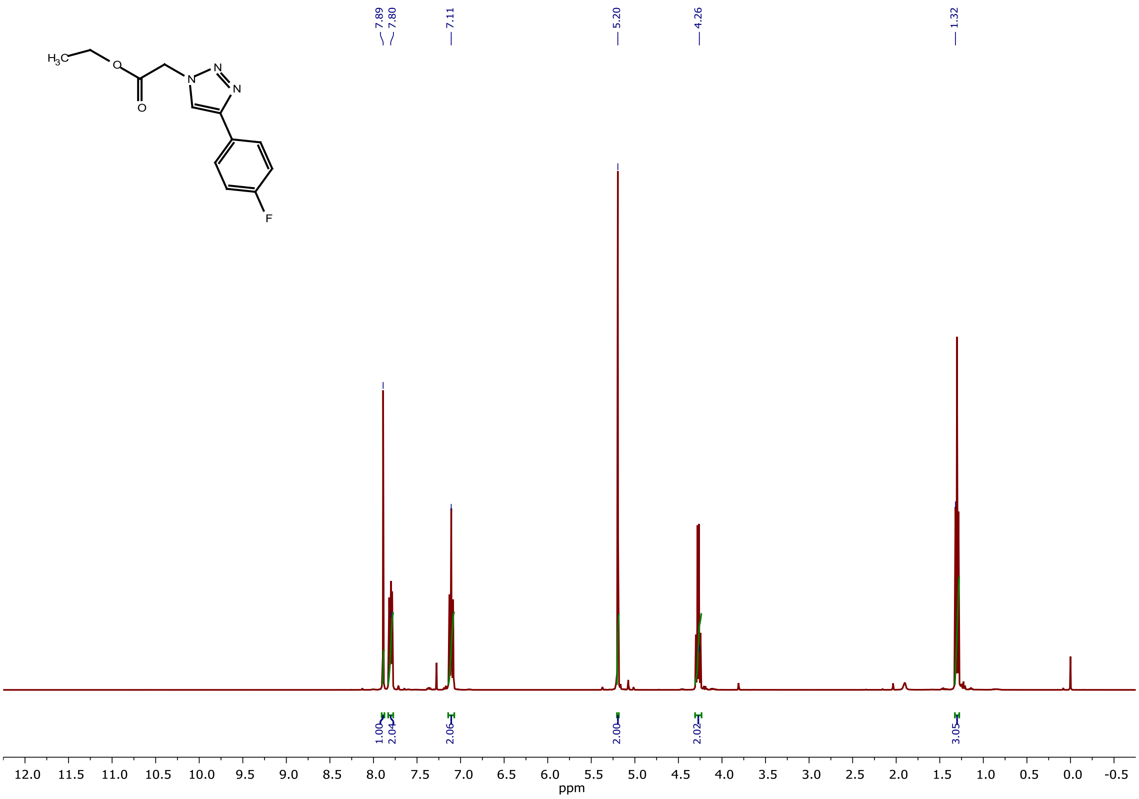

Figure S20. ^{1}H NMR (400 MHz, $CDCl_3$) of ethyl 2-(4-(4-chlorophenyl)-1*H*-1,2,3-triazol-1-yl)acetate **(6c).**

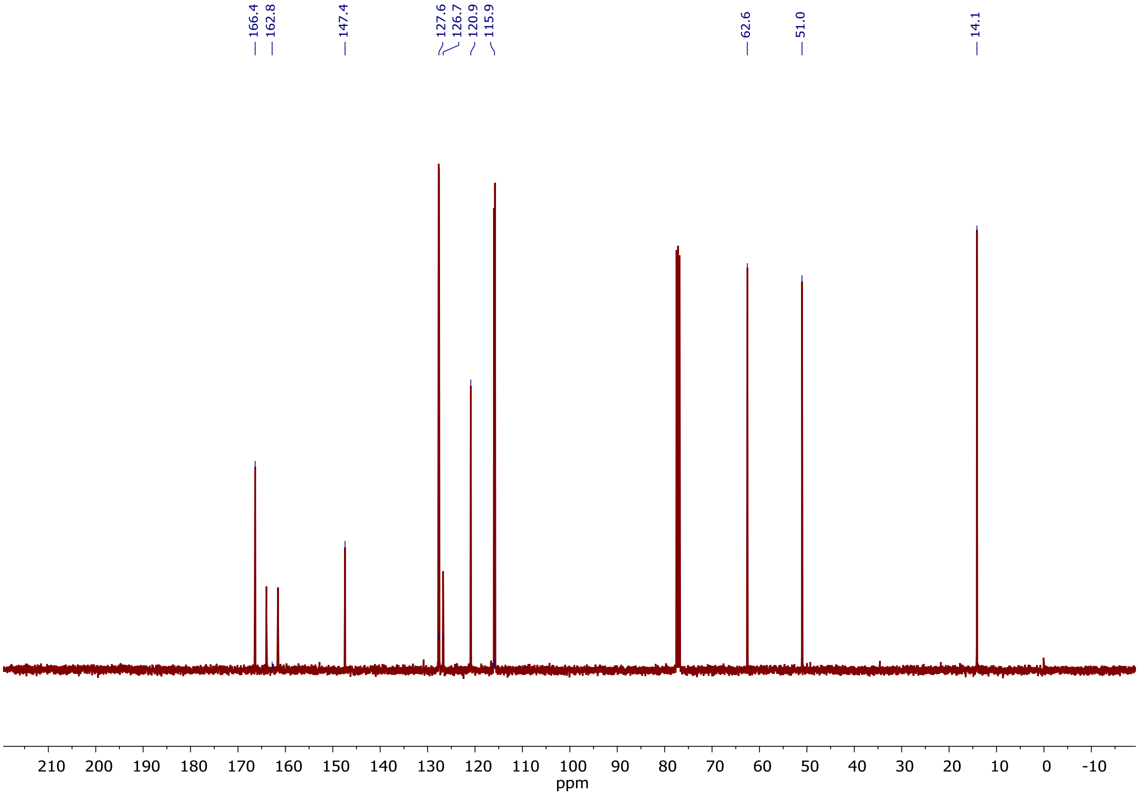

Figure S21. ^{13}C NMR (75 MHz, $CDCl_3$) of ethyl 2-(4-(4-chlorophenyl)-1*H*-1,2,3-triazol-1-yl)acetate **(6c).**

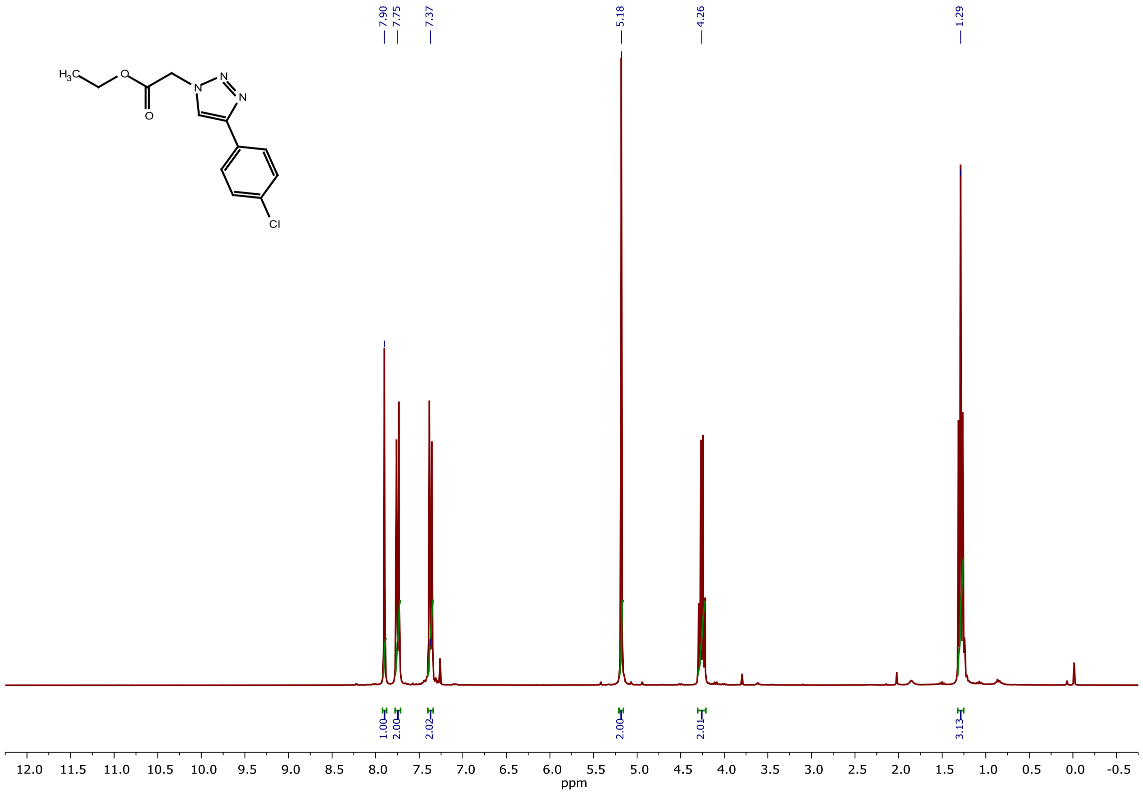

Figure S22. ^{1}H NMR (400 MHz, $CDCl_3$) of ethyl 2-(4-(4-chlorophenyl)-1*H*-1,2,3-triazol-1-yl)acetate (**6d**).

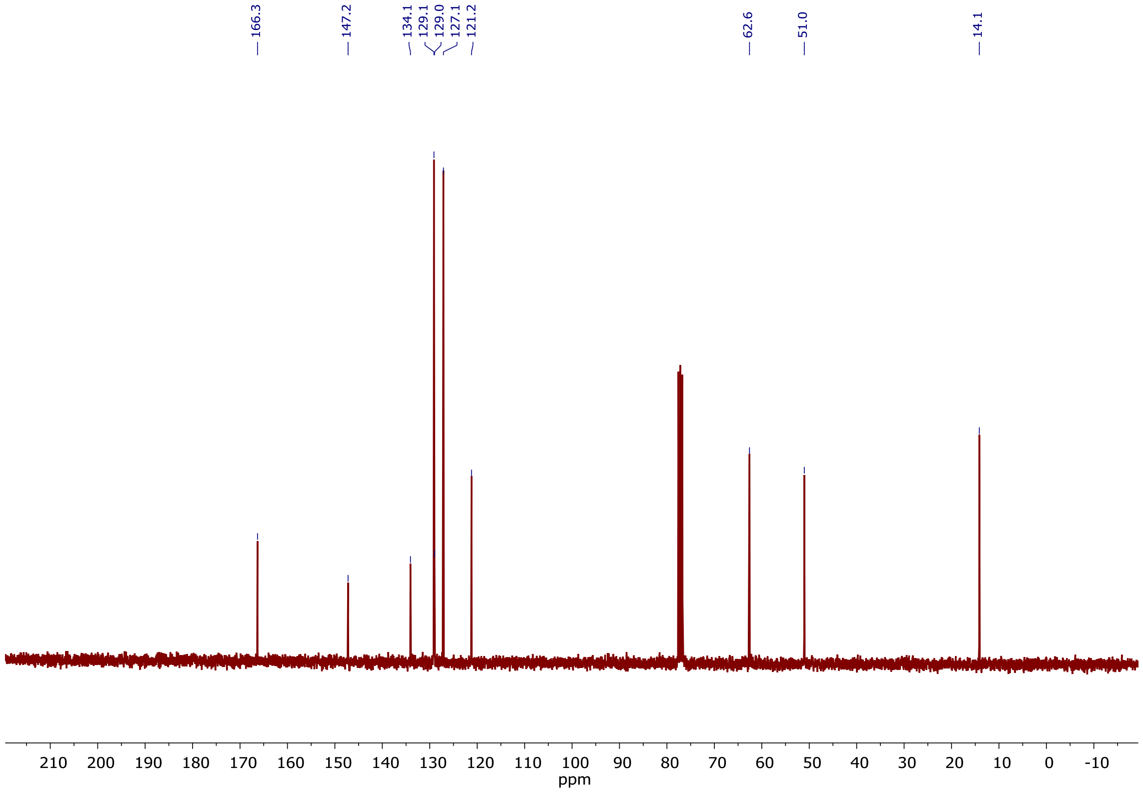

Figure S23. ^{13}C NMR (75 MHz, $CDCl_3$) of ethyl 2-(4-(4-chlorophenyl)-1*H*-1,2,3-triazol-1-yl)acetate (**6d**).

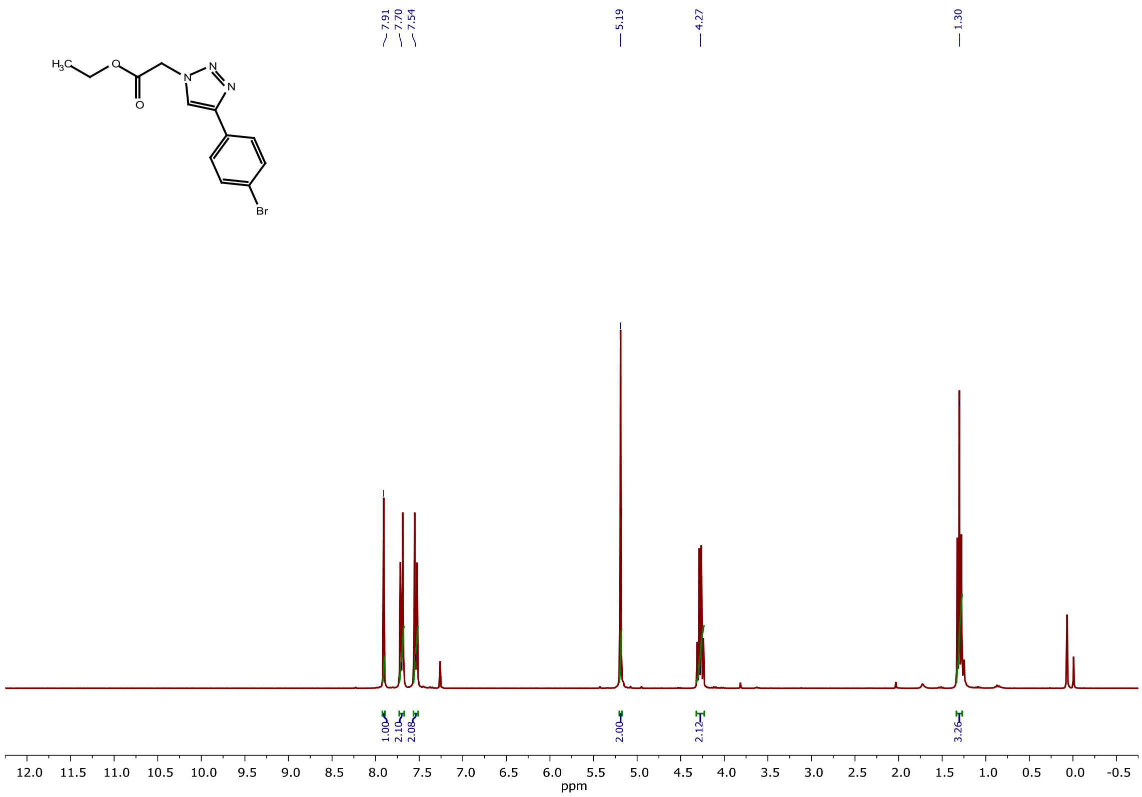

Figure S24. ^{1}H NMR (400 MHz, $CDCl_3$) of ethyl 2-(4-(4-bromophenyl)-1*H*-1,2,3-triazol-1-yl)acetate (**6e**).

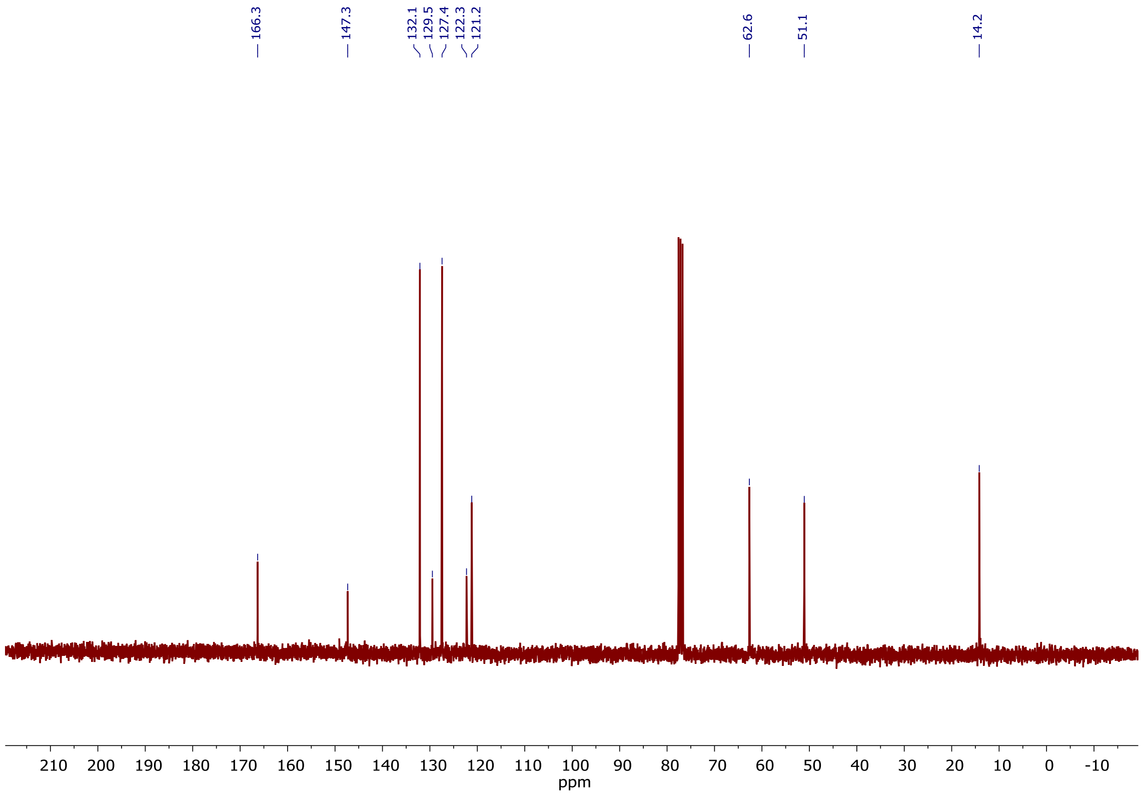

Figure S25. ^{13}C NMR (75 MHz, $CDCl_3$) of ethyl 2-(4-(4-bromophenyl)-1*H*-1,2,3-triazol-1-yl)acetate (**6e**).

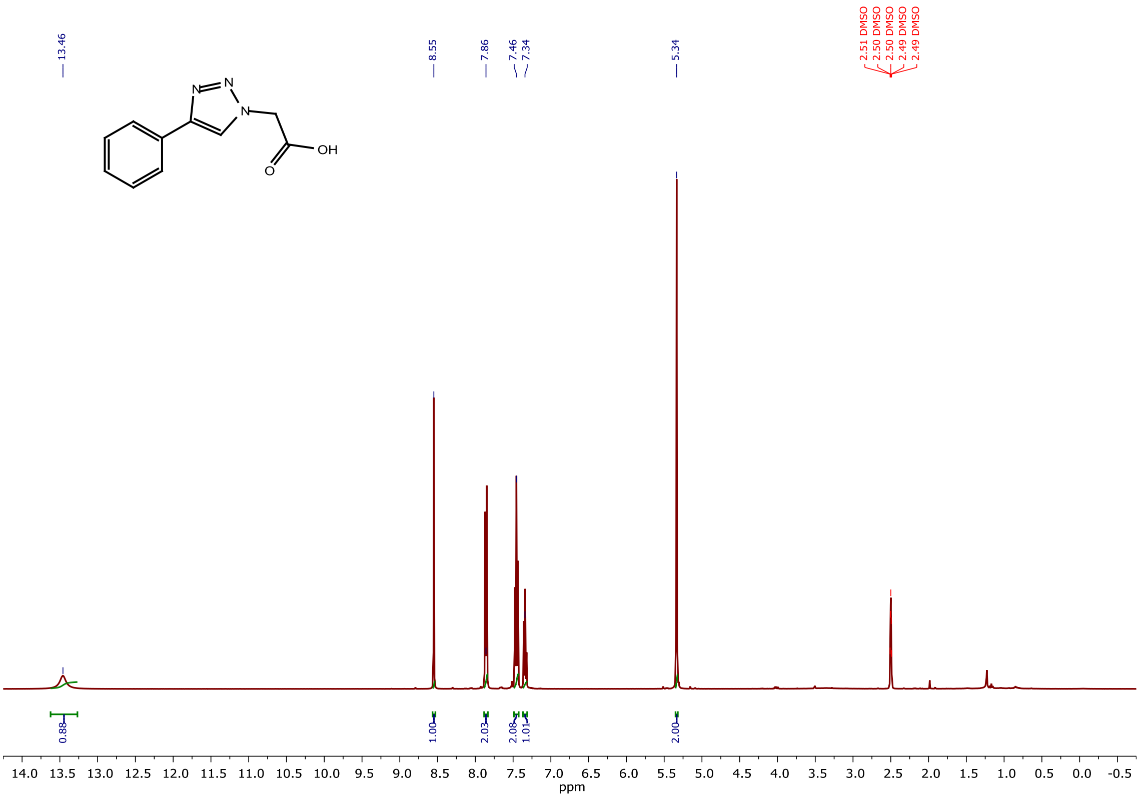

Figure S26. ^{1}H NMR (400 MHz, DMSO-d_6) of 2-(4-Phenyl-1*H*-[1,2,3]triazolyl)acetic Acid (**7a**).

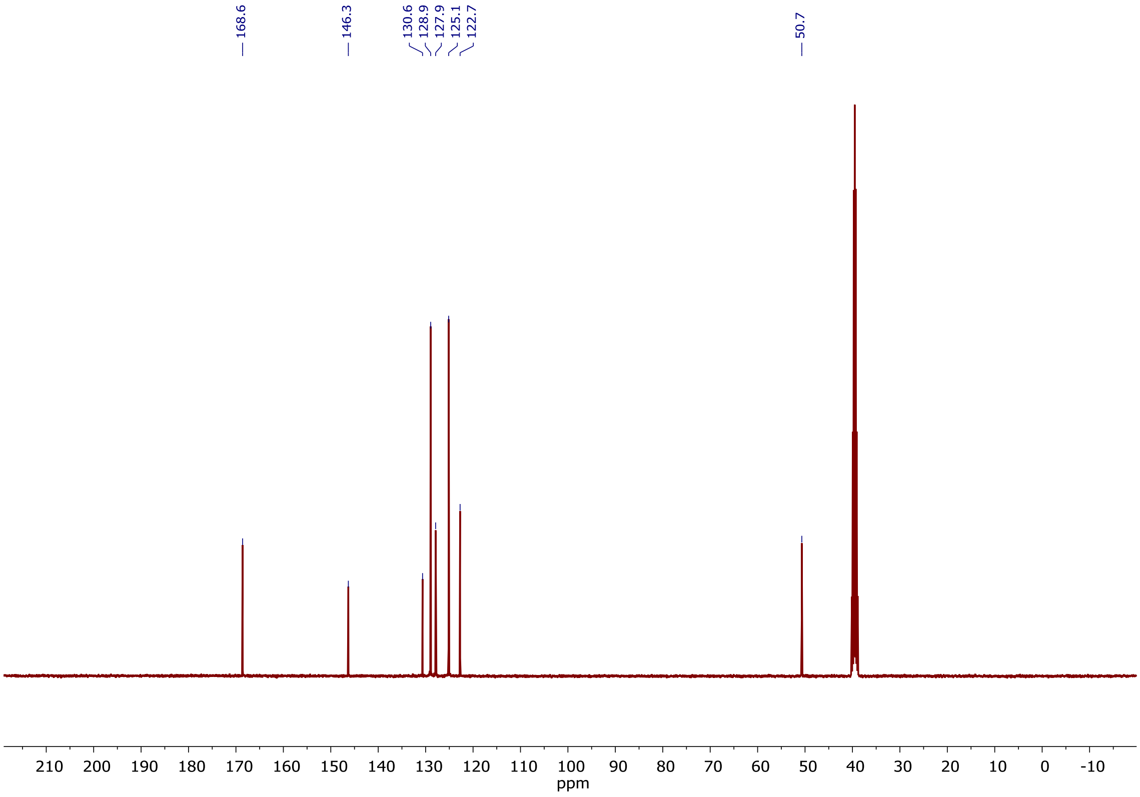

Figure S27. ^{13}C NMR (100 MHz, DMSO-d_6) of 2-(4-Phenyl-1*H*-[1,2,3]triazolyl)acetic Acid (**7a**).

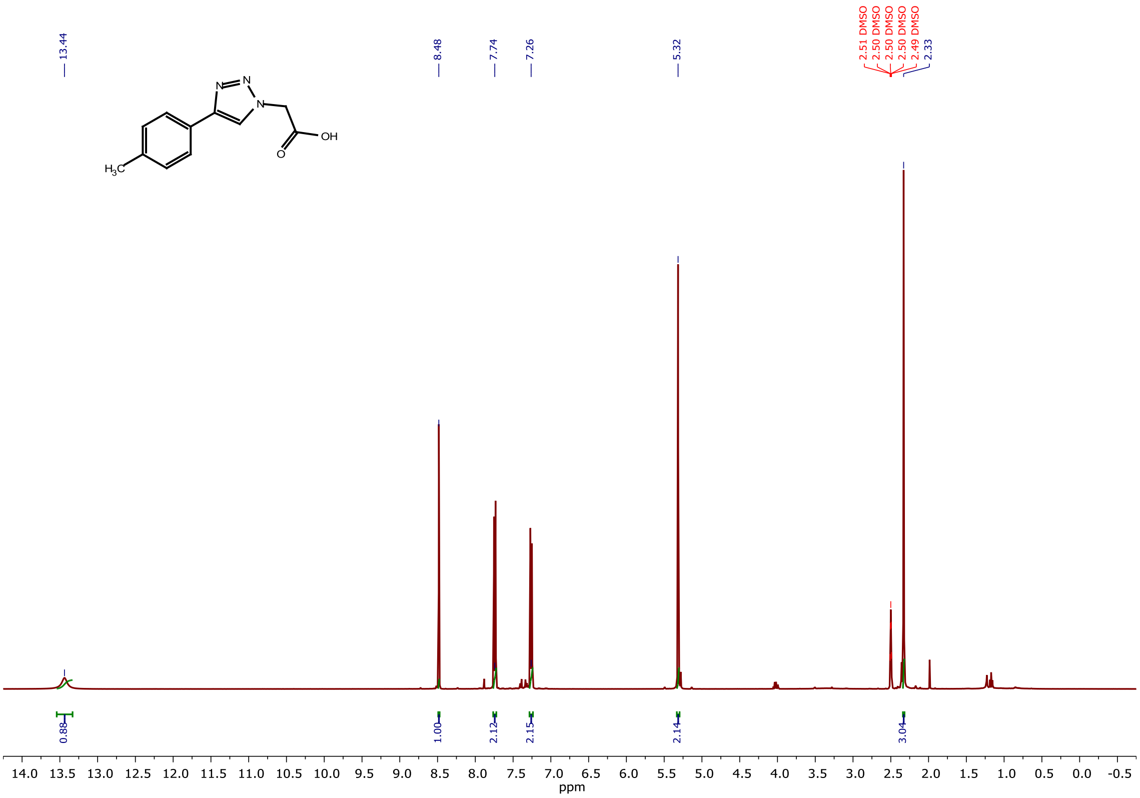

Figure S28. ¹H NMR (400 MHz, DMSO-d_6) of 2-(4-(*p*-tolyl)-1*H*-1,2,3-triazol-1-yl)acetic acid (**7b**).

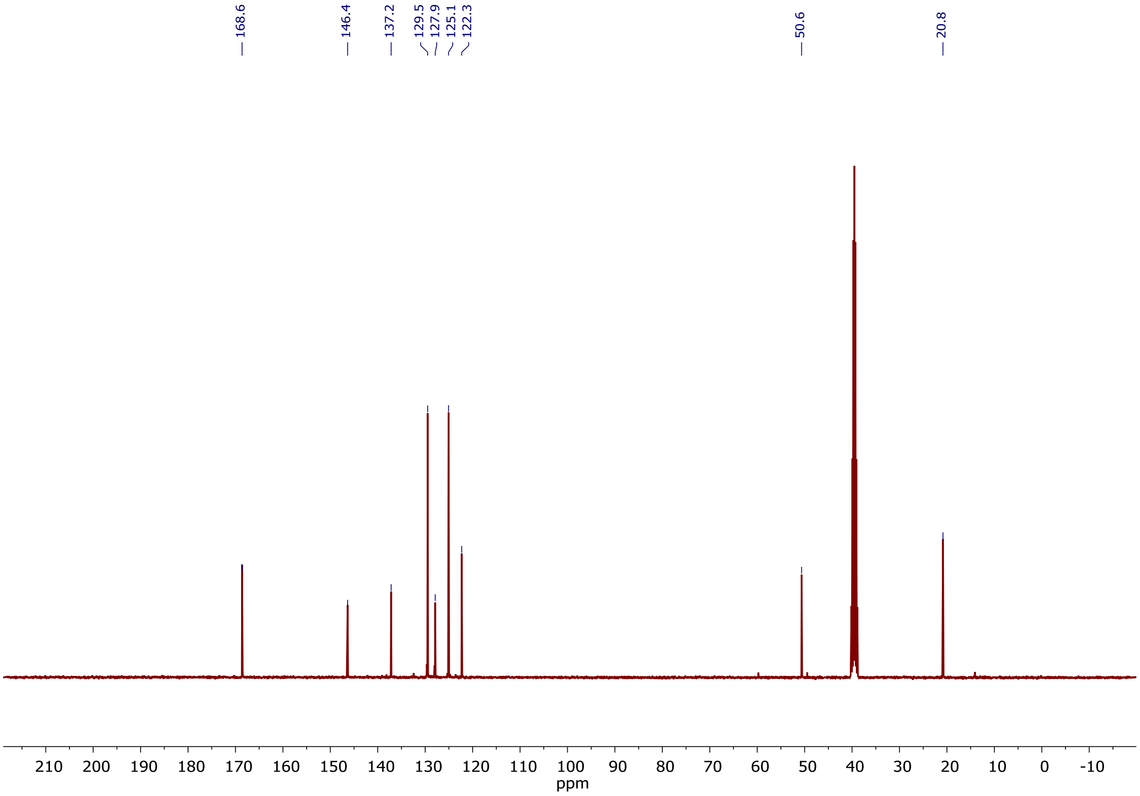

Figure S29. ^{13}C NMR (100 MHz, DMSO-*d*6) of 2-(4-(*p*-tolyl)-1*H*-1,2,3-triazol-1-yl)acetic acid (**7b**).

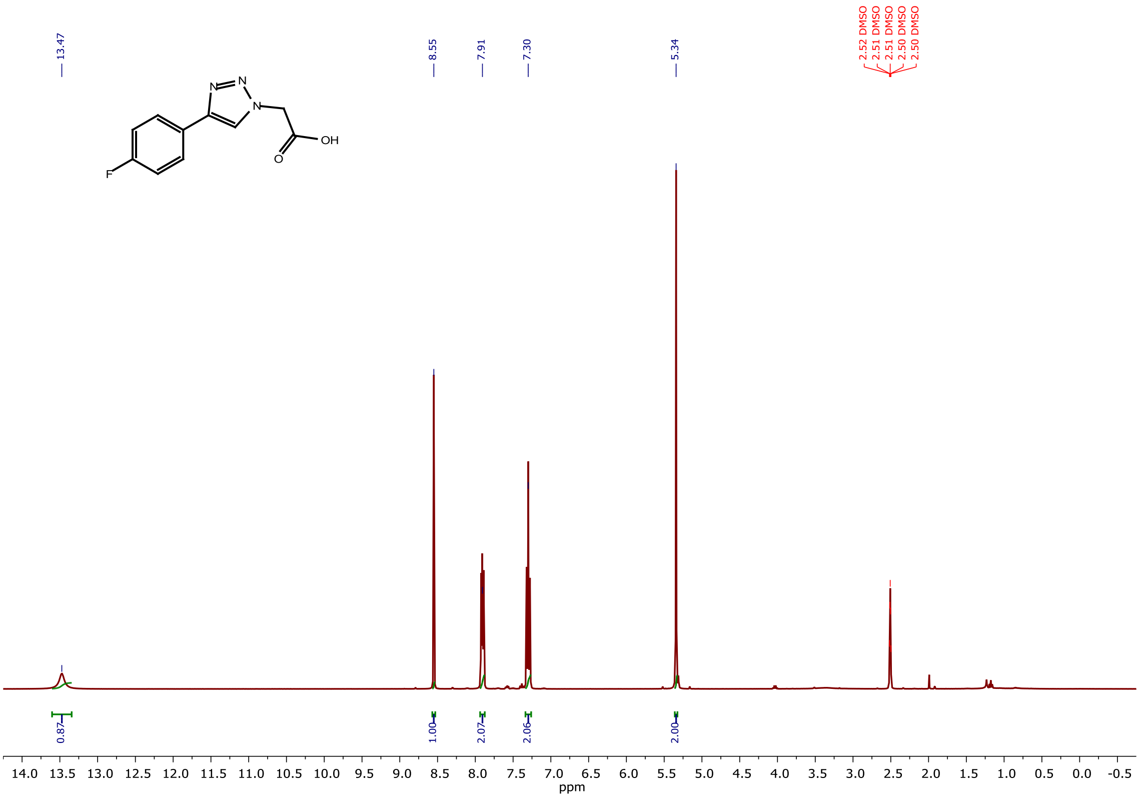

Figure S30. ^{1}H NMR (400 MHz, DMSO-*d*6) of 2-(4-(4-fluorophenyl)-1*H*-1,2,3-triazol-1-yl)acetic acid (**7c**).

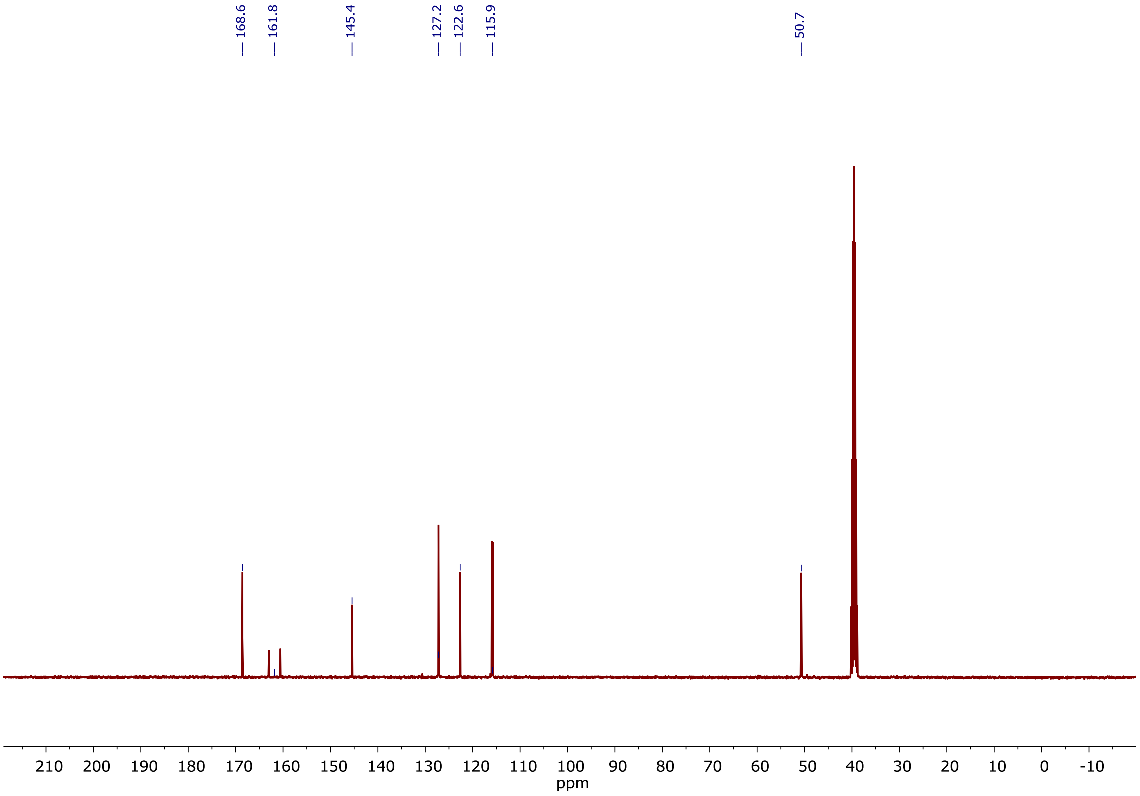

Figure S31. ^{13}C NMR (100 MHz, DMSO-*d*6) of 2-(4-(4-fluorophenyl)-1*H*-1,2,3-triazol-1-yl)acetic acid (**7c**).

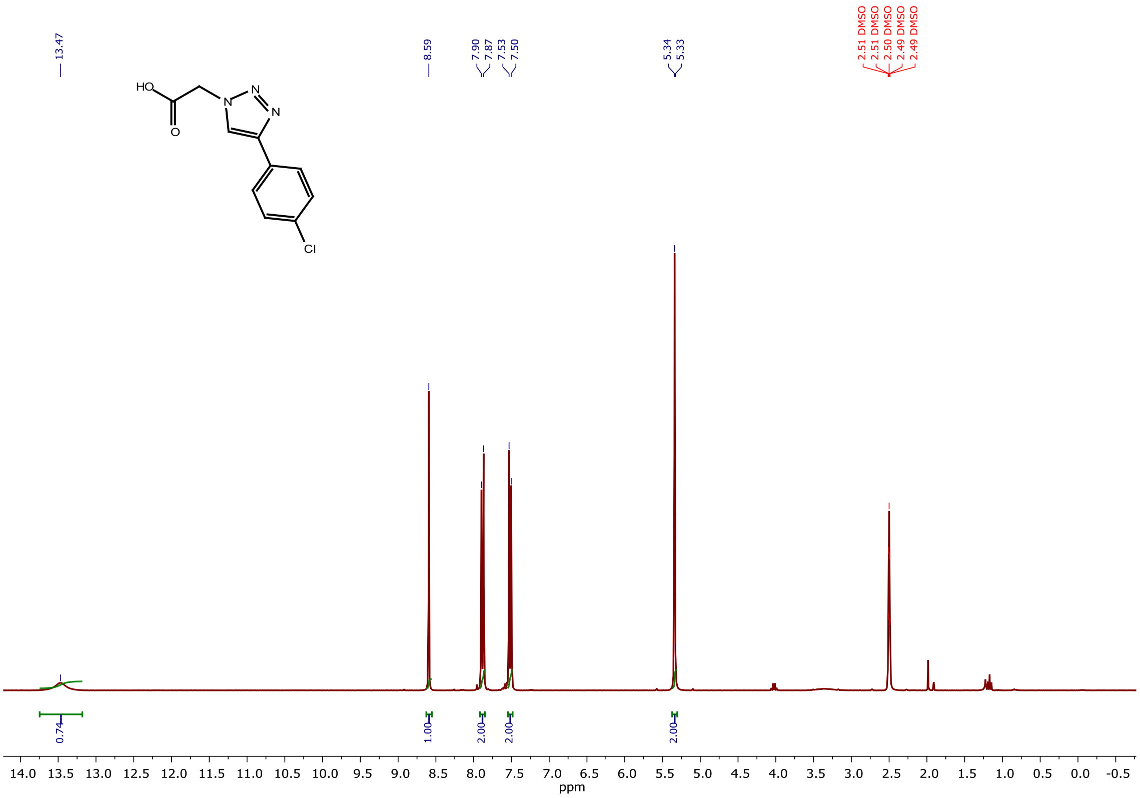

Figure S32. ^{1}H NMR (300 MHz, DMSO-*d*6) of 2-(4-(4-chlorophenyl)-1*H*-1,2,3-triazol-1-yl)acetic acid (**7d**).

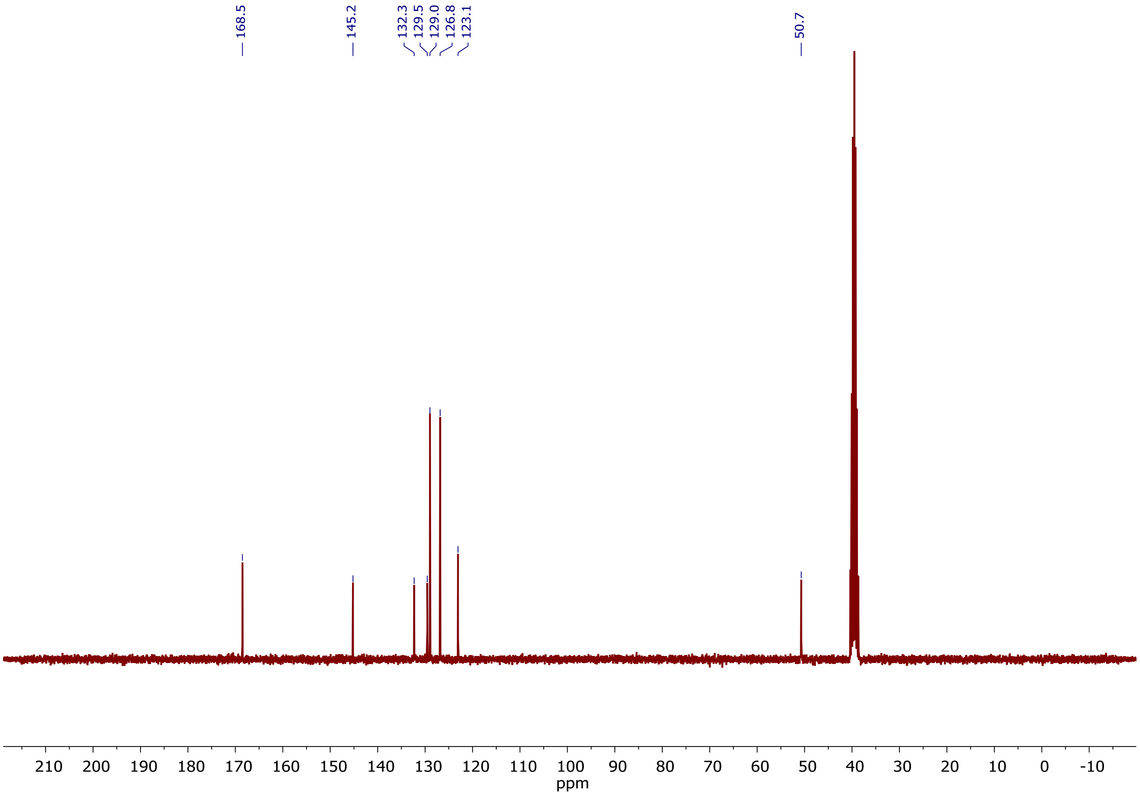

Figure S33. ^{13}C NMR (75 MHz, DMSO-*d*6) of 2-(4-(4-chlorophenyl)-1*H*-1,2,3-triazol-1-yl)acetic acid (**7d**).

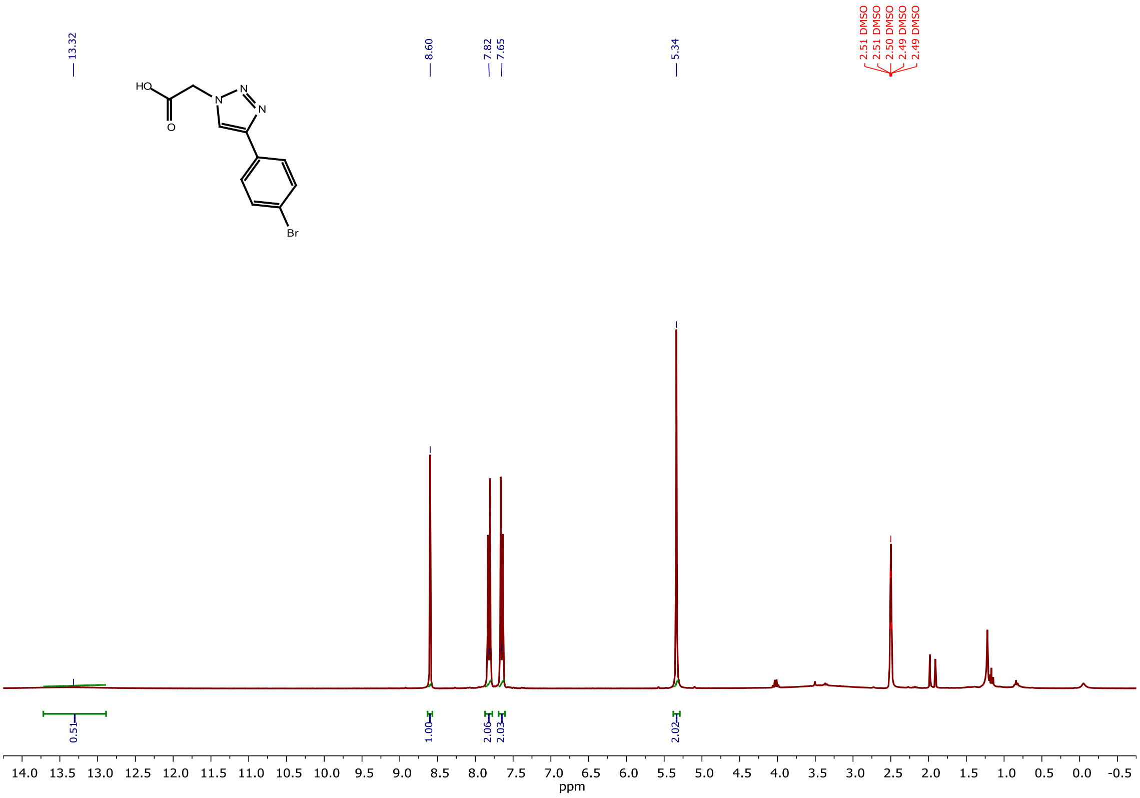

Figure S34. ^{1}H NMR (300 MHz, DMSO-d_6) of 2-(4-(4-bromophenyl)-1*H*-1,2,3-triazol-1-yl)acetic acid (**7e**).

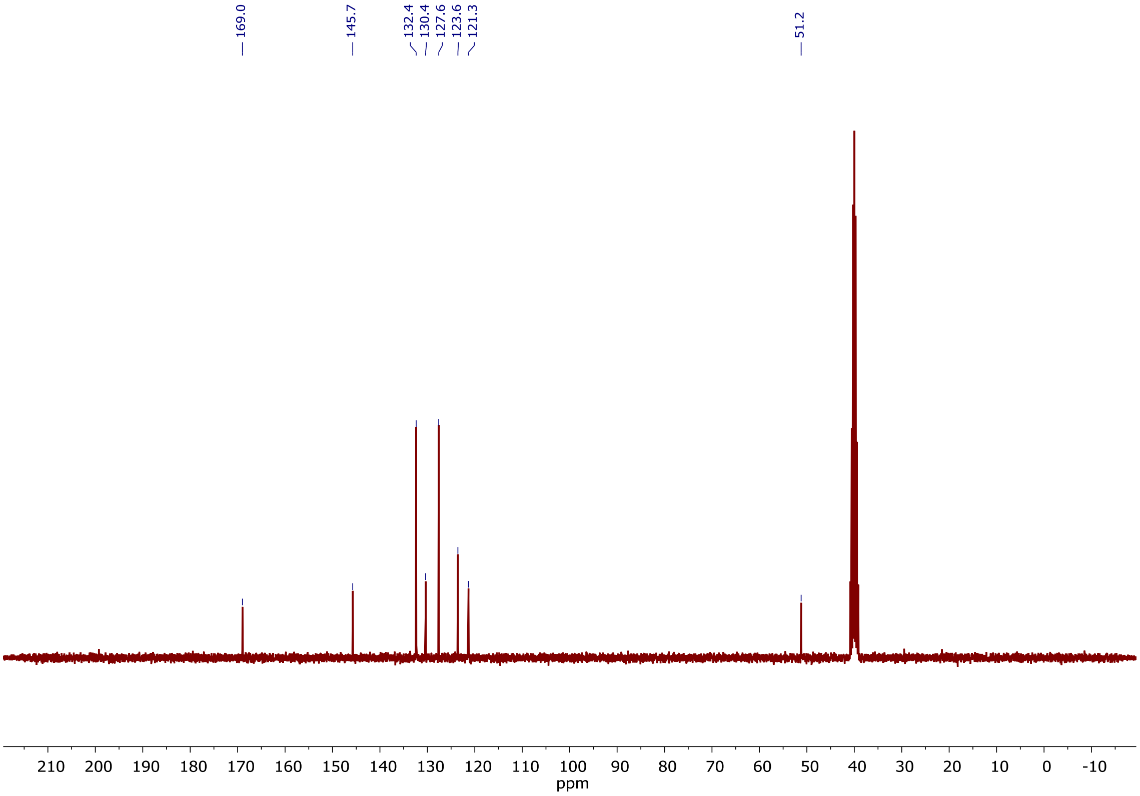

Figure S35. ^{13}C NMR (75 MHz, DMSO-*d*6) of 2-(4-(4-bromophenyl)-1*H*-1,2,3-triazol-1-yl)acetic acid (**7e**).

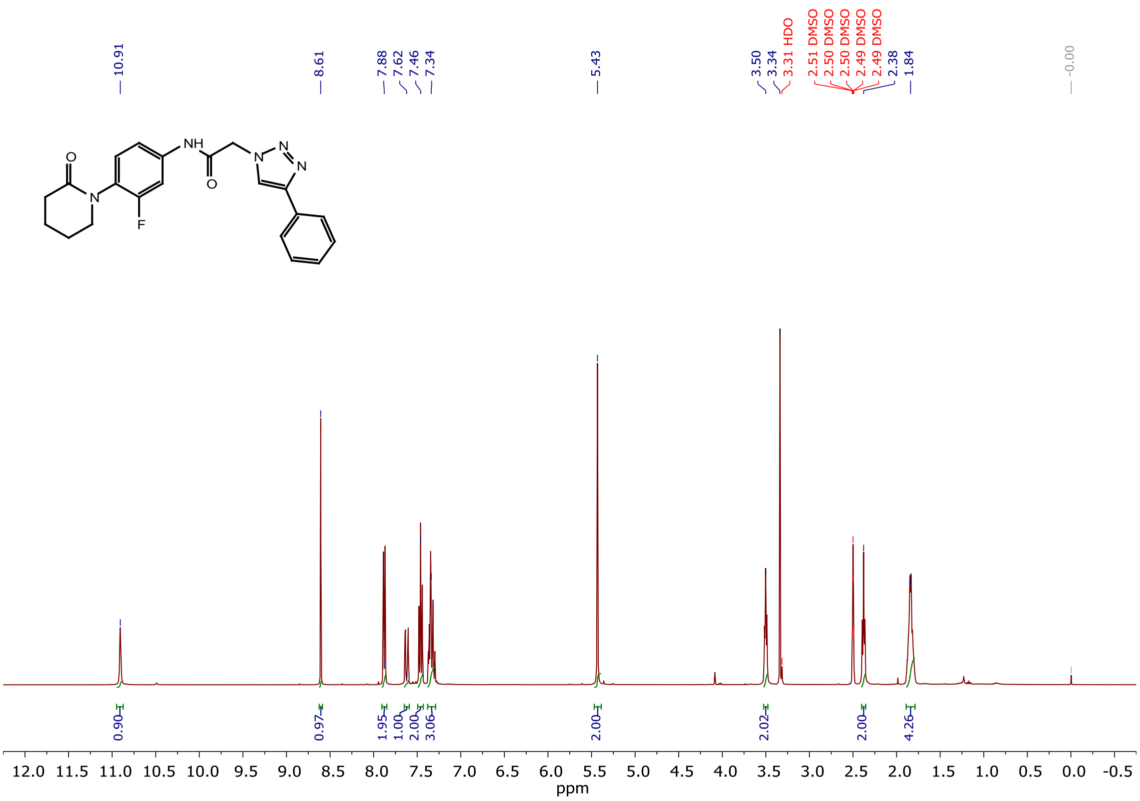

Figure S36. ^{1}H NMR (400 MHz, DMSO-*d*6) of *N*-(3-fluoro-4-(2-oxopiperidin-1-yl)phenyl)-2-(4-phenyl-1*H*-1,2,3-triazol-1-yl)acetamide (**8a**).

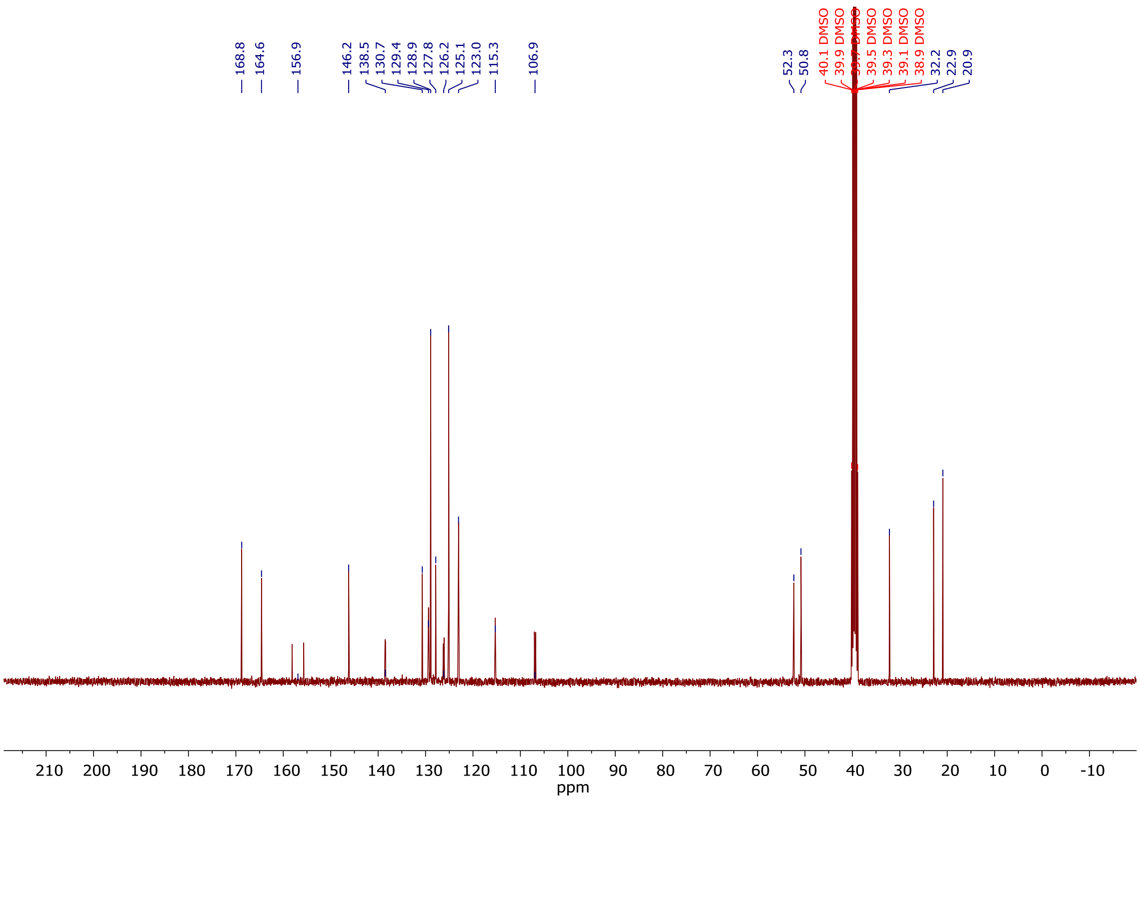

Figure S37. ^{13}C NMR (100 MHz, DMSO-*d*6) of *N*-(3-fluoro-4-(2-oxopiperidin-1-yl)phenyl)-2-(4-phenyl-1*H*-1,2,3-triazol-1-yl)acetamide (**8a**).

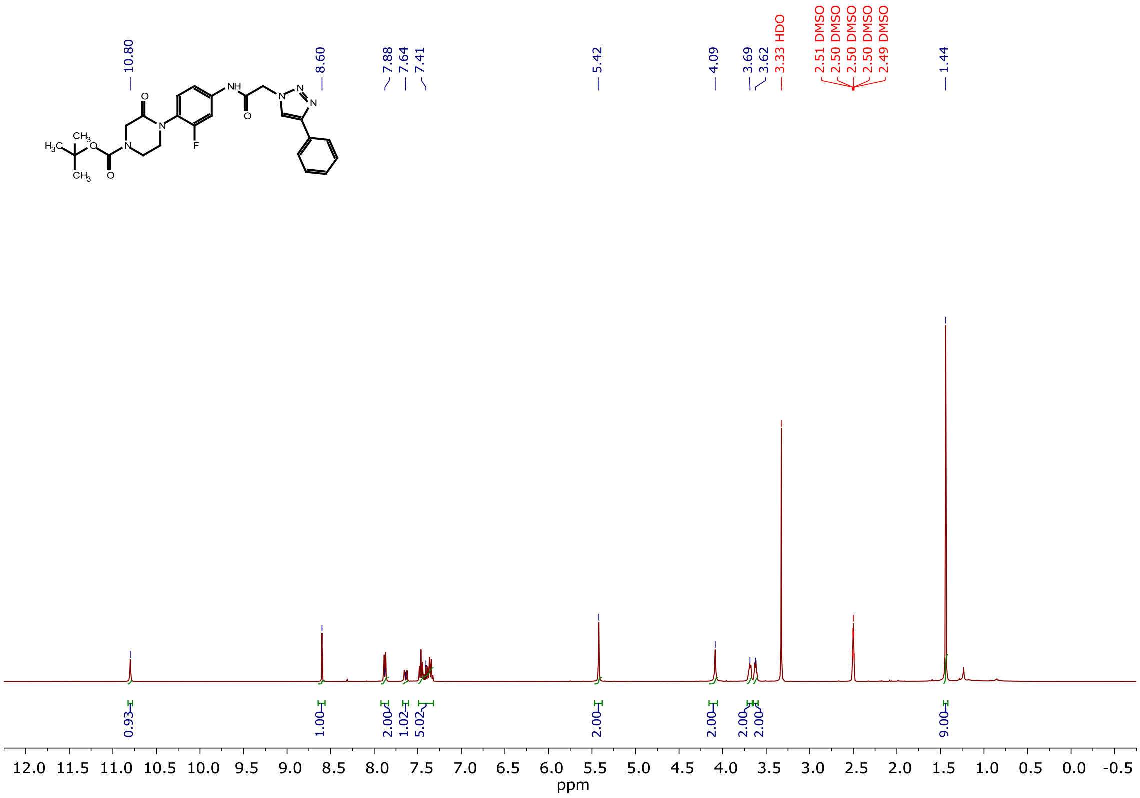

Figure S38. ^{1}H NMR (400 MHz, DMSO-*d*6) of *tert*-butyl 4-(2-fluoro-4-(2-(4-phenyl-1*H*-1,2,3-triazol-1-yl)acetamido)phenyl)-3-oxopiperazine-1-carboxylate (**8b**).

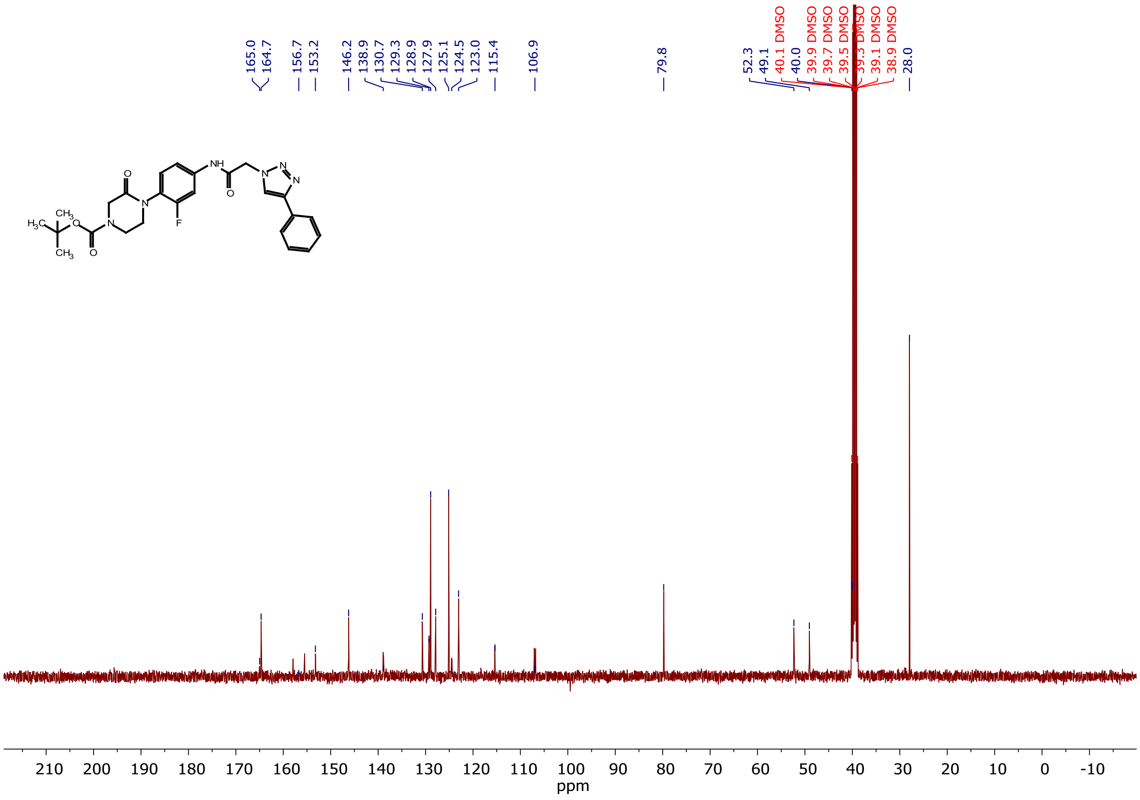

Figure S39. ^{13}C NMR (100 MHz, DMSO-d_6) of *tert*-butyl 4-(2-fluoro-4-(2-(4-phenyl-1*H*-1,2,3-triazol-1-yl)acetamido)phenyl)-3-oxopiperazine-1-carboxylate (**8b**).

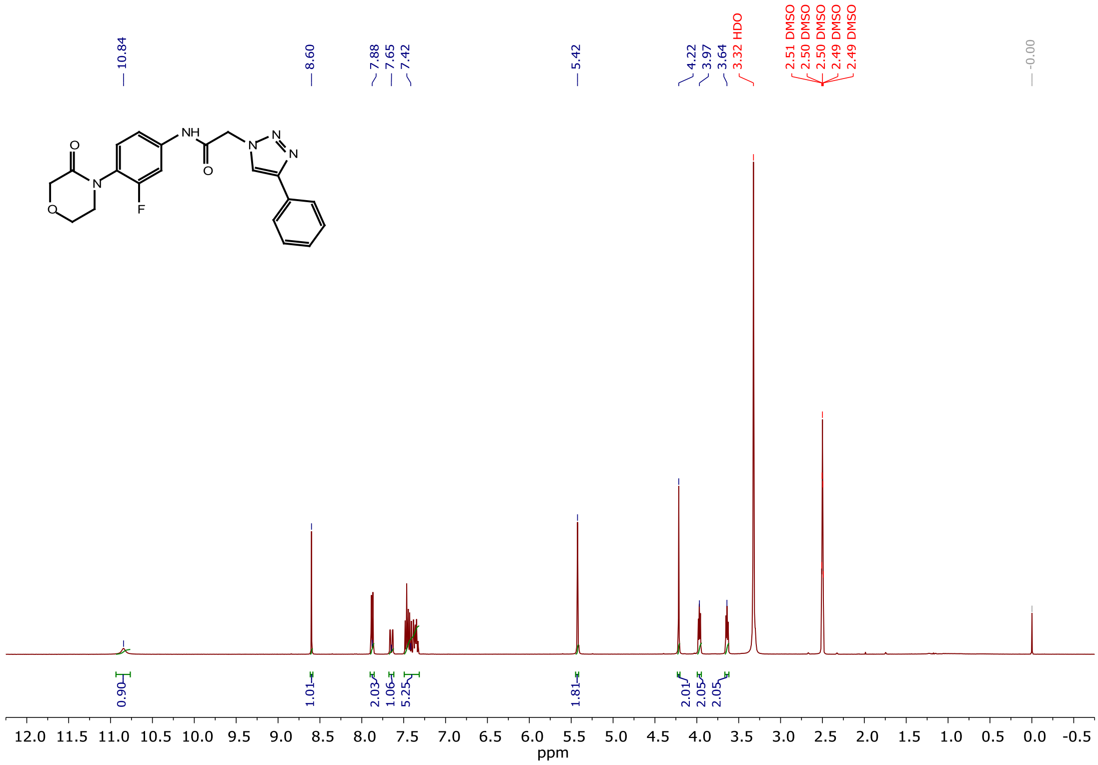

Figure S40. ^{1}H NMR (400 MHz, DMSO-d_6) of *N*-(3-fluoro-4-(3-oxomorpholino)phenyl)-2-(4-phenyl-1*H*-1,2,3-triazol-1-yl)acetamide (**8c**).

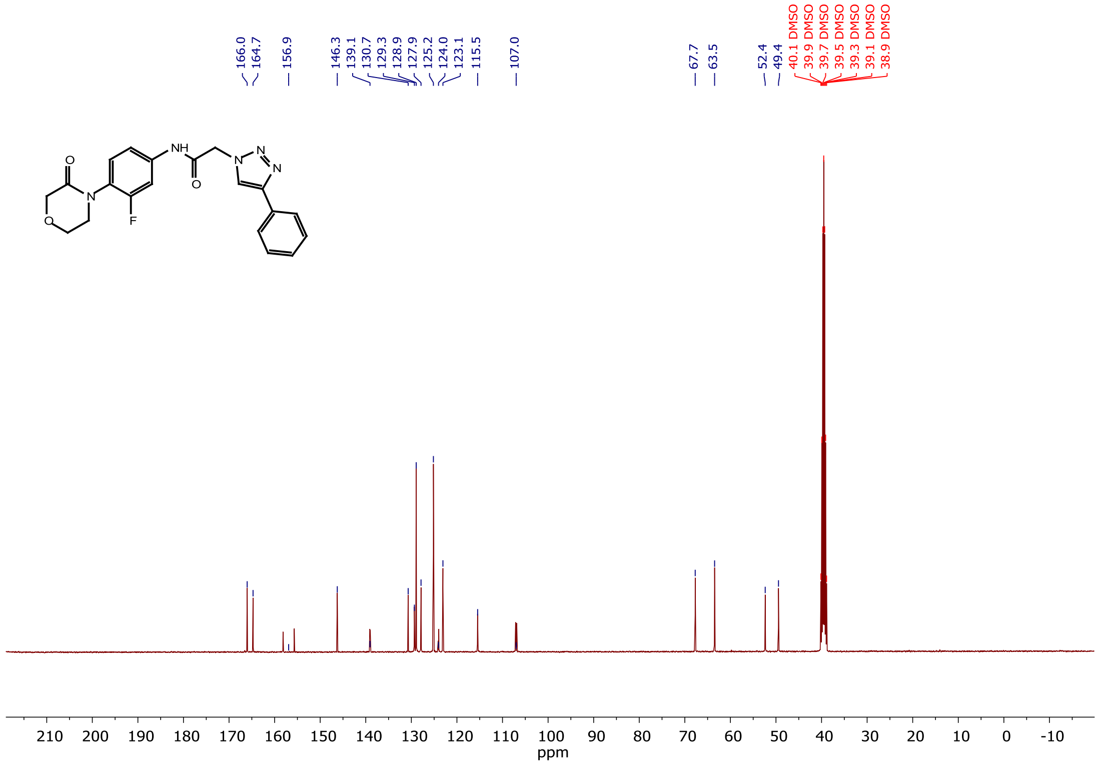

Figure S41. ^{13}C NMR (100 MHz, DMSO-d_6) of *N*-(3-fluoro-4-(3-oxomorpholino)phenyl)-2-(4-phenyl-1*H*-1,2,3-triazol-1-yl)acetamide (**8c**).

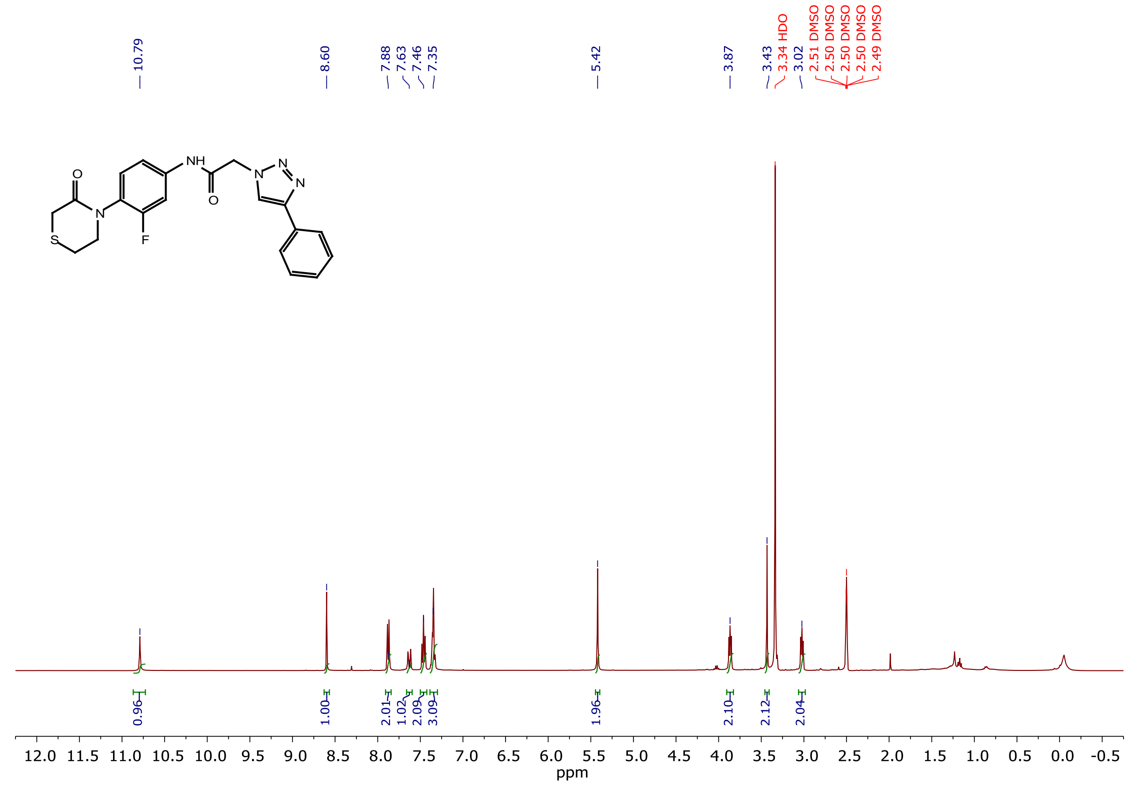

Figure S42. ^{1}H NMR (400 MHz, DMSO-d_6) of *N*-(3-fluoro-4-(3-oxothiomorpholino)phenyl)-2-(4-phenyl-1*H*-1,2,3-triazol-1-yl)acetamide (**8d**).

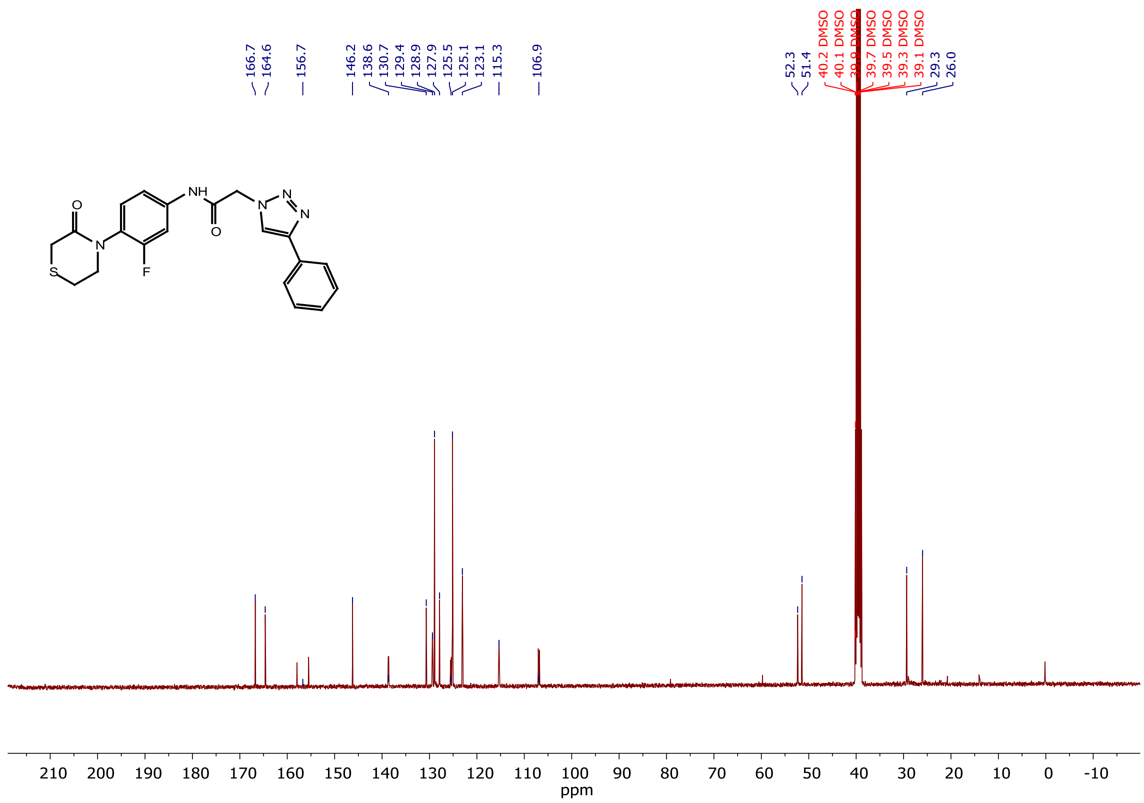

Figure S43. ^{13}C NMR (100 MHz, DMSO-d_6) of *N*-(3-fluoro-4-(3-oxothiomorpholino)phenyl)-2-(4-phenyl-1*H*-1,2,3-triazol-1-yl)acetamide (**8d**).

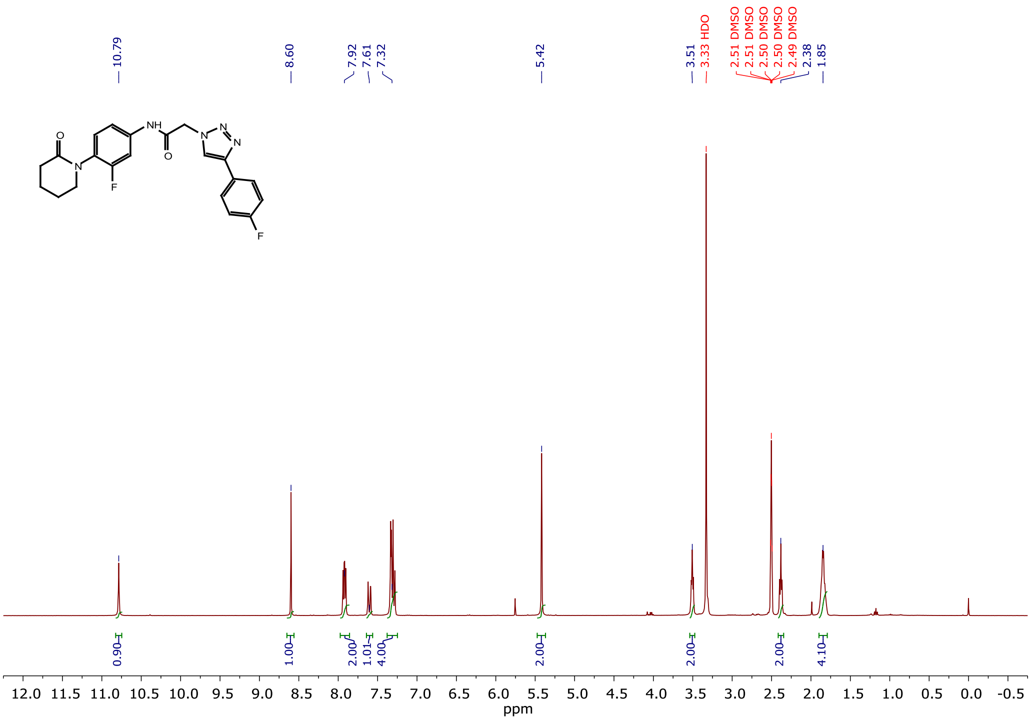

Figure S44. ^{1}H NMR (400 MHz, DMSO-*d*6) of *N*-(3-fluoro-4-(2-oxopiperidin-1-yl)phenyl)-2-(4-(4-fluorophenyl)-1*H*-1,2,3-triazol-1-yl)acetamide (**8e**).

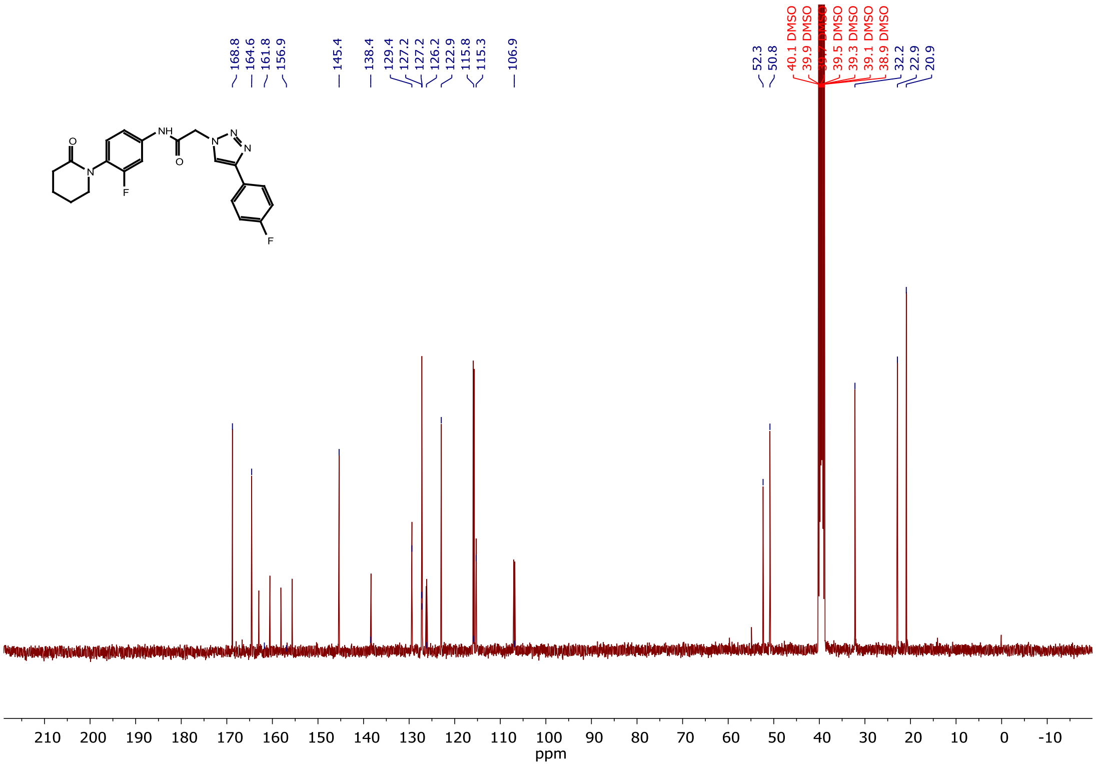

Figure S45. ^{13}C NMR (100 MHz, DMSO-*d*6) of *N*-(3-fluoro-4-(2-oxopiperidin-1-yl)phenyl)-2-(4-(4-fluorophenyl)-1*H*-1,2,3-triazol-1-yl)acetamide (**8e**).

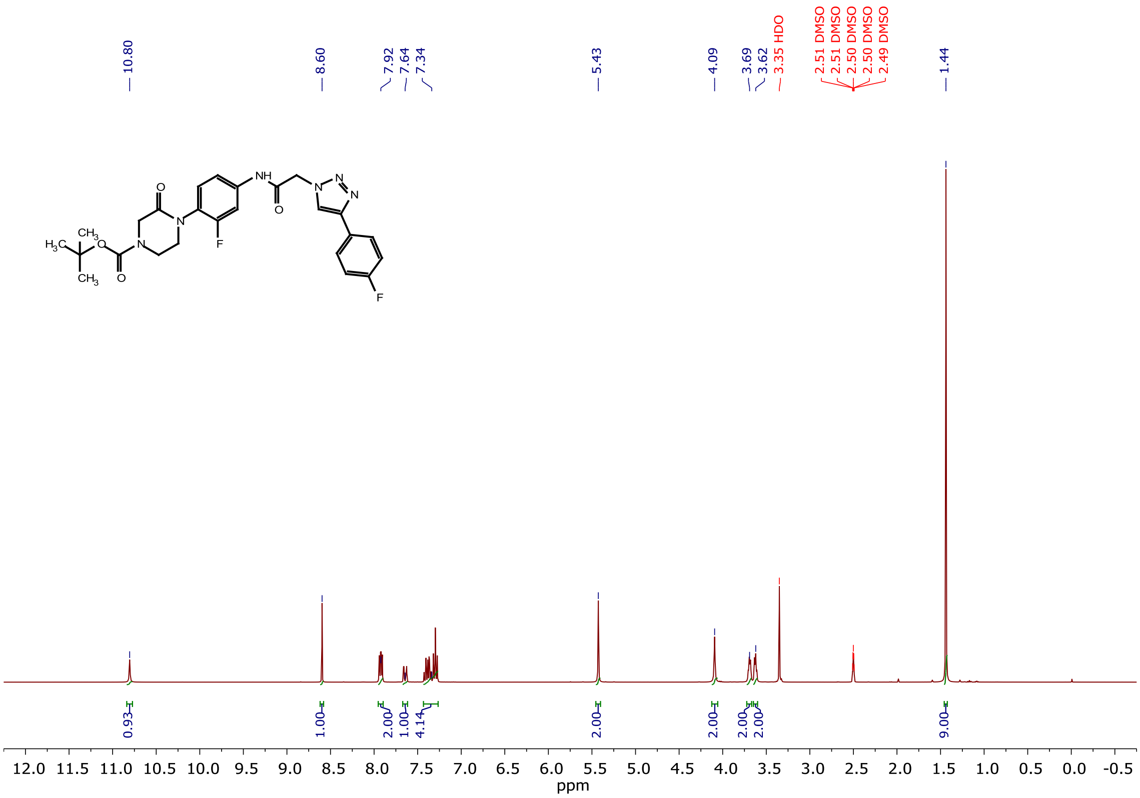

Figure S46. ^{1}H NMR (400 MHz, DMSO-*d*6) of *tert*-butyl 4-(2-fluoro-4-(2-(4-(4-fluorophenyl)-1*H*-1,2,3-triazol-1-yl)acetamido)phenyl)-3-oxopiperazine-1-carboxylate (**8f**).

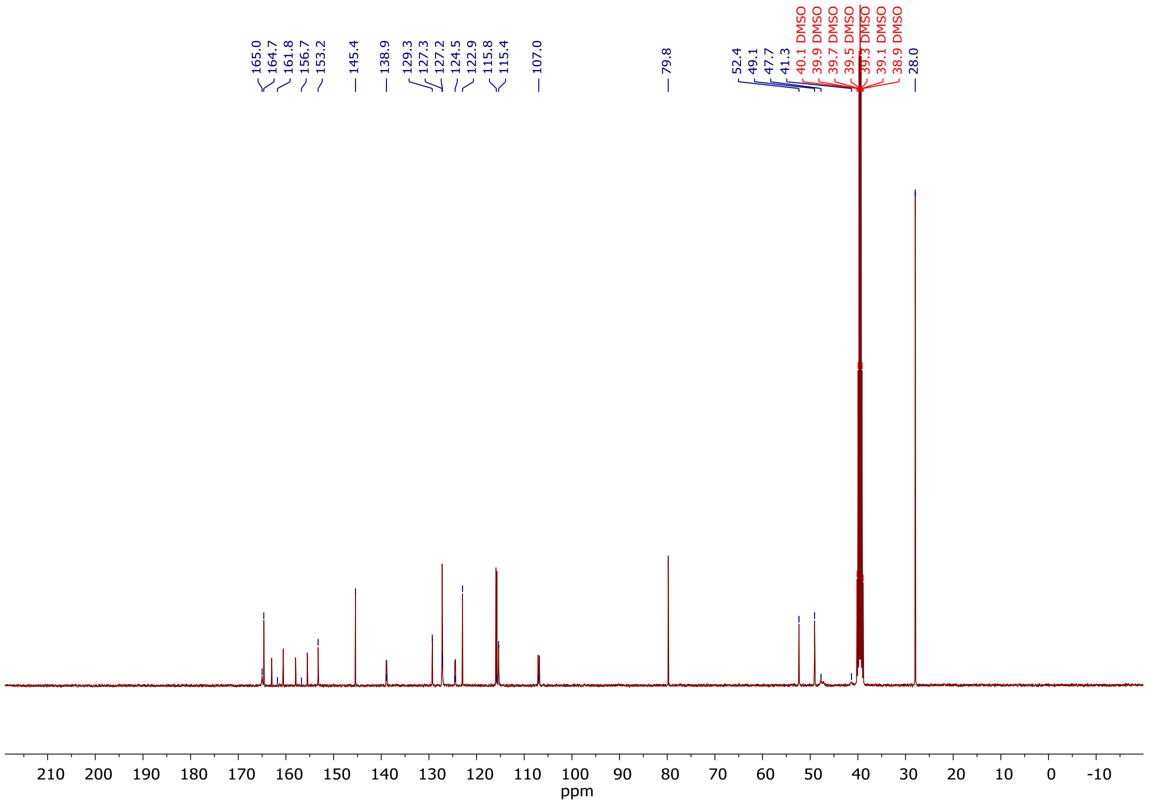

Figure S47. ^{13}C NMR (100 MHz, DMSO-d_6) of *tert*-butyl 4-(2-fluoro-4-(2-(4-(4-fluorophenyl)-1*H*-1,2,3-triazol-1-yl)acetamido)phenyl)-3-oxopiperazine-1-carboxylate (**8f**).

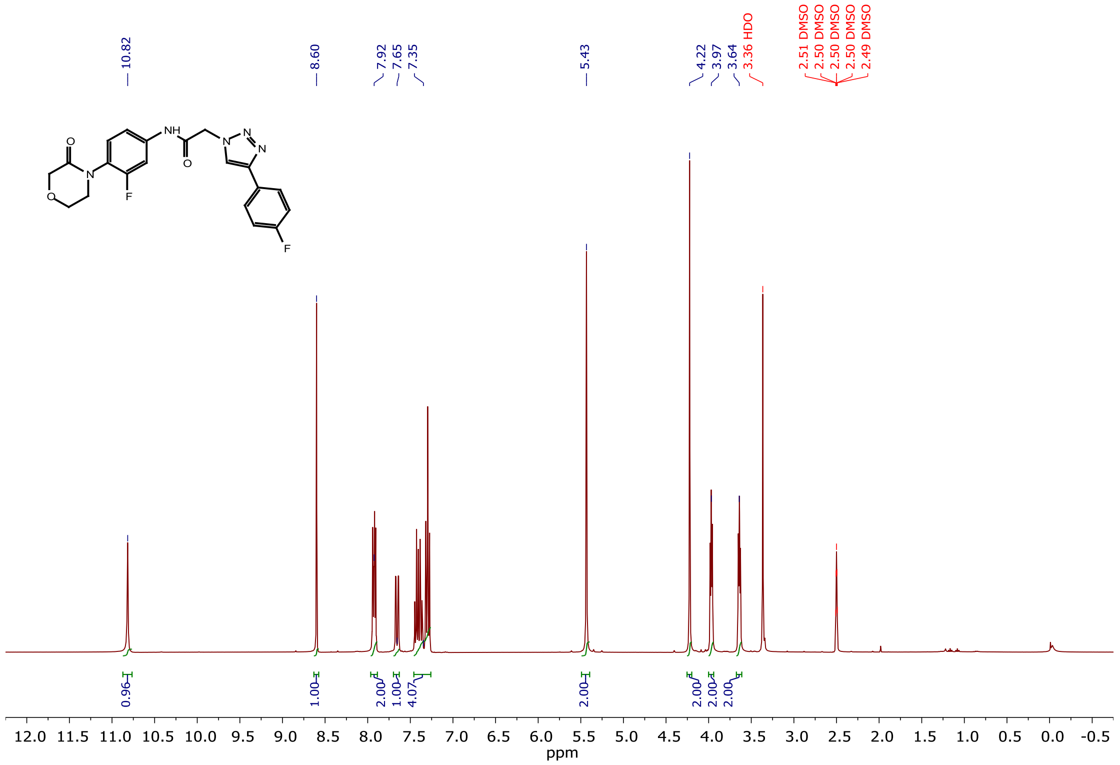

Figure S48. ^{1}H NMR (400 MHz, DMSO-d_6) of *N*-(3-fluoro-4-(3-oxomorpholino)phenyl)-2-(4-(4-fluorophenyl)-1*H*-1,2,3-triazol-1-yl)acetamide (**8g**).

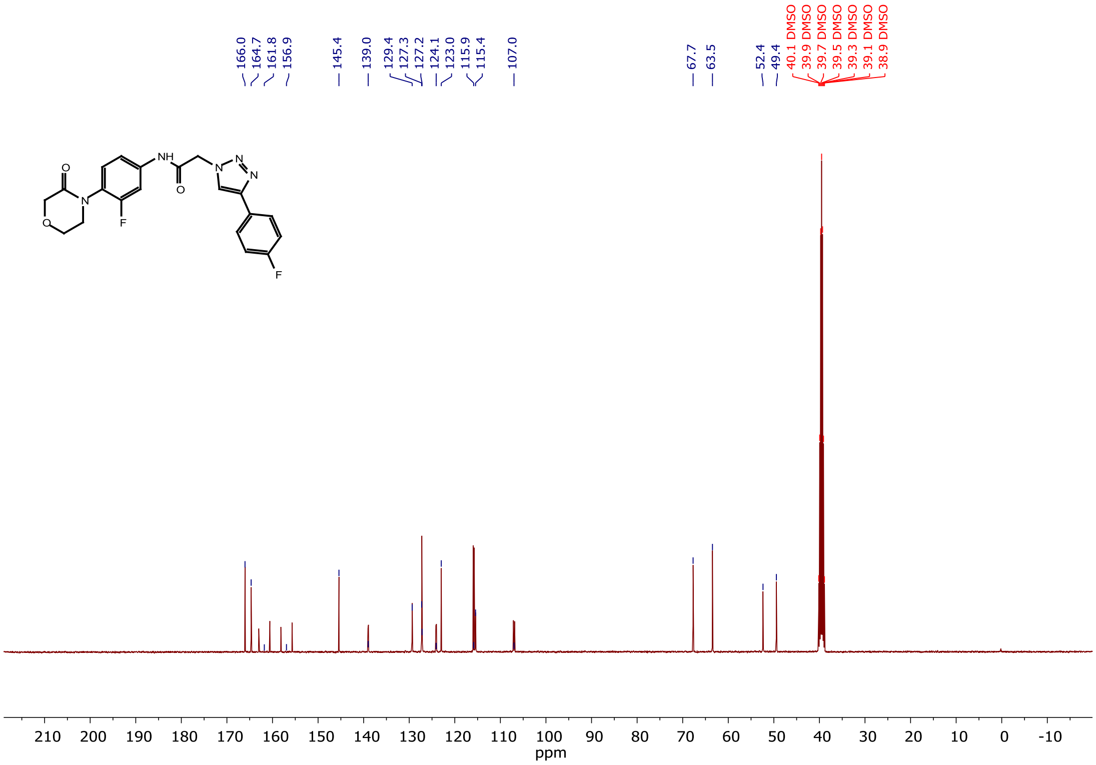

Figure S49. ^{13}C NMR (100 MHz, DMSO-d_6) *N*-(3-fluoro-4-(3-oxomorpholino)phenyl)-2-(4-(4-fluorophenyl)-1*H*-1,2,3-triazol-1-yl)acetamide (**8g**).

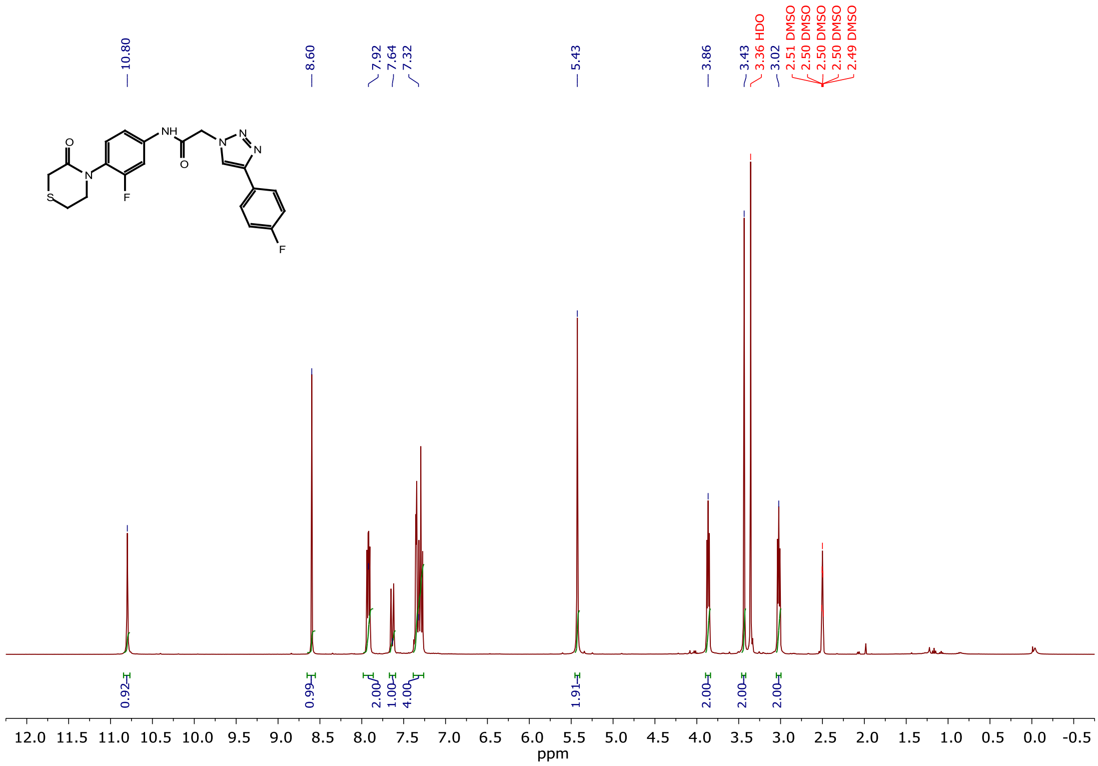

Figure S50. ^{1}H NMR (400 MHz, DMSO-d_6) of *N*-(3-fluoro-4-(3-oxothiomorpholino)phenyl)-2-(4-(4-fluorophenyl)-1*H*-1,2,3-triazol-1-yl)acetamide (**8h**).

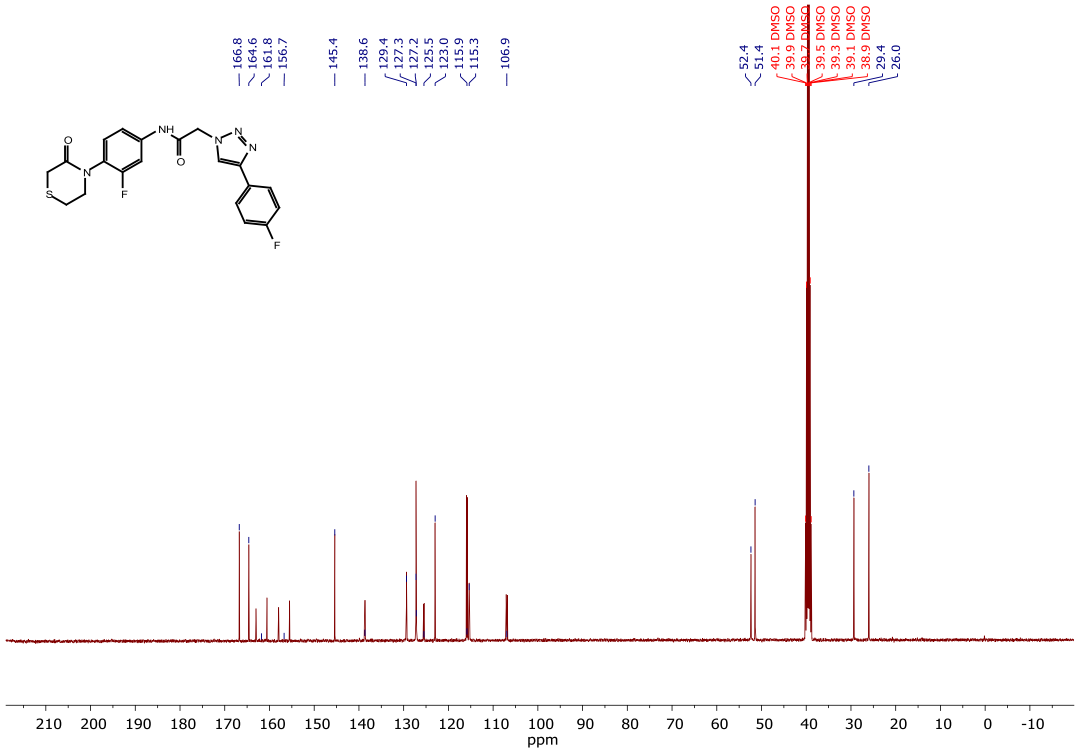

Figure S51. ^{13}C NMR (100 MHz, DMSO-d_6) of *N*-(3-fluoro-4-(3-oxothiomorpholino)phenyl)-2-(4-(4-fluorophenyl)-1*H*-1,2,3-triazol-1-yl)acetamide (**8h**).

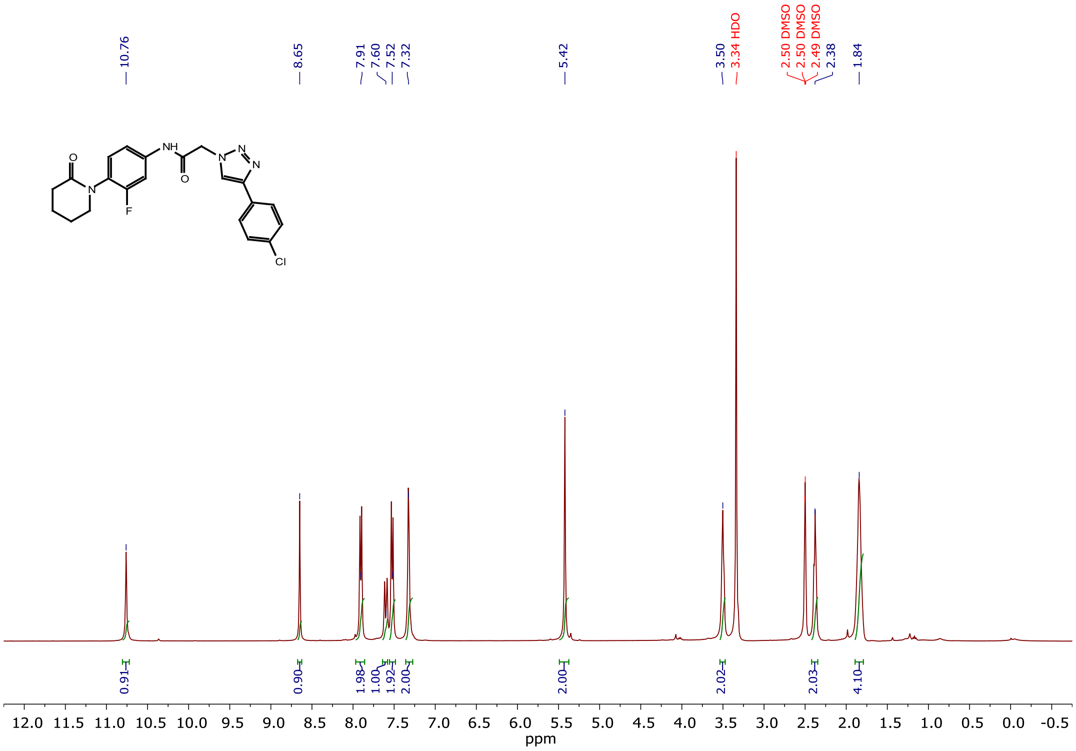

Figure S52. ^{1}H NMR (400 MHz, DMSO-*d*6) of 2-(4-(4-chlorophenyl)-1*H*-1,2,3-triazol-1-yl)-*N*-(3-fluoro-4-(2-oxopiperidin-1-yl)phenyl)acetamide (**8i**).

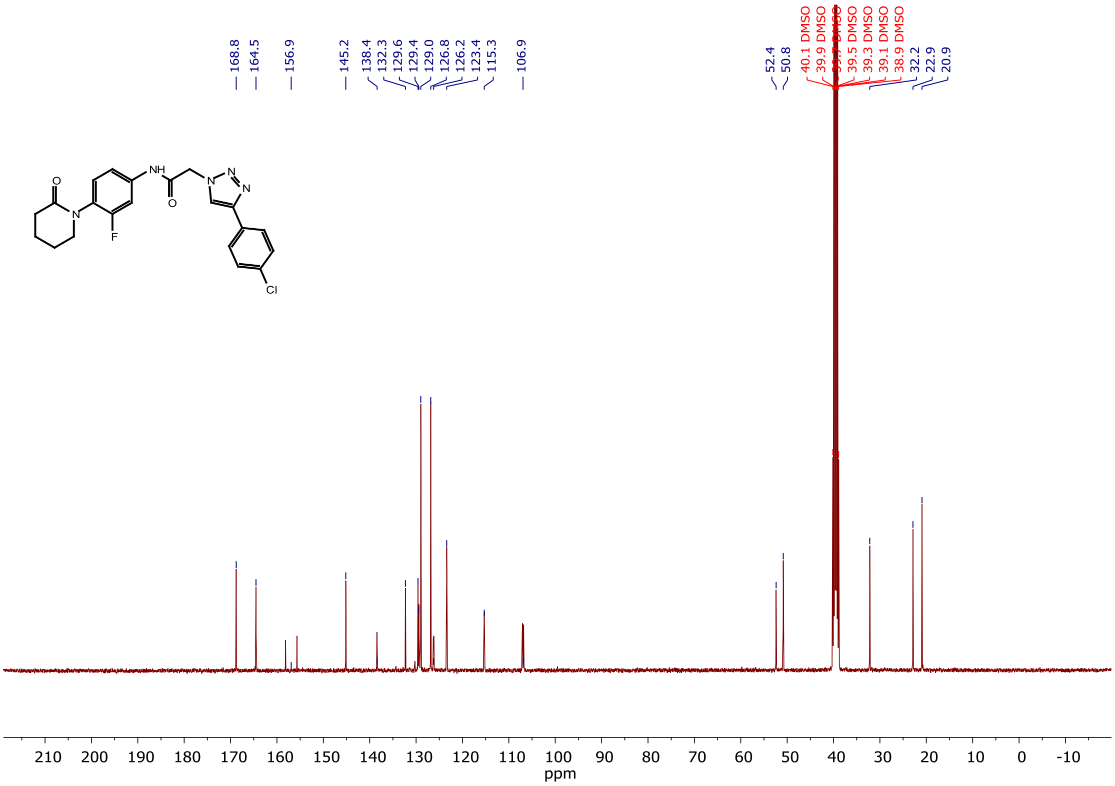

Figure S53. ^{13}C NMR (100 MHz, DMSO-*d*6) of 2-(4-(4-chlorophenyl)-1*H*-1,2,3-triazol-1-yl)-*N*-(3-fluoro-4-(2-oxopiperidin-1-yl)phenyl)acetamide (**8i**).

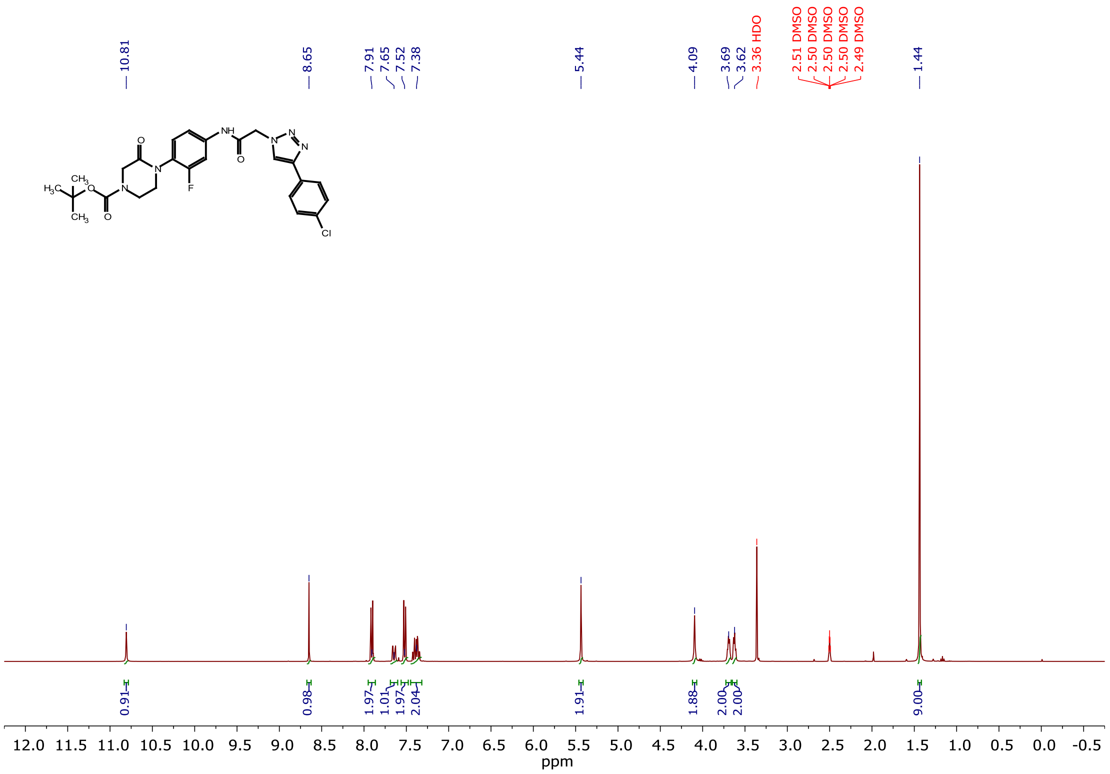

Figure S54. ^{1}H NMR (400 MHz, DMSO-*d*$_6$) of *tert*-butyl 4-(4-(2-(4-(4-chlorophenyl)-1*H*-1,2,3-triazol-1-yl)acetamido)-2-fluorophenyl)-3-oxopiperazine-1-carboxylate (**8j**).

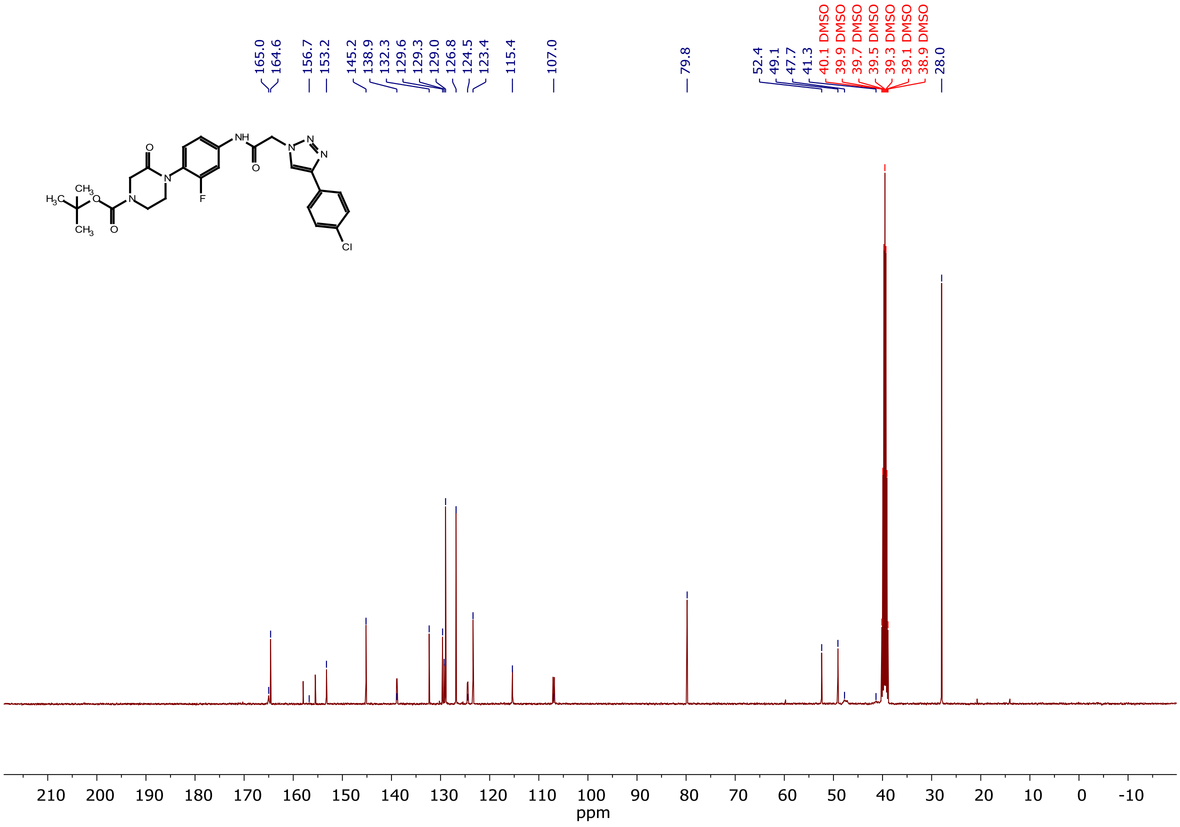

Figure S55. ^{13}C NMR (100 MHz, DMSO-d_6) of *tert*-butyl 4-(4-(2-(4-(4-chlorophenyl)-1*H*-1,2,3-triazol-1-yl)acetamido)-2-fluorophenyl)-3-oxopiperazine-1-carboxylate (**8j**).

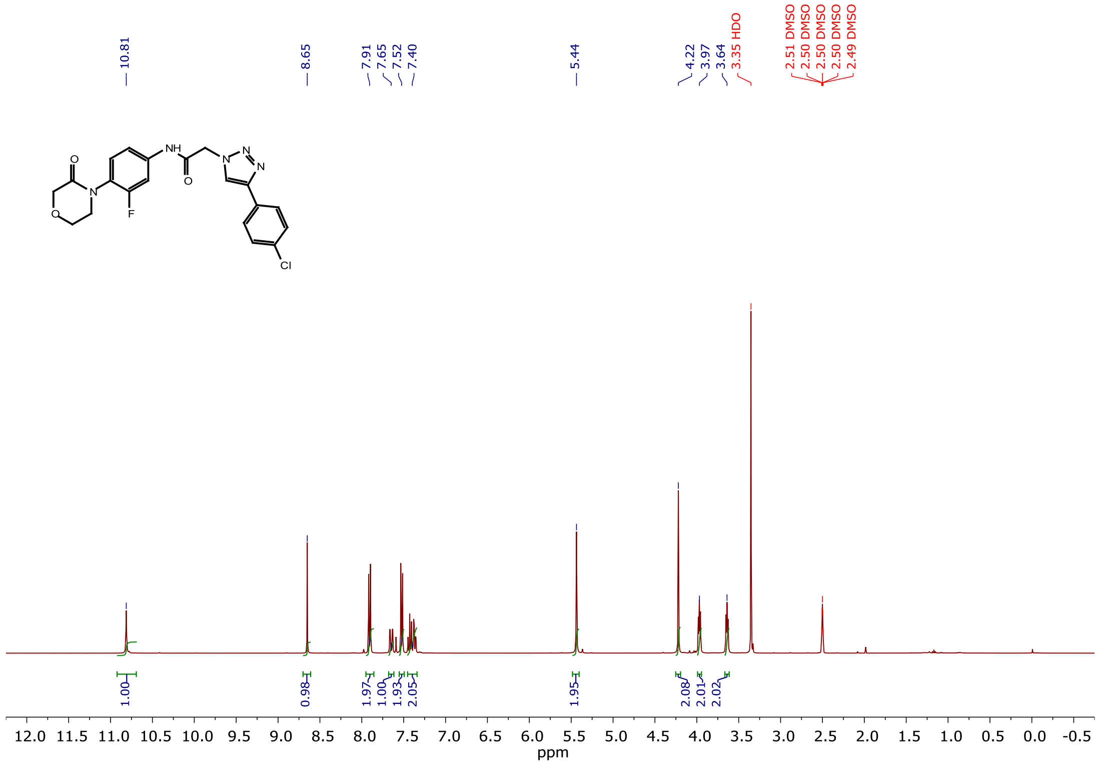

Figure S56. ^{1}H NMR (400 MHz, DMSO-d_6) of 2-(4-(4-chlorophenyl)-1*H*-1,2,3-triazol-1-yl)-*N*-(3-fluoro-4-(3-oxomorpholino)phenyl)acetamide (**8k**).

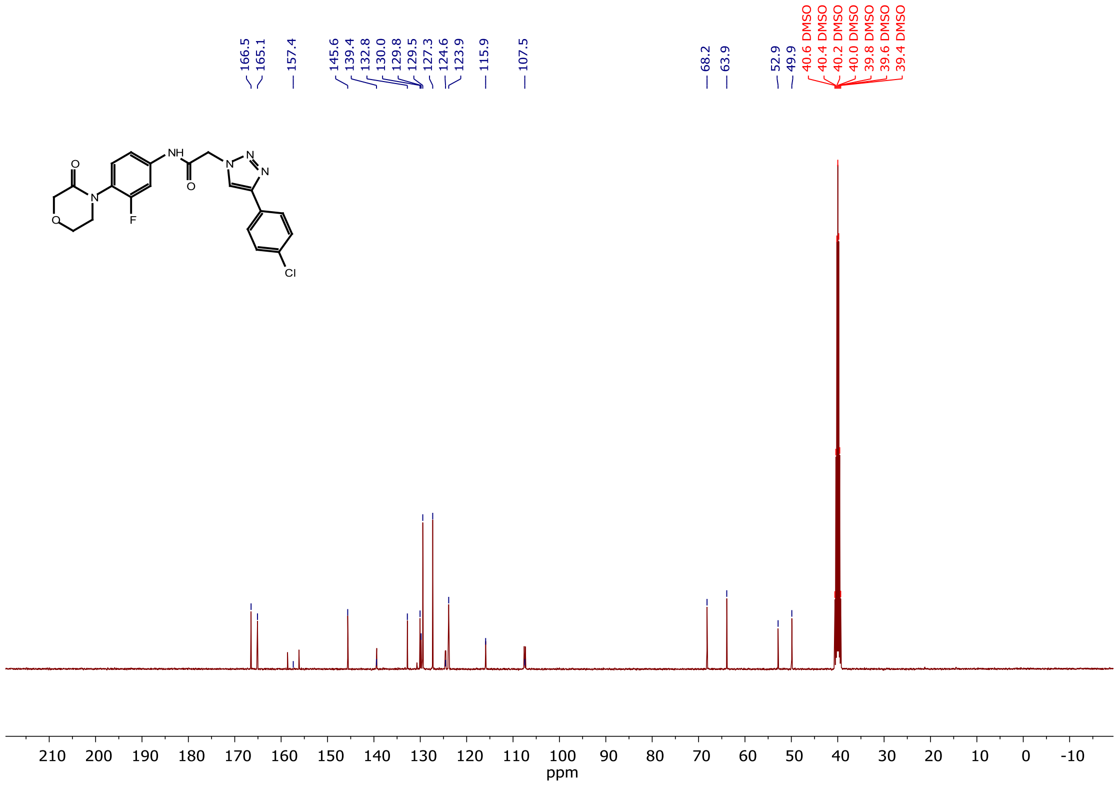

Figure S57. ^{13}C NMR (100 MHz, DMSO-*d*6) of 2-(4-(4-chlorophenyl)-1*H*-1,2,3-triazol-1-yl)-*N*-(3-fluoro-4-(3-oxomorpholino)phenyl)acetamide (**8k**).

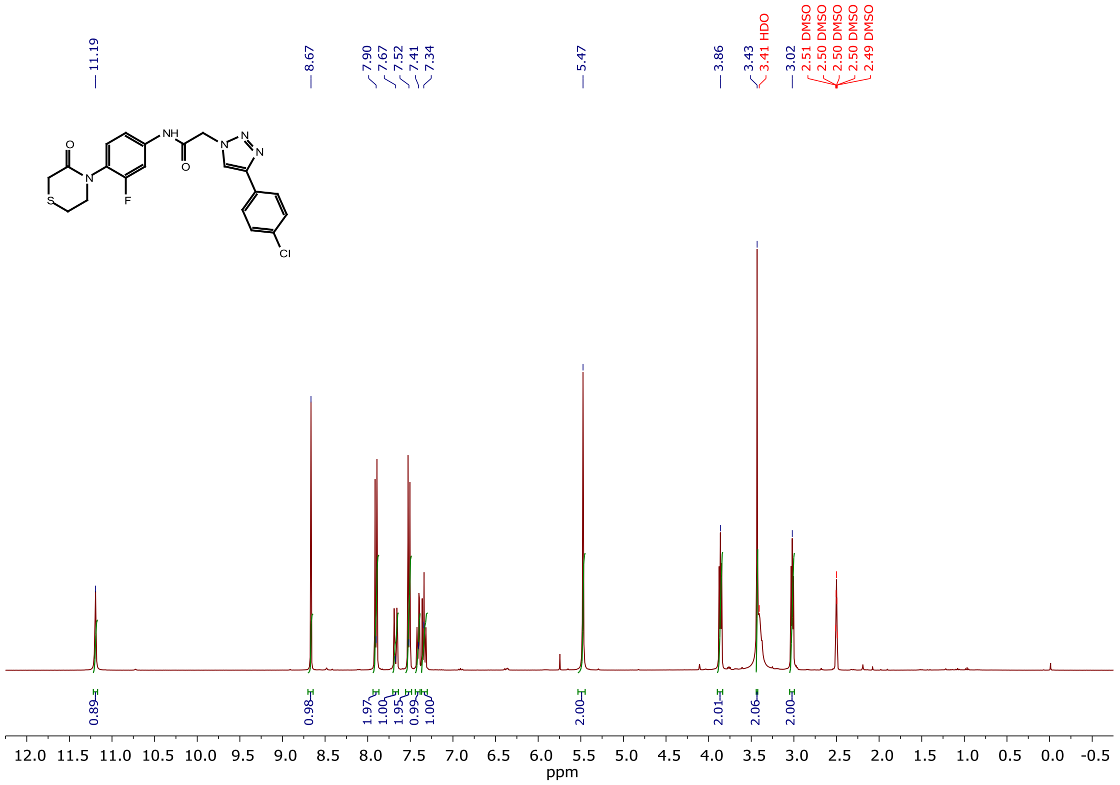

Figure S58. ^{1}H NMR (400 MHz, DMSO-*d*6) of 2-(4-(4-chlorophenyl)-1*H*-1,2,3-triazol-1-yl)-*N*-(3-fluoro-4-(3-oxothiomorpholino)phenyl)acetamide (**8l**).

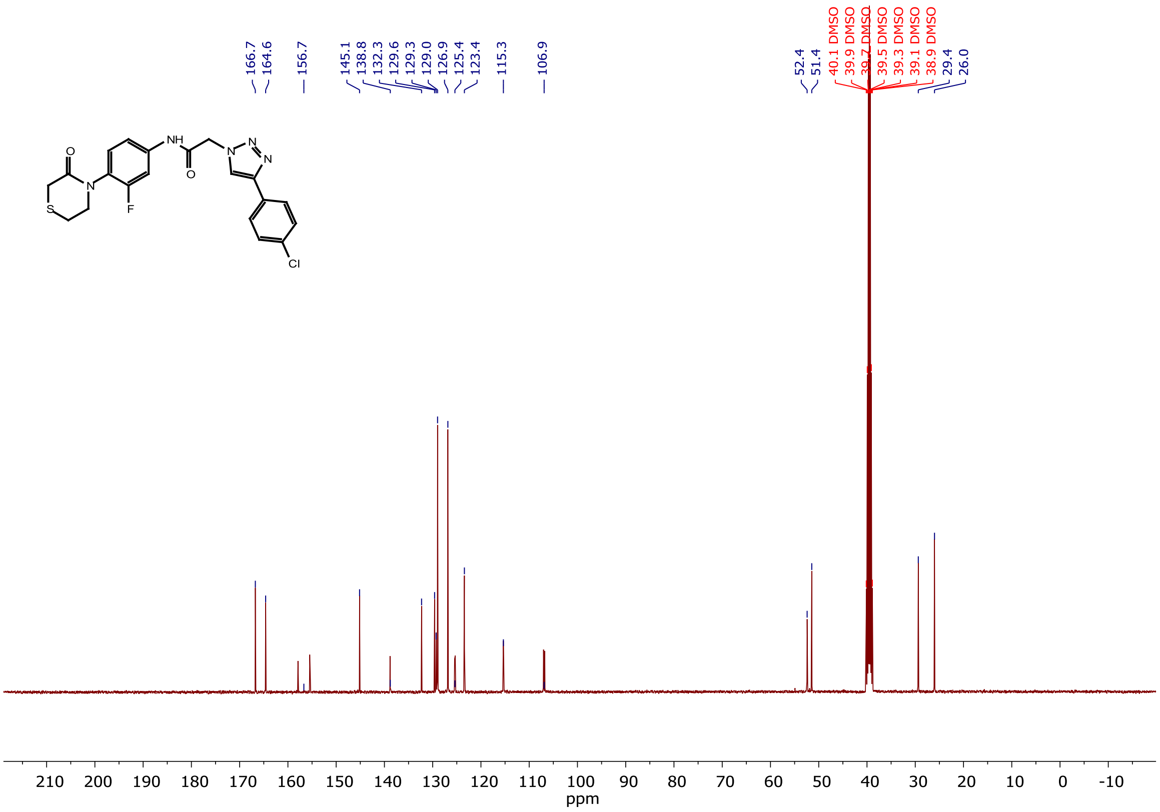

Figure S59. ^{13}C NMR (100 MHz, DMSO-d_6) of 2-(4-(4-chlorophenyl)-1*H*-1,2,3-triazol-1-yl)-*N*-(3-fluoro-4-(3-oxothiomorpholino)phenyl)acetamide (**8l**).

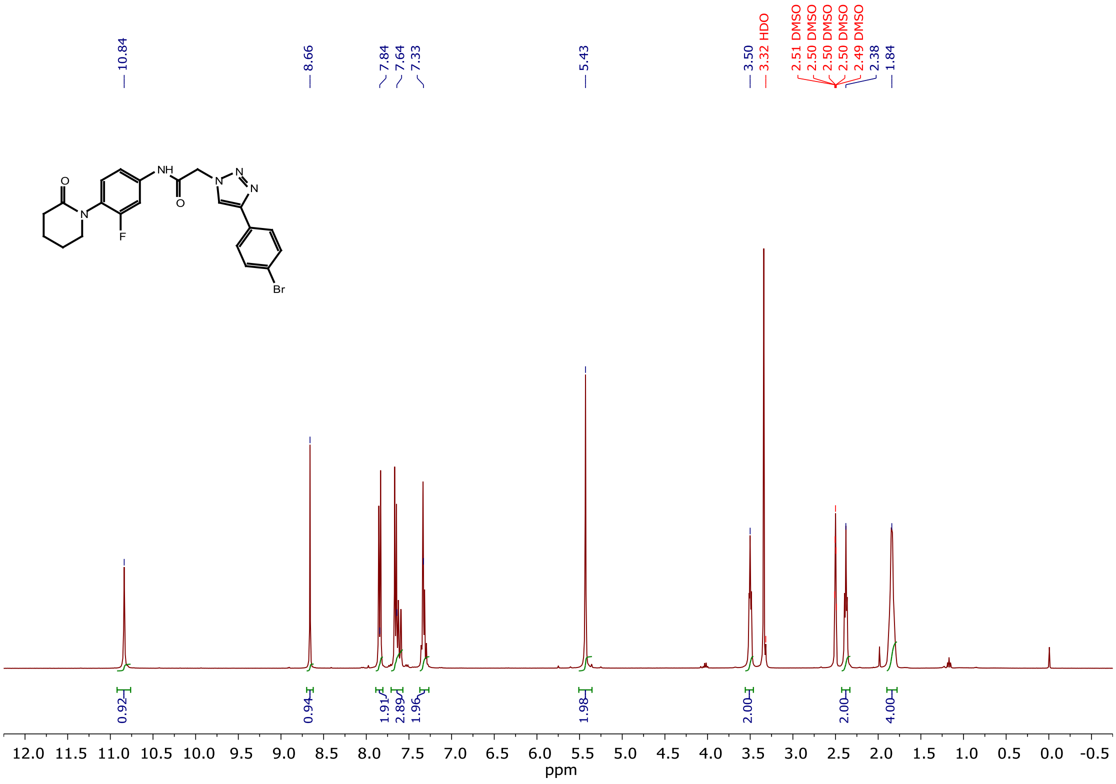

Figure S60. ^{1}H NMR (400 MHz, DMSO-d_6) of 2-(4-(4-bromophenyl)-1*H*-1,2,3-triazol-1-yl)-*N*-(3-fluoro-4-(2-oxopiperidin-1-yl)phenyl)acetamide (**8m**).

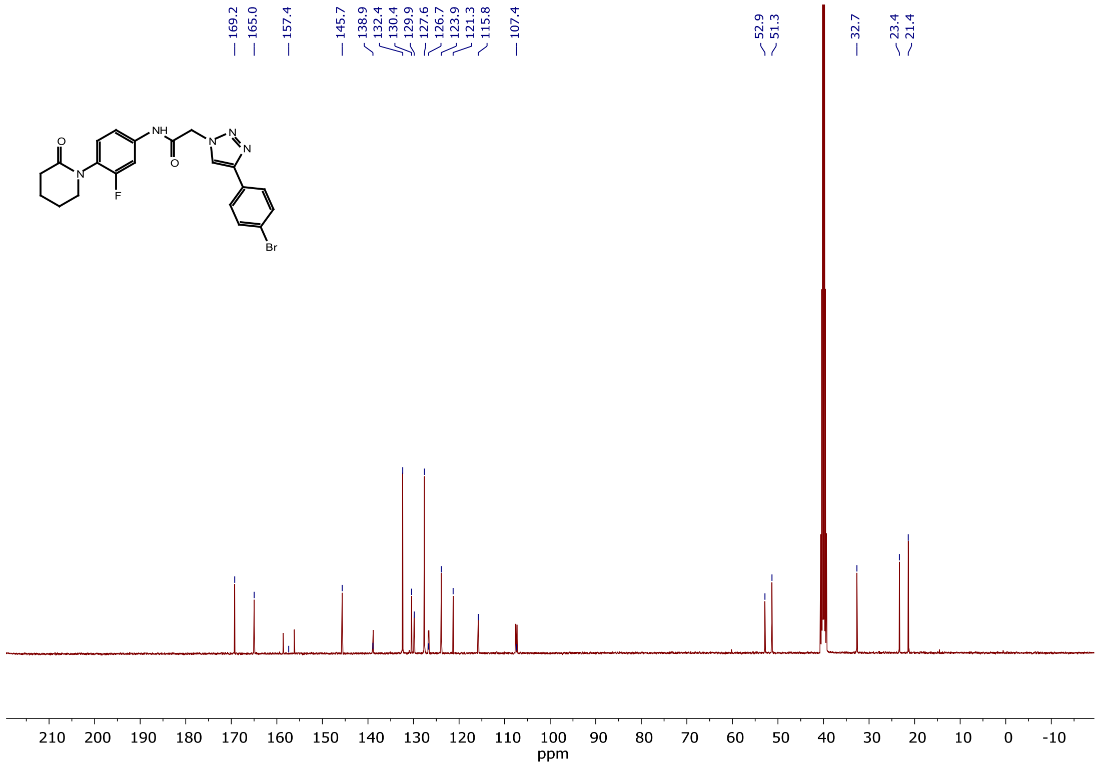

Figure S61. ^{13}C NMR (100 MHz, DMSO-d_6) of 2-(4-(4-bromophenyl)-1*H*-1,2,3-triazol-1-yl)-*N*-(3-fluoro-4-(2-oxopiperidin-1-yl)phenyl)acetamide (**8m**).

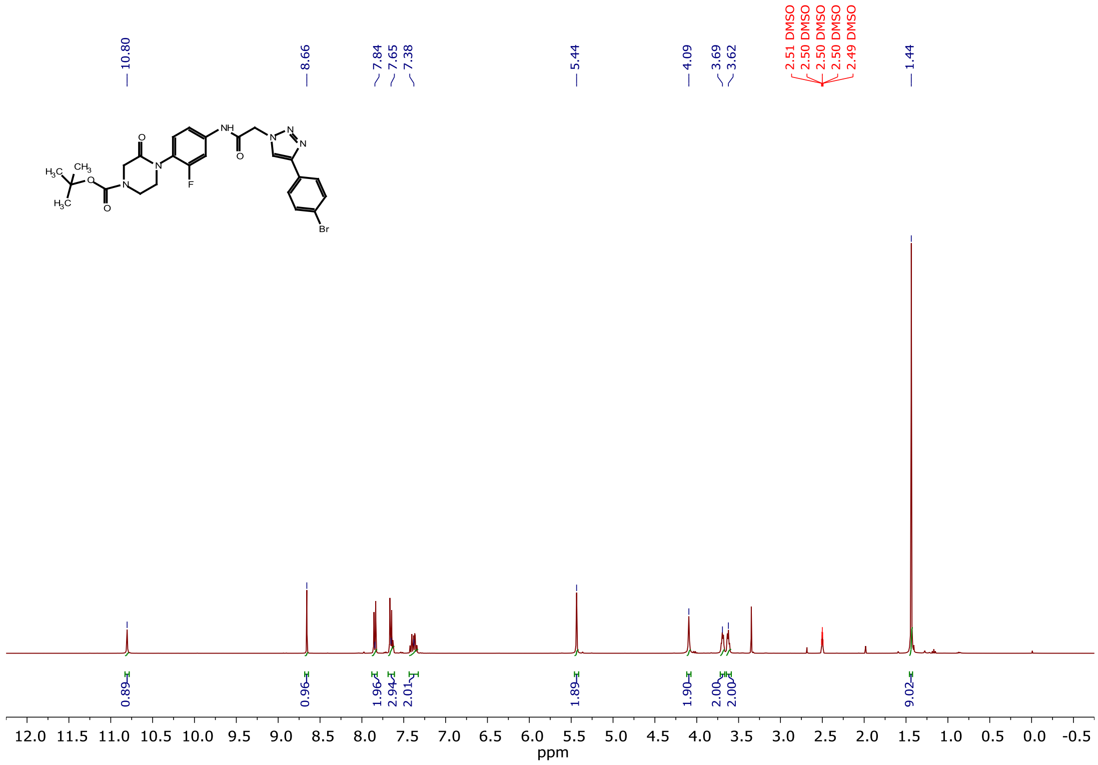

Figure S62. ^{1}H NMR (400 MHz, DMSO-d_6) of *tert*-butyl 4-(4-(2-(4-(4-bromophenyl)-1*H*-1,2,3-triazol-1-yl)acetamido)-2-fluorophenyl)-3-oxopiperazine-1-carboxylate (**8n**).

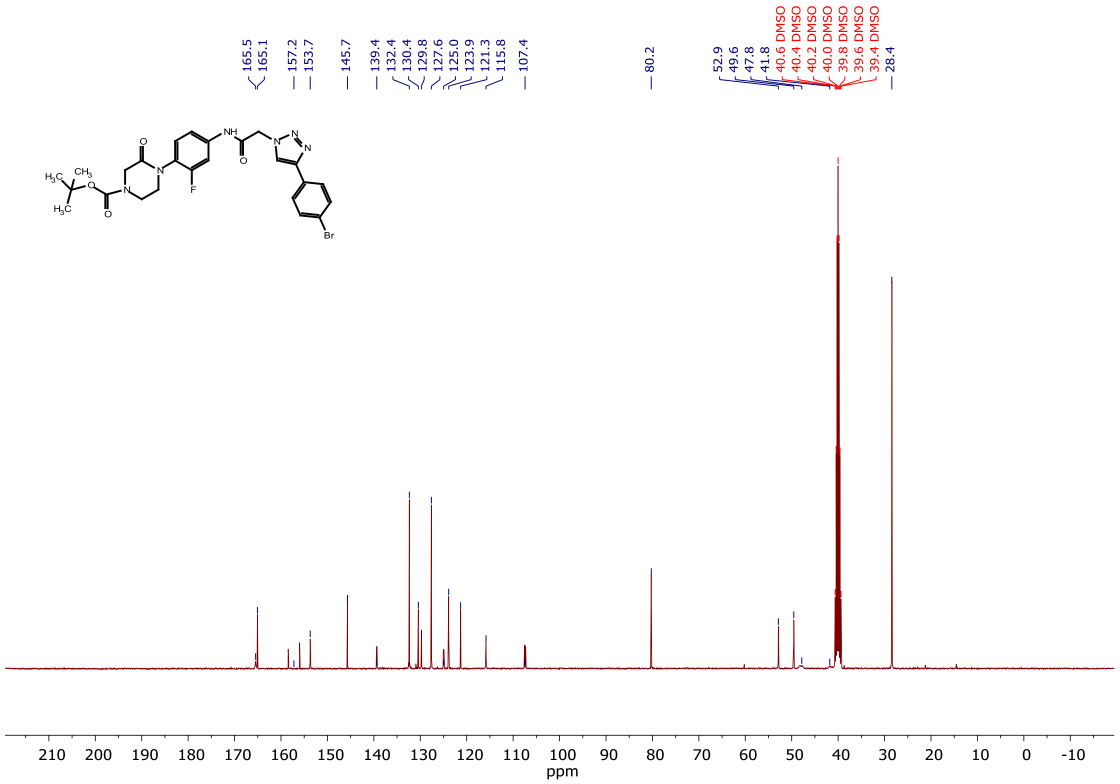

Figure S63. ^{13}C NMR (100 MHz, DMSO-d_6) of *tert*-butyl 4-(4-(2-(4-(4-bromophenyl)-1*H*-1,2,3-triazol-1-yl)acetamido)-2-fluorophenyl)-3-oxopiperazine-1-carboxylate (**8n**).

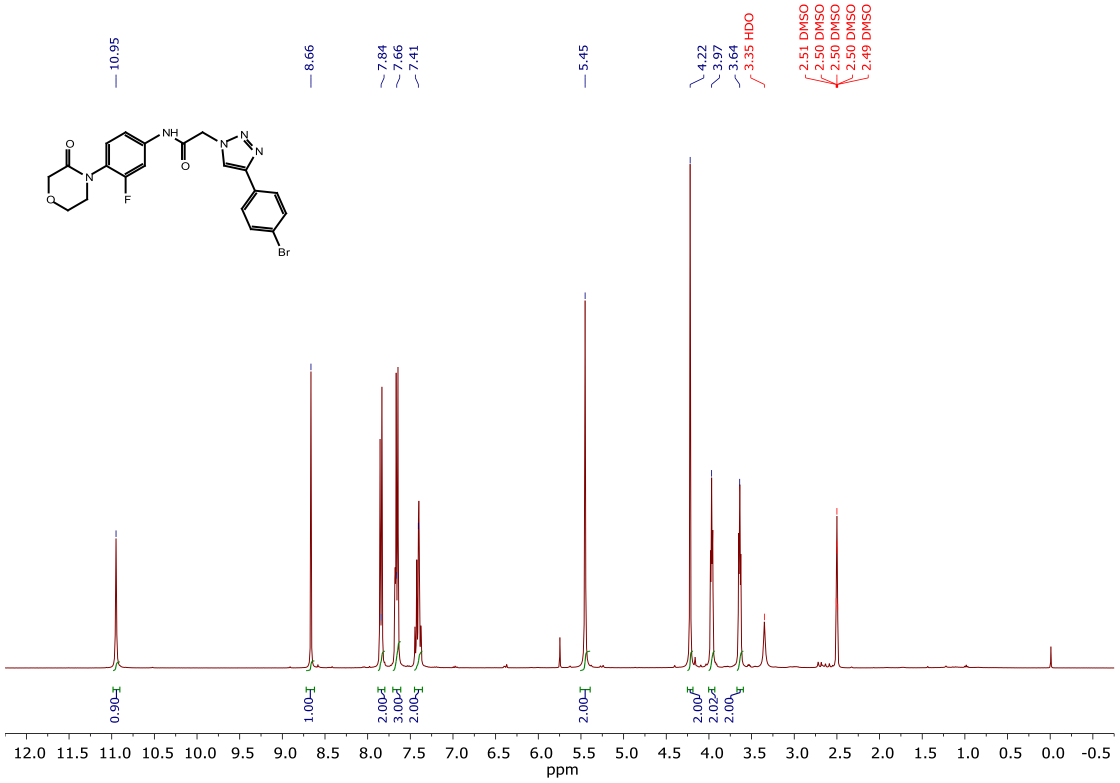

Figure S64. ^{1}H NMR (400 MHz, DMSO-d_6) of 2-(4-(4-bromophenyl)-1*H*-1,2,3-triazol-1-yl)-*N*-(3-fluoro-4-(3-oxomorpholino)phenyl)acetamide (**8o**).

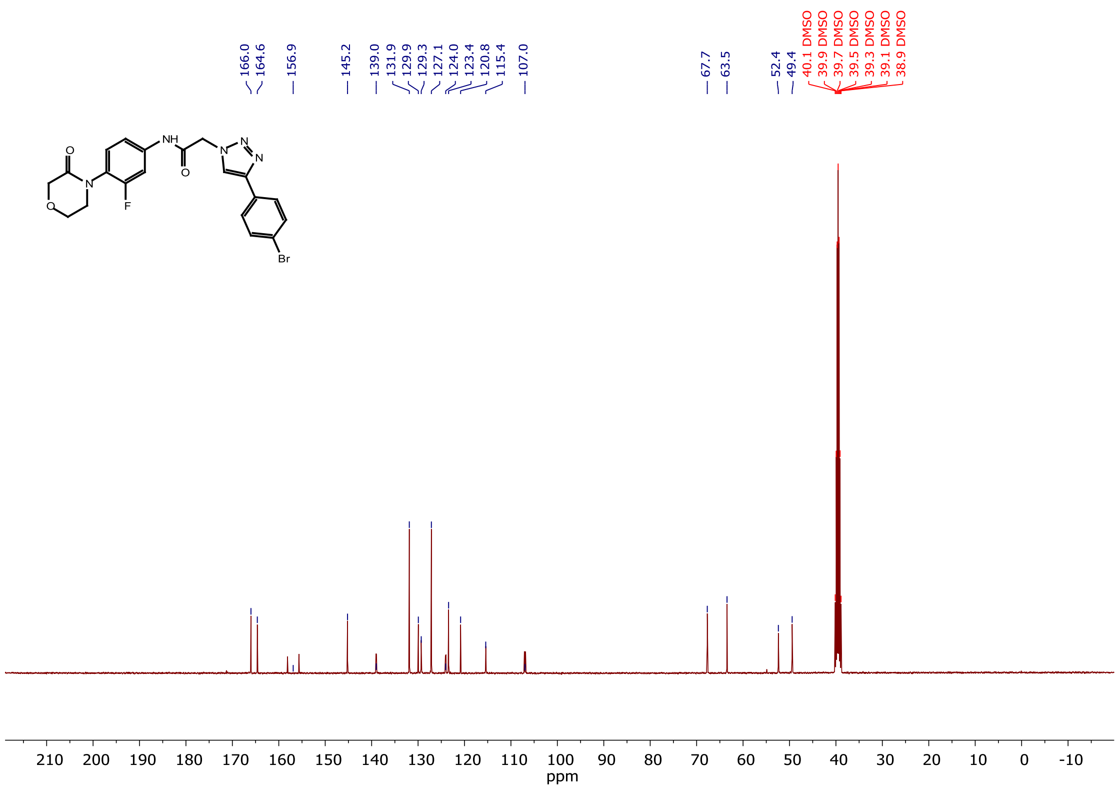

Figure S65. ^{13}C NMR (100 MHz, DMSO-d_6) of 2-(4-(4-bromophenyl)-1*H*-1,2,3-triazol-1-yl)-*N*-(3-fluoro-4-(3-oxomorpholino)phenyl)acetamide (**8o**).

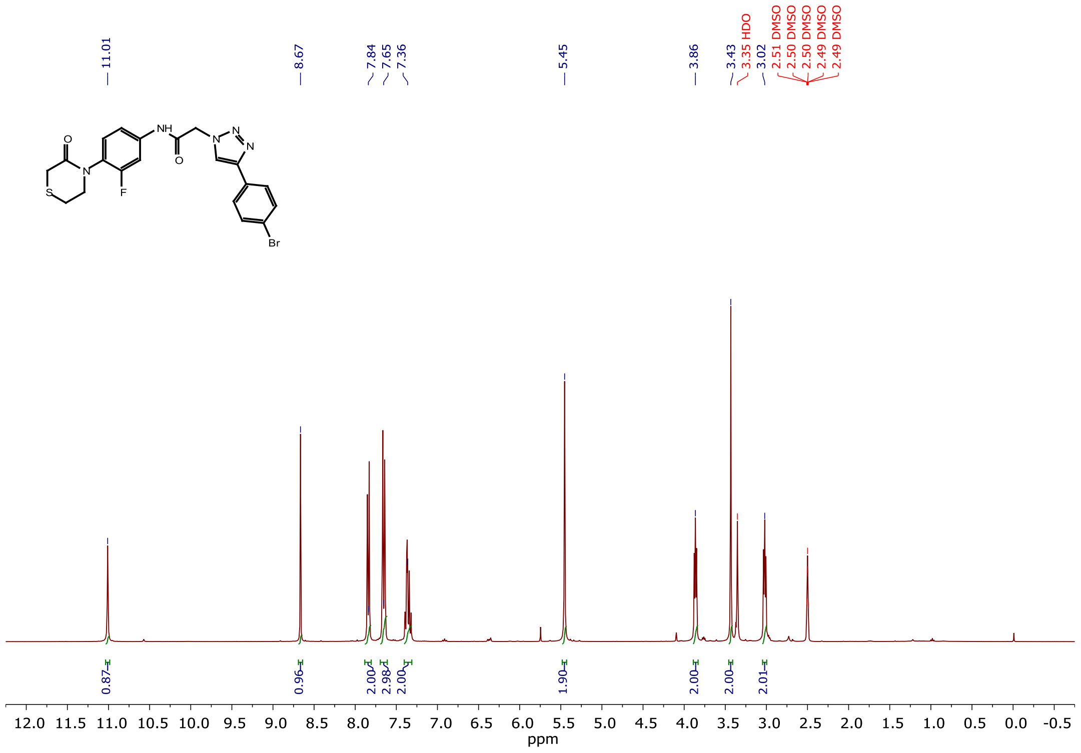

Figure S66. ^{1}H NMR (400 MHz, DMSO-d_6) 2-(4-(4-bromophenyl)-1*H*-1,2,3-triazol-1-yl)-*N*-(3-fluoro-4-(3-oxothiomorpholino)phenyl)acetamide (**8p**).

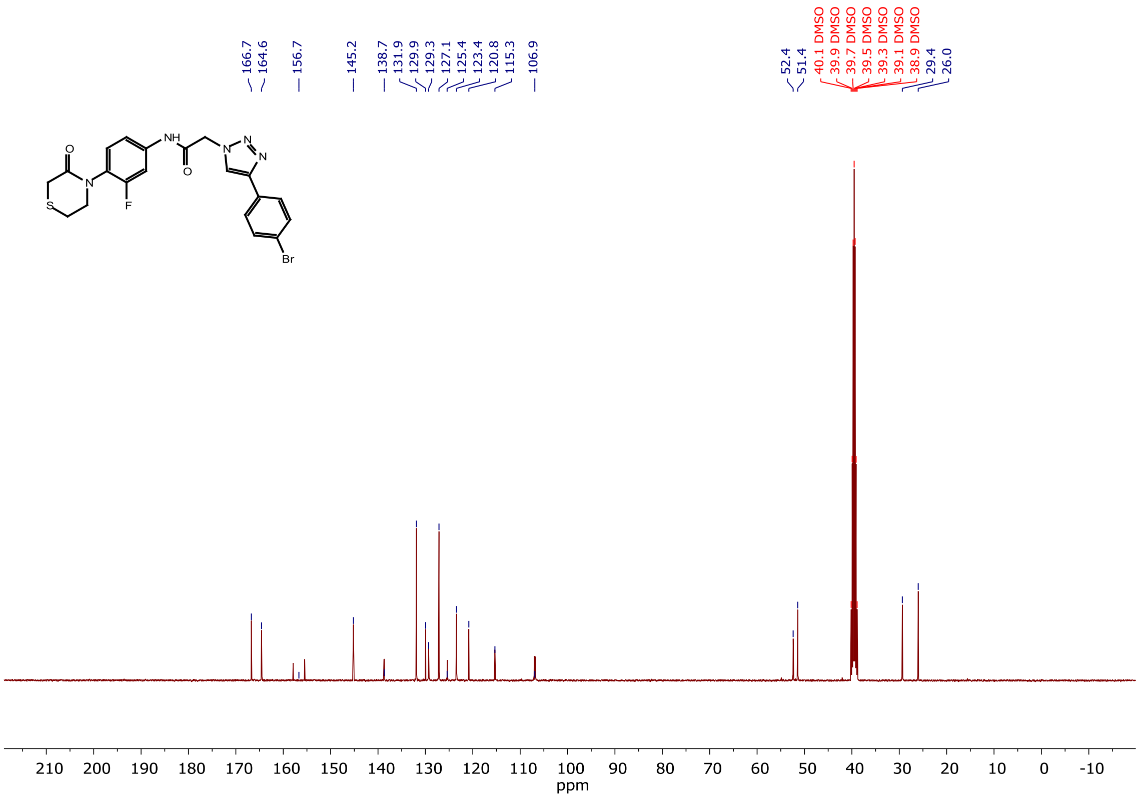

Figure S67. ^{13}C NMR (100 MHz, DMSO-d_6) of 2-(4-(4-bromophenyl)-1*H*-1,2,3-triazol-1-yl)-*N*-(3-fluoro-4-(3-oxothiomorpholino)phenyl)acetamide (**8p**).

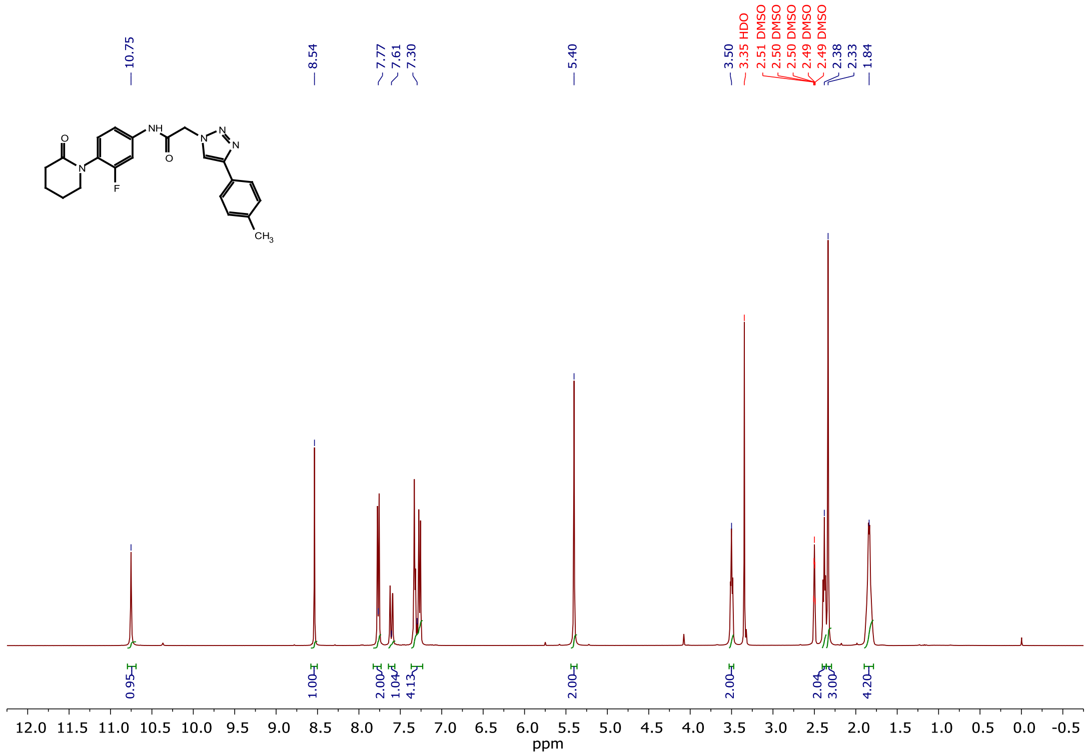

Figure S68. ^{1}H NMR (400 MHz, DMSO-d_6) of *N*-(3-fluoro-4-(2-oxopiperidin-1-yl)phenyl)-2-(4-(*p*-tolyl)-1*H*-1,2,3-triazol-1-yl)acetamide(**8q**).

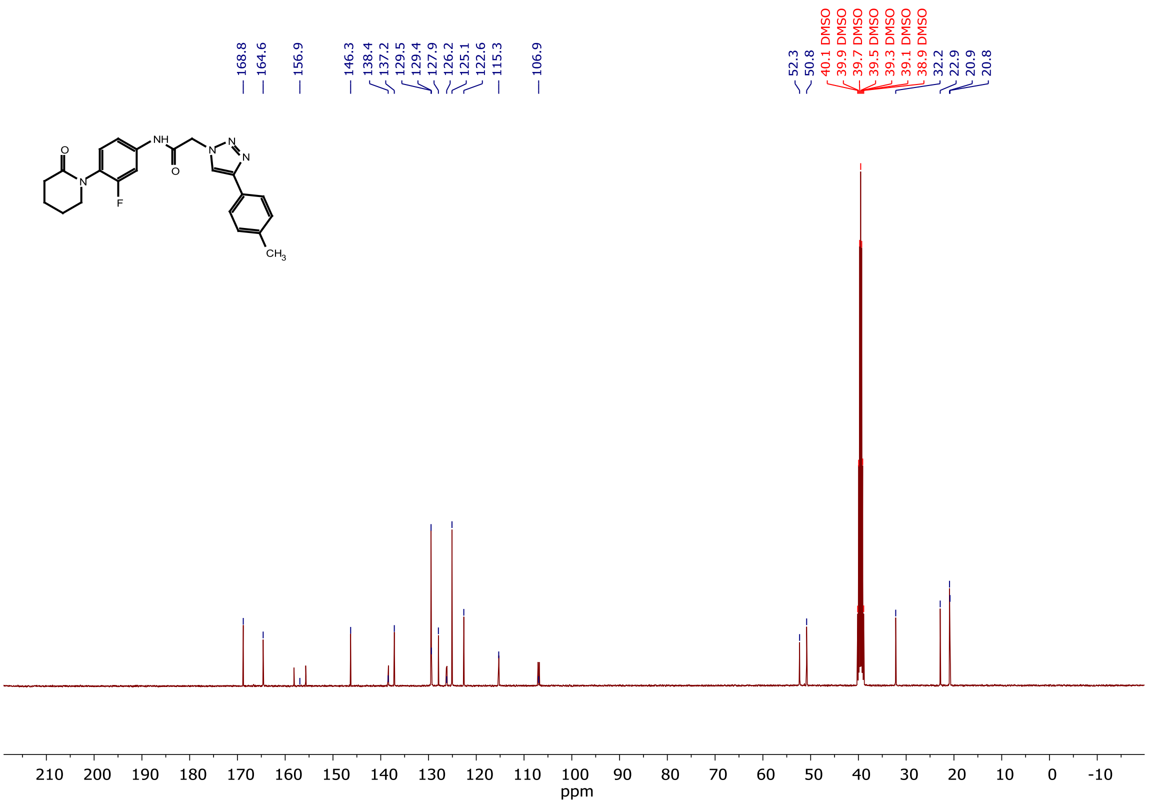

Figure S69. ^{13}C NMR (100 MHz, DMSO-d_6) of *N*-(3-fluoro-4-(2-oxopiperidin-1-yl)phenyl)-2-(4-(*p*-tolyl)-1*H*-1,2,3-triazol-1-yl)acetamide (**8q**).

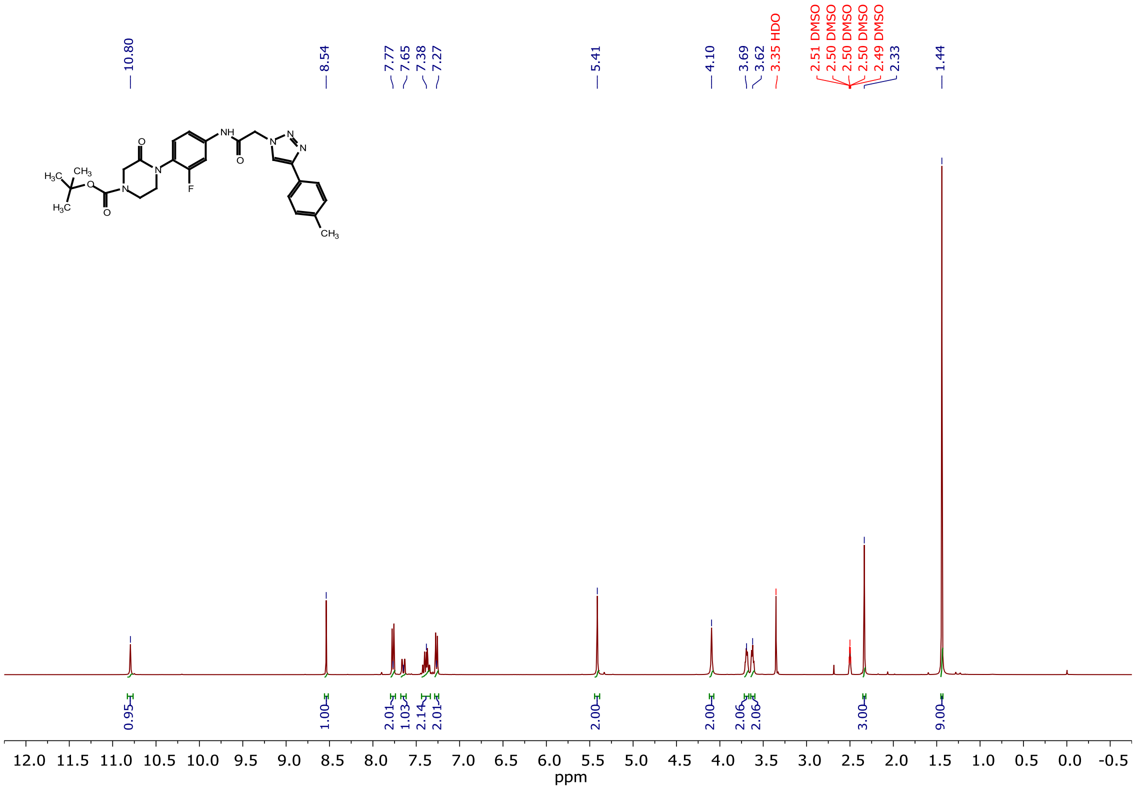

Figure S70. ^{1}H NMR (400 MHz, DMSO-d_6) of *tert*-butyl4-(2-fluoro-4-(2-(4-(*p*-tolyl)-1*H*-1,2,3-triazol-1-yl)acetamido)phenyl)-3-oxopiperazine-1-carboxylate (**8r**).

.

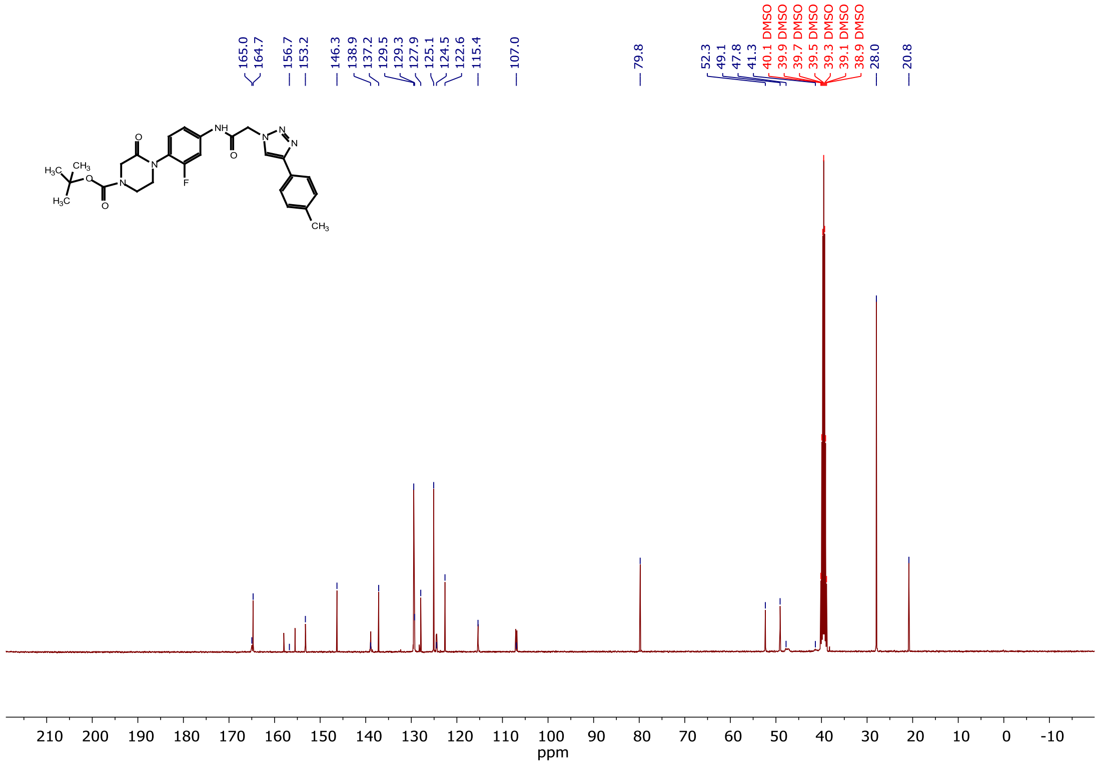

Figure S71. ^{13}C NMR (100 MHz, DMSO-d_6) of *tert*-butyl4-(2-fluoro-4-(2-(4-(*p*-tolyl)-1*H*-1,2,3-triazol-1-yl)acetamido)phenyl)-3-oxopiperazine-1-carboxylate (**8r**).

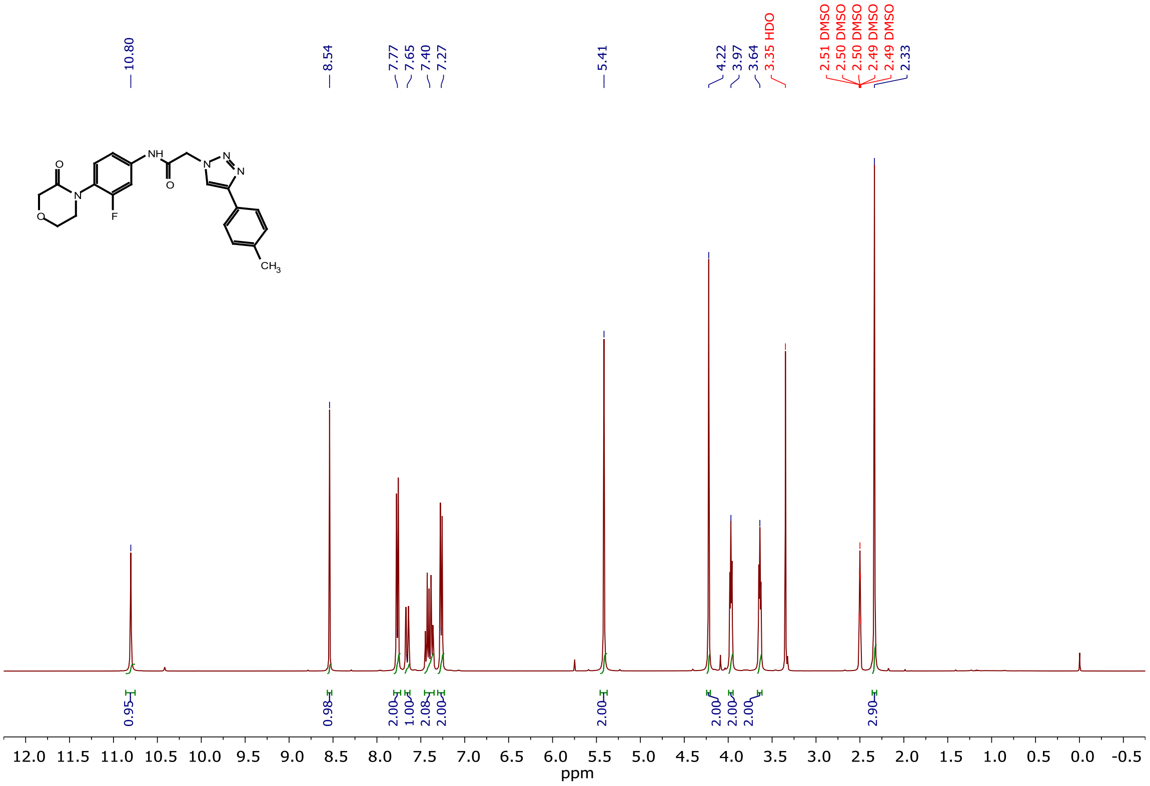

Figure S72. ^{1}H NMR (400 MHz, DMSO-d_6) of *N*-(3-fluoro-4-(3-oxomorpholino)phenyl)-2-(4-(*p*-tolyl)-1*H*-1,2,3-triazol-1-yl)acetamide (**8s**).

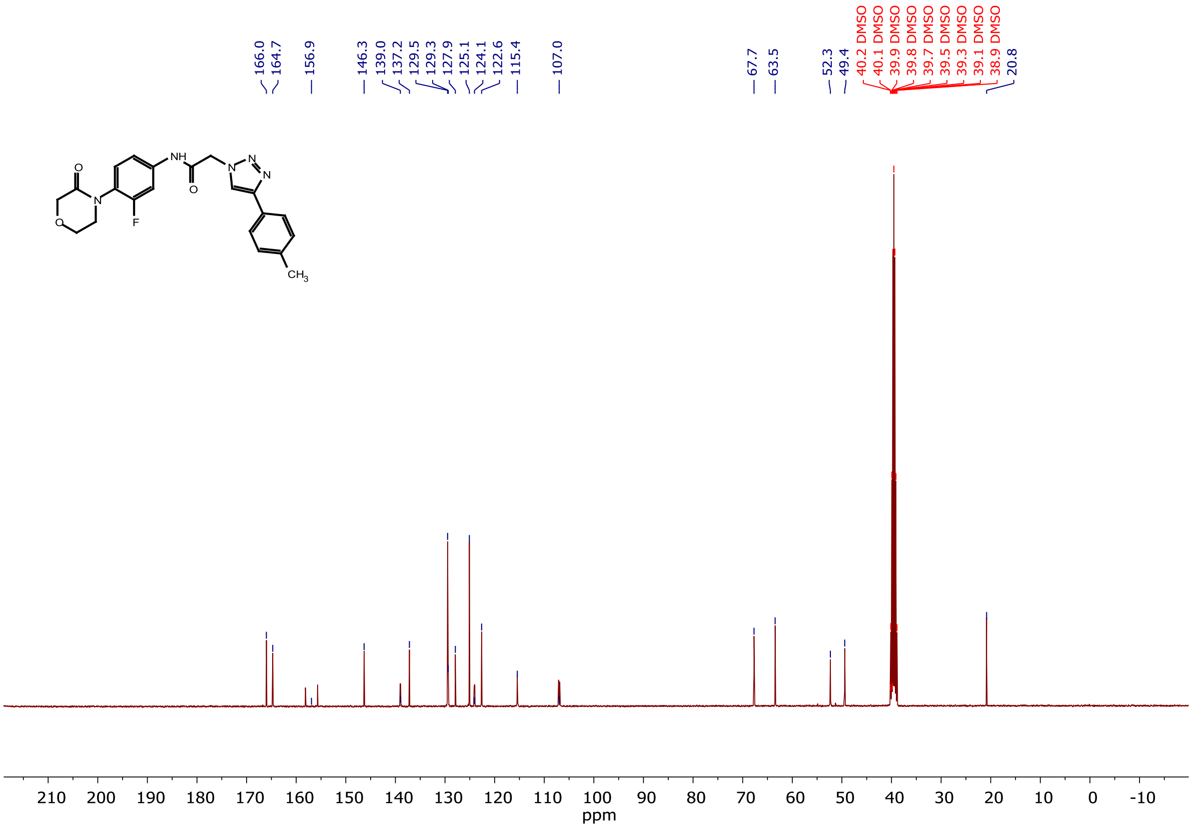

Figure S73. ^{13}C NMR (100 MHz, DMSO-d_6) of *N*-(3-fluoro-4-(3-oxomorpholino)phenyl)-2-(4-(*p*-tolyl)-1*H*-1,2,3-triazol-1-yl)acetamide (**8s**).

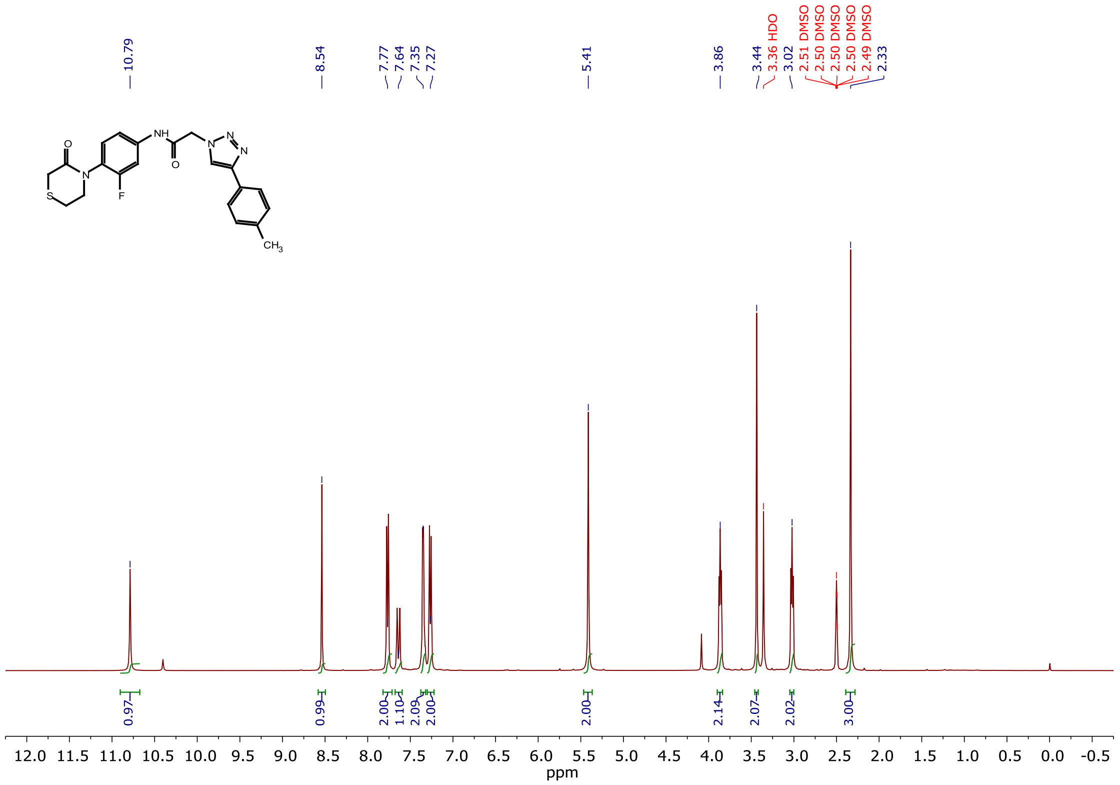

Figure 74. ^{1}H NMR (400 MHz, DMSO-*d*6) of *N*-(3-fluoro-4-(3-oxothiomorpholino)phenyl)-2-(4-(*p*-tolyl)-1*H*-1,2,3-triazol-1-yl)acetamide (**8t**).

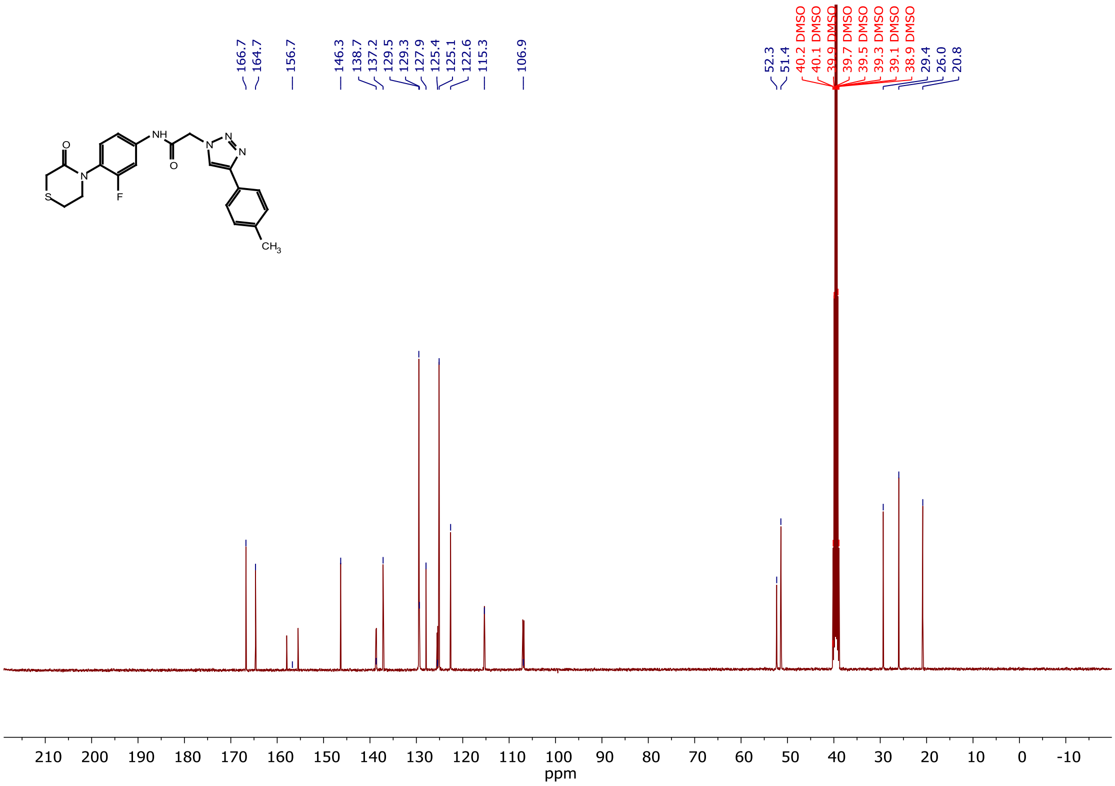

Figure S75. ^{13}C NMR (100 MHz, DMSO-d6) of *N*-(3-fluoro-4-(3-oxothiomorpholino)phenyl)-2-(4-(*p*-tolyl)-1*H*-1,2,3-triazol-1-yl)acetamide (**8t**).

11.2. *^{19}F NMR spectra*

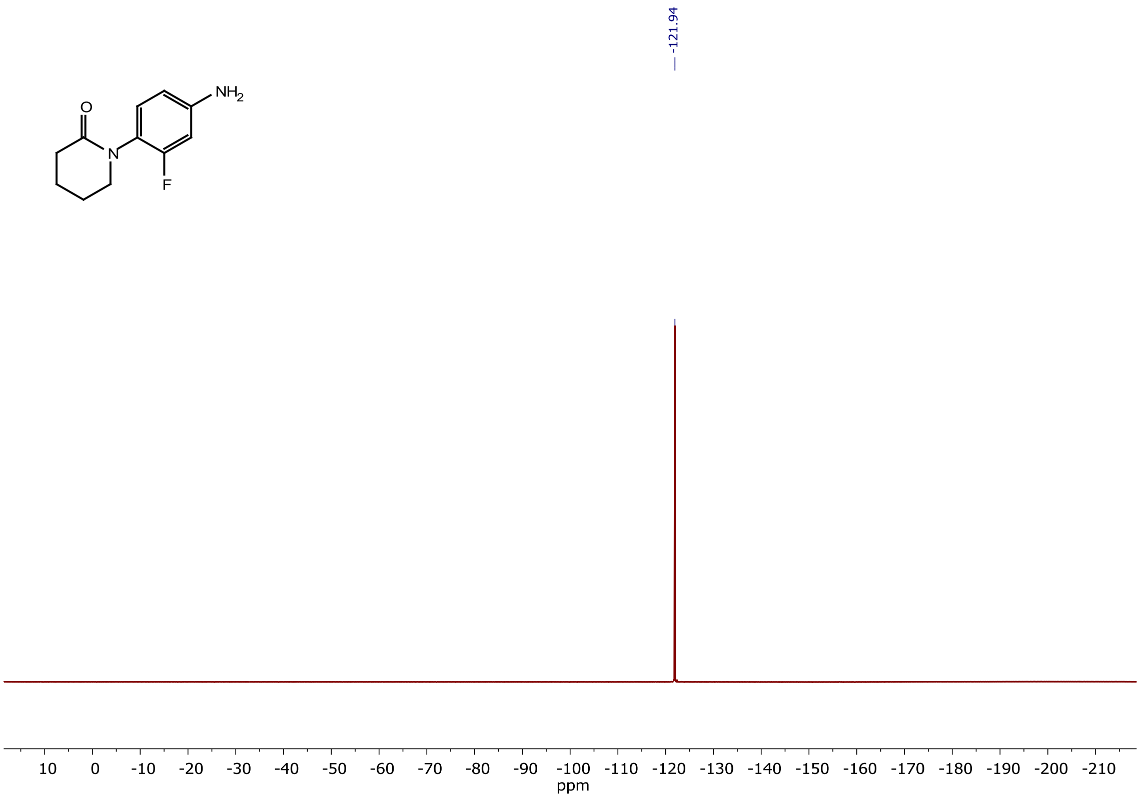

Figure S76. ^{19}F NMR (376 MHz, $CDCl_3$) 1-(4-amino-2-fluorophenyl)piperidin-2-one (**3a**).

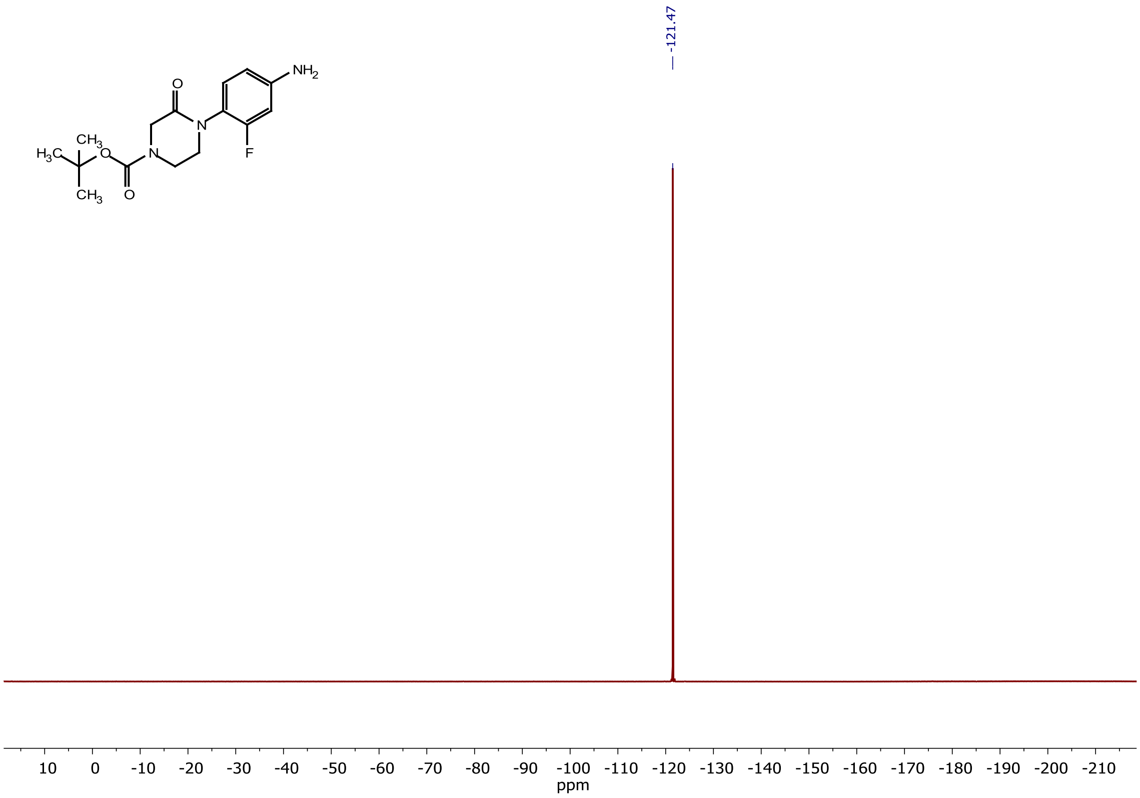

Figure S77. ^{19}F NMR (376 MHz, $CDCl_3$) *t*ert-butyl 4-(4-amino-2-fluorophenyl)-3-oxopiperazine-1-carboxylate (**3b**).

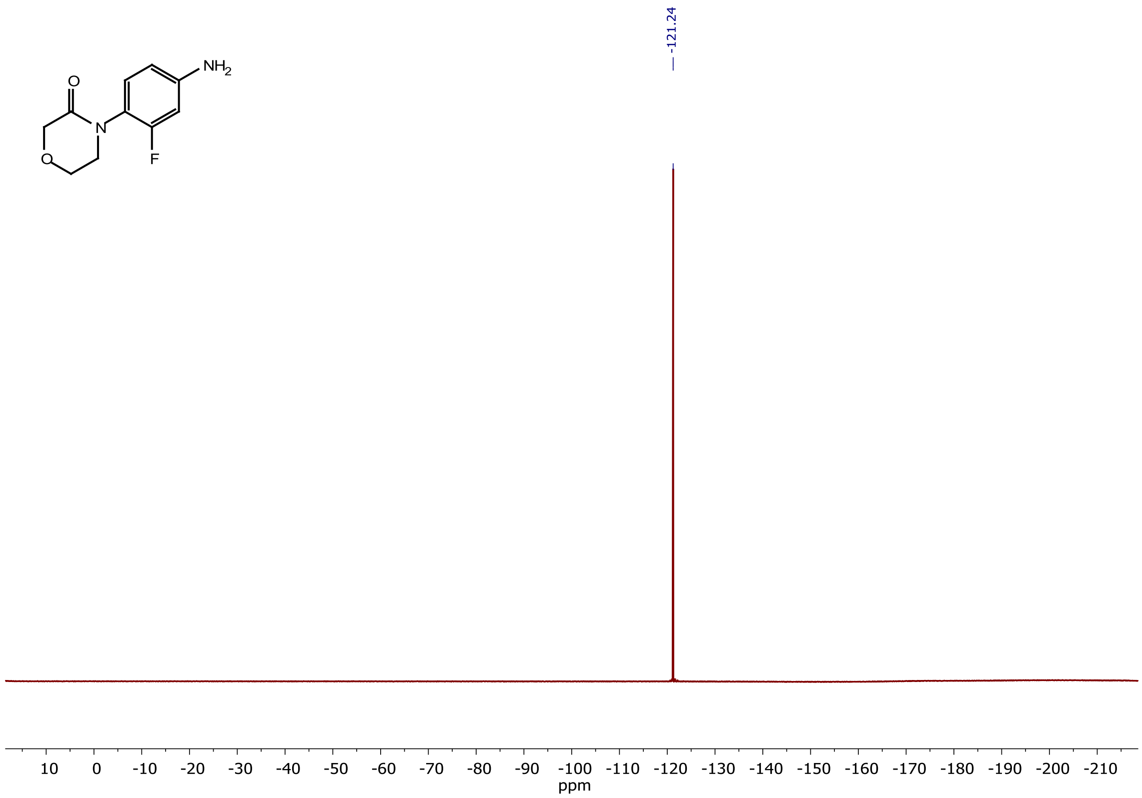

Figure S78 ^{19}F NMR (376 MHz, $CDCl_3$) 4-(4-amino-2-fluorophenyl)morpholin-3-one (**3c**).

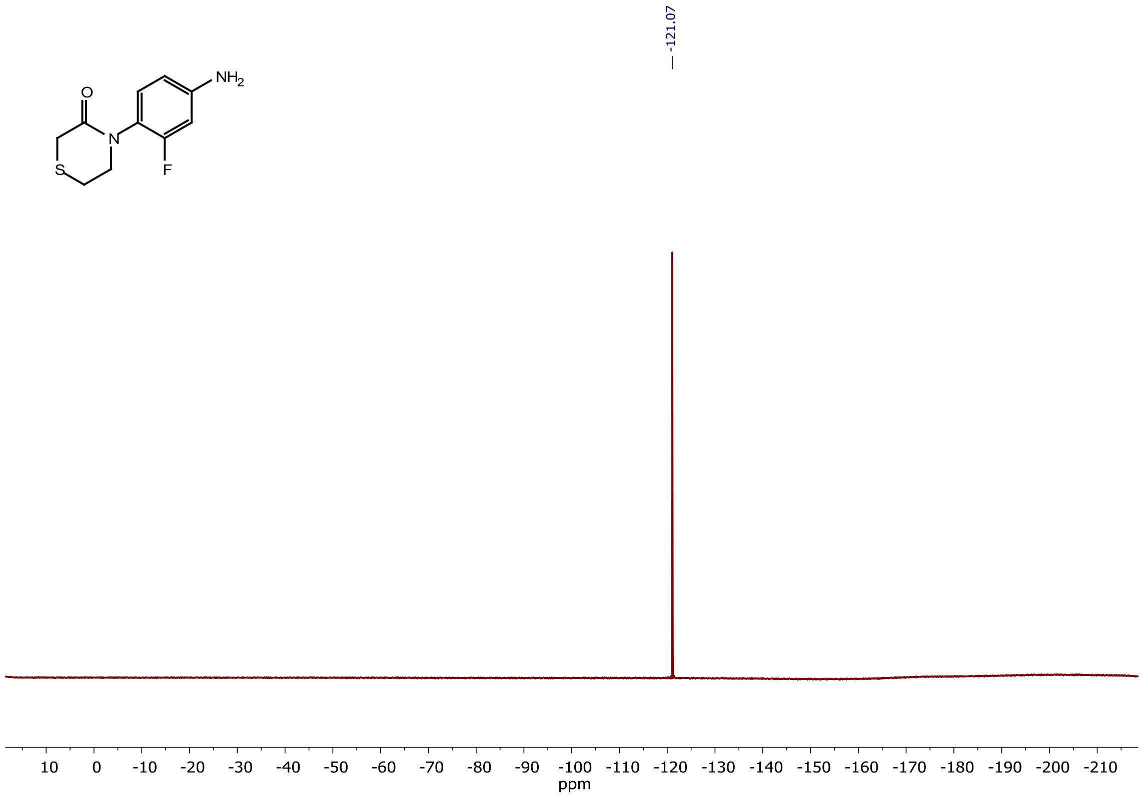

Figure S79. ^{19}F NMR (376 MHz, $CDCl_3$) 4-(4-amino-2-fluorophenyl)thiomorpholin-3-one (**3d**).

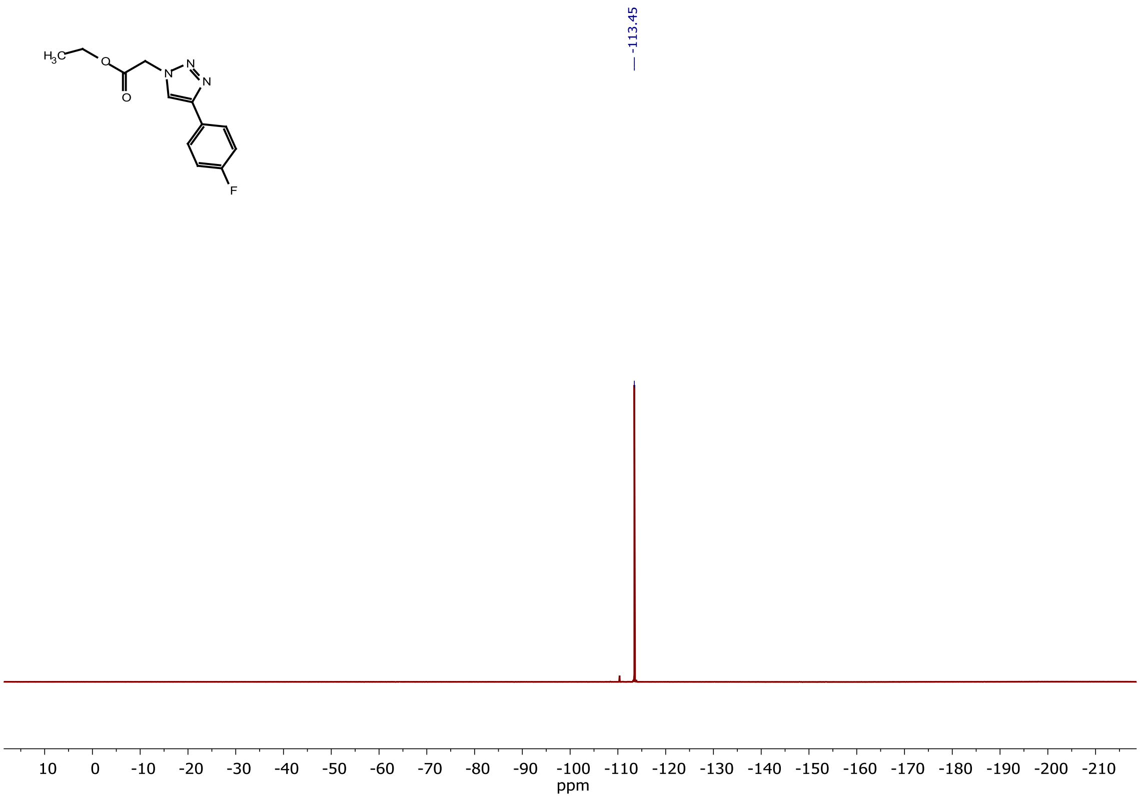

Figure S80. ^{19}F NMR (376 MHz, $CDCl_3$) ethyl 2-(4-(4-fluorophenyl)-1*H*-1,2,3-triazol-1-yl)acetate (**6c**).

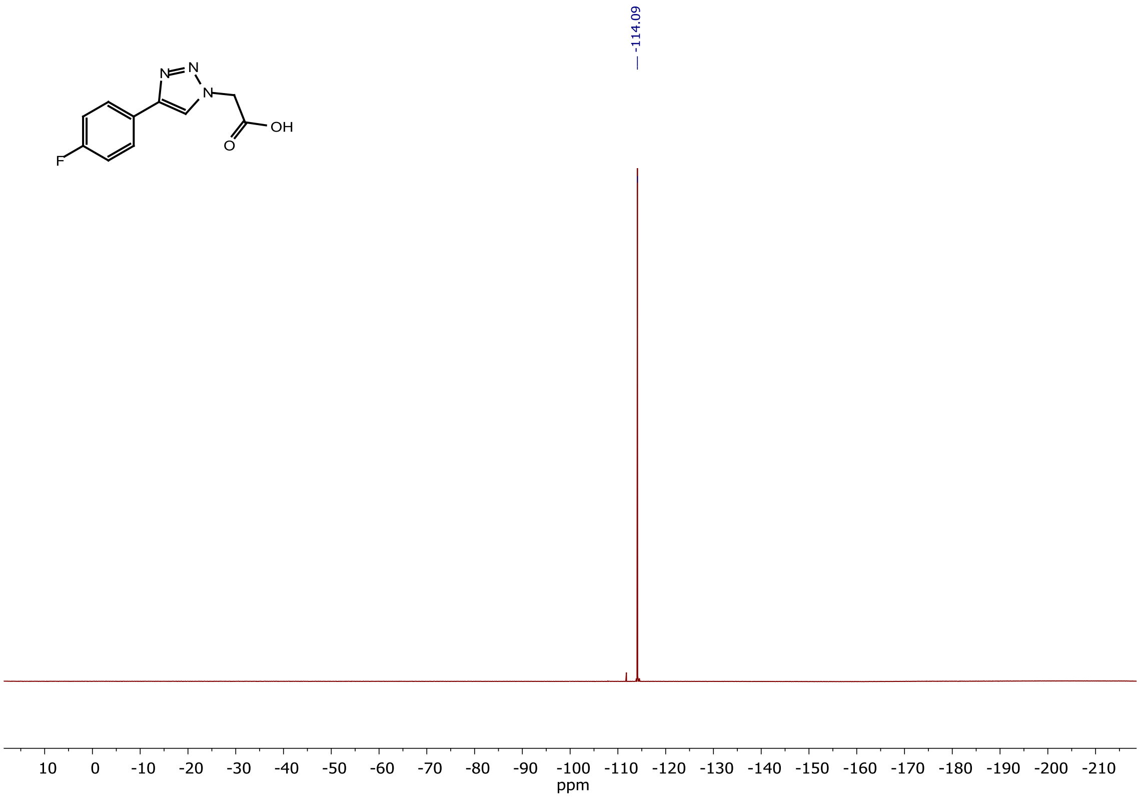

Figure S81. ^{19}F NMR (376 MHz, DMSO-d_6) 2-(4-(4-fluorophenyl)-1*H*-1,2,3-triazol-1-yl)acetic acid (**7c**).

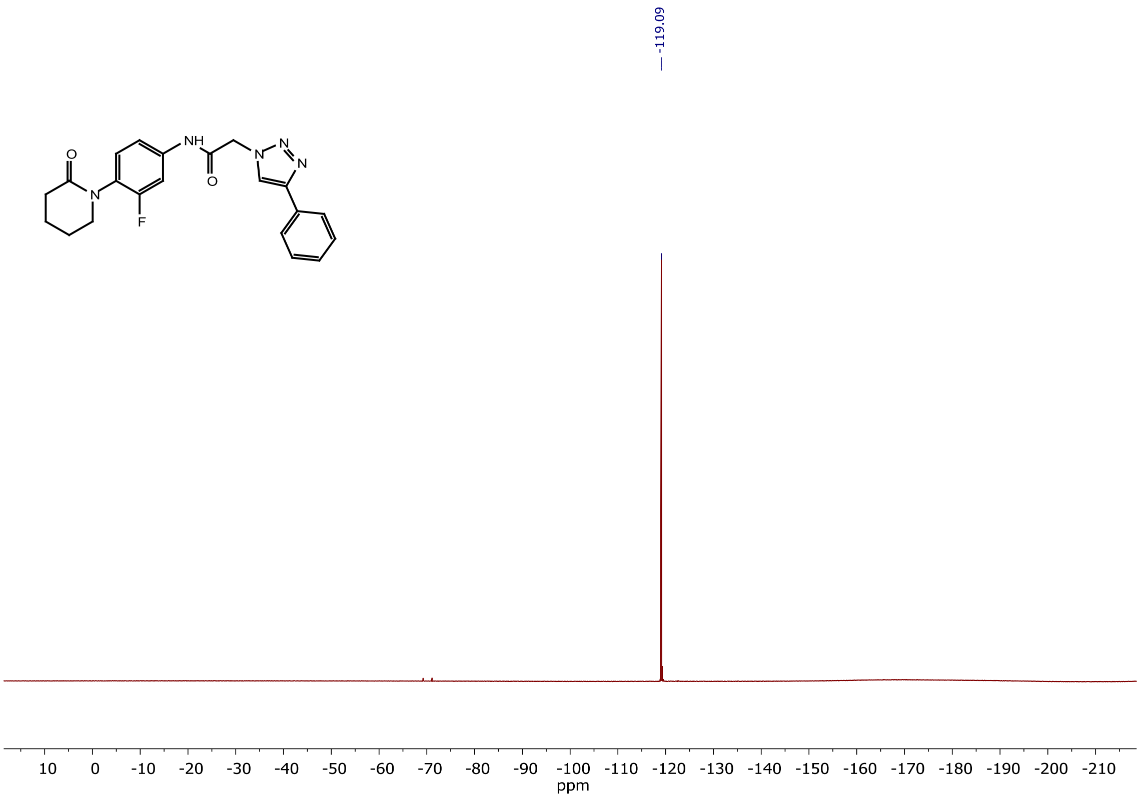

Figure S82. ^{19}F NMR (376 MHz, DMSO-d_6) *N*-(3-fluoro-4-(2-oxopiperidin-1-yl)phenyl)-2-(4-phenyl-1*H*-1,2,3-triazol-1-yl)acetamide (**8a**).

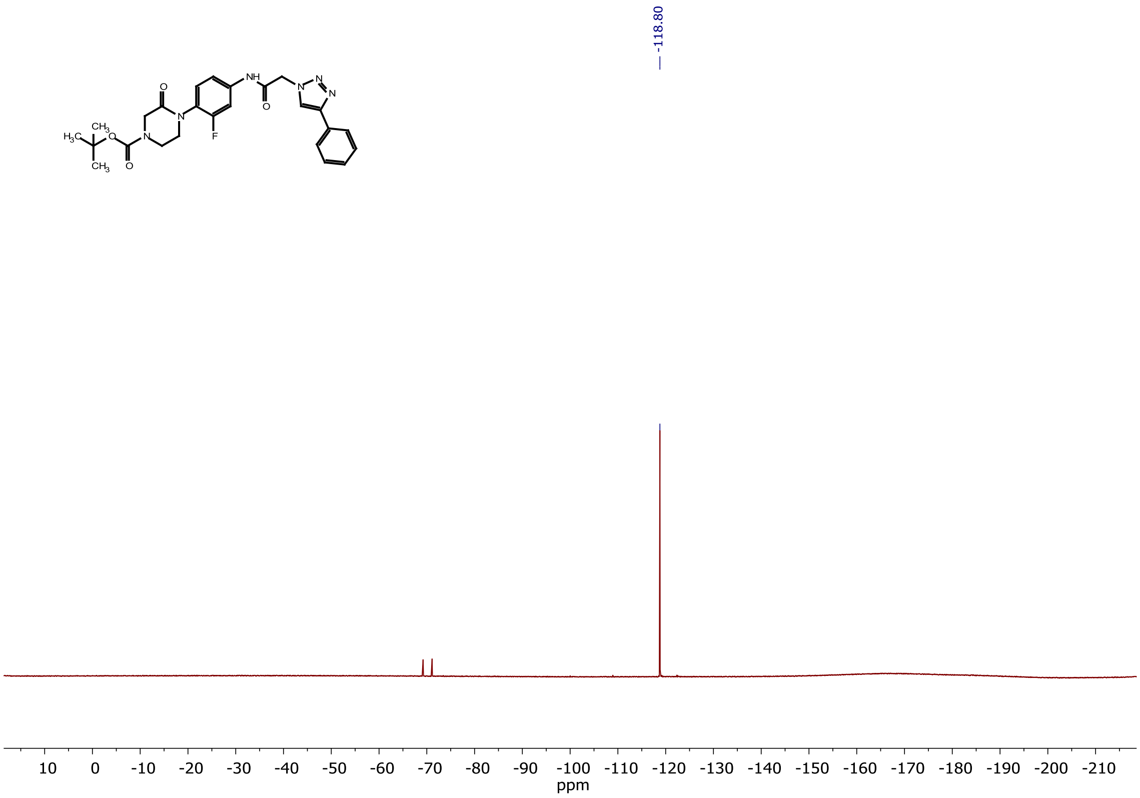

Figure S83. ^{19}F NMR (376 MHz, DMSO-d_6) *tert*-butyl 4-(2-fluoro-4-(2-(4-phenyl-1*H*-1,2,3-triazol-1-yl)acetamido)phenyl)-3-oxopiperazine-1 carboxylate (**8b**).

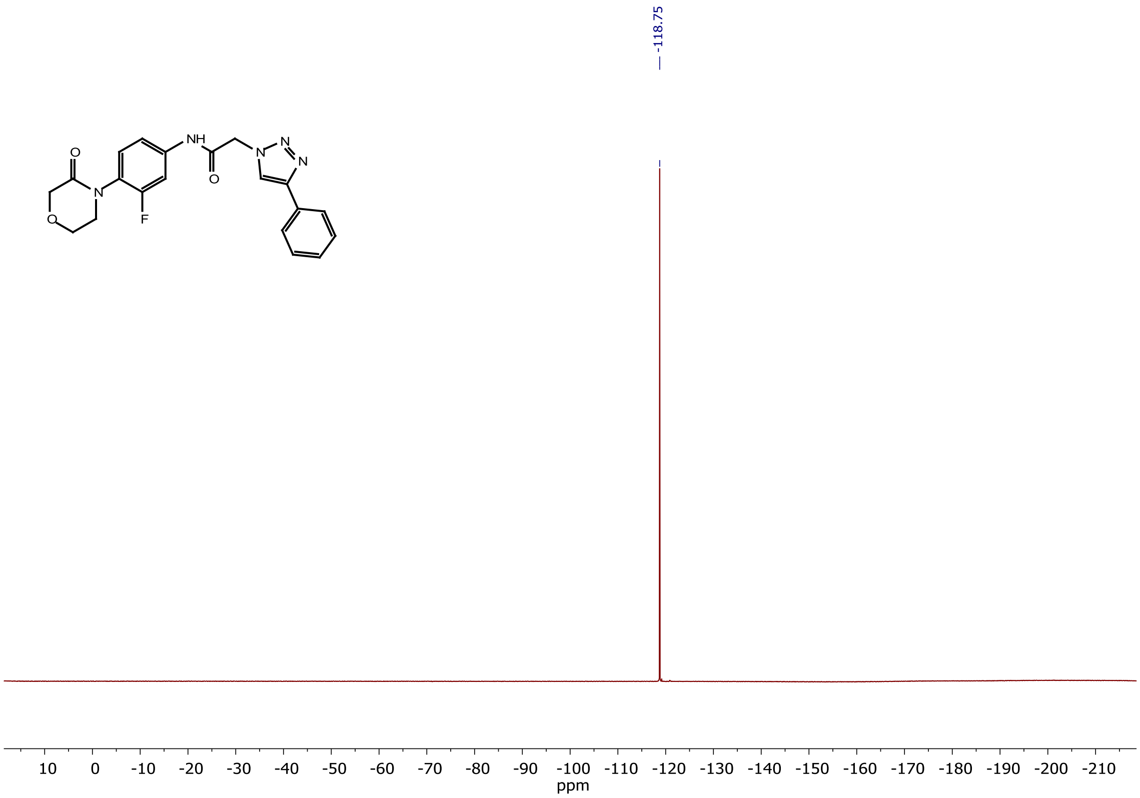

Figure S84. ^{19}F NMR (376 MHz, DMSO-d_6) *N*-(3-fluoro-4-(3-oxomorpholino)phenyl)-2-(4-phenyl-1*H*-1,2,3-triazol-1-yl)acetamide (**8c**).

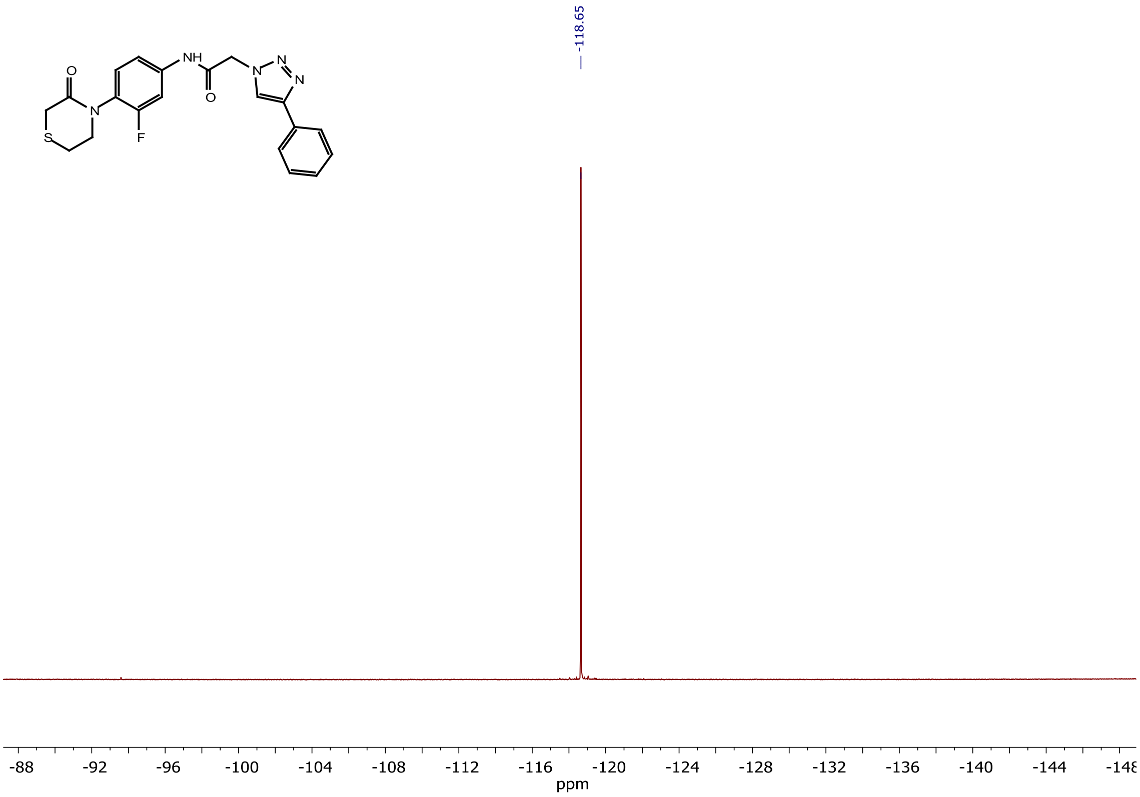

Figure S85. ^{19}F NMR (376 MHz, DMSO-d_6) *N*-(3-fluoro-4-(3-oxothiomorpholino)phenyl)-2-(4-phenyl-1*H*-1,2,3-triazol-1-yl)acetamide (**8d**).

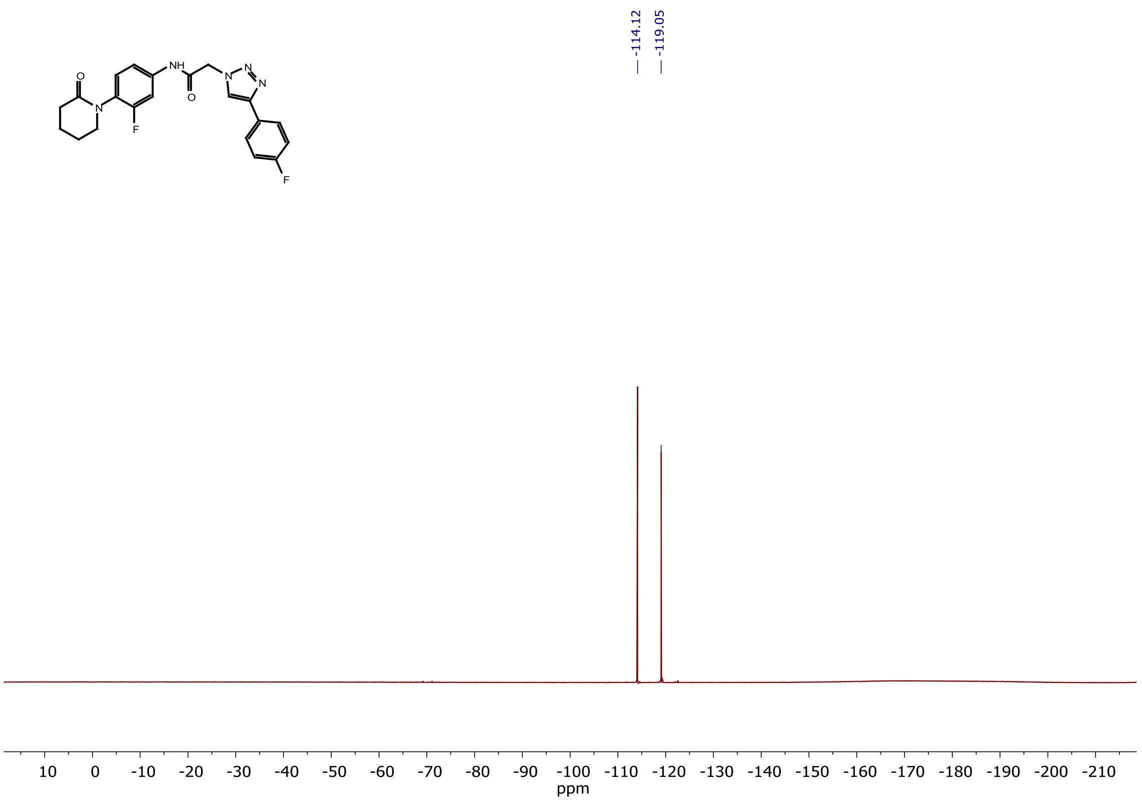

Figure S86. ^{19}F NMR (376 MHz, DMSO-*d*$_6$) *N*-(3-fluoro-4-(2-oxopiperidin-1-yl)phenyl)-2-(4-(4-fluorophenyl)-1*H*-1,2,3-triazol-1-yl)acetamide (**8e**).

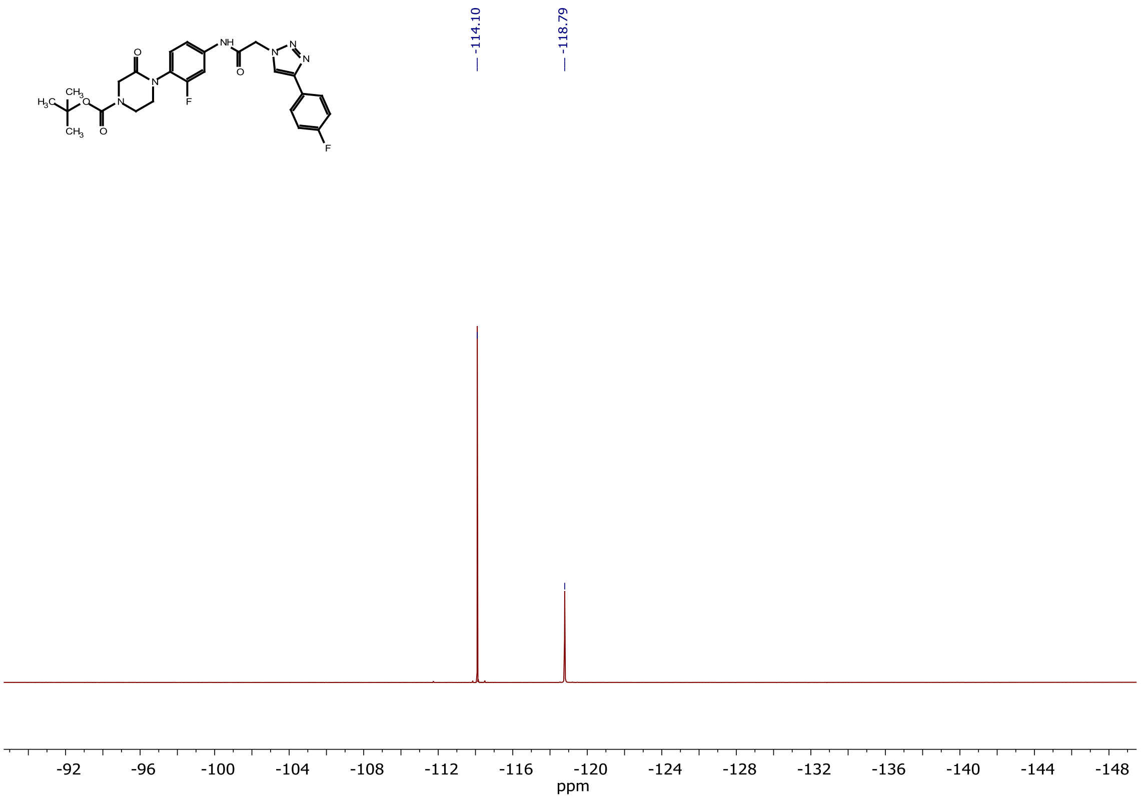

Figure S87. ^{19}F NMR (376 MHz, DMSO-*d*$_6$) *tert*-butyl 4-(2-fluoro-4-(2-(4-(4-fluorophenyl)-1*H*-1,2,3-triazol-1-yl)acetamido)phenyl)-3-oxopiperazine-1-carboxylate (**8f**).

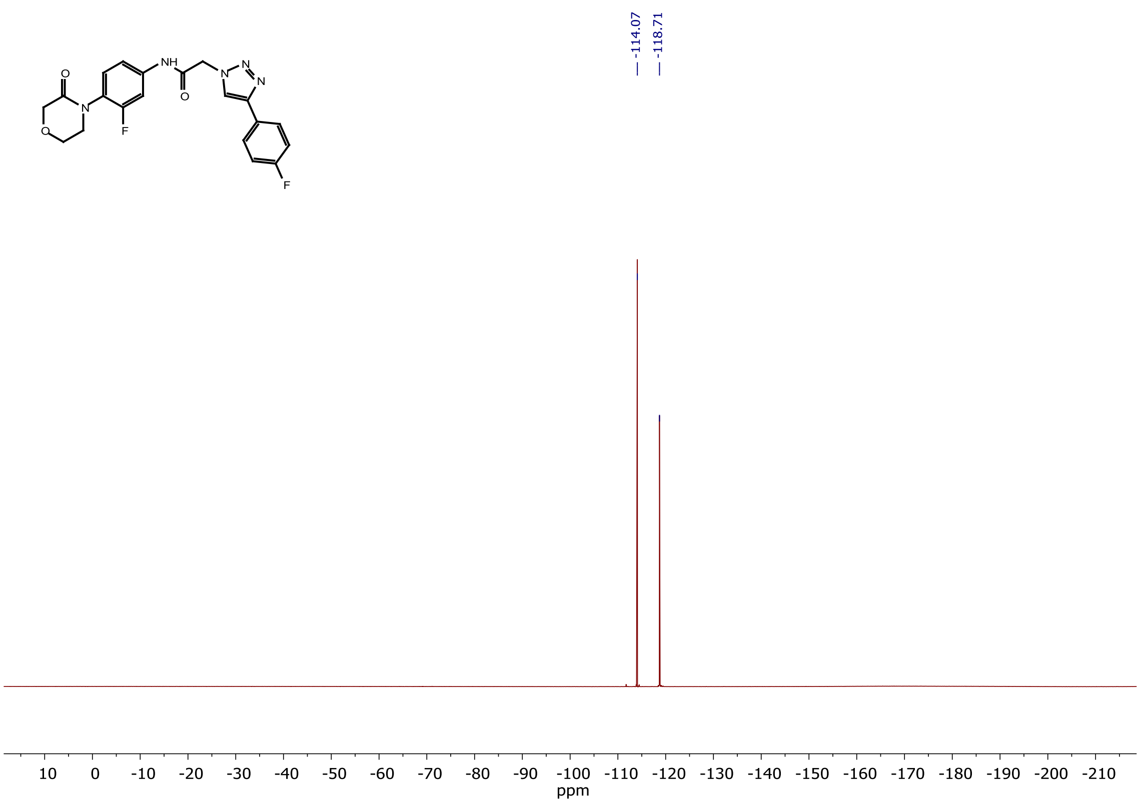

Figure S88. ^{19}F NMR (376 MHz, DMSO-*d*₆) *N*-(3-fluoro-4-(3-oxomorpholino)phenyl)-2-(4-(4-fluorophenyl)-1*H*-1,2,3-triazol-1-yl)acetamide (**8g**).

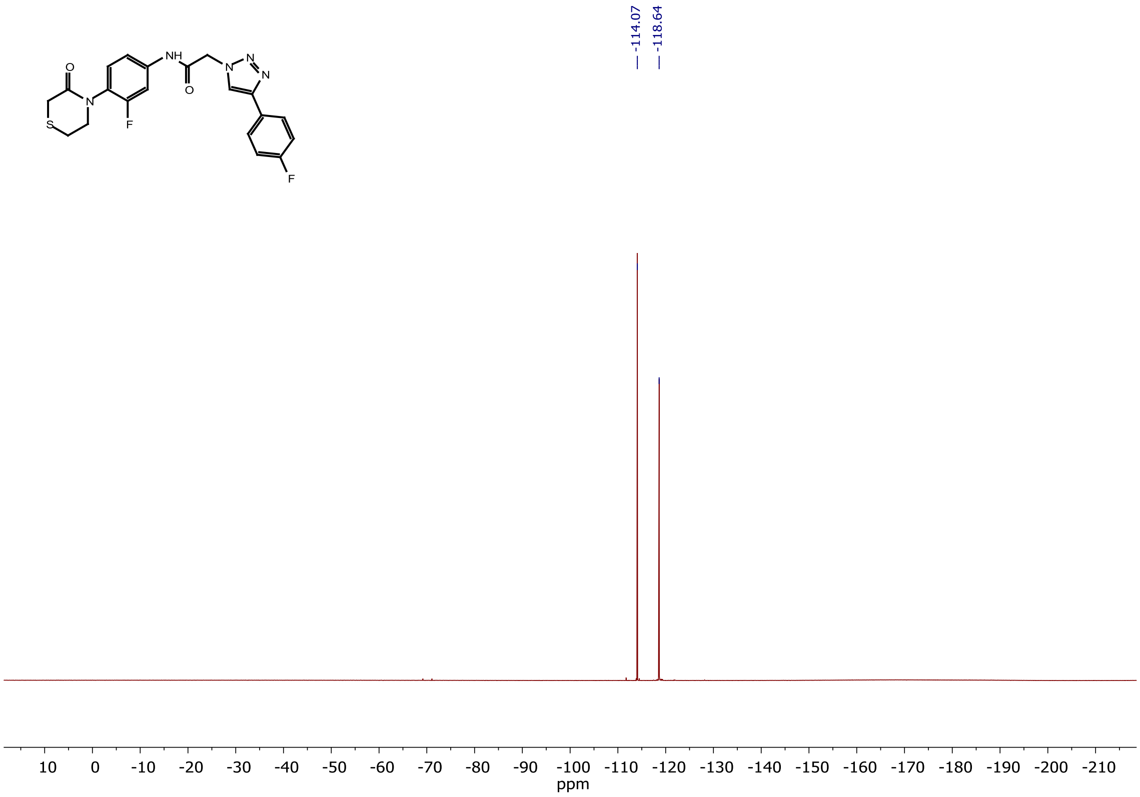

Figure S89. ^{19}F NMR (376 MHz, DMSO-*d*₆) *N*-(3-fluoro-4-(3-oxothiomorpholino)phenyl)-2-(4-(4-fluorophenyl)-1*H*-1,2,3-triazol-1-yl)acetamide (**8h**).

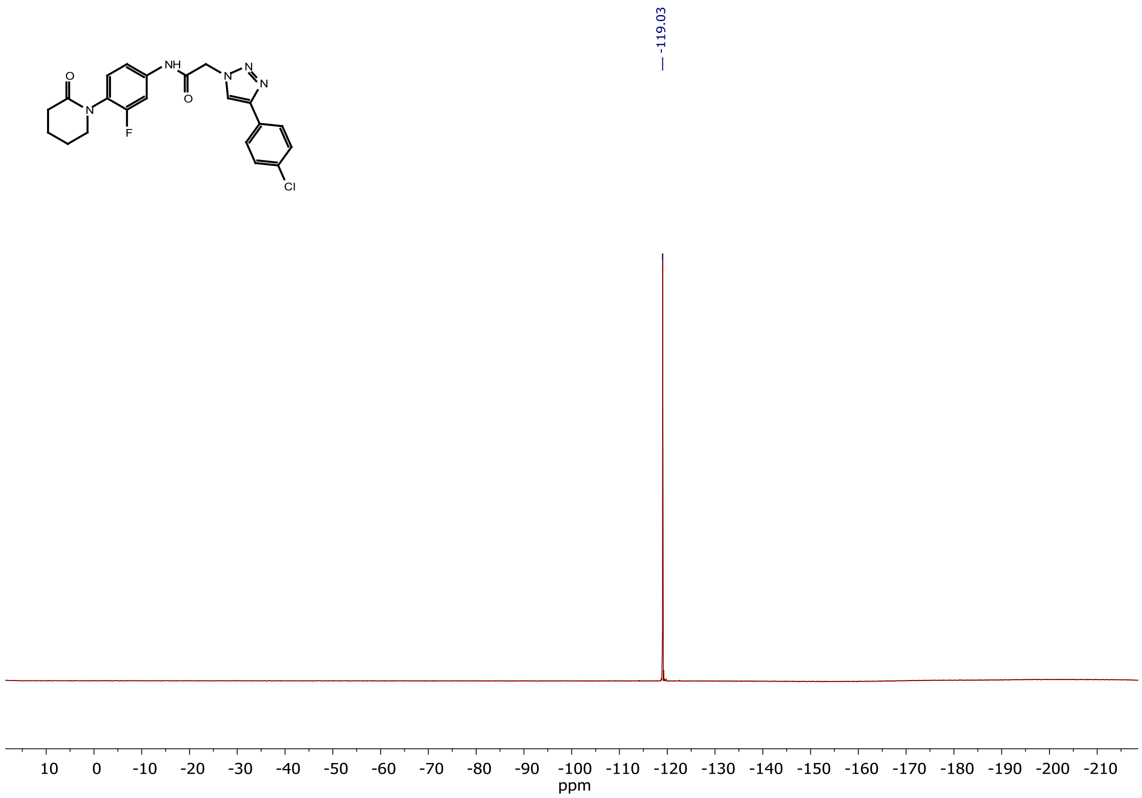

Figure S90. ^{19}F NMR (376 MHz, DMSO-*d*6) 2-(4-(4-chlorophenyl)-1*H*-1,2,3-triazol-1-yl)-N-(3-fluoro-4-(2-oxopiperidin-1-yl)phenyl)acetamide (**8i**).

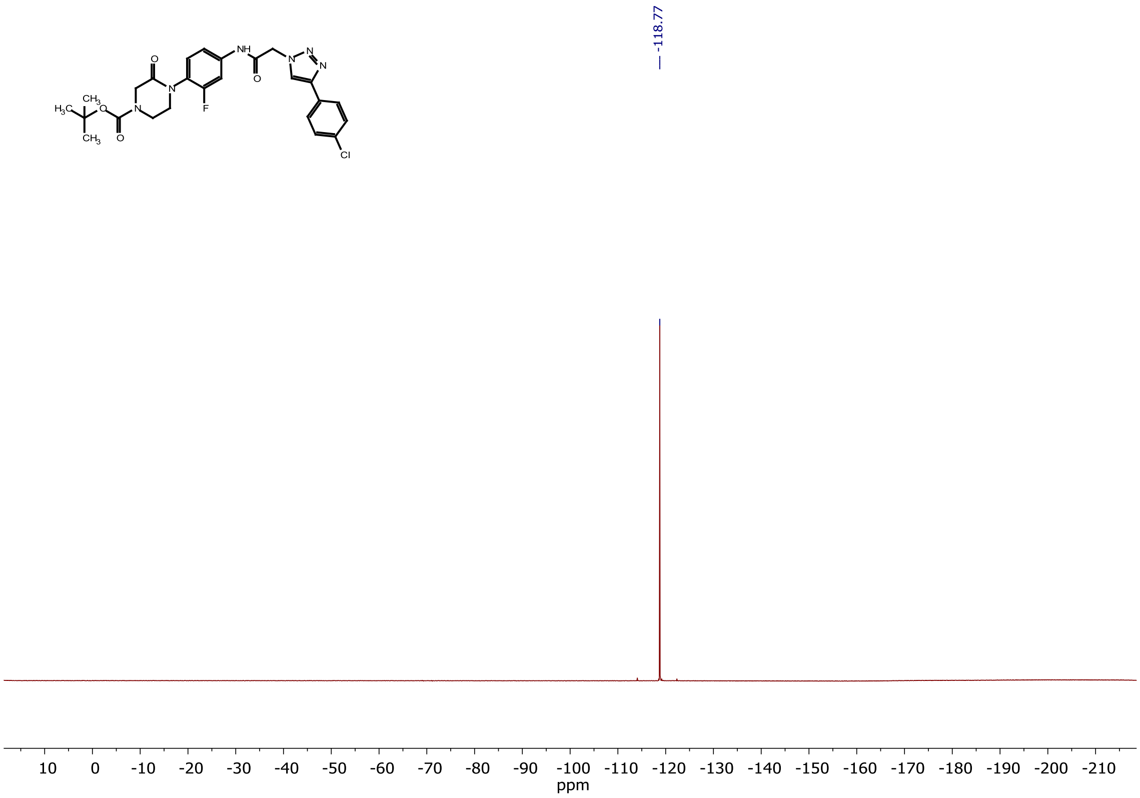

Figure S91. ^{19}F NMR (376 MHz, DMSO-*d*6) *tert*-butyl 4-(4-(2-(4-(4-chlorophenyl)-1*H*-1,2,3-triazol-1-yl)acetamido)-2-fluorophenyl)-3-oxopiperazine-1-carboxylate (**8j**).

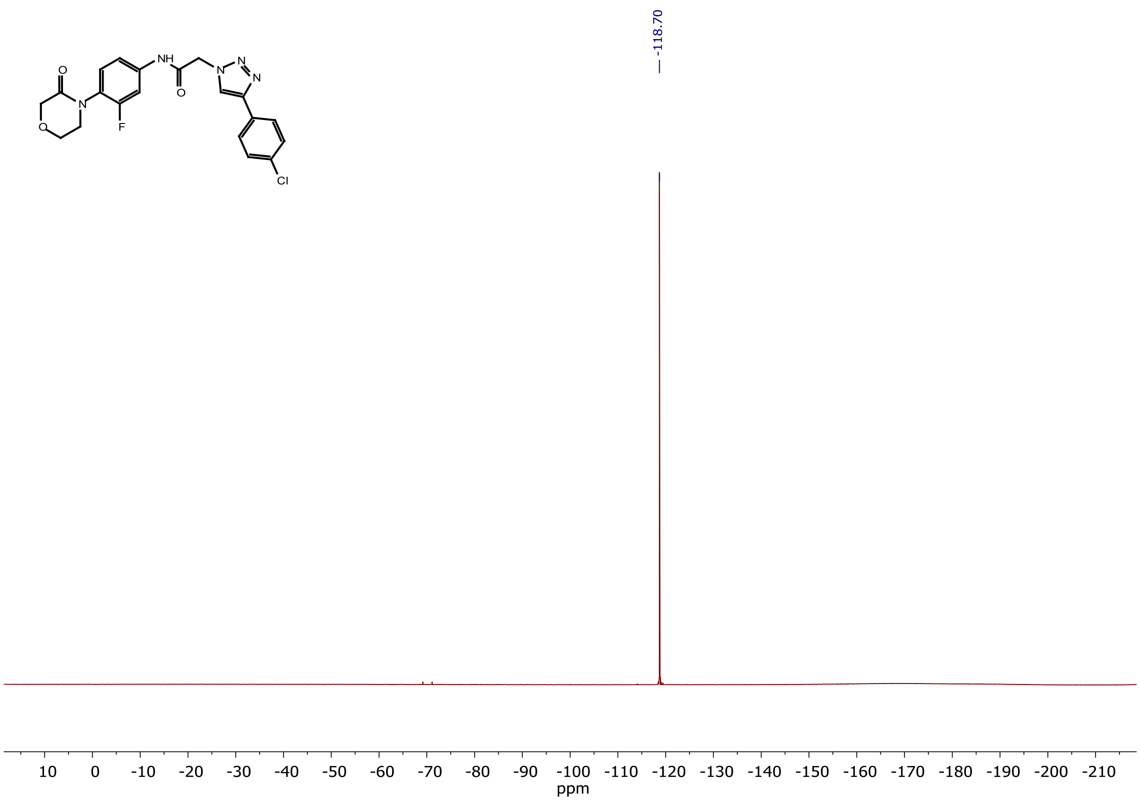

Figure S92. ^{19}F NMR (376 MHz, DMSO-d_6) 2-(4-(4-chlorophenyl)-1*H*-1,2,3-triazol-1-yl)-*N*-(3-fluoro-4-(3-oxomorpholino)phenyl)acetamide (**8k**).

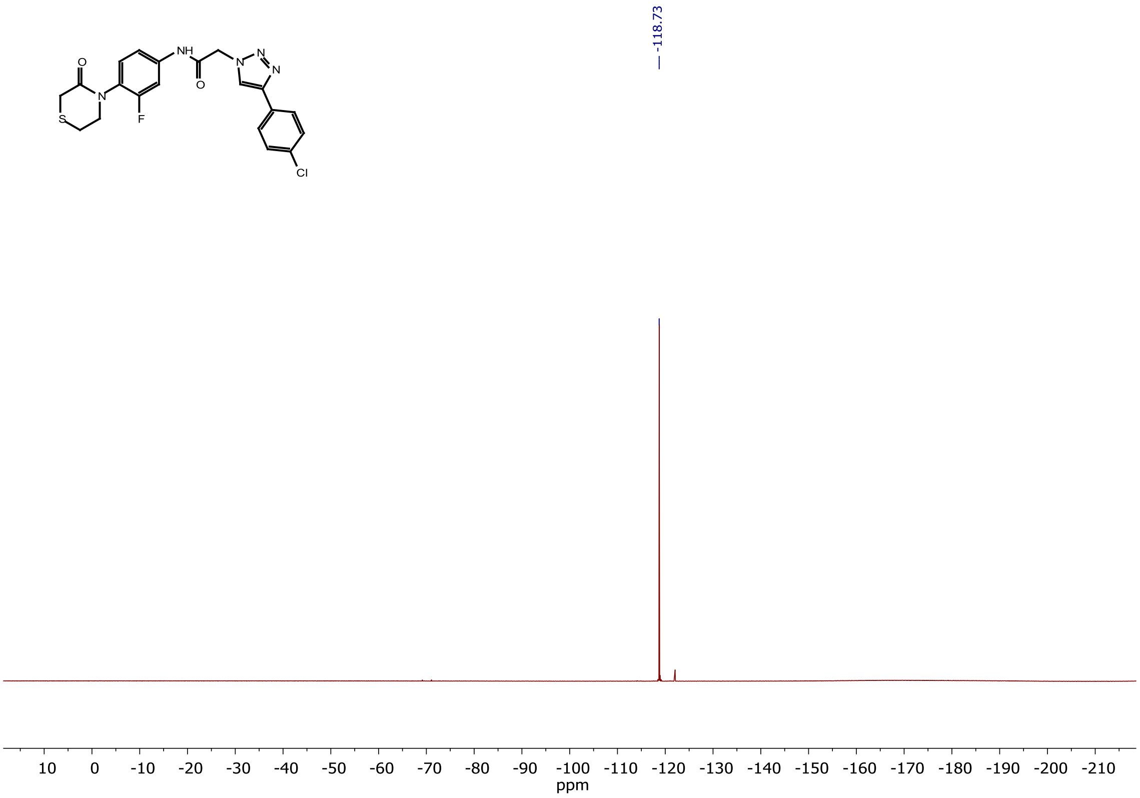

Figure S93. ^{19}F NMR (376 MHz, DMSO-d_6) 2-(4-(4-chlorophenyl)-1*H*-1,2,3-triazol-1-yl)-*N*-(3-fluoro-4-(3-oxothiomorpholino)phenyl)acetamide (**8l**).

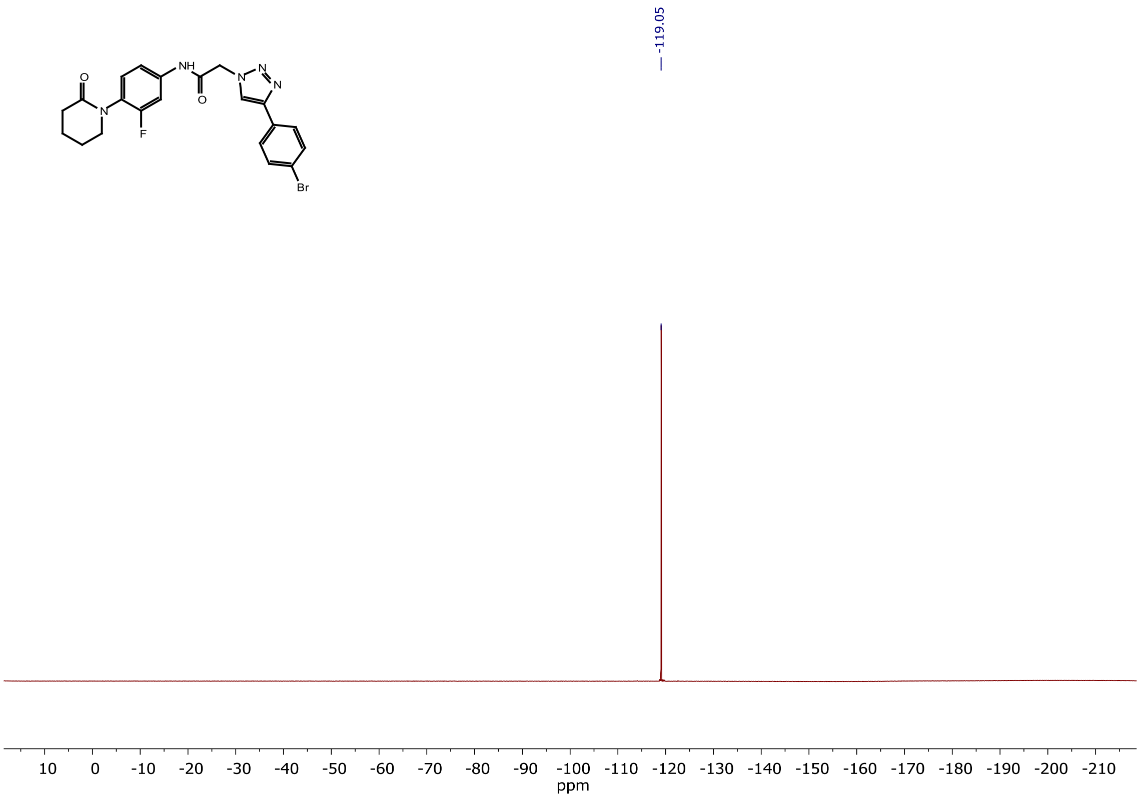

Figure S94. ^{19}F NMR (376 MHz, DMSO-d_6) 2-(4-(4-bromophenyl)-1*H*-1,2,3-triazol-1-yl)-*N*-(3-fluoro-4-(2-oxopiperidin-1-yl)phenyl)acetamide (**8m**).

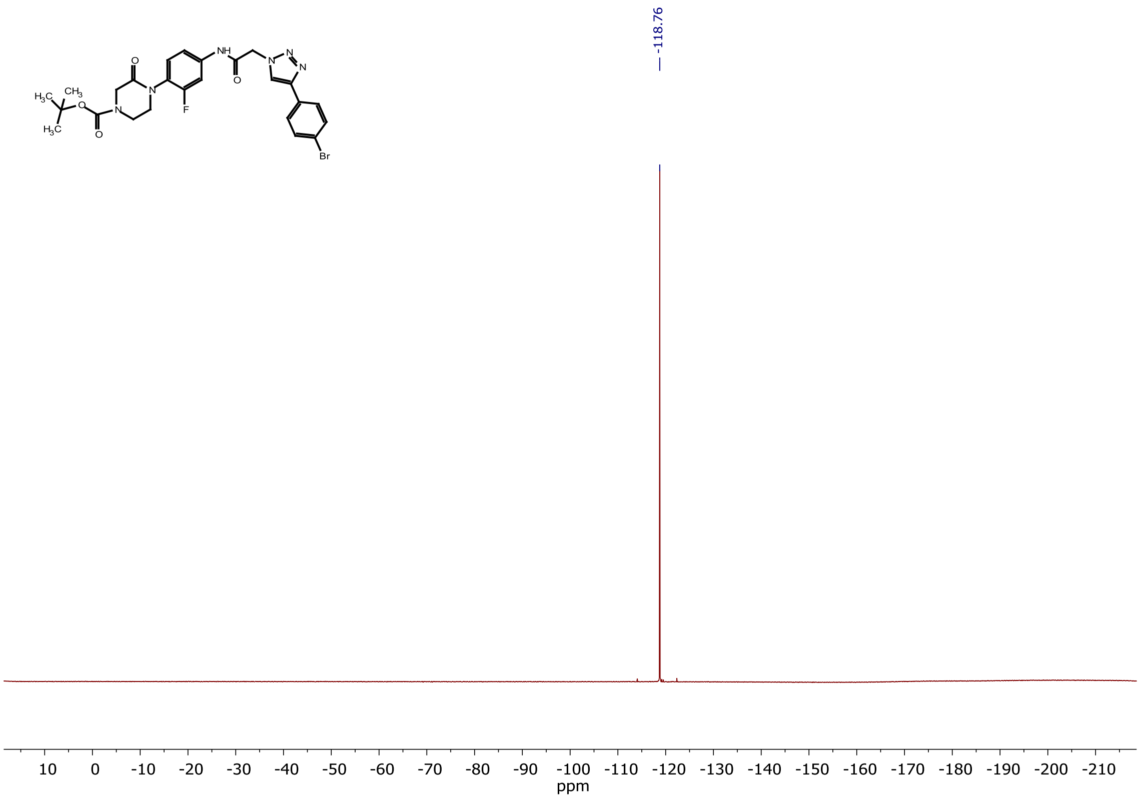

Figure S95. ^{19}F NMR (376 MHz, DMSO-d_6) *tert*-butyl 4-(4-(2-(4-(4-bromophenyl)-1*H*-1,2,3-triazol-1-yl)acetamido)-2-fluorophenyl)-3-oxopiperazine-1-carboxylate (**8n**).

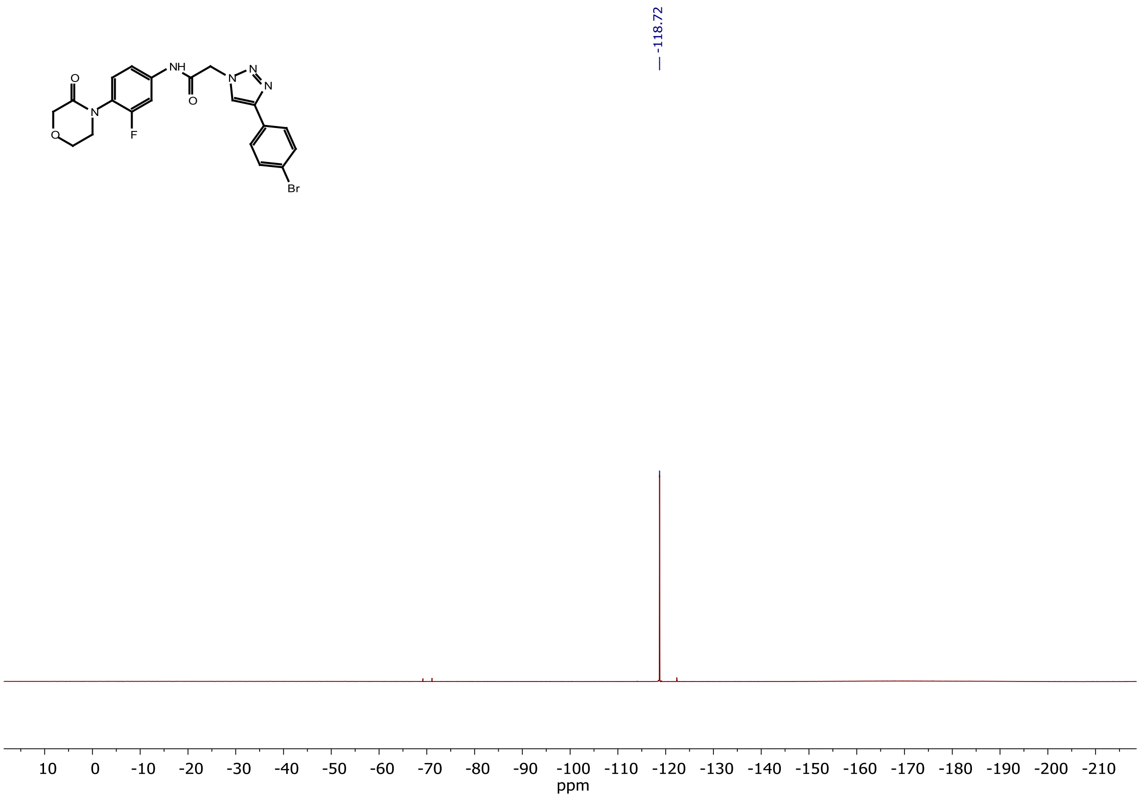

Figure S96. ^{19}F NMR (376 MHz, DMSO-d_6) 2-(4-(4-bromophenyl)-1*H*-1,2,3-triazol-1-yl)-N-(3-fluoro-4-(3-oxomorpholino)phenyl)acetamide (**8o**).

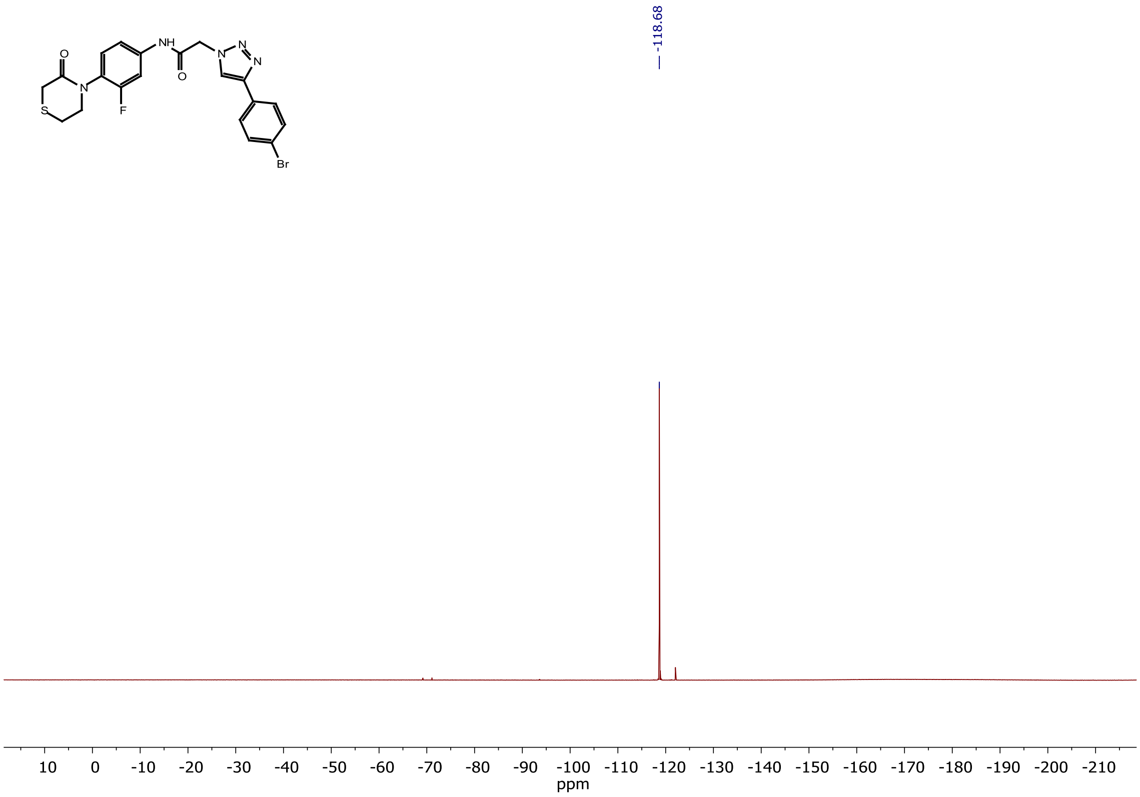

Figure S97. ^{19}F NMR (376 MHz, DMSO-d_6) 2-(4-(4-bromophenyl)-1*H*-1,2,3-triazol-1-yl)-N-(3-fluoro-4-(3-oxothiomorpholino)phenyl)acetamide (**8p**).

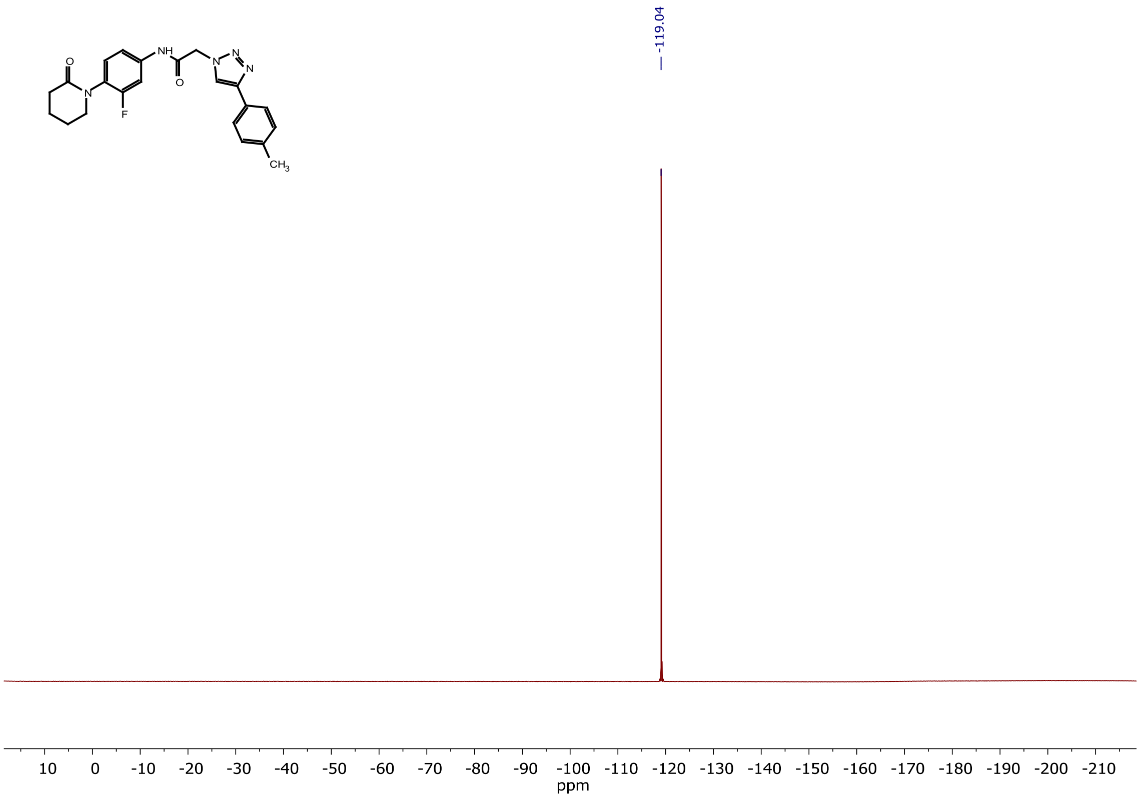

Figure S98. ^{19}F NMR (376 MHz, DMSO-d_6) *N*-(3-fluoro-4-(2-oxopiperidin-1-yl)phenyl)-2-(4-(*p*-tolyl)-1*H*-1,2,3-triazol-1-yl)acetamide (**8q**).

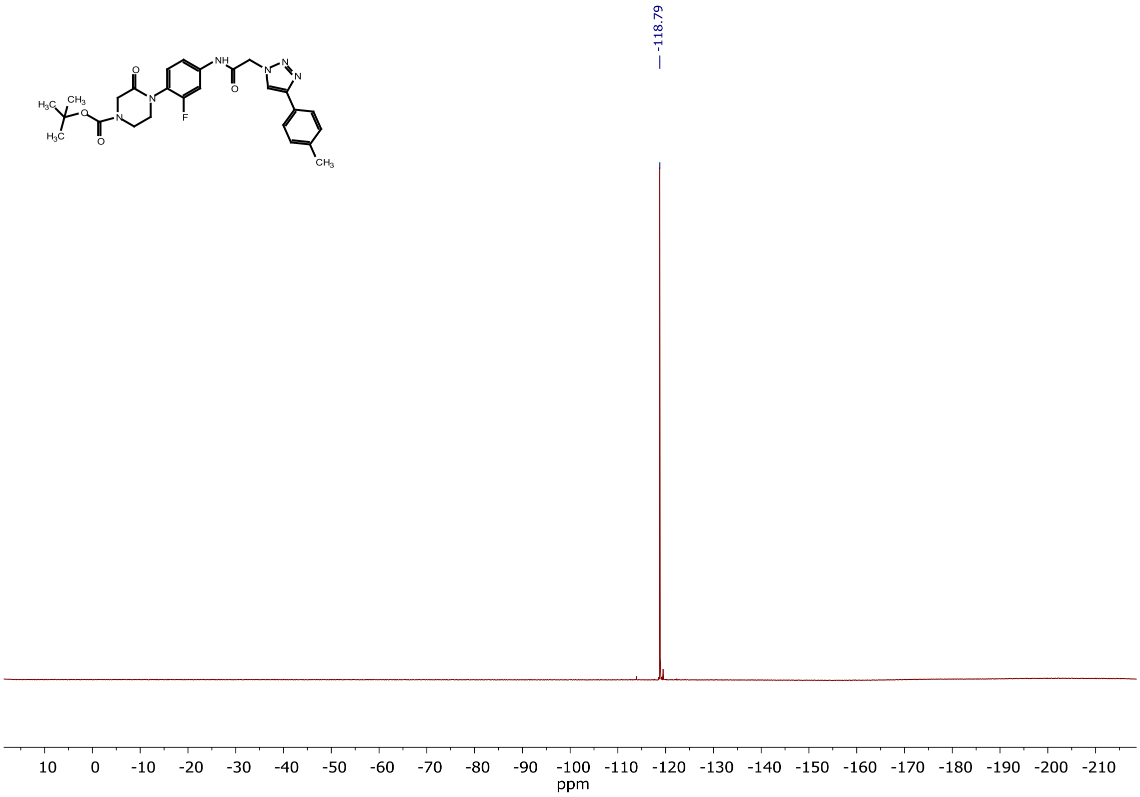

Figure S99. ^{19}F NMR (376 MHz, DMSO-d_6) *tert*-butyl4-(2-fluoro-4-(2-(4-(*p*-tolyl)-1*H*-1,2,3-triazol-1-yl)acetamido)phenyl)-3-oxopiperazine-1-carboxylate (**8r**).

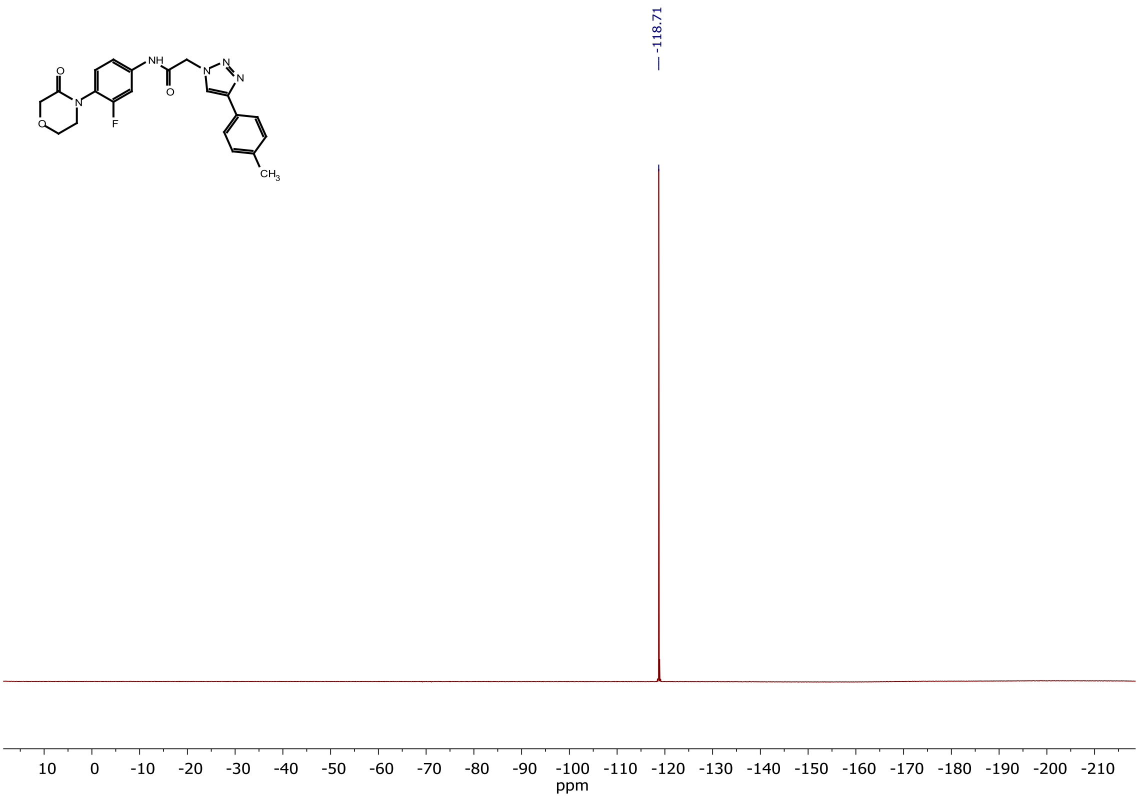

Figure S100. ^{19}F NMR (376 MHz, DMSO-d_6) *N*-(3-fluoro-4-(3-oxomorpholino)phenyl)-2-(4-(p-tolyl)-1*H*-1,2,3-triazol-1-yl)acetamide (**8s**).

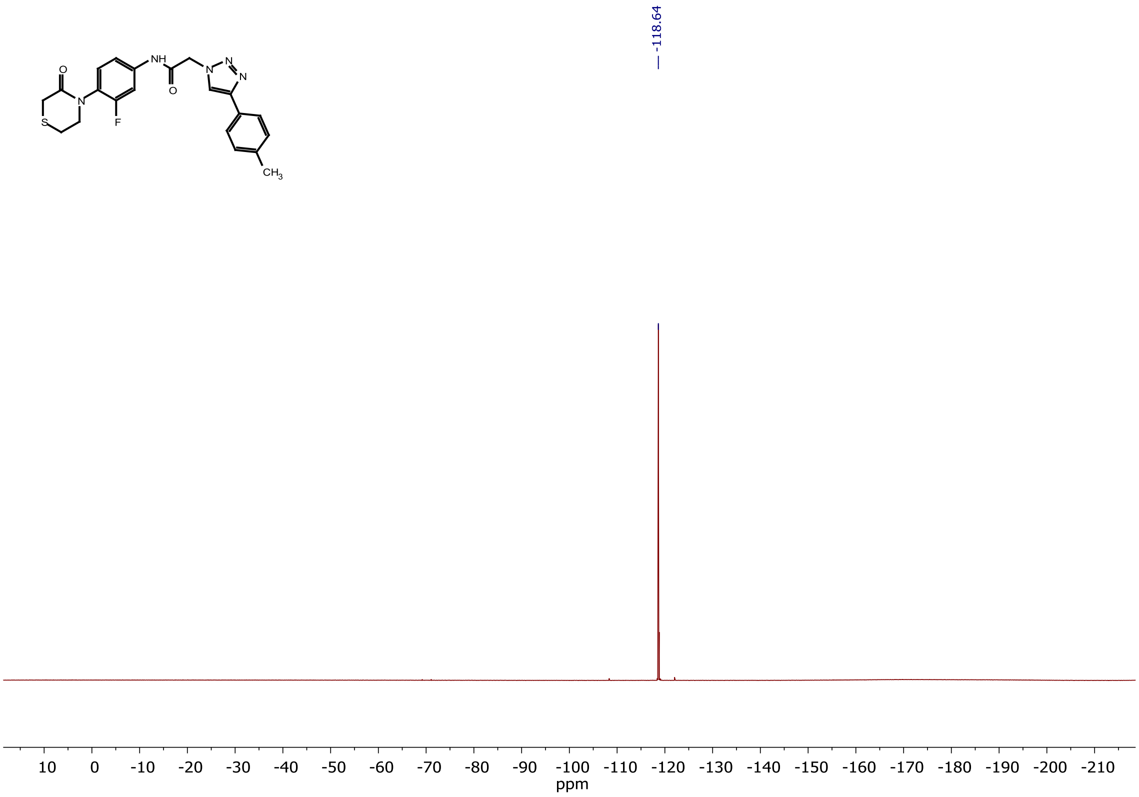

Figure S101. ^{19}F NMR (376 MHz, DMSO-d_6) *N*-(3-fluoro-4-(3-oxothiomorpholino)phenyl)-2-(4-(*p*-tolyl)-1*H*-1,2,3-triazol-1-yl)acetamide (**8t**).

11.3. FTIR spectra

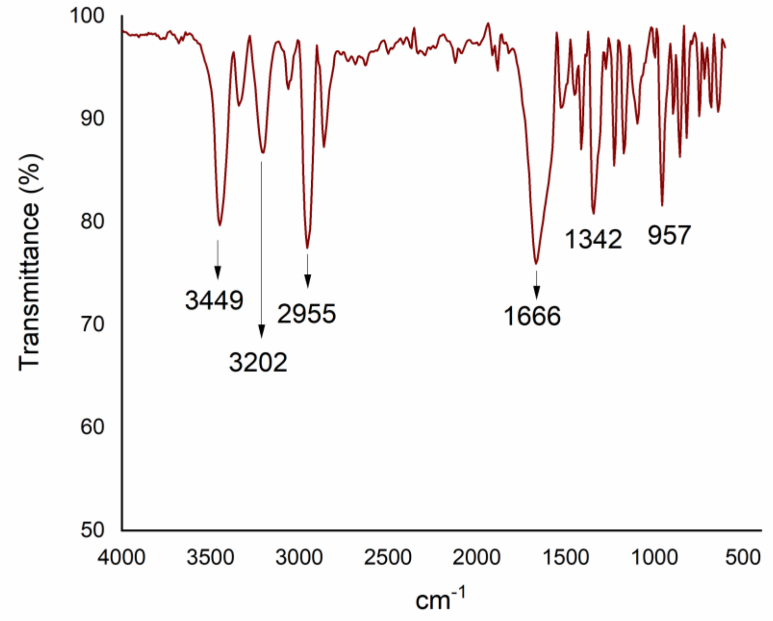

Figure S102. IR of 1-(4-amino-2-fluorophenyl)piperidin-2-one (**3a**).

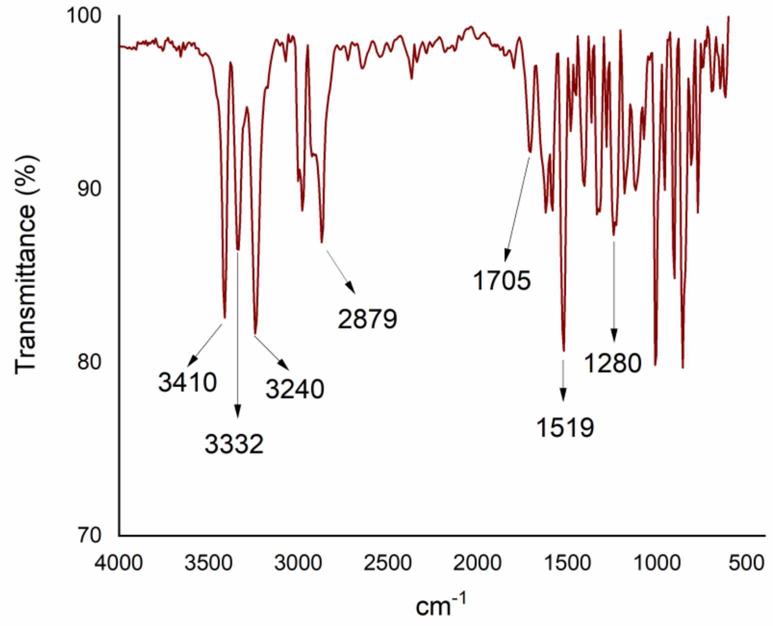

Figure S103. IR of *tert*-butyl 4-(4-amino-2-fluorophenyl)-3-oxopiperazine-1-carboxylate (**3b**).

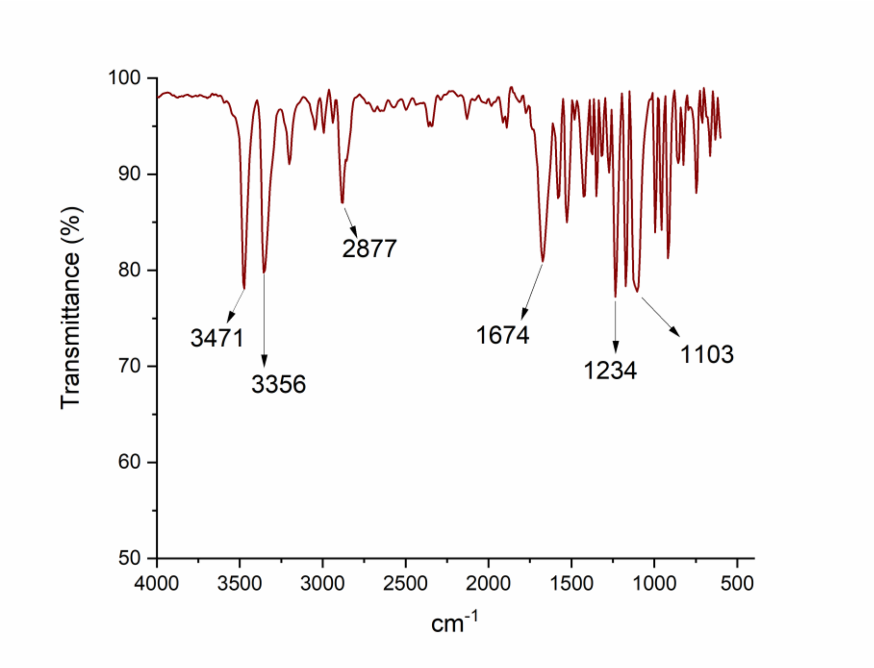

Figure S104. IR of 4-(4-amino-2-fluorophenyl)morpholin-3-one (**3c**).

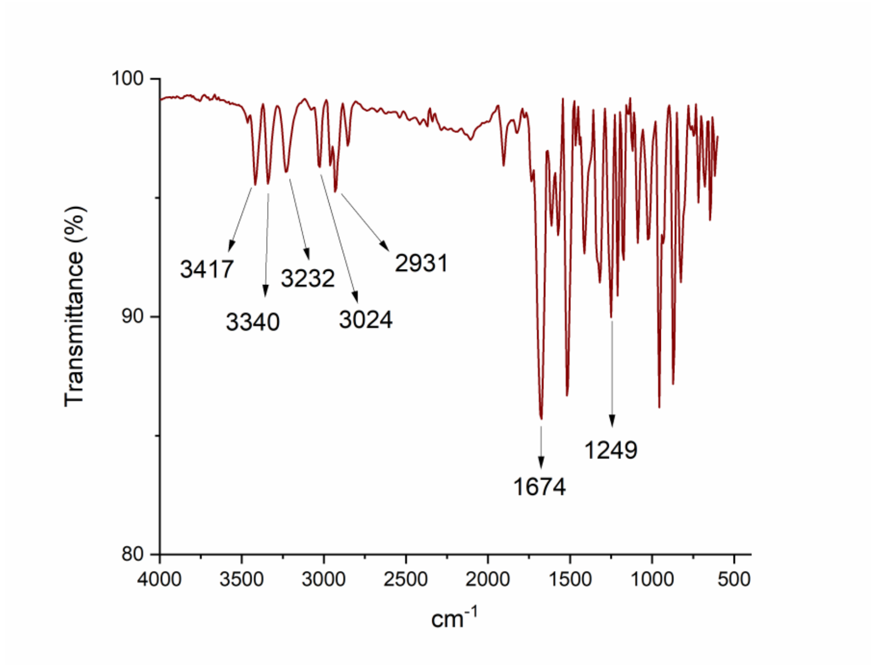

Figure S105. IR of 4-(4-amino-2-fluorophenyl)thiomorpholin-3-one (**3d**).

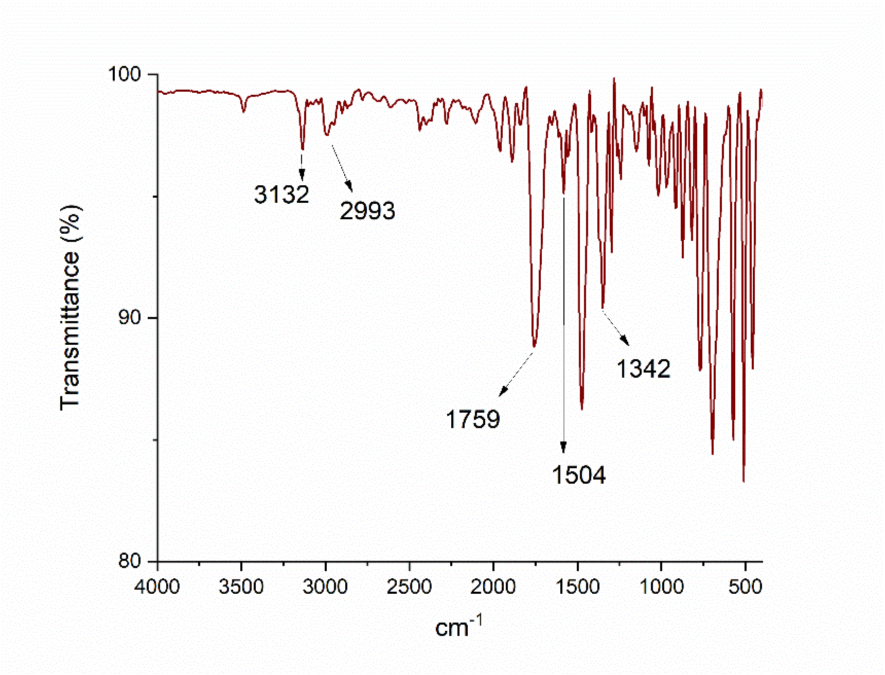

Figure S106. IR of Ethyl 2-(4-phenyl-1*H*-1,2,3-triazol-1-yl)acetate (**6a**).

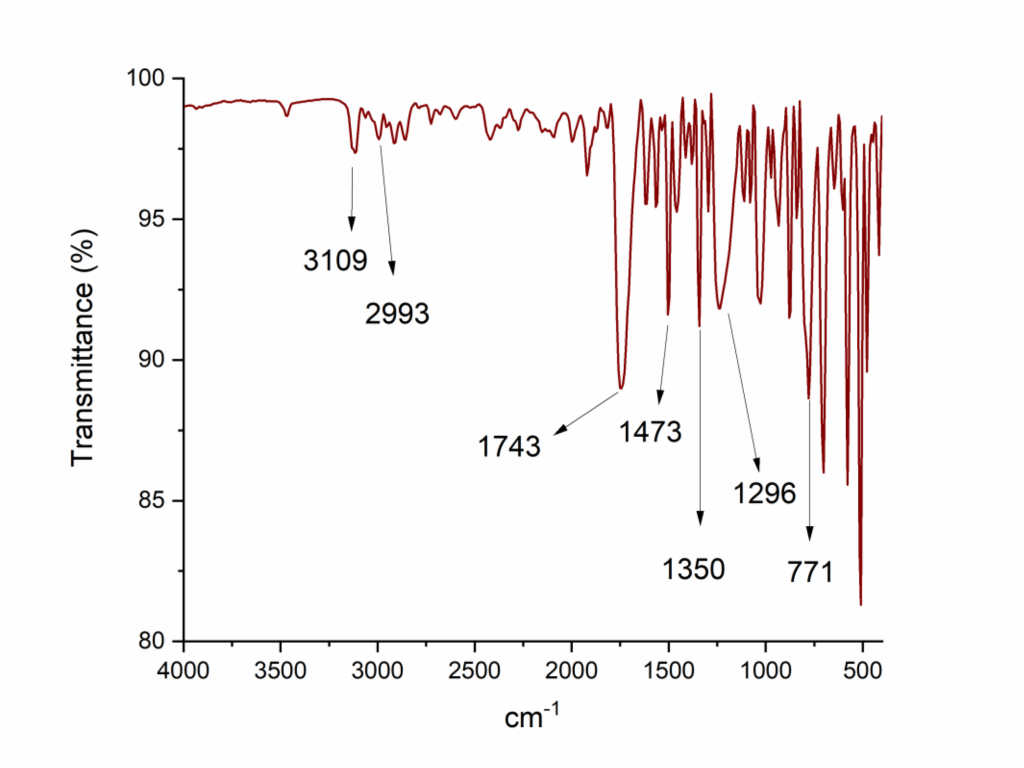

Figure S107. IR of Ethyl 2-(4-(*p*-tolyl)-1*H*-1,2,3-triazol-1-yl)acetate (**6b**).

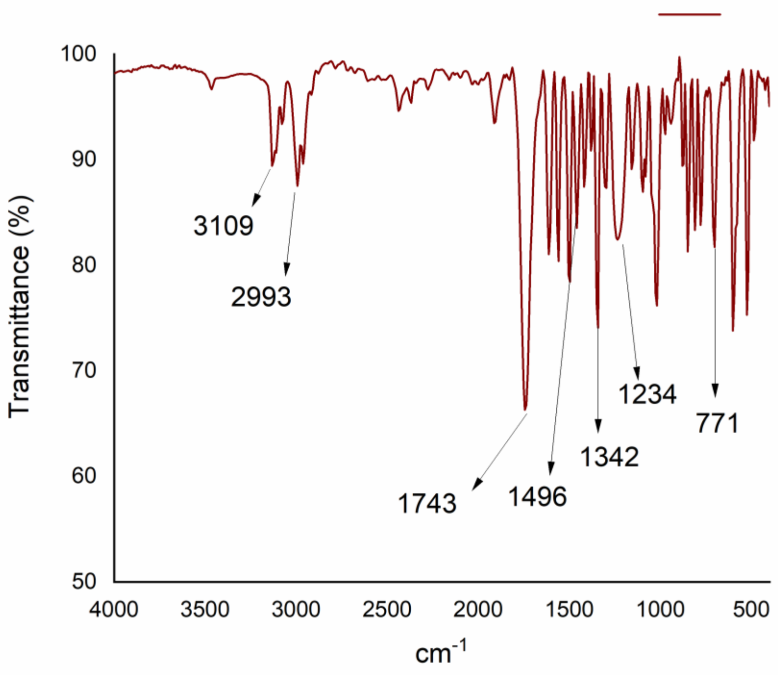

Figure S108. IR of Ethyl 2-(4-(4-fluorophenyl)-1*H*-1,2,3-triazol-1-yl)acetate (**6c**).

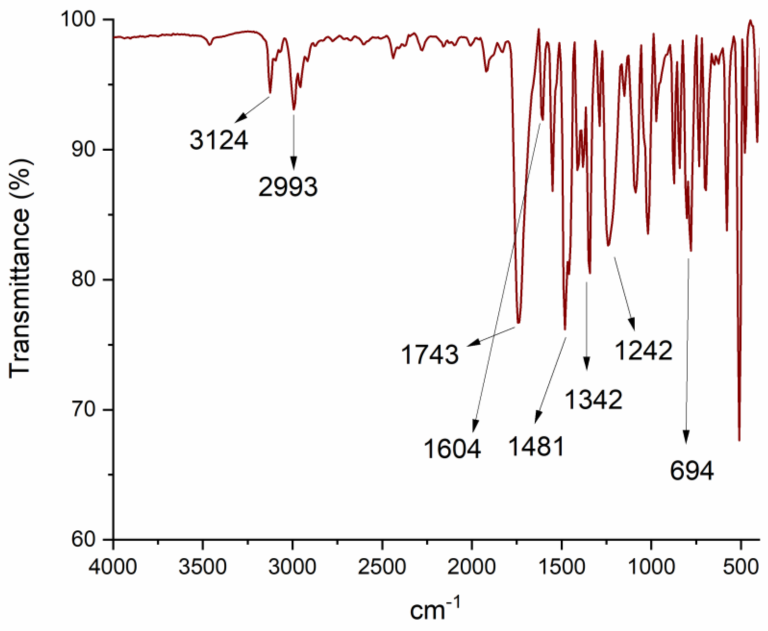

Figure S109. IR of Ethyl 2-(4-(4-chlorophenyl)-1*H*-1,2,3-triazol-1-yl)acetate (**6d**).

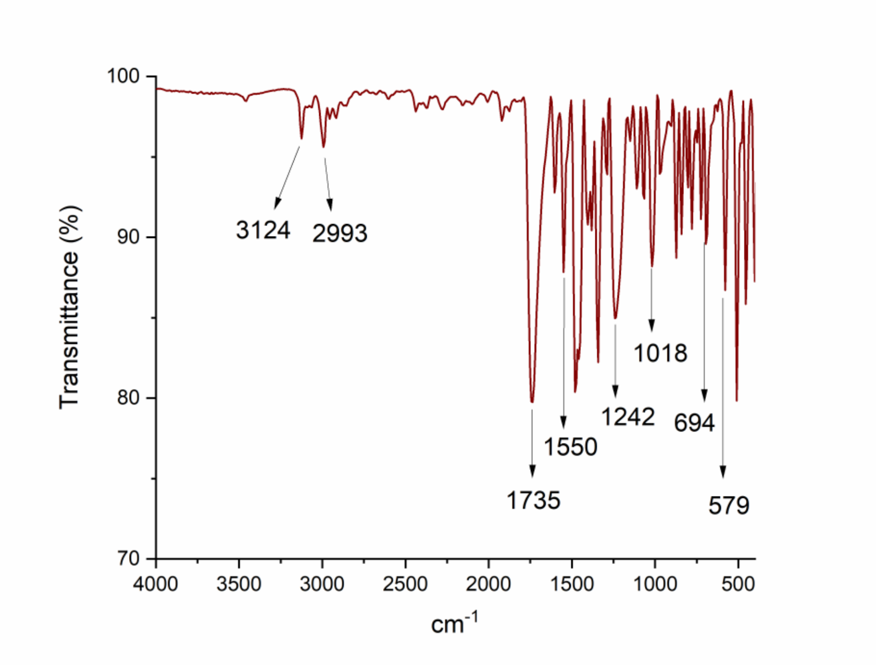

Figure S110. IR of Ethyl 2-(4-(4-bromophenyl)-1*H*-1,2,3-triazol-1-yl)acetate (**6e**).

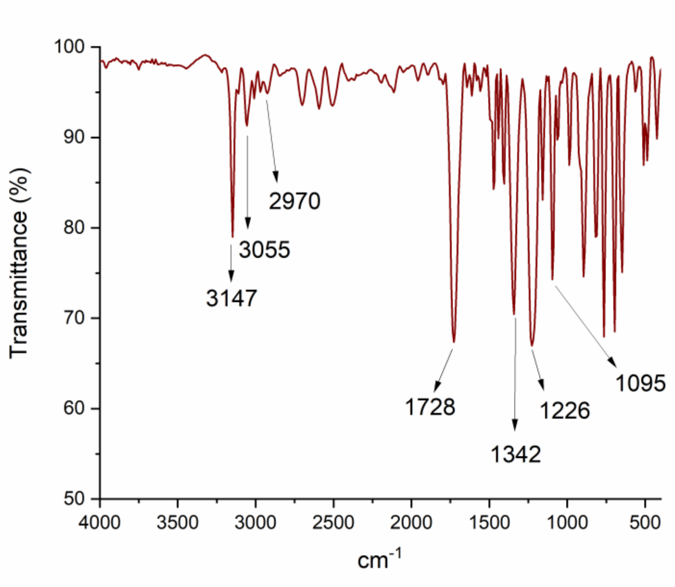

Figure S111. IR of 2-(4-phenyl-1*H*-1,2,3-triazol-1-yl)acetic acid (**7a**).

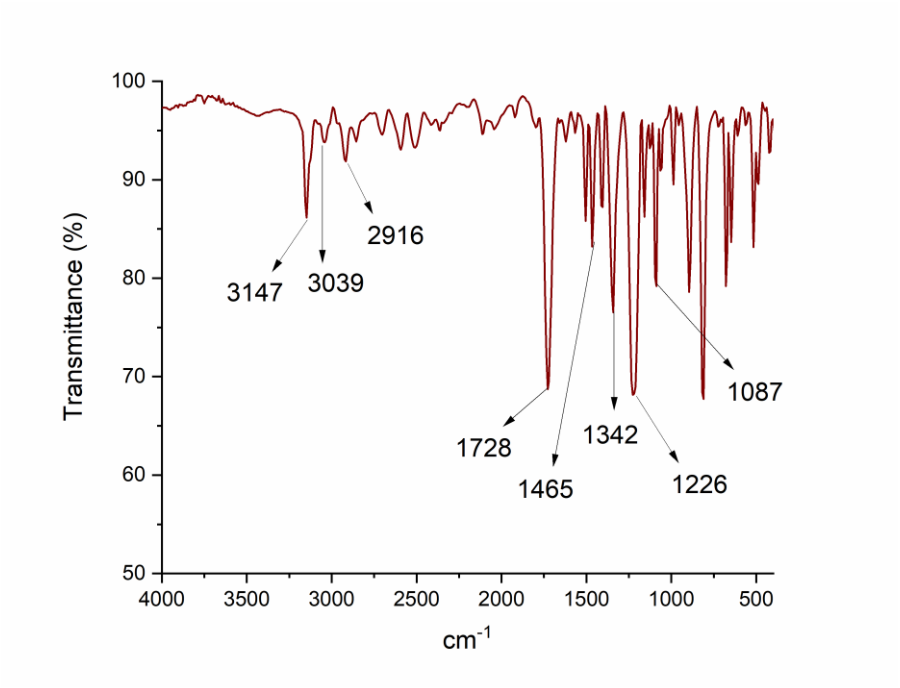

Figure S112. IR of 2-(4-(*p*-tolyl)-1*H*-1,2,3-triazol-1-yl)acetic acid (**7b**).

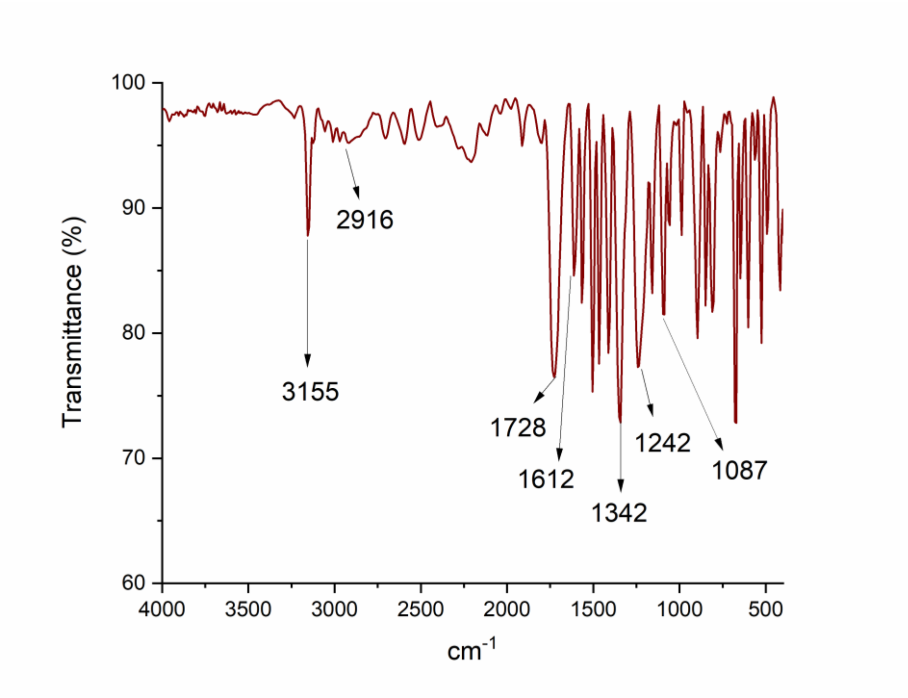

Figure S113. IR of 2-(4-(4-fluorophenyl)-1*H*-1,2,3-triazol-1-yl)acetic acid (**7c**).

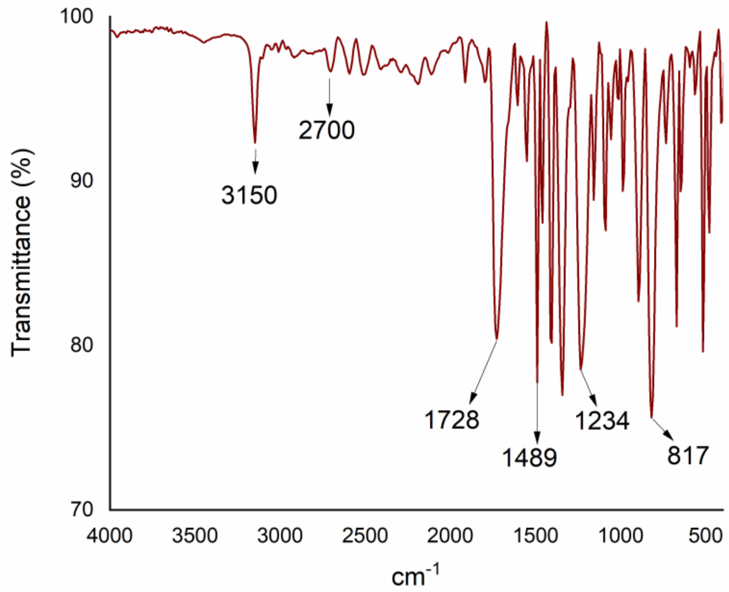

Figure S114. IR of 2-(4-(4-chlorophenyl)-1*H*-1,2,3-triazol-1-yl)acetic acid (**7d**).

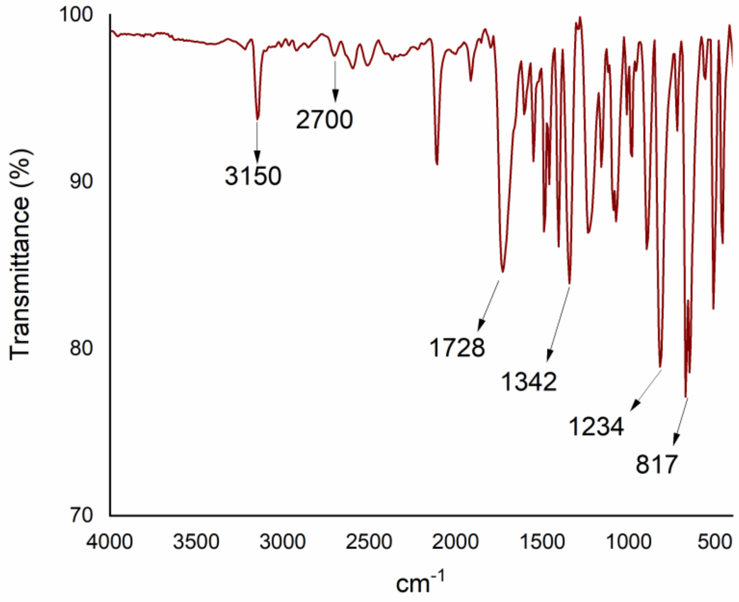

Figure S115. IR of 2-(4-(4-bromophenyl)-1*H*-1,2,3-triazol-1-yl)acetic acid (**7e**).

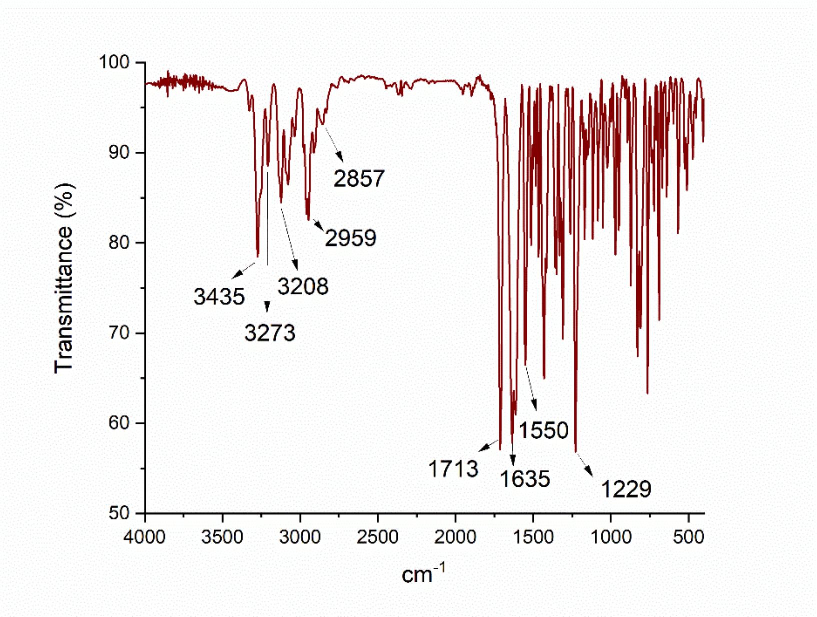

Figure S116. IR of *N*-(3-fluoro-4-(2-oxopiperidin-1-yl)phenyl)-2-(4-phenyl-1*H*-1,2,3-triazol-1-yl)acetamide (**8a**).

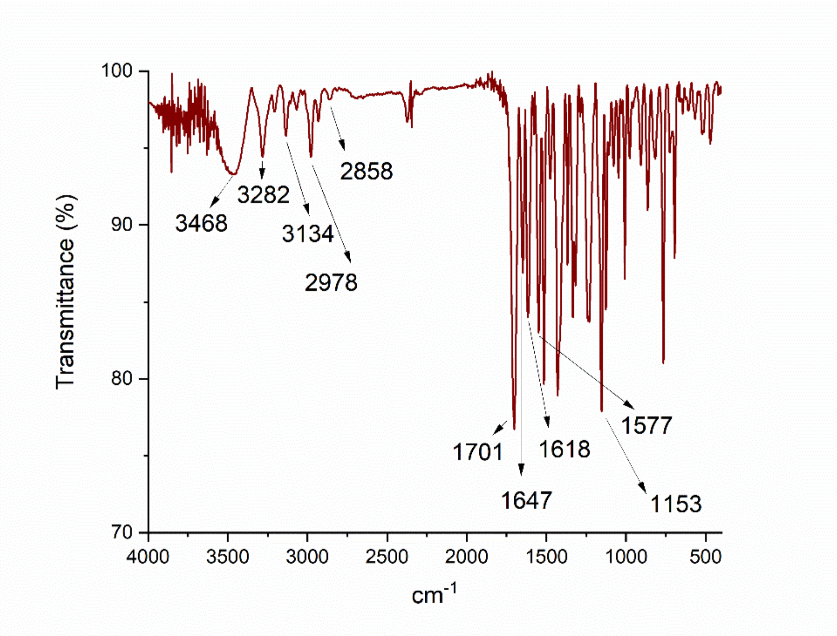

Figure S117. IR of *tert*-butyl 4-(2-fluoro-4-(2-(4-phenyl-1*H*-1,2,3-triazol-1-yl)acetamido)phenyl)-3-oxopiperazine-1-carboxylate (**8b**).

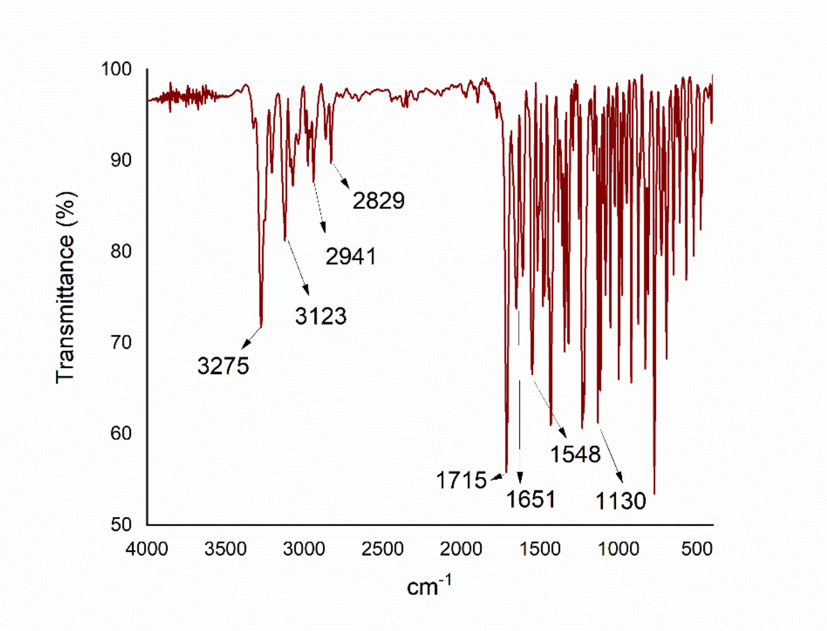

Figure S118. IR of *N*-(3-fluoro-4-(3-oxomorpholino)phenyl)-2-(4-phenyl-1*H*-1,2,3-triazol-1-yl)acetamide (**8c**).

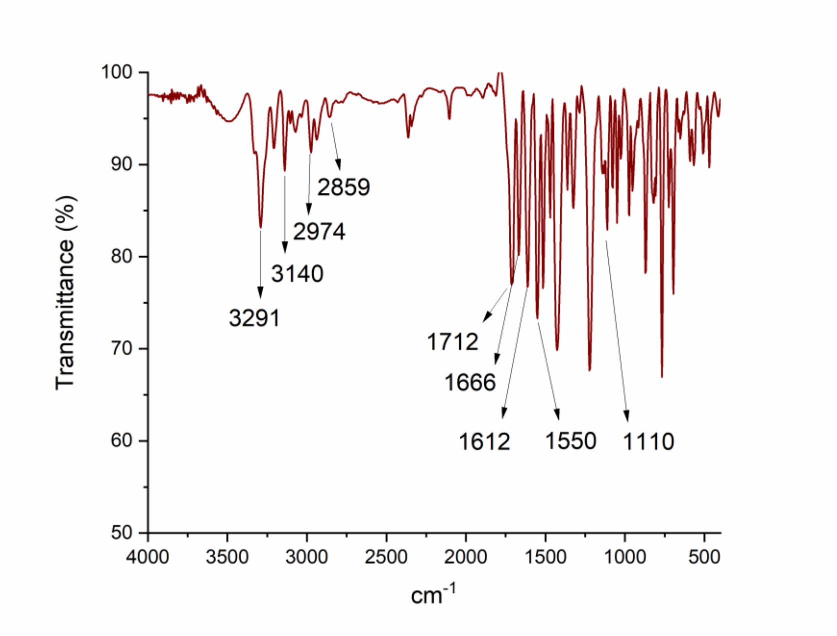

Figure S119. IR of *N*-(3-fluoro-4-(3-oxothiomorpholino)phenyl)-2-(4-phenyl-1*H*-1,2,3-triazol-1-yl)acetamide (**8d**).

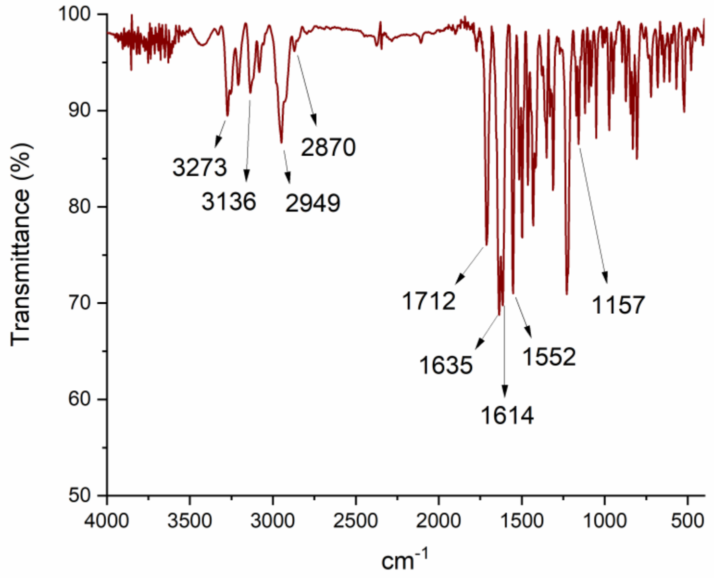

Figure S120. IR of *N*-(3-fluoro-4-(2-oxopiperidin-1-yl)phenyl)-2-(4-(4-fluorophenyl)-1*H*-1,2,3-triazol-1-yl)acetamide (**8e**).

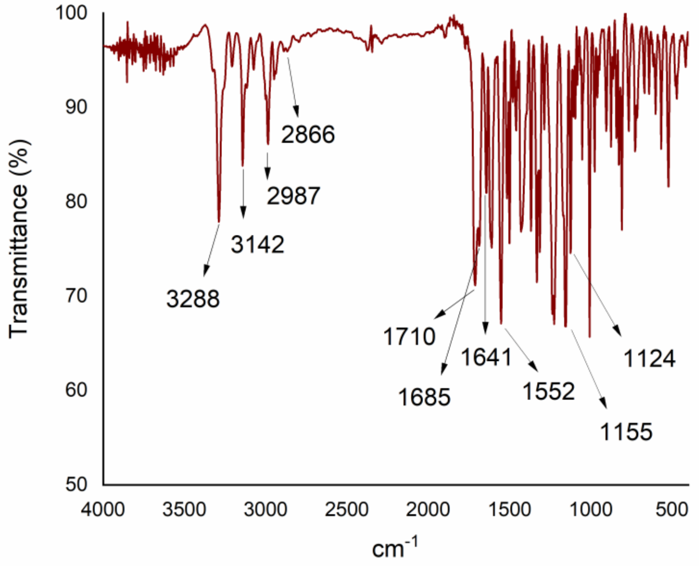

Figure S121. IR of *tert*-butyl 4-(2-fluoro-4-(2-(4-(4-fluorophenyl)-1*H*-1,2,3-triazol-1-yl)acetamido)phenyl)-3-oxopiperazine-1-carboxylate (**8f**).

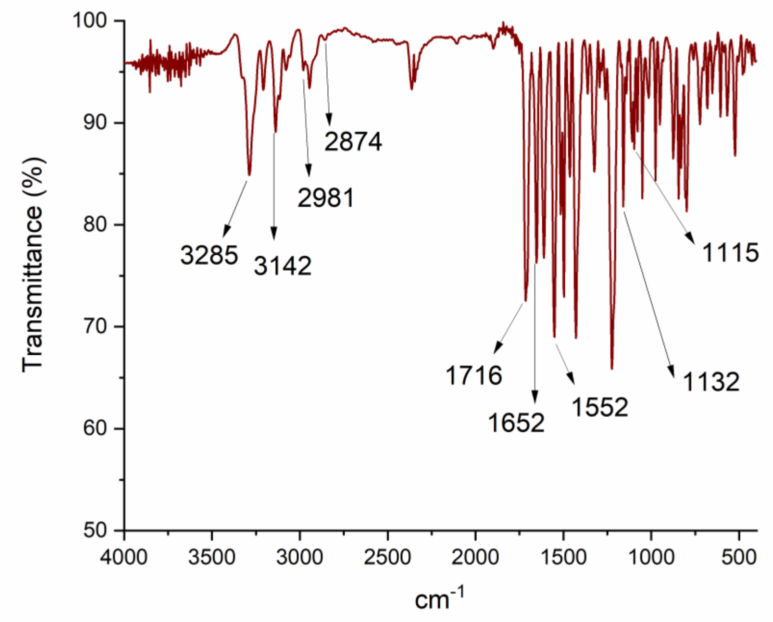

Figure S122. IR of *N*-(3-fluoro-4-(3-oxomorpholino)phenyl)-2-(4-(4-fluorophenyl)-1*H*-1,2,3-triazol-1-yl)acetamide (**8g**).

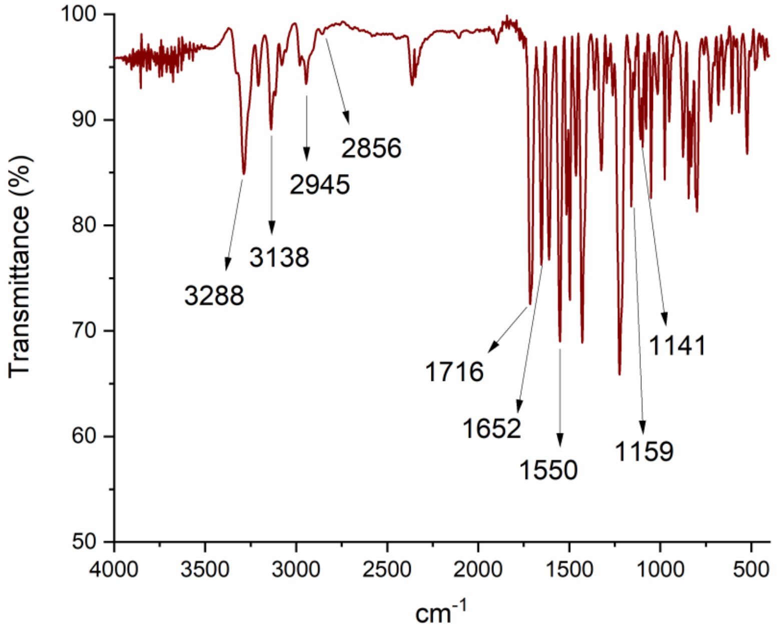

Figure S123. IR of *N*-(3-fluoro-4-(3-oxothiomorpholino)phenyl)-2-(4-(4-fluorophenyl)-1*H*-1,2,3-triazol-1-yl)acetamide (**8h**).

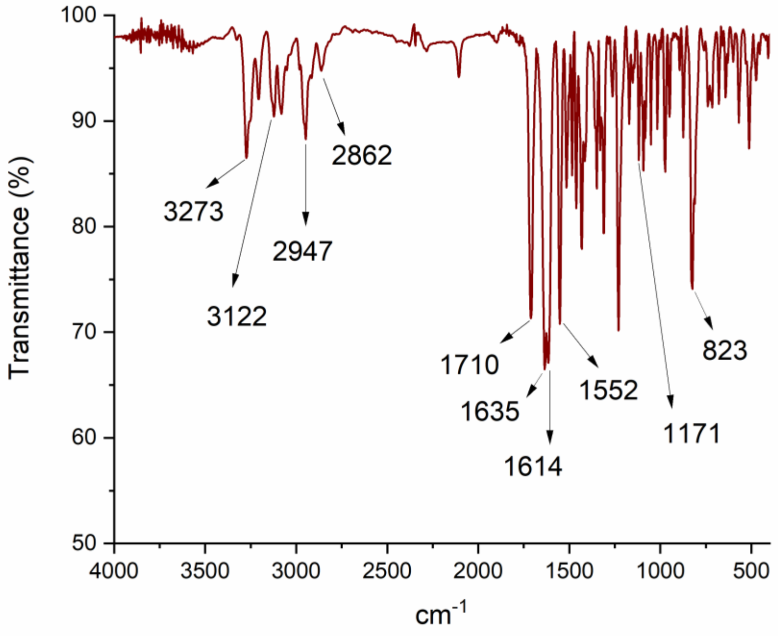

Figure S124. IR of 2-(4-(4-chlorophenyl)-1*H*-1,2,3-triazol-1-yl)-*N*-(3-fluoro-4-(2-oxopiperidin-1-yl)phenyl)acetamide (**8i**).

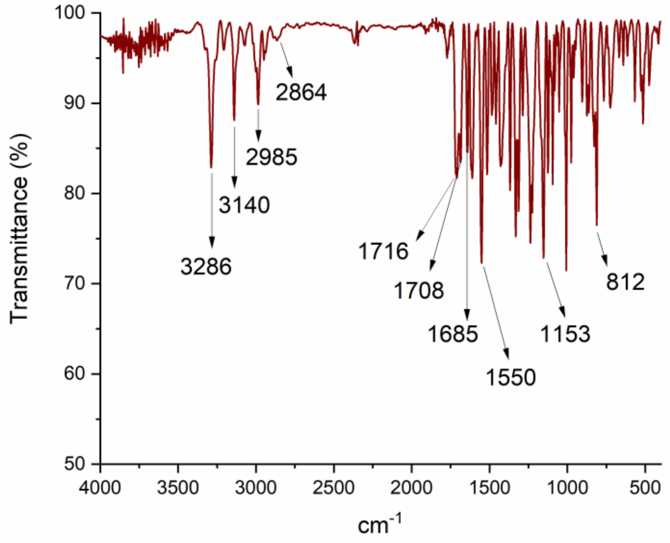

Figure S125. IR of *tert*-butyl 4-(4-(2-(4-(4-chlorophenyl)-1*H*-1,2,3-triazol-1-yl)acetamido)-2-fluorophenyl)-3-oxopiperazine-1-carboxylate (**8j**).

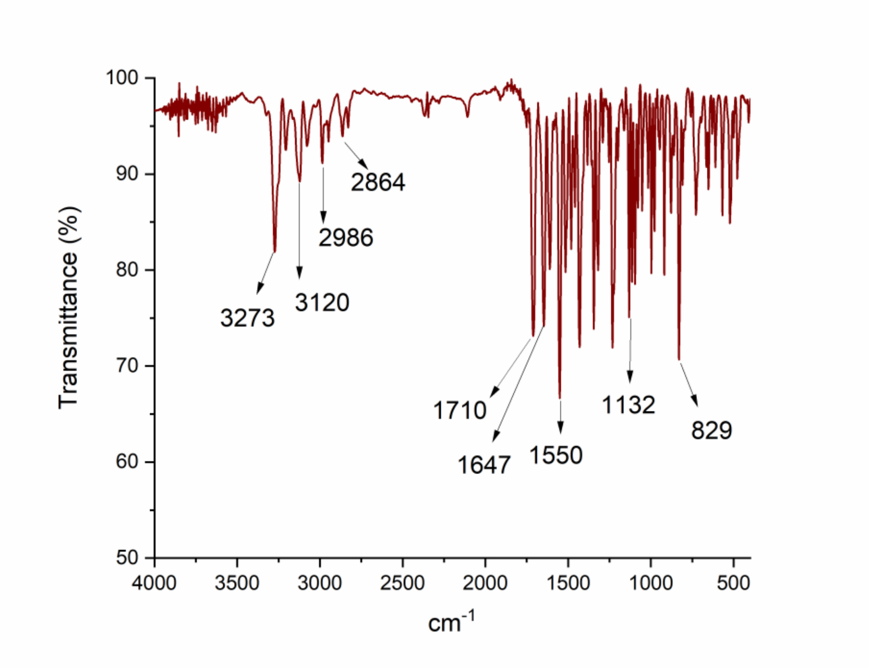

Figure S126. IR of 2-(4-(4-chlorophenyl)-1*H*-1,2,3-triazol-1-yl)-*N*-(3-fluoro-4-(3-oxomorpholino)phenyl)acetamide (**8k**).

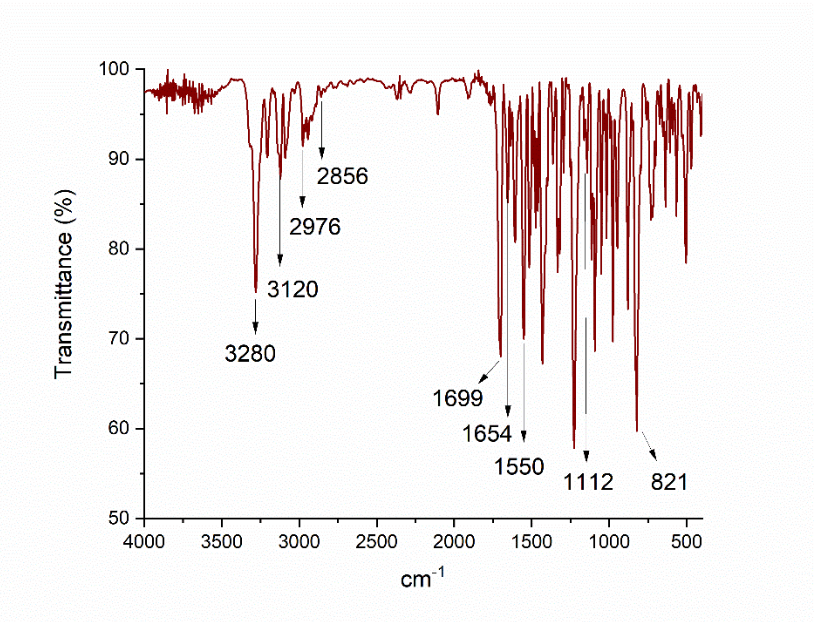

Figure S127. IR of 2-(4-(4-chlorophenyl)-1*H*-1,2,3-triazol-1-yl)-*N*-(3-fluoro-4-(3-oxothiomorpholino)phenyl)acetamide (**8l**).

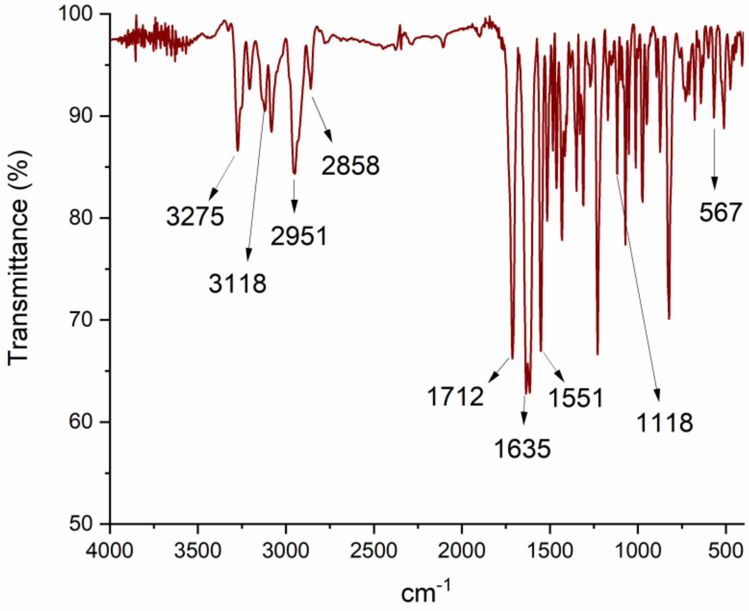

Figure S128. IR of 2-(4-(4-bromophenyl)-1*H*-1,2,3-triazol-1-yl)-*N*-(3-fluoro-4-(2-oxopiperidin-1-yl)phenyl)acetamide (**8m**).

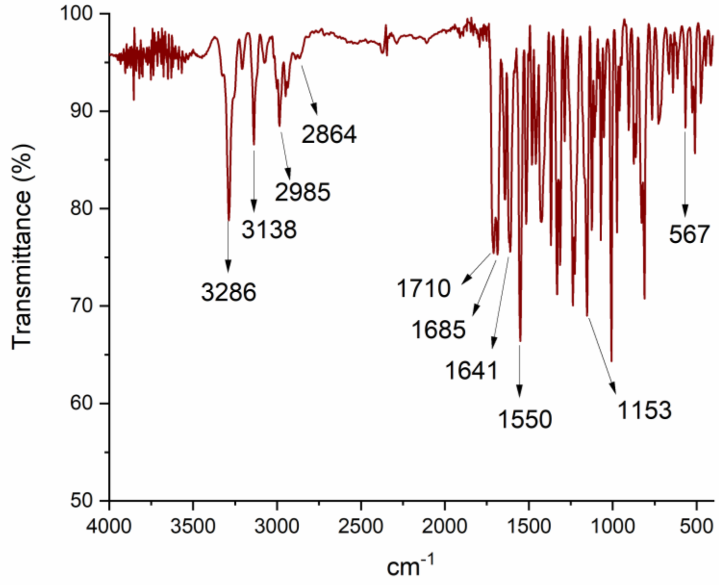

Figure S129. IR of 2-(4-(4-bromophenyl)-1*H*-1,2,3-triazol-1-yl)-*N*-(3-fluoro-4-(2-oxopiperidin-1-yl)phenyl)acetamide (**8n**).

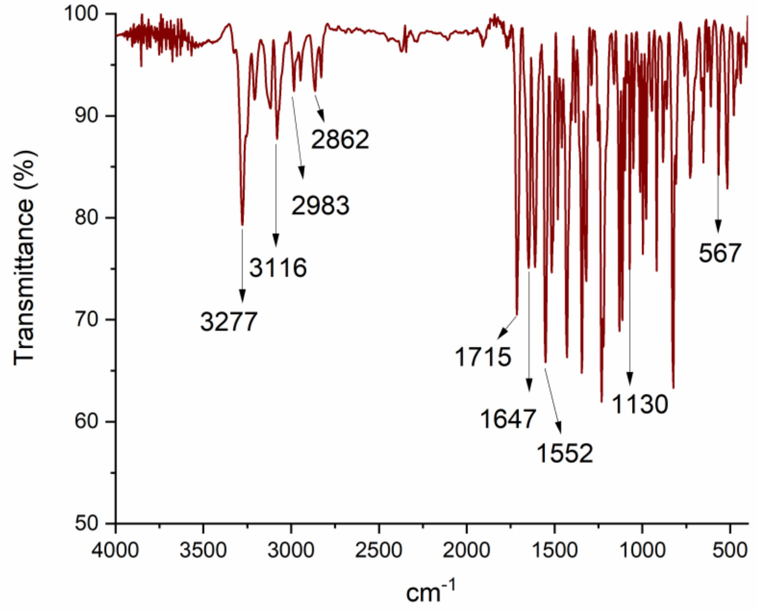

Figure S130. IR of 2-(4-(4-bromophenyl)-1*H*-1,2,3-triazol-1-yl)-*N*-(3-fluoro-4-(3-oxomorpholino)phenyl)acetamide (**8o**).

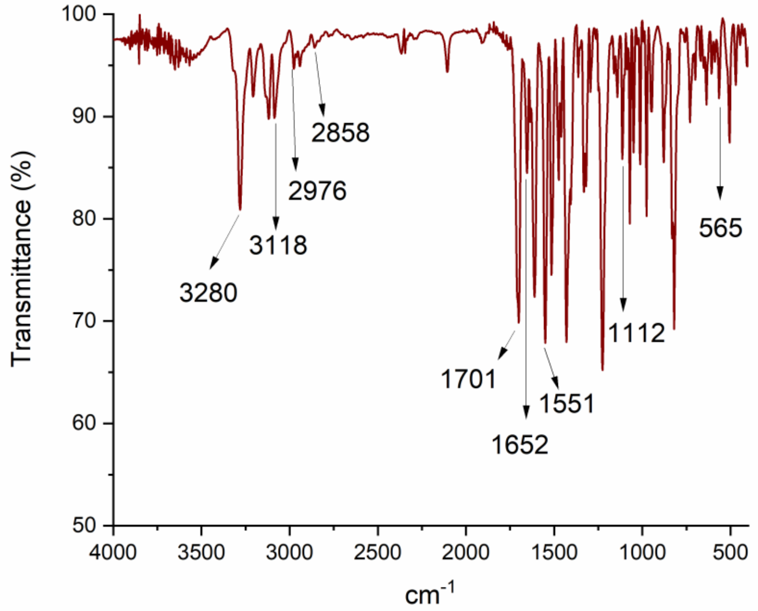

Figure S131. IR of 2-(4-(4-bromophenyl)-1*H*-1,2,3-triazol-1-yl)-*N*-(3-fluoro-4-(3-oxothiomorpholino)phenyl)acetamide (**8p**).

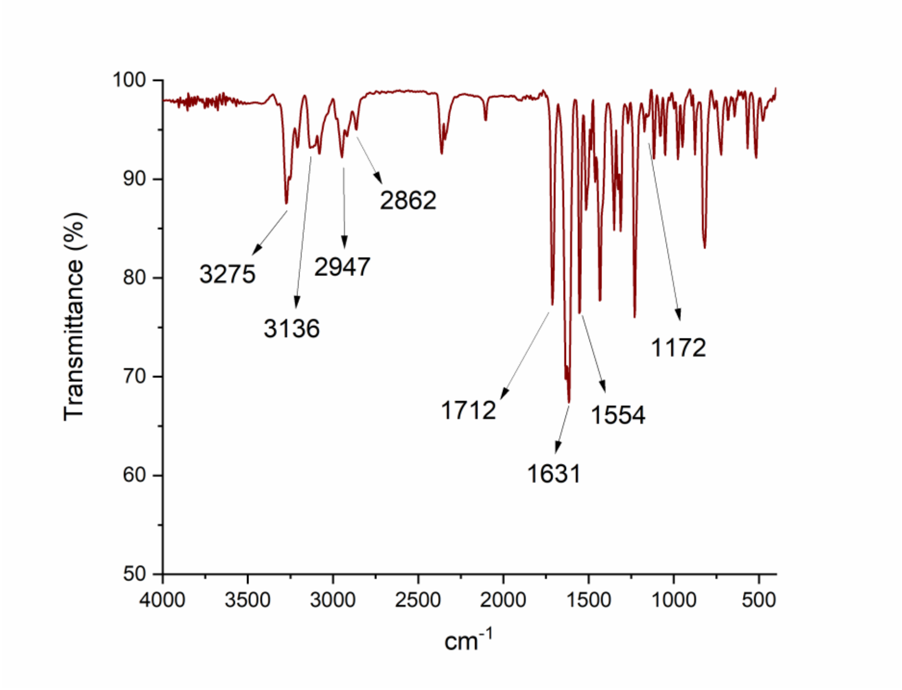

Figure S132. IR of *N*-(3-fluoro-4-(2-oxopiperidin-1-yl)phenyl)-2-(4-(*p*-tolyl)-1*H*-1,2,3-triazol-1-yl)acetamide(**8q**).

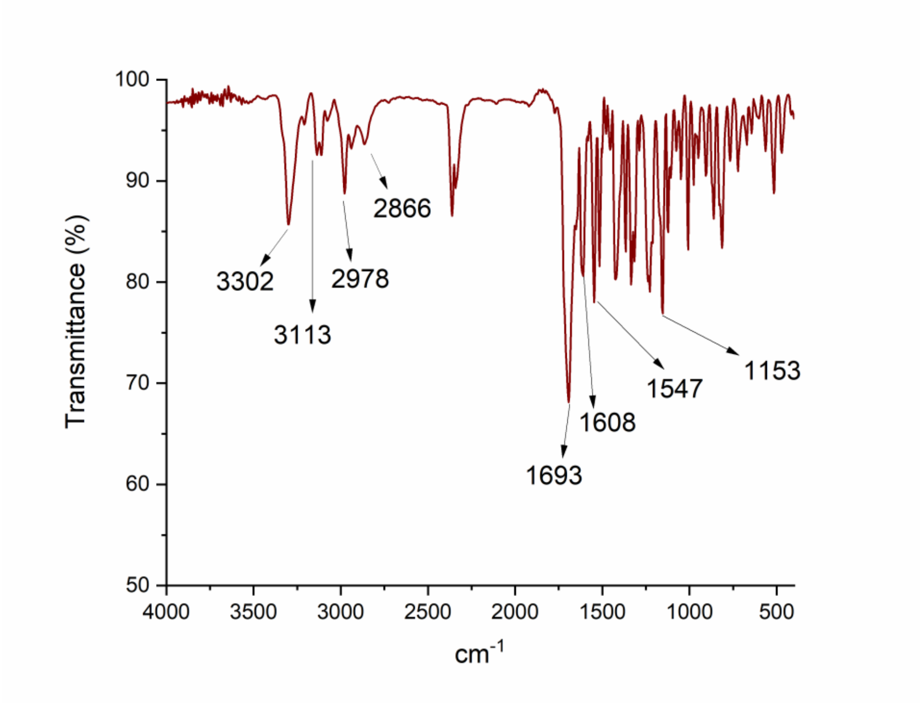

Figure S133. IR of *tert*-butyl4-(2-fluoro-4-(2-(4-(*p*-tolyl)-1*H*-1,2,3-triazol-1-yl)acetamido)phenyl)-3-oxopiperazine-1-carboxylate (**8r**).

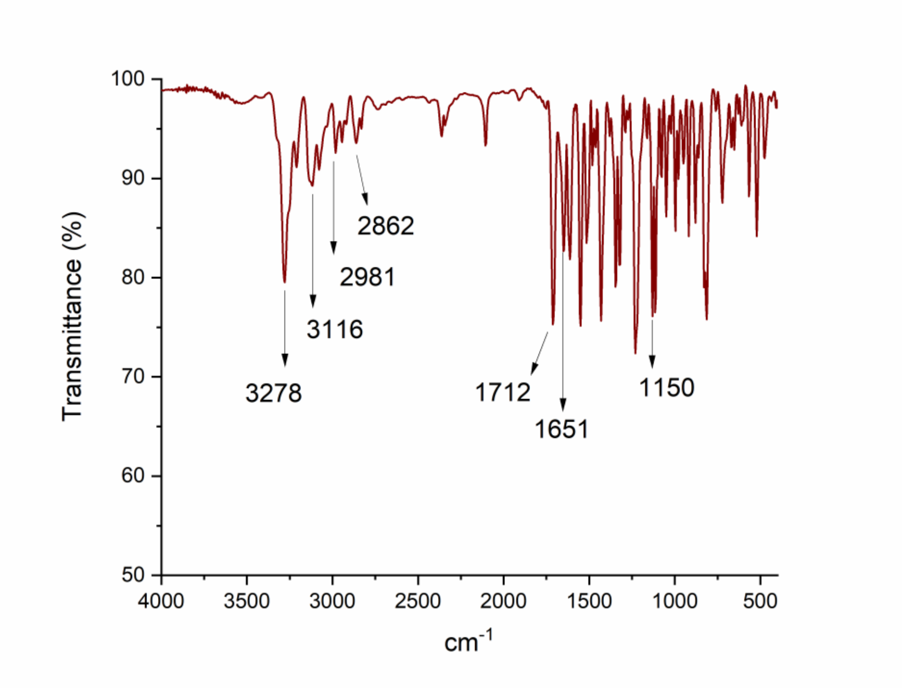

Figure S134. IR of *N*-(3-fluoro-4-(3-oxomorpholino)phenyl)-2-(4-(*p*-tolyl)-1*H*-1,2,3-triazol-1-yl)acetamide (**8s**).

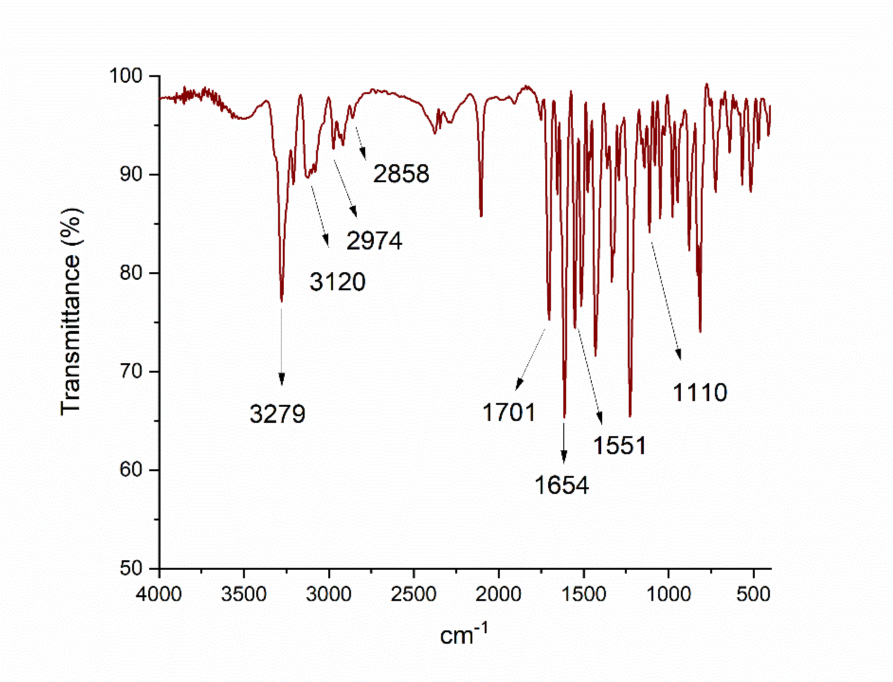

Figure S135. IR of *N*-(3-fluoro-4-(3-oxothiomorpholino)phenyl)-2-(4-(*p*-tolyl)-1*H*-1,2,3-triazol-1-yl)acetamide (**8t**).

11.4. *HRMS spectra*

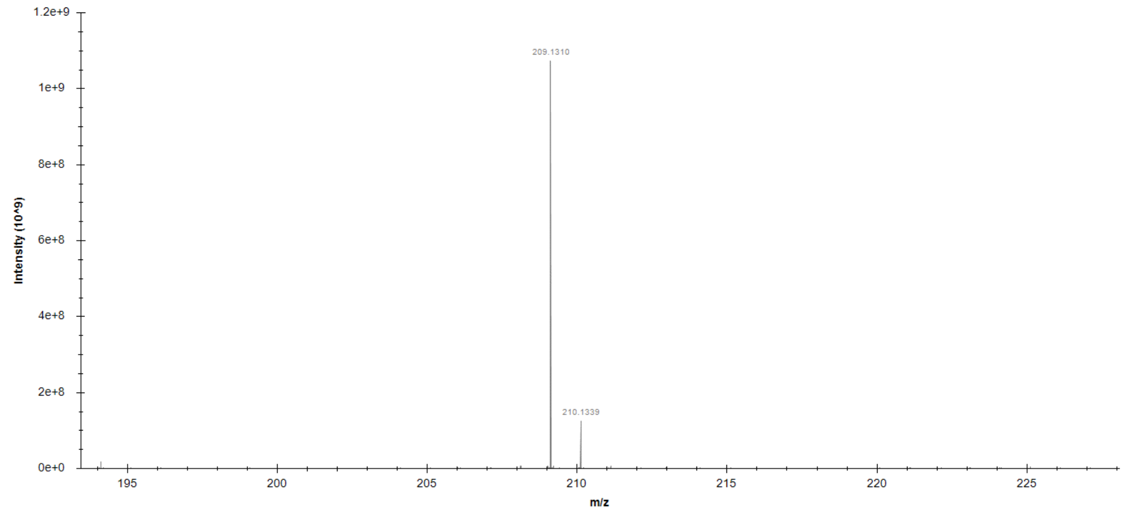

Figure S136. HRMS of 1-(4-amino-2-fluorophenyl)piperidin-2-one (**3a**)

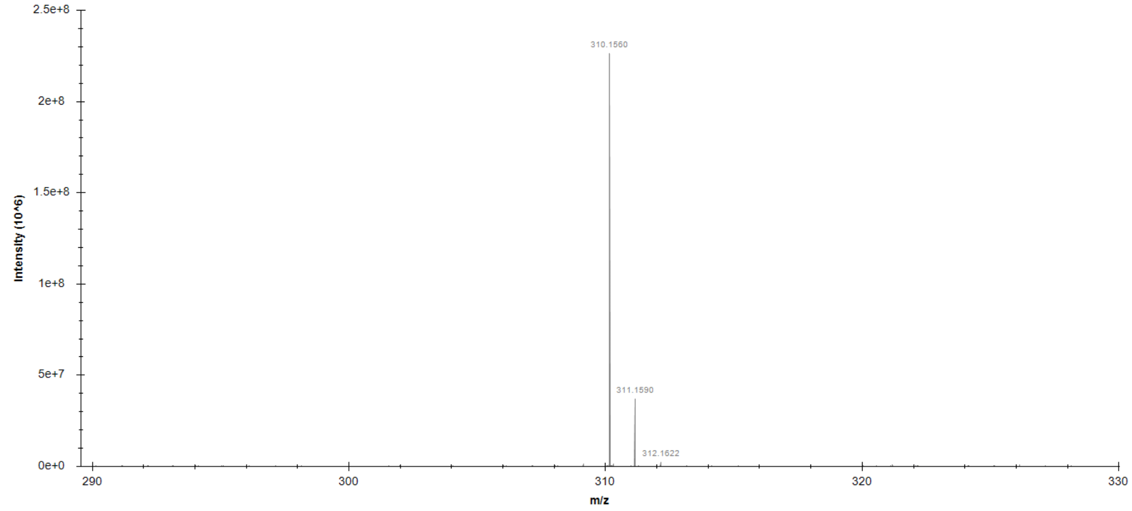

Figure S137. HRMS of *tert*-butyl 4-(4-amino-2-fluorophenyl)-3-oxopiperazine-1-carboxylate (**3b**)

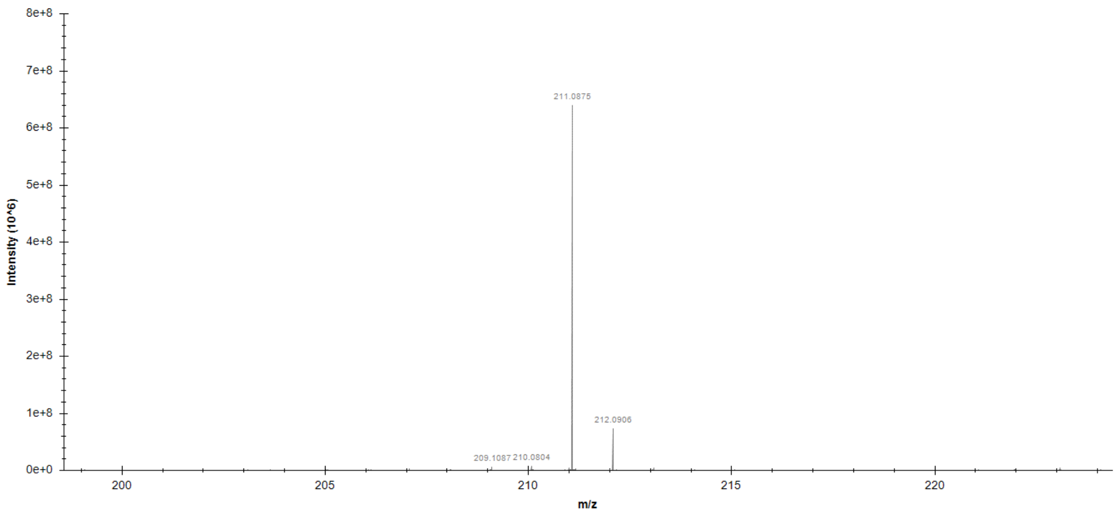

Figure S138. HRMS of 4-(4-amino-2-fluorophenyl)morpholin-3-one (**3c**)

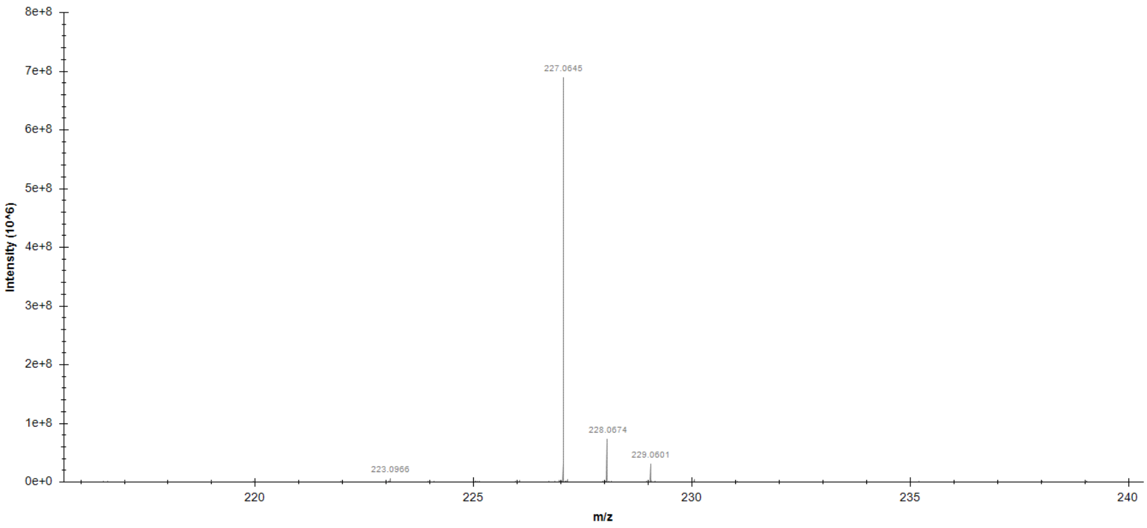

Figure S139. HRMS 4-(4-amino-2-fluorophenyl)thiomorpholin-3-one (**3d**).

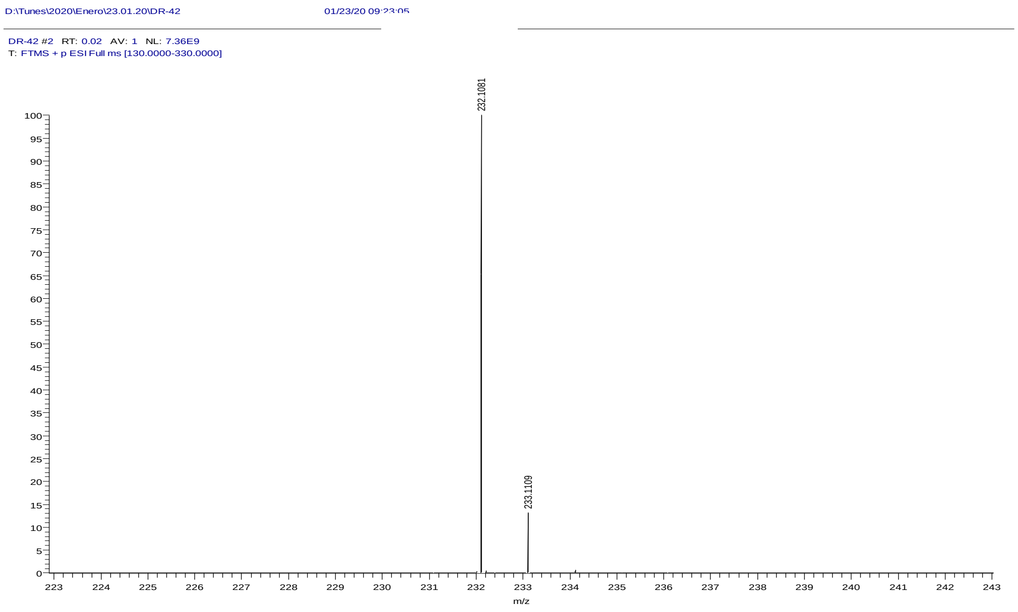

Figure S140. HRMS ethyl 2-(4-phenyl-1*H*-1,2,3-triazol-1-yl)acetate (**6a**).

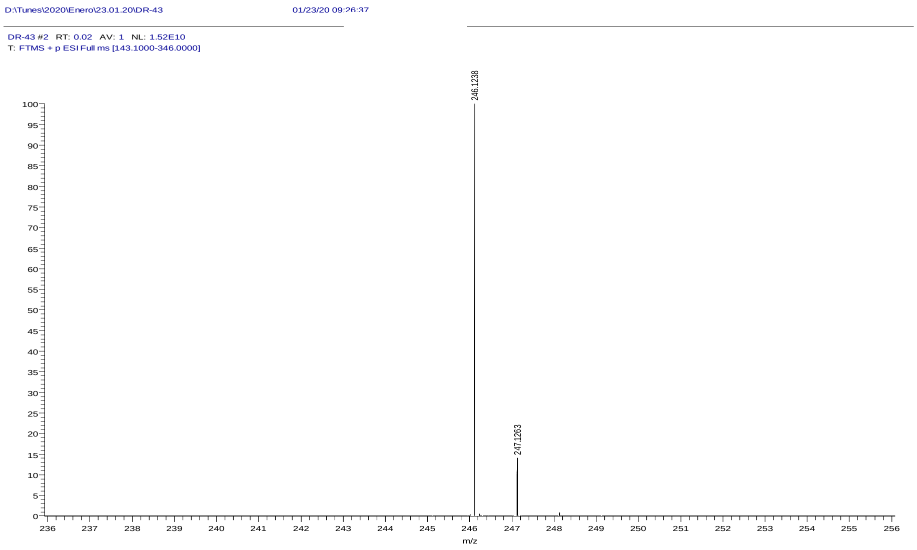

Figure S141. HRMS ethyl 2-(4-(*p*-tolyl)-1*H*-1,2,3-triazol-1-yl)acetate (**6b).**

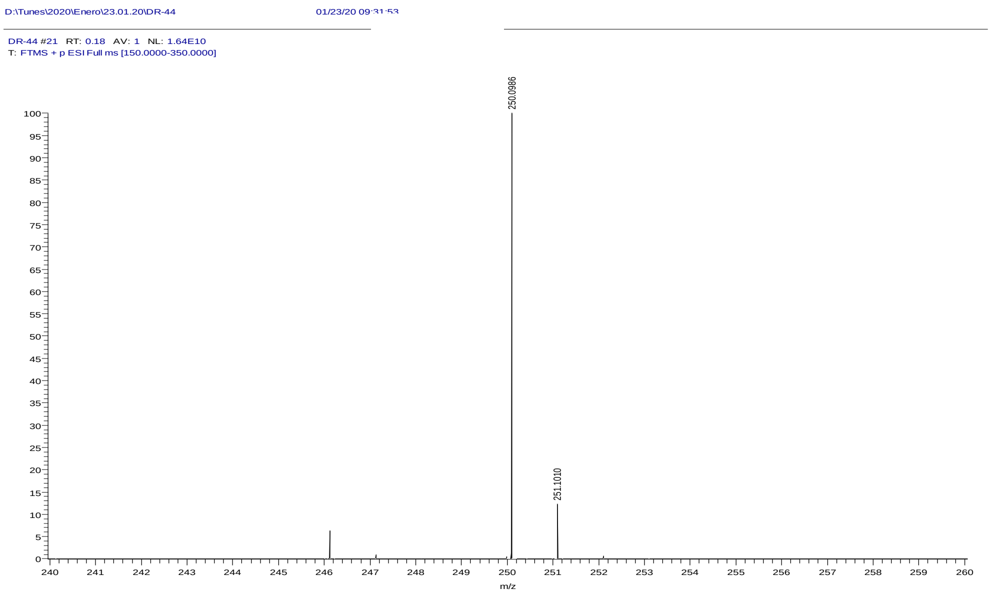

Figure S142. HRMS 2-(4-(4-fluorophenyl)-1*H*-1,2,3-triazol-1-yl)acetate (**6c**).

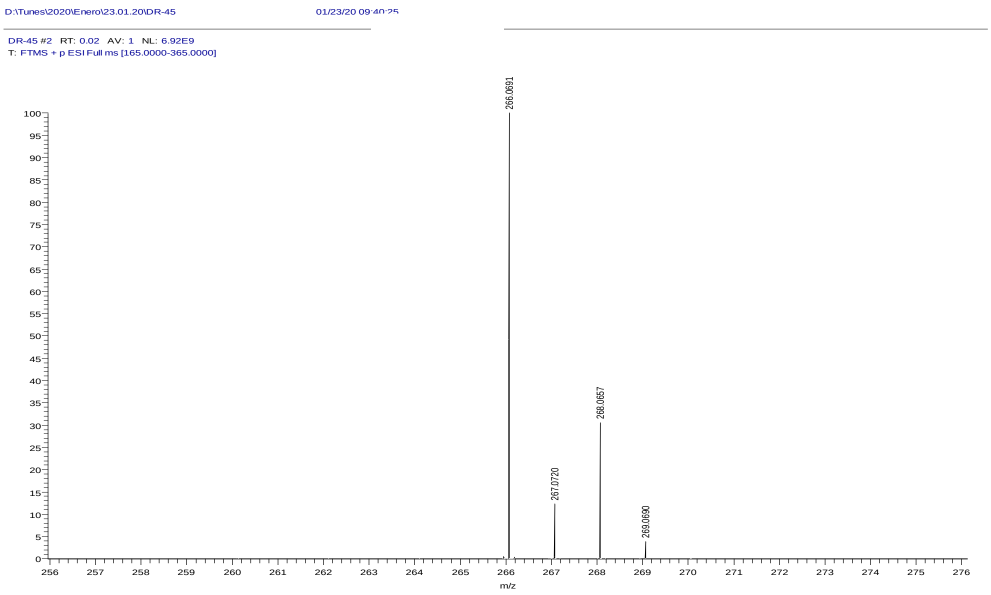

Figure S143. HRMS ethyl 2-(4-(4-chlorophenyl)-1*H*-1,2,3-triazol-1-yl)acetate (**6d**).

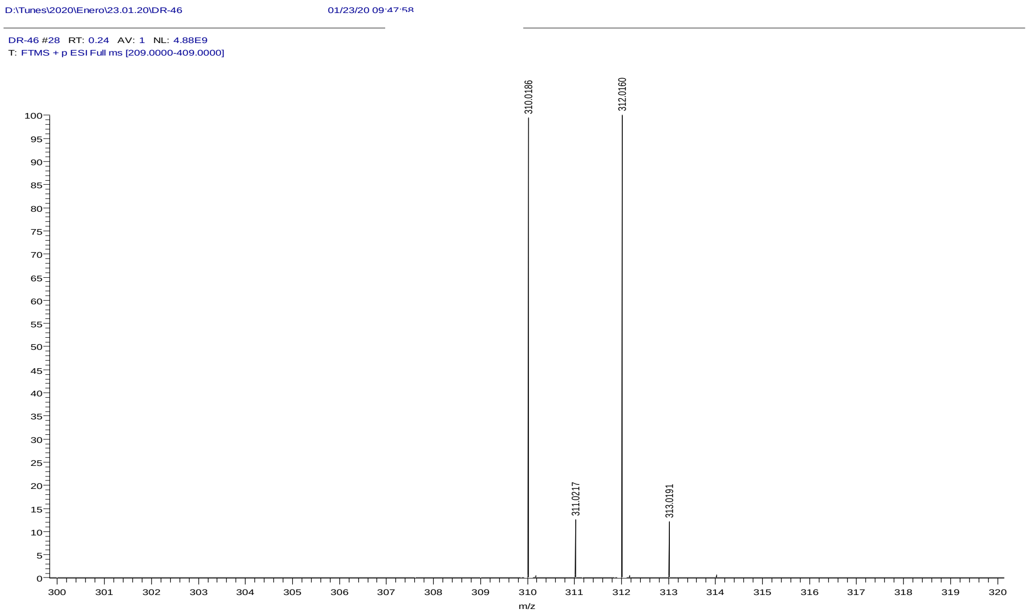

Figure S144. HRMS ethyl 2-(4-(4-bromophenyl)-1*H*-1,2,3-triazol-1-yl)acetate (**6e**).

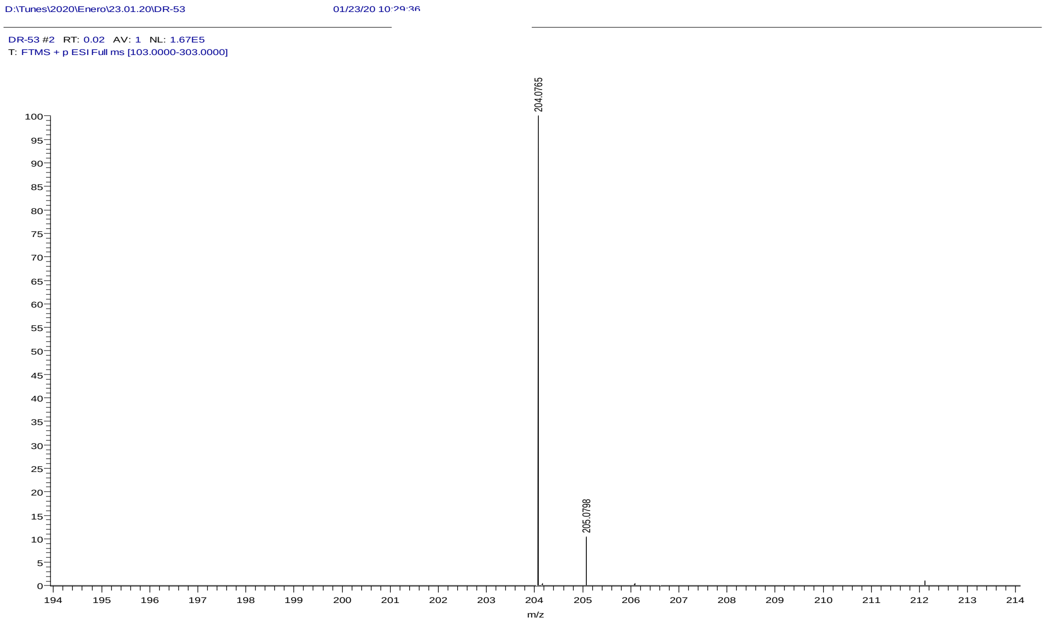

Figure S145. HRMS 2-(4-phenyl-1*H*-1,2,3-triazol-1-yl)acetic acid (**7a**).

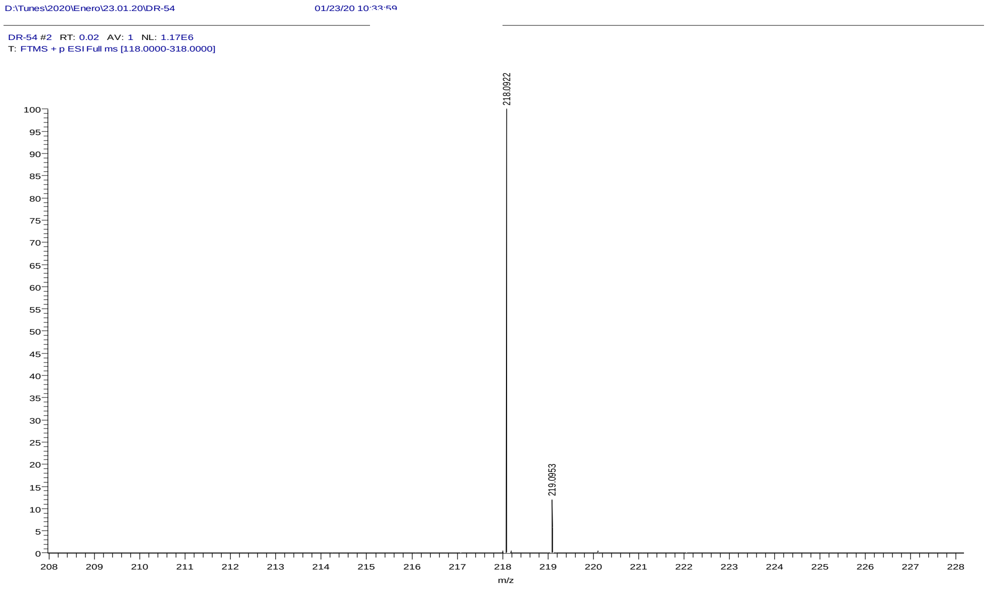

Figure S146. HRMS 2-(4-(*p*-tolyl)-1*H*-1,2,3-triazol-1-yl)acetic acid (**7b**).

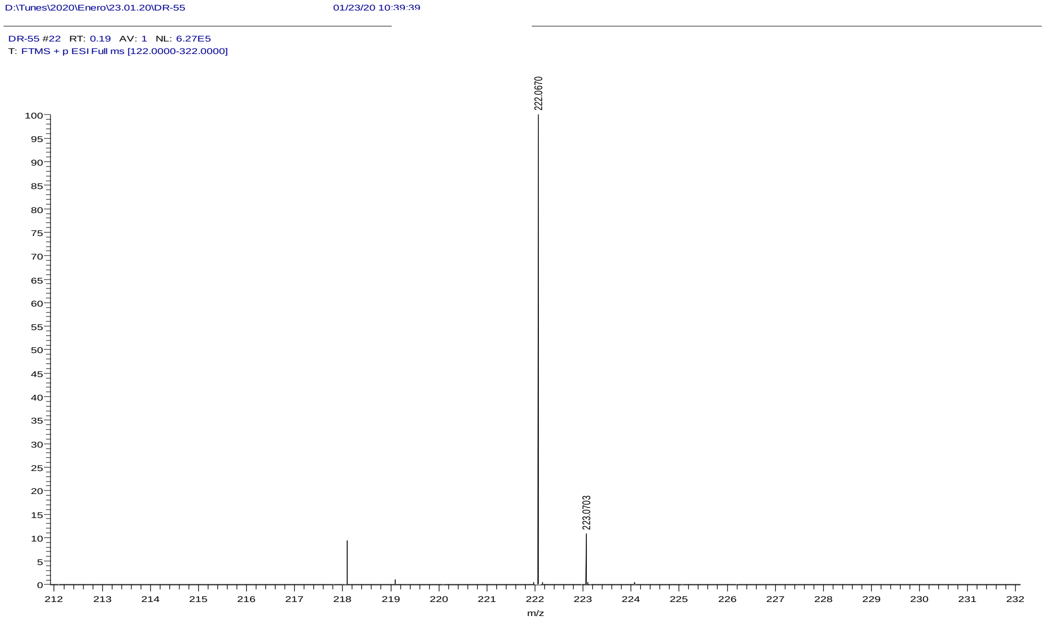

Figure S147. HRMS 2-(4-(4-fluorophenyl)-1*H*-1,2,3-triazol-1-yl)acetic acid (**7c**).

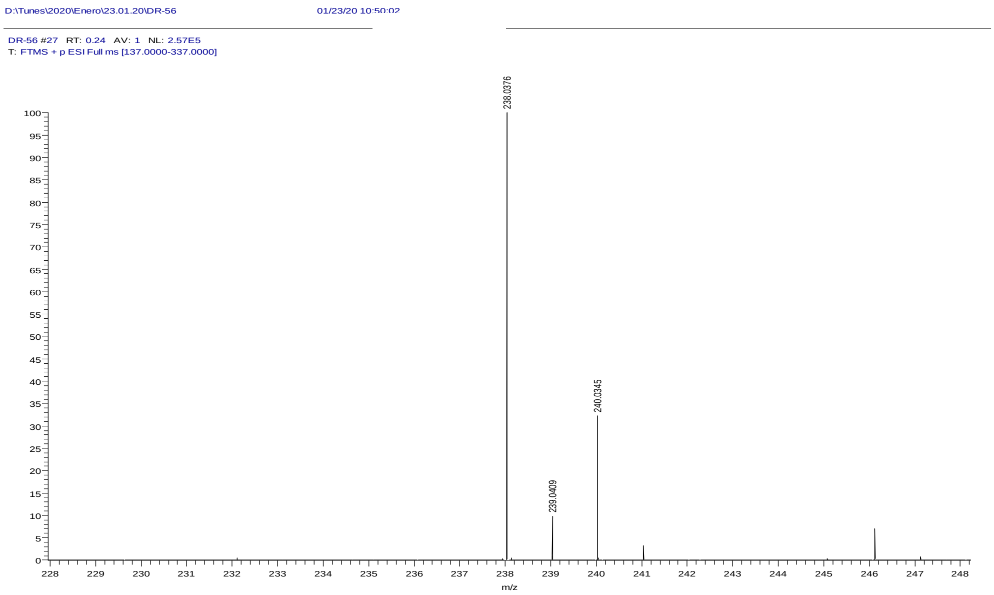

Figure S148. HRMS 2-(4-(4-chlorophenyl)-1*H*-1,2,3-triazol-1-yl)acetic acid (**7d**).

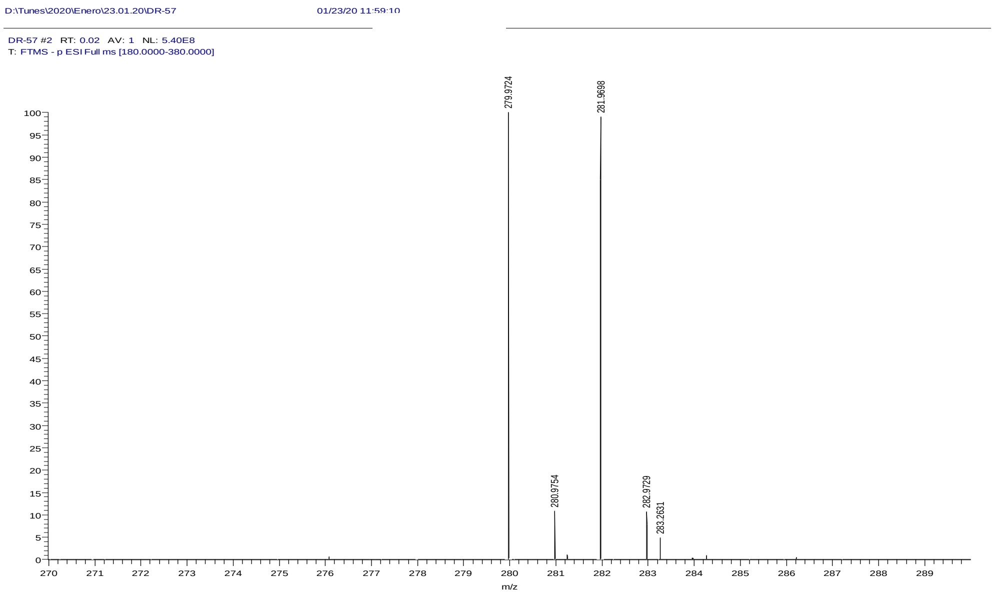

Figure S149. HRMS 2-(4-(4-bromophenyl)-1*H*-1,2,3-triazol-1-yl)acetic acid (**7e**).

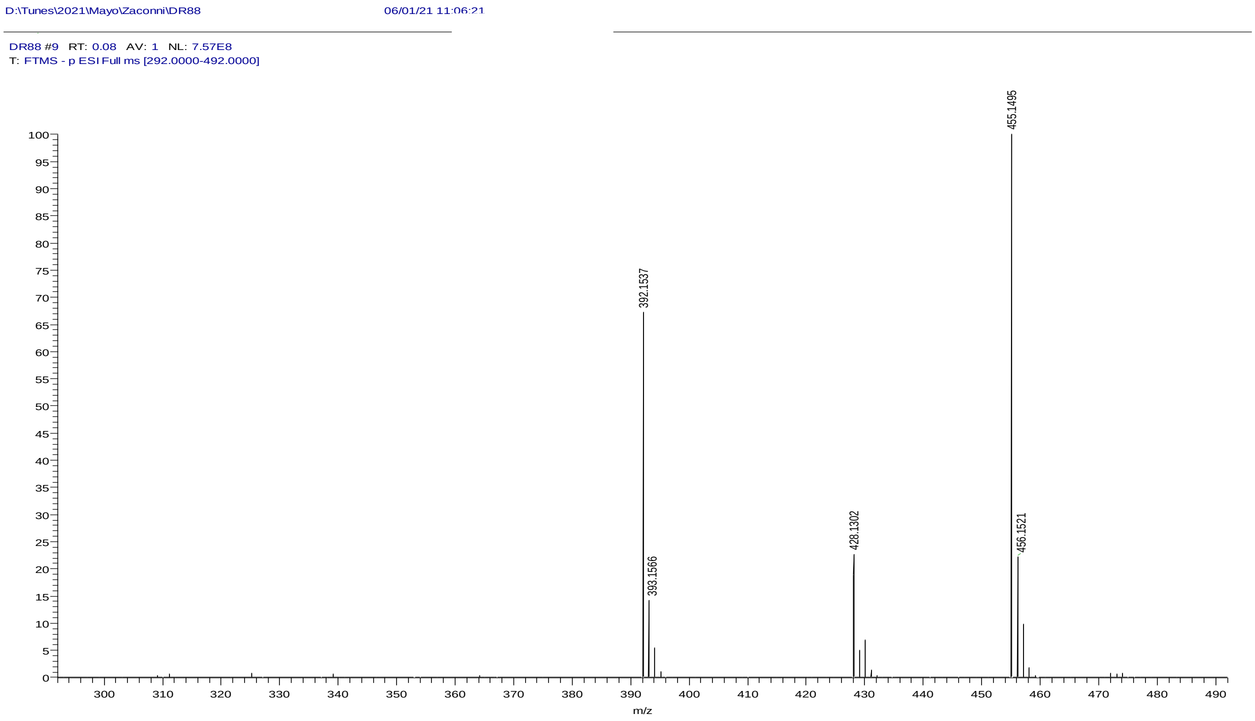

Figure S150. IR *N*-(3-fluoro-4-(2-oxopiperidin-1-yl)phenyl)-2-(4-phenyl-1*H*-1,2,3-triazol-1-yl)acetamide (**8a**).

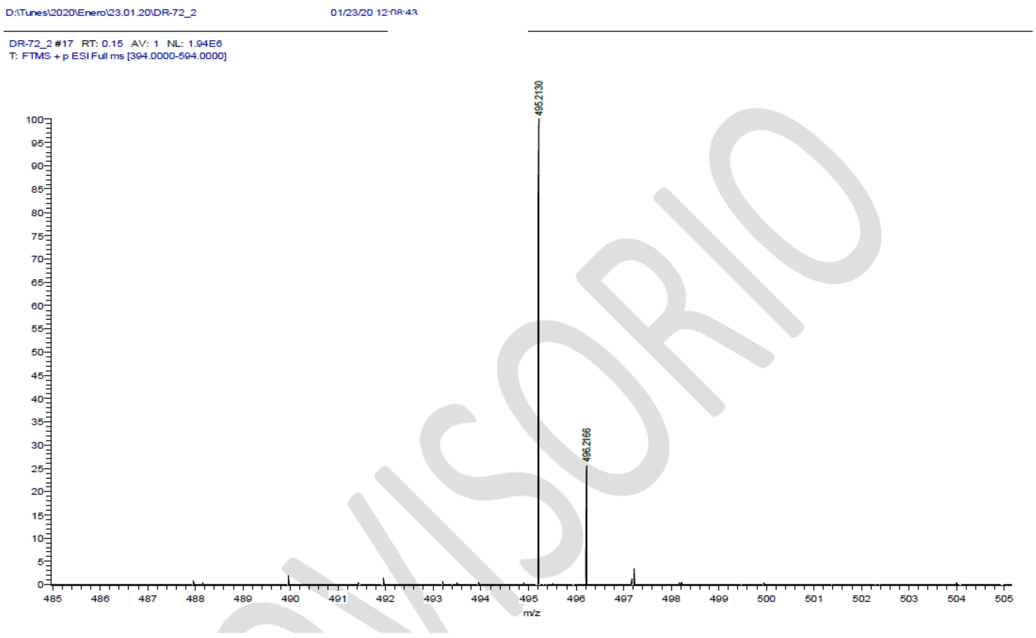

Figure S151. IR *tert*-butyl 4-(2-fluoro-4-(2-(4-phenyl-1*H*-1,2,3-triazol-1-yl)acetamido)phenyl)-3-oxopiperazine-1-carboxylate (**8b**).

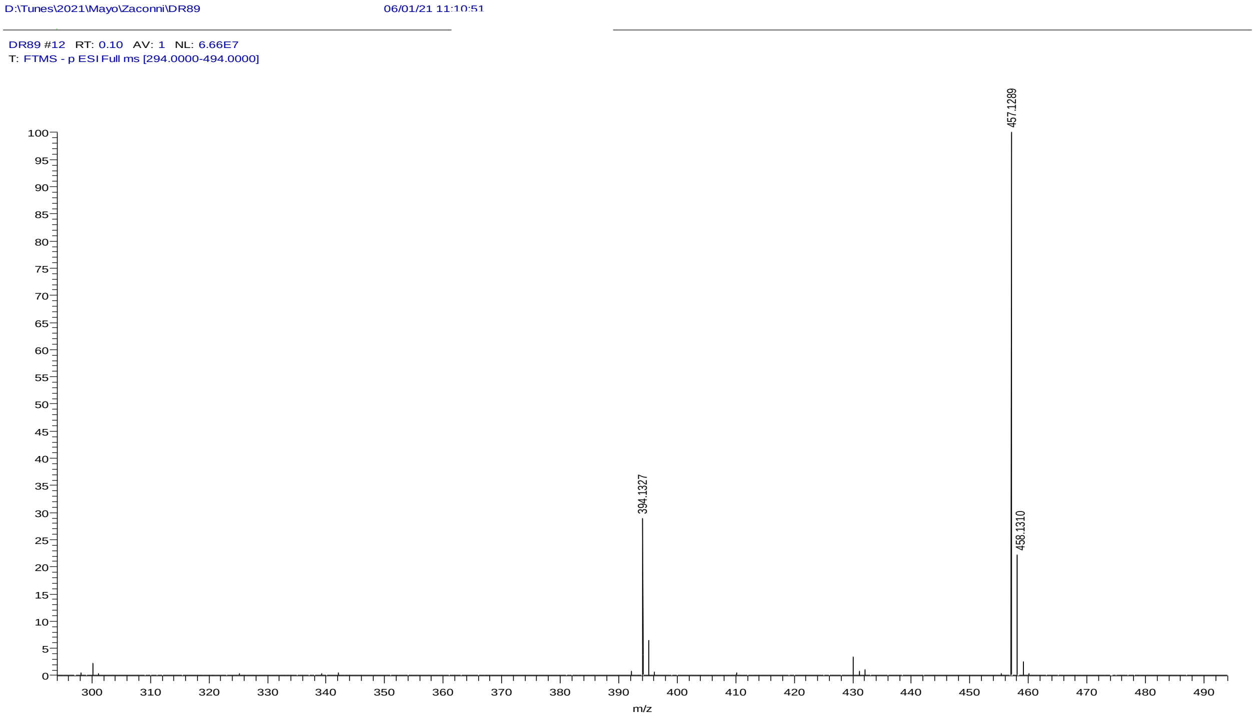

Figure S152. IR *N*-(3-fluoro-4-(3-oxomorpholino)phenyl)-2-(4-phenyl-1*H*-1,2,3-triazol-1-yl)acetamide (**8c**).

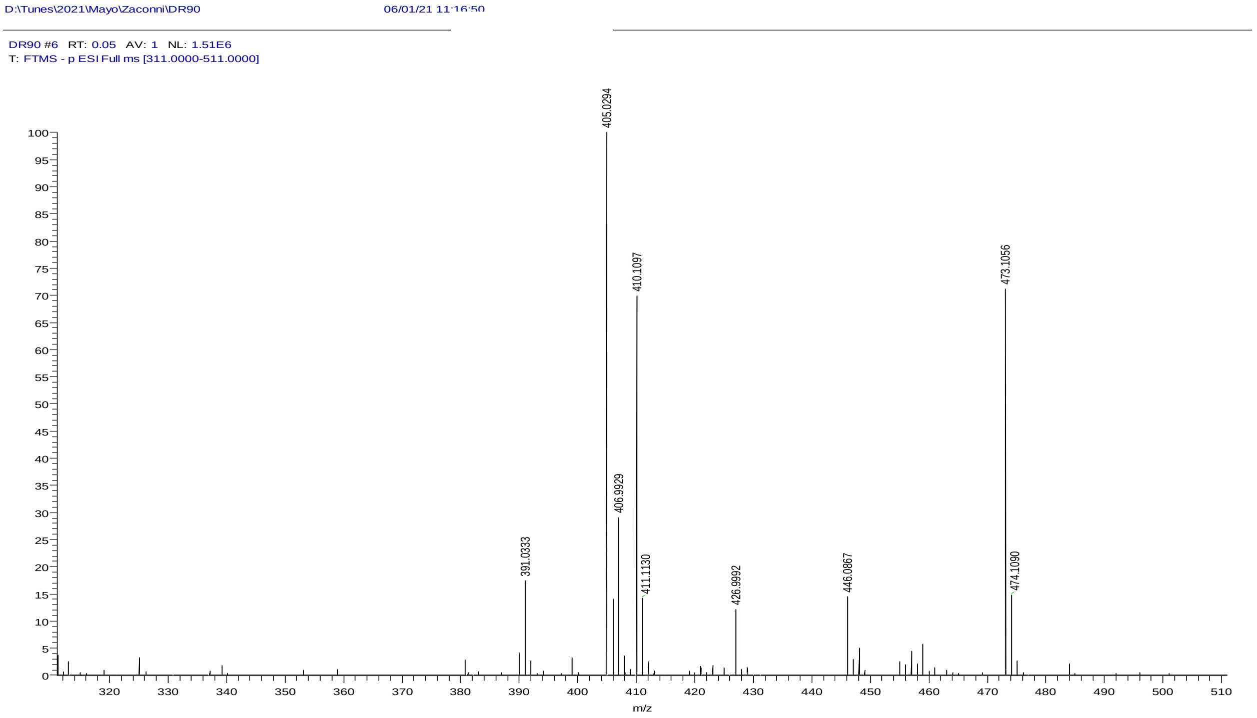

Figure S153. IR *N*-(3-fluoro-4-(3-oxothiomorpholino)phenyl)-2-(4-phenyl-1*H*-1,2,3-triazol-1-yl)acetamide (**8d**).

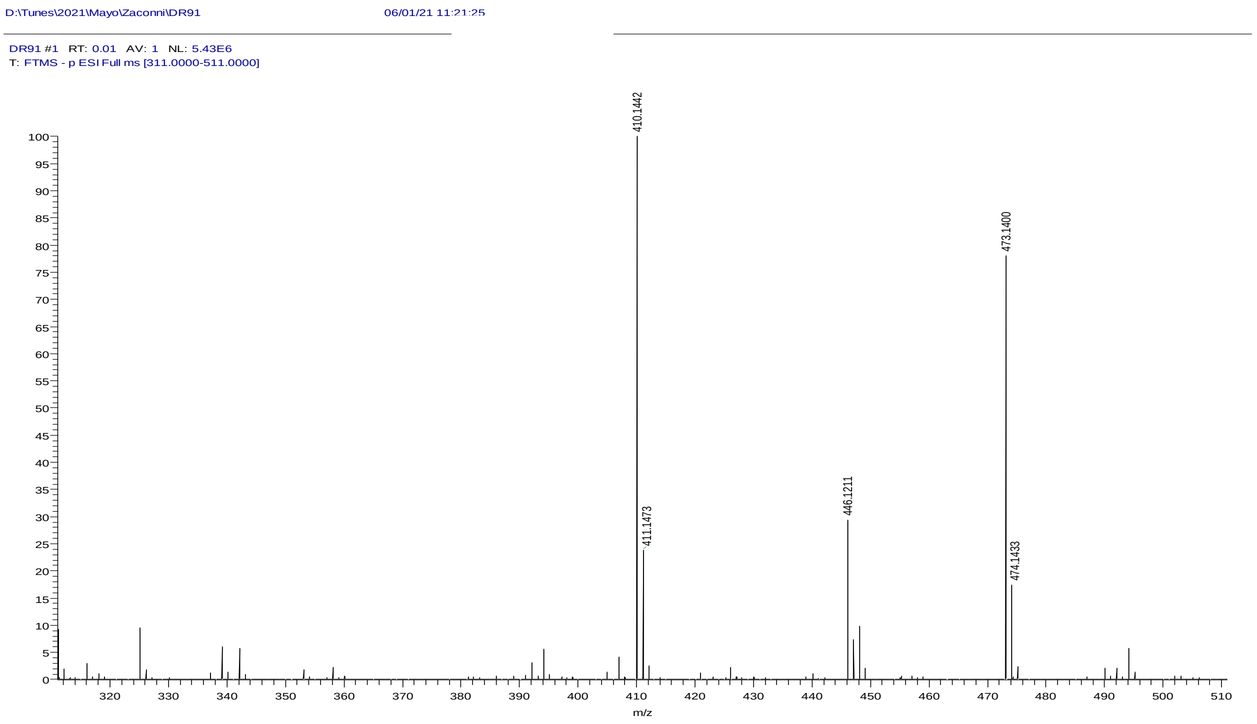

Figure S154. IR *N*-(3-fluoro-4-(2-oxopiperidin-1-yl)phenyl)-2-(4-(4-fluorophenyl)-1*H*-1,2,3-triazol-1-yl)acetamide (**8e**).

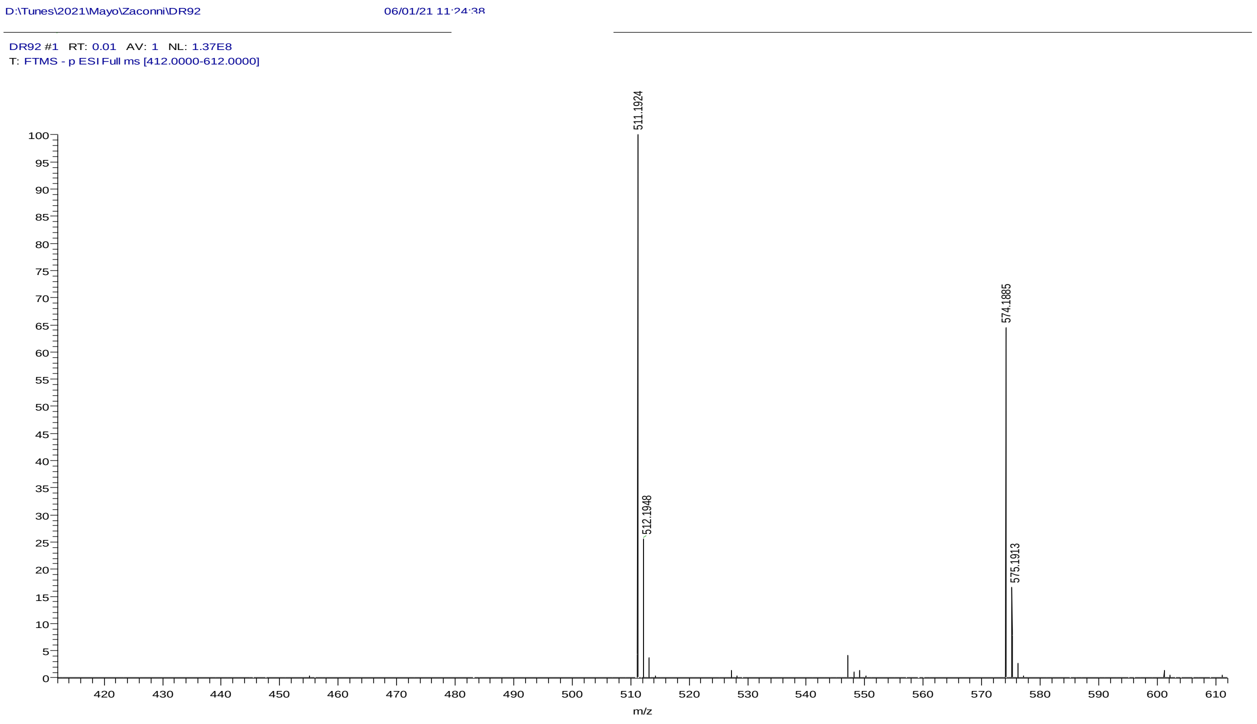

Figure S155. IR *tert*-butyl 4-(2-fluoro-4-(2-(4-(4-fluorophenyl)-1*H*-1,2,3-triazol-1-yl)acetamido)phenyl)-3-oxopiperazine-1-carboxylate (**8f**).

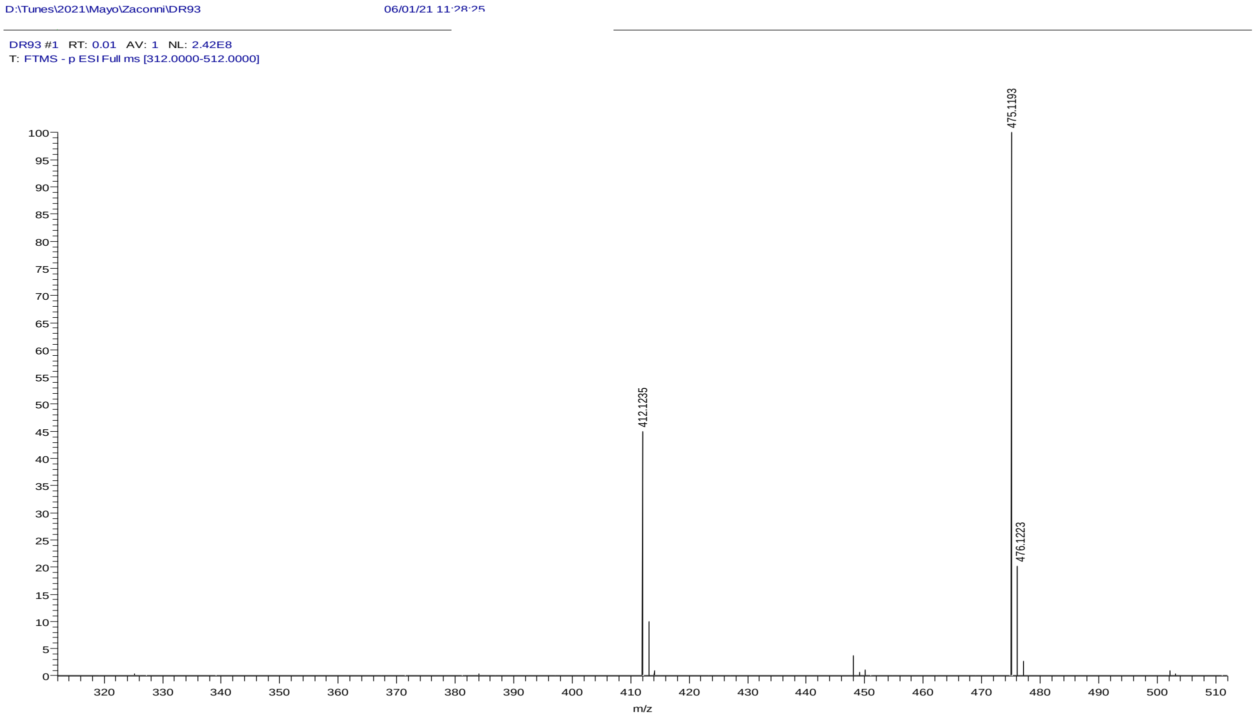

Figure S156. IR *N*-(3-fluoro-4-(3-oxomorpholino)phenyl)-2-(4-(4-fluorophenyl)-1*H*-1,2,3-triazol-1-yl)acetamide (**8g**).

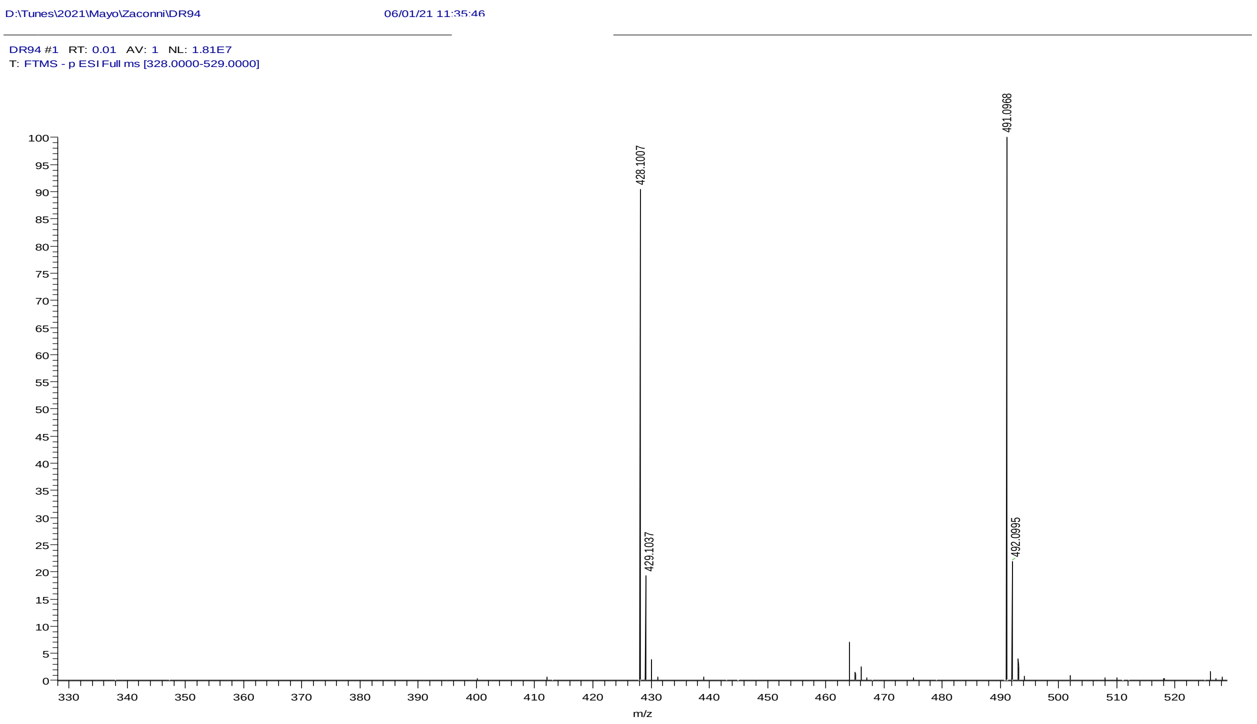

Figure S157. IR *N*-(3-fluoro-4-(3-oxothiomorpholino)phenyl)-2-(4-(4-fluorophenyl)-1*H*-1,2,3-triazol-1-yl)acetamide (**8h**).

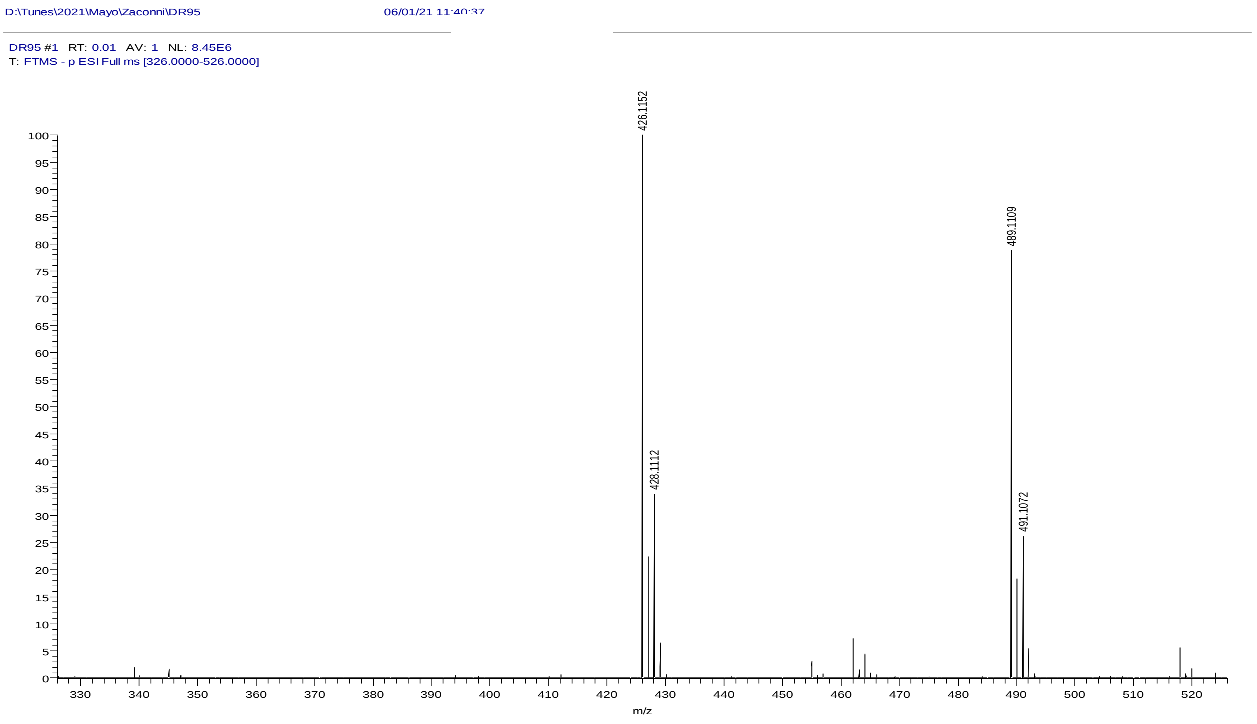

Figure S158. IR 2-(4-(4-chlorophenyl)-1*H*-1,2,3-triazol-1-yl)-*N*-(3-fluoro-4-(2-oxopiperidin-1-yl)phenyl)acetamide (**8i**).

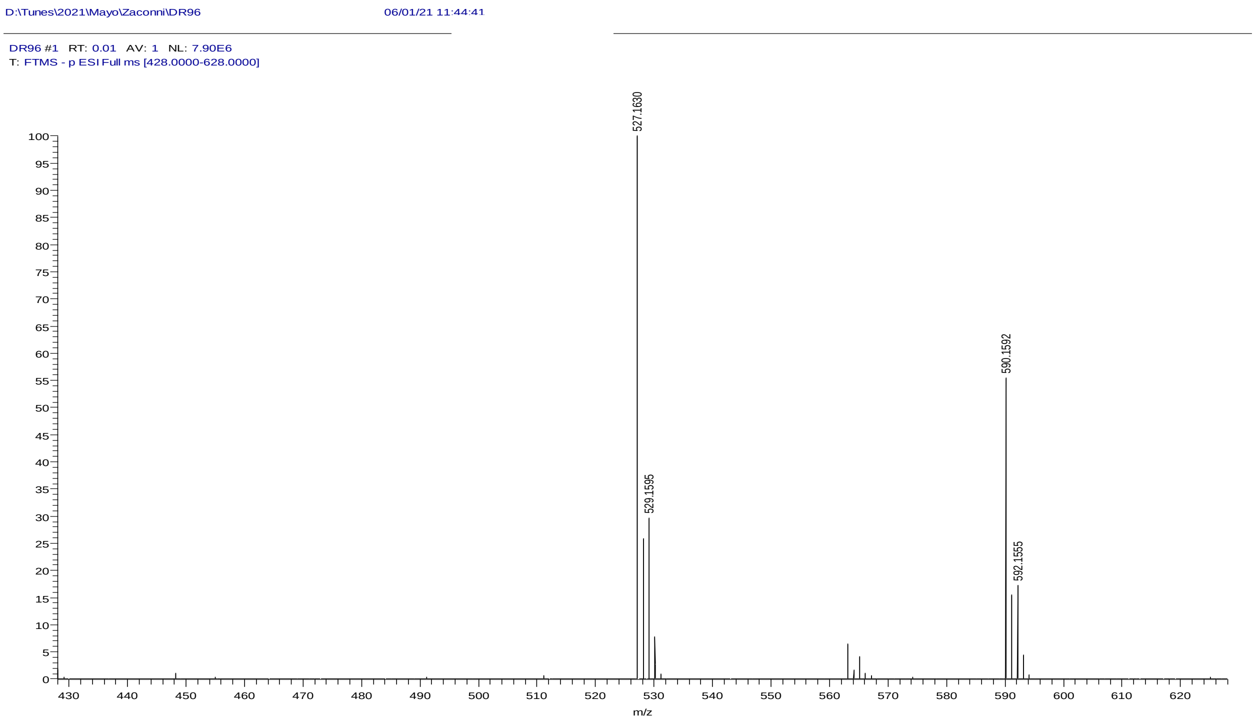

Figure S159. IR *tert*-butyl 4-(4-(2-(4-(4-chlorophenyl)-1*H*-1,2,3-triazol-1-yl)acetamido)-2-fluorophenyl)-3-oxopiperazine-1-carboxylate (**8j**).

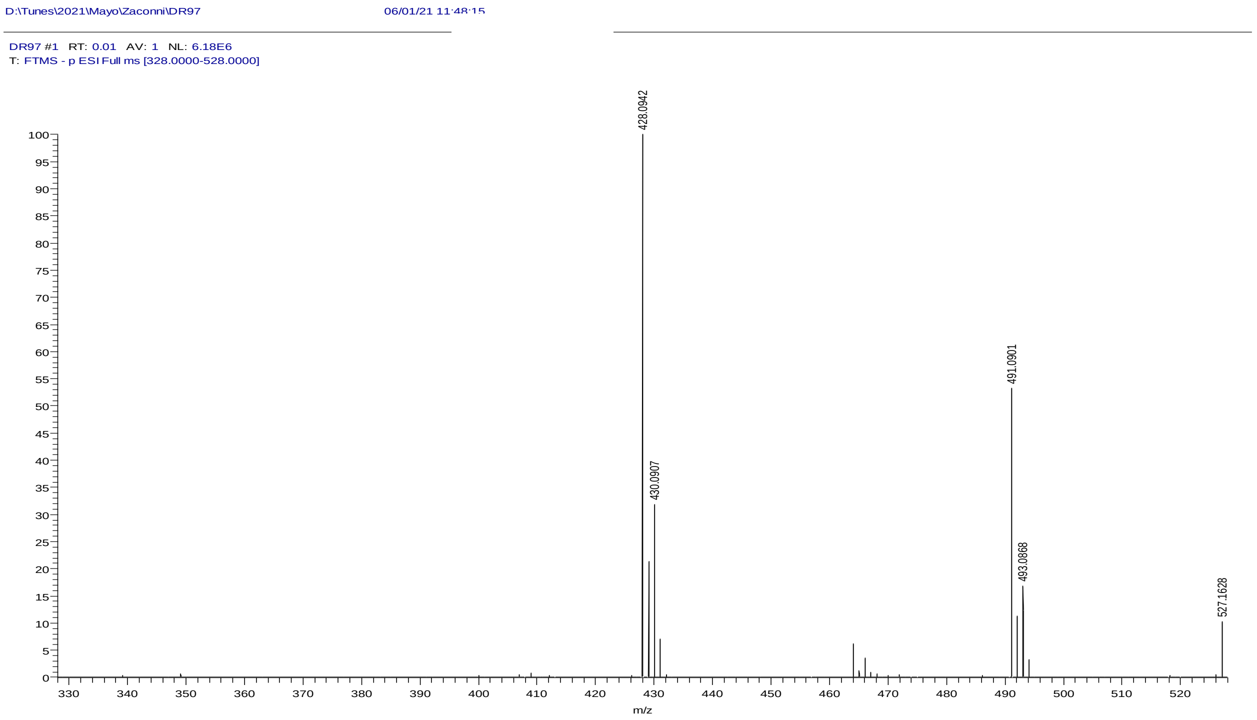

Figure S160. IR 2-(4-(4-chlorophenyl)-1*H*-1,2,3-triazol-1-yl)-*N*-(3-fluoro-4-(3-oxomorpholino)phenyl)acetamide (**8k**).

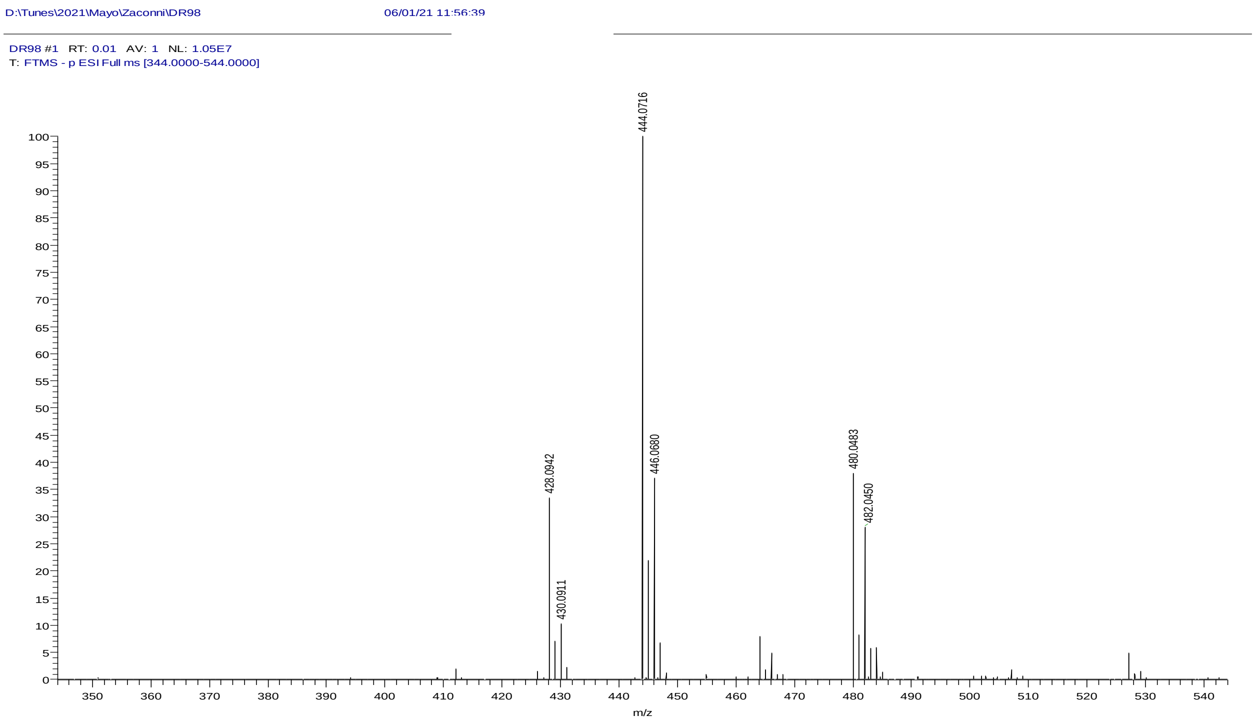

Figure S161. IR 2-(4-(4-chlorophenyl)-1*H*-1,2,3-triazol-1-yl)-*N*-(3-fluoro-4-(3-oxothiomorpholino)phenyl)acetamide (**8l**).

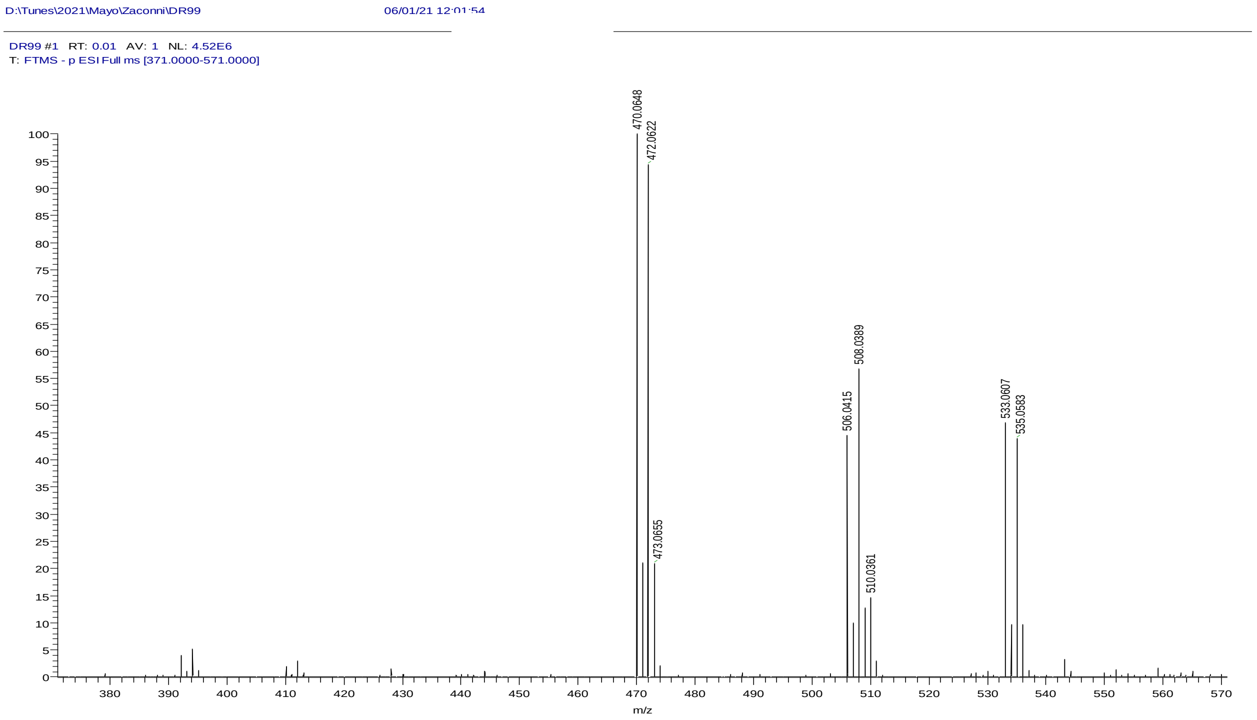

Figure S162. IR 2-(4-(4-bromophenyl)-1*H*-1,2,3-triazol-1-yl)-*N*-(3-fluoro-4-(2-oxopiperidin-1-yl)phenyl)acetamide (**8m**).

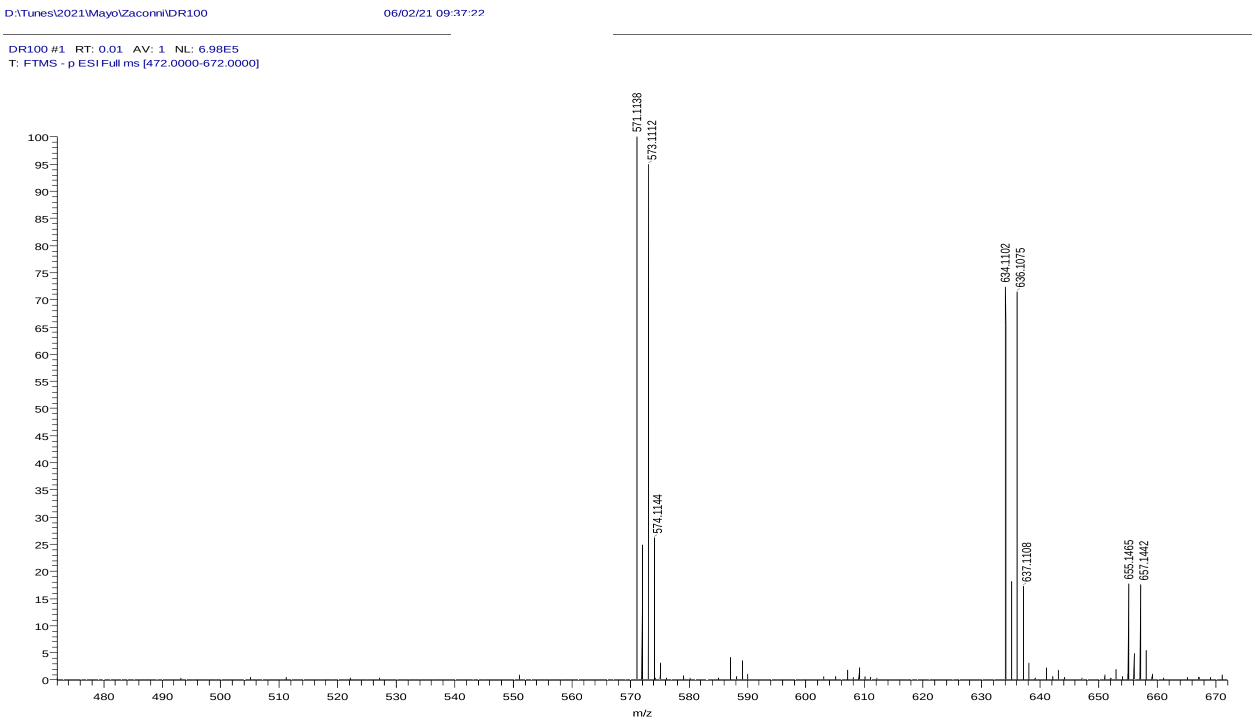

Figure S163. IR 2-(4-(4-bromophenyl)-1*H*-1,2,3-triazol-1-yl)-*N*-(3-fluoro-4-(2-oxopiperidin-1-yl)phenyl)acetamide (**8n**).

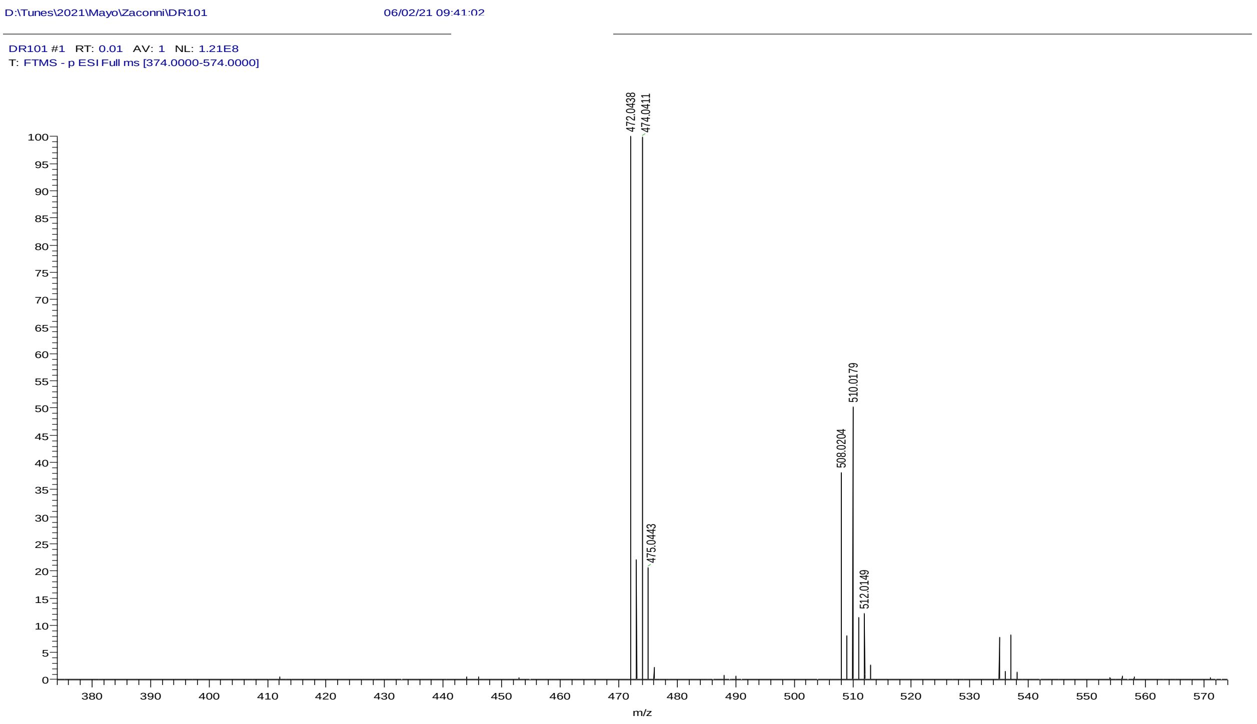

Figure S164. IR 2-(4-(4-bromophenyl)-1*H*-1,2,3-triazol-1-yl)-*N*-(3-fluoro-4-(3-oxomorpholino)phenyl)acetamide (**8o**).

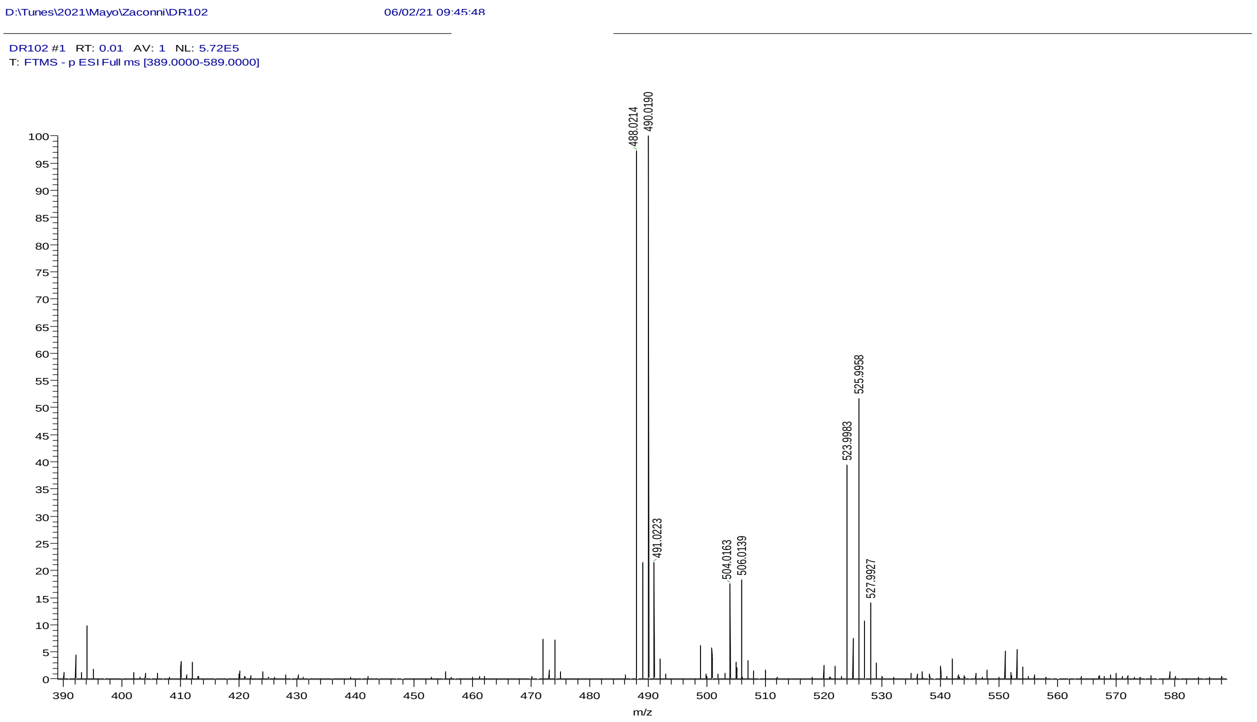

Figure S165. IR 2-(4-(4-bromophenyl)-1*H*-1,2,3-triazol-1-yl)-*N*-(3-fluoro-4-(3-oxothiomorpholino)phenyl)acetamide (**8p**).

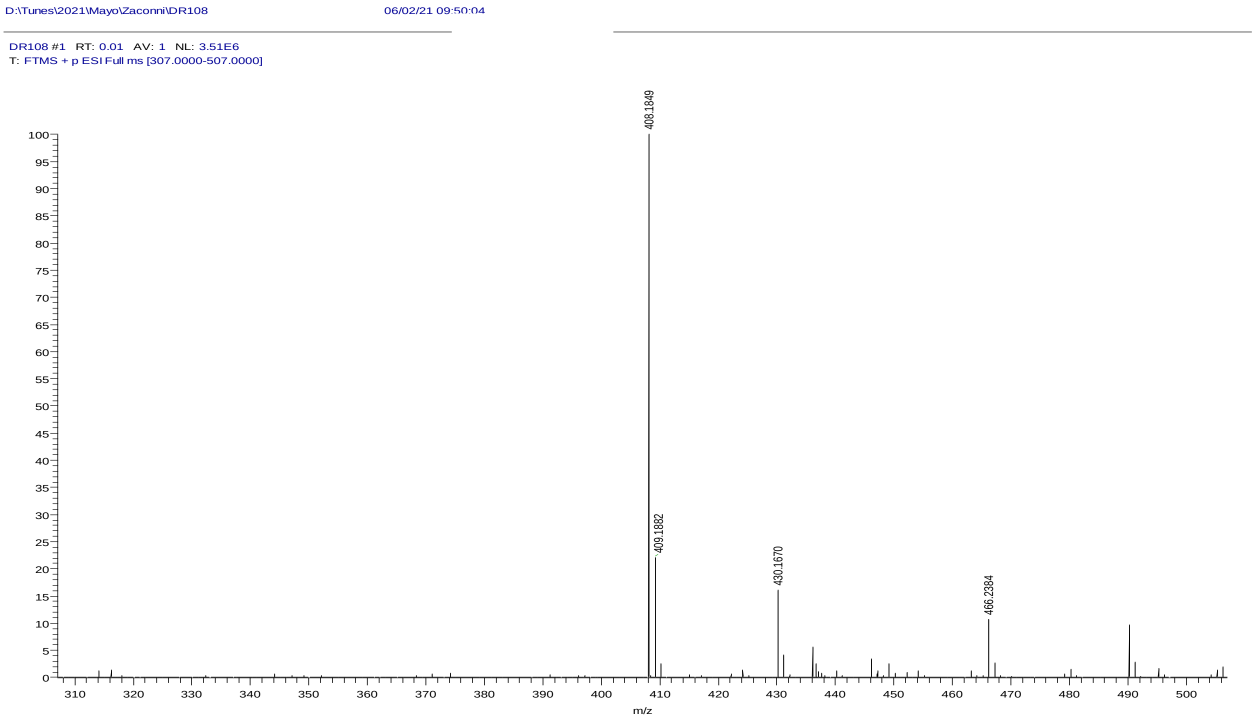

Figure S166. IR *N*-(3-fluoro-4-(2-oxopiperidin-1-yl)phenyl)-2-(4-(*p*-tolyl)-1*H*-1,2,3-triazol-1-yl)acetamide(**8q**).

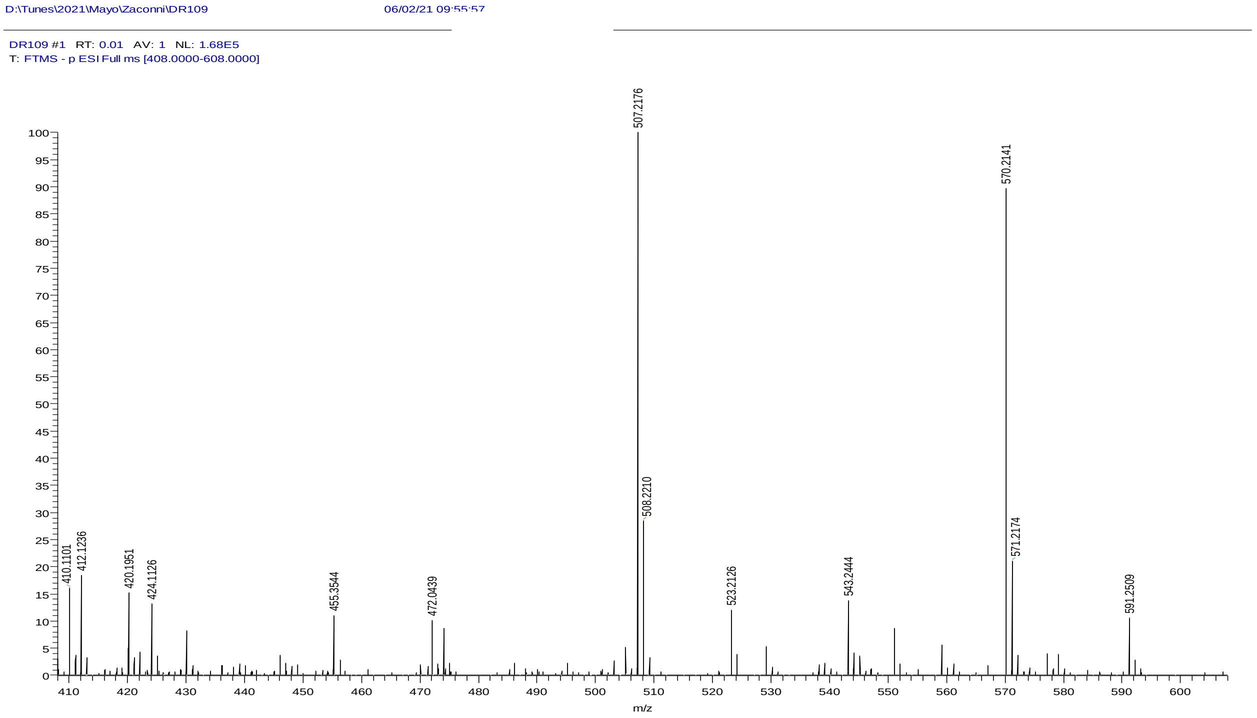

Figure S167. IR *tert*-butyl4-(2-fluoro-4-(2-(4-(*p*-tolyl)-1*H*-1,2,3-triazol-1-yl)acetamido)phenyl)-3-oxopiperazine-1-carboxylate (**8r**).

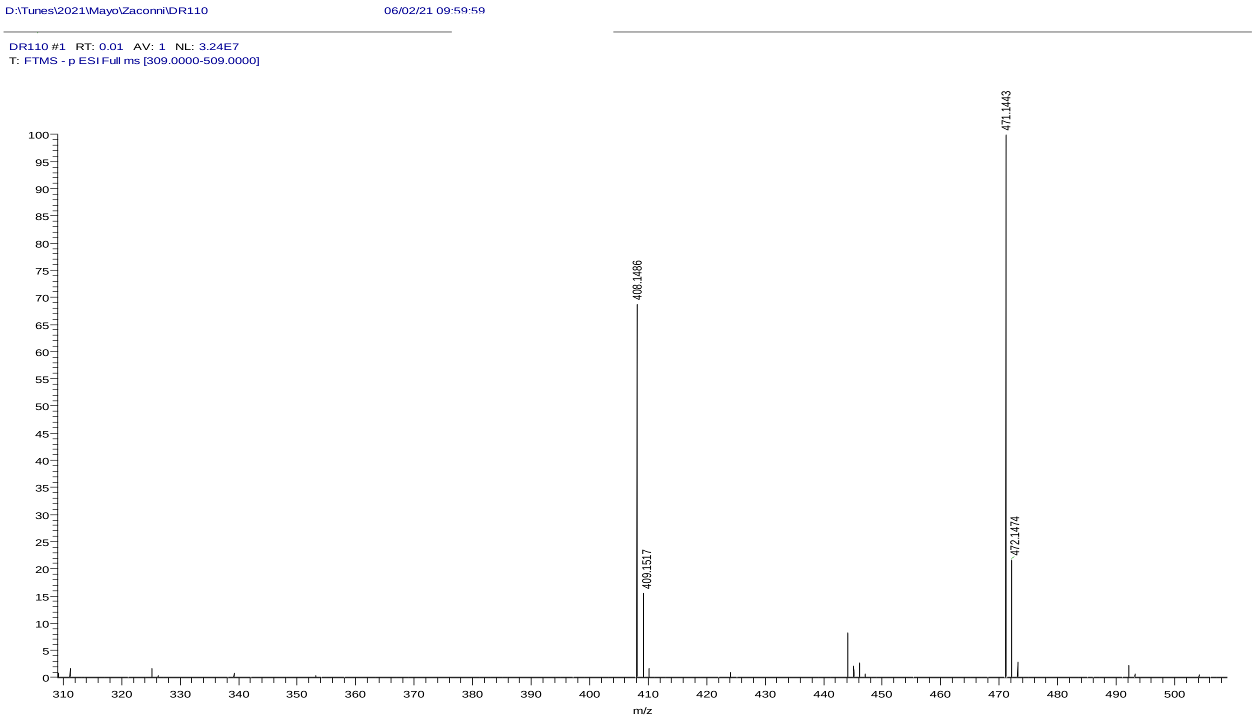

Figure S168. IR *N*-(3-fluoro-4-(3-oxomorpholino)phenyl)-2-(4-(*p*-tolyl)-1*H*-1,2,3-triazol-1-yl)acetamide (**8s**).

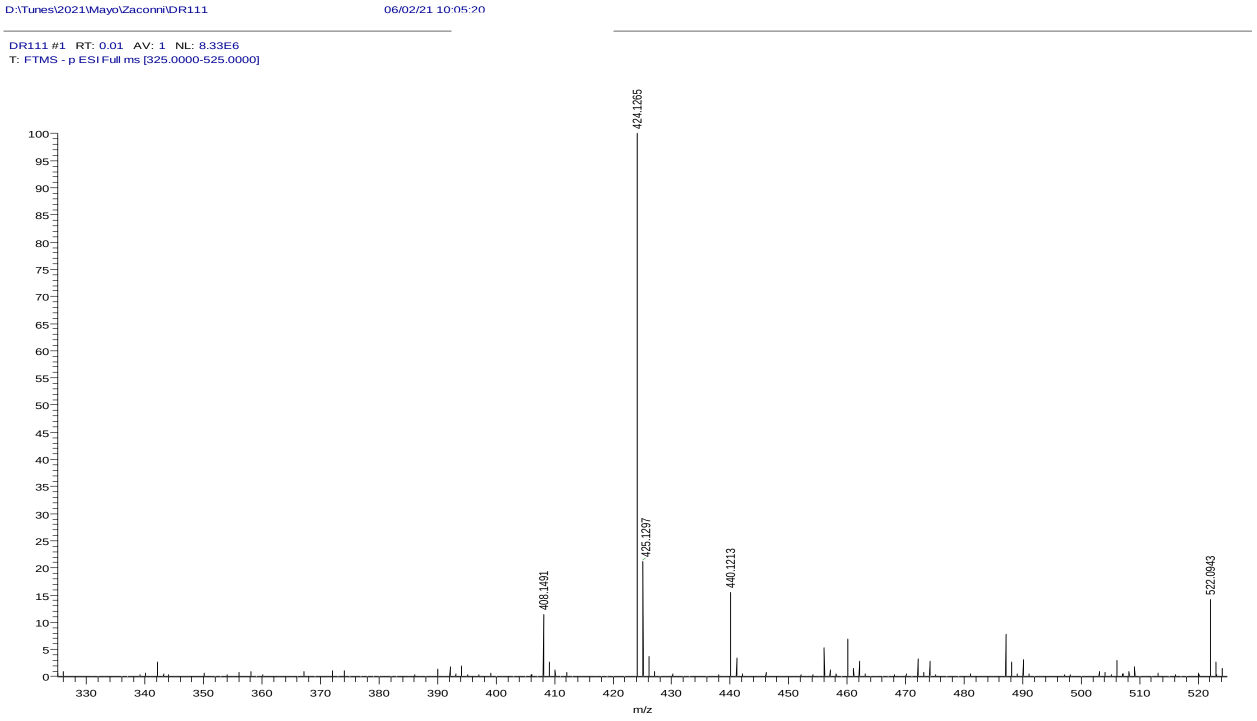

Figure S169. IR *N*-(3-fluoro-4-(3-oxothiomorpholino)phenyl)-2-(4-(*p*-tolyl)-1*H*-1,2,3-triazol-1-yl)acetamide (**8t**).

12. *In Silico* Pharmacokinetics Prediction

Compound	logPo/w	mol MW	H-Bonds Donors	H-Bonds Acceptors	logS	logBB	PMDCK	PHOA
**8a**	3.37	393.42	1.0	8.0	-5.89	-0.88	473.25	100.00
**8b**	4.03	494.52	1.0	10.5	-7.64	-1.52	212.50	95.19
**8c**	2.34	395.39	1.0	9.7	-4.84	-0.97	356.08	89.17
**8d**	3.33	411.45	1.0	8.5	-6.04	-0.85	748.56	95.84
**8e**	3.59	411.41	1.0	8.0	-6.29	-0.81	796.60	100.00
**8f**	4.27	512.52	1.0	10.5	-8.03	-1.44	369.45	83.30
**8g**	2.57	413.38	1.0	9.7	-5.20	-0.86	644.79	90.53
**8h**	3.56	429.44	1.0	8.5	-6.45	-0.78	1276.12	96.76
**8i**	3.85	427.87	1.0	8.0	-6.67	-0.77	1088.18	100.00
**8j**	4.52	528.97	1.0	10.5	-8.41	-1.40	502.85	84.80
**8k**	2.82	429.84	1.0	9.7	-5.56	-0.82	879.37	92.01
**8l**	3.82	445.90	1.0	8.5	-6.85	-0.76	1663.45	100.00
**8m**	3.92	472.32	1.0	8.0	-6.81	-0.78	1120.08	100.00
**8n**	4.60	573.42	1.0	10.5	-8.49	-1.36	568.10	85.62
**8o**	2.90	474.29	1.0	9.7	-5.67	-0.81	946.10	92.45
**8p**	3.90	490.35	1.0	8.5	-6.89	-0.70	1982.33	100.00
**8q**	3.67	407.45	1.0	8.0	-6.46	-0.91	471.17	100.00
**8r**	4.35	508.55	1.0	10.5	-8.19	-1.53	224.32	84.48
**8s**	2.63	409.42	1.0	9.7	-5.39	-1.02	344.80	90.64
**8t**	3.64	425.48	1.0	8.5	-6.60	-0.88	749.32	100.00

logPo/w = Predicted octanol/water partition coefficient. Recommended values −2.0–6.5. H-Bonds Donors = estimated number of hydrogen bonds that would be donated in an aqueous solution. Values are averages taken over a number of configurations, thus, they can be non-integer. H-Bonds Acceptors = Estimated number of hydrogen bonds that would be accepted in an aqueous solution. Values are averages taken over a number of configurations, thus, they can be non-integer. logS = Predicted aqueous solubility. Recommended values −6.5 to 0.5. logBB = Predicted brain/blood partition coefficient. Recommended values −3.0 to 1.2. logBB = Passive blood-brain partitioning values expressed in terms of logarithm. Predicted apparent MDCK cell permeability in nm/sec. MDCK cells are considered to be a good mimic for the blood-brain barrier. The predictions are for non-active transport. <25 poor, >500 great. PHOA= Predicted human oral absorption on 0 to 100% scale. Recommended values >80% is high <25% is poor.

13. References

(1) Alonso, F.; Moglie, Y.; Radivoy, G.; Yus, M. Click Chemistry from Organic Halides, Diazonium Salts and Anilines in Water Catalysed by Copper Nanoparticles on Activated Carbon. *Org. Biomol. Chem.* **2011**, *9* (18), 6385–6395. https://doi.org/10.1039/C1OB05735A.

(2) Mitrofanov, A. Y.; Murashkina, A. V; Martín-García, I.; Alonso, F.; Beletskaya, I. P. Formation of C–C, C–S and C–N Bonds Catalysed by Supported Copper Nanoparticles. *Catal. Sci. Technol.* **2017**, *7* (19), 4401–4412. https://doi.org/10.1039/C7CY01343D.

(3) Alonso, F.; Moglie, Y.; Radivoy, G.; Yus, M. Unsupported Copper Nanoparticles in the 1,3-Dipolar Cycloaddition of Terminal Alkynes and Azides. *European J. Org. Chem.* **2010**, *2010* (10), 1875–1884. https://doi.org/10.1002/ejoc.200901446.

(4) Coelho, A.; Diz, P.; Caamaño, O.; Sotelo, E. Polymer-Supported 1,5,7-Triazabicyclo[4.4.0]Dec-5-Ene as Polyvalent Ligands in the Copper-Catalyzed Huisgen 1,3-Dipolar Cycloaddition. *Adv. Synth. Catal.* **2010**, *352* (7), 1179–1192. https://doi.org/10.1002/adsc.200900680.

(5) Odlo, K.; Høydahl, E. A.; Hansen, T. V. One-Pot Synthesis of 1,4-Disubstituted 1,2,3-Triazoles from Terminal Acetylenes and in Situ Generated Azides. *Tetrahedron Lett.* **2007**, *48* (12), 2097–2099. https://doi.org/https://doi.org/10.1016/j.tetlet.2007.01.130.
